# Diels–Alder reactions and electrophilic substitutions with atypical regioselectivity enable functionalization of terminal rings of anthracene

**DOI:** 10.1038/s42004-020-00407-9

**Published:** 2020-11-06

**Authors:** Vinh Ngoc Huynh, Michael Leitner, Aditya Bhattacharyya, Lisa Uhlstein, Peter Kreitmeier, Patrick Sakrausky, Julia Rehbein, Oliver Reiser

**Affiliations:** 1 Institut für Organische Chemie, Universität Regensburg, Universitätsstr. 31, 93053 Regensburg, Germany; 2 University of Science, Vietnam National University, 227 Nguyễn Văn Cừ street, district 5, Ho Chi Minh City, Vietnam

**Keywords:** Reaction mechanisms, Density functional theory, Synthetic chemistry methodology

## Abstract

Reversing the regioselectivity of the renowned Diels–Alder reaction by overriding the usual thermodynamic and kinetic governing factors has always been a formidable challenge to synthetic organic chemists. Anthracenes are well-known to undergo [4 + 2]-cycloadditions with dienophiles at their 9,10-positions (central ring) over 1,4-positions (terminal ring) guided by the relative aromatic stabilization energy of the two possible products, and also by harboring the largest orbital coefficients of the highest occupied molecular orbital (HOMO) at the 9,10-positions. We, herein, report a 1,4-selective [4 + 2]-cycloaddition strategy of 9,10-unsubstituted anthracenes by installing electron-donating substituents on the terminal rings which is heretofore unprecedented to the best of our knowledge. The developed synthetic strategy does not require any premeditated engagement of the 9,10-positions either with any sterically bulky or electron-withdrawing substituents and allows delicate calibration of the regioselectivity by modulating the electron-donating strength of the substituents on the terminal rings. Likewise, the regioselective functionalization of the terminal anthracene ring in electrophilic substitution reactions is demonstrated. A mechanistic rationale is offered with the aid of detailed computational studies, and finally, synthetic applications are presented.

## Abstract (web summary)

Anthracenes typically undergo Diels-Alder reactions or electrophilic substitutions at the central ring, but can be biased towards reacting at the terminal ring by appropriate blocking groups at the 9,10-positions. Here an alternative approach involving electron-donating groups at the 1,4-positions induces regioselective reactivity at the terminal ring.

## Introduction

**Fig. 1:**
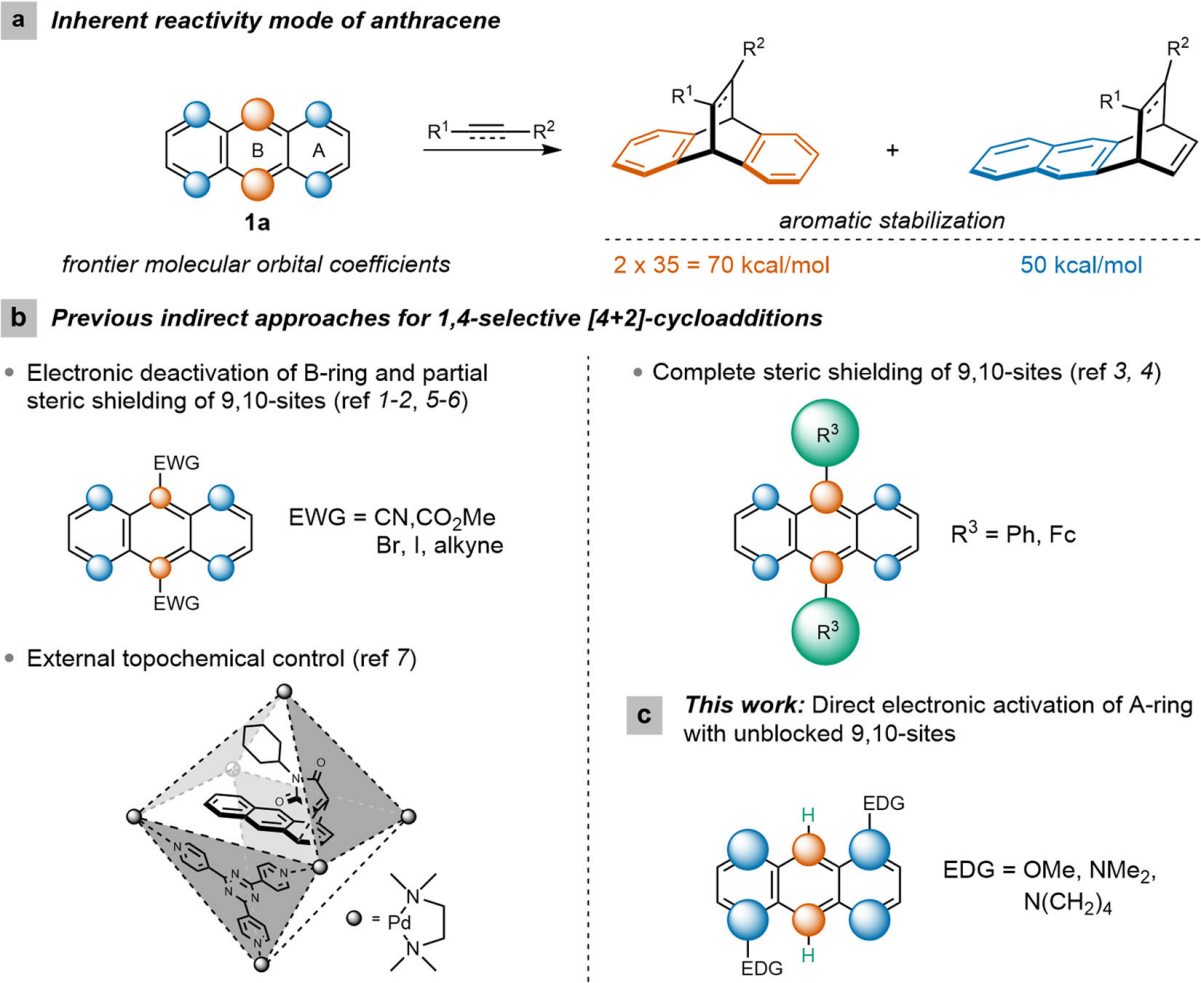
Reactivity profile of anthracenes. (**a**) Inherent reactivity of anthracene with dienophiles. The selectivity favoring the 9,10- vs. the 1,4-positions is governed by both thermodynamic (aromatic stabilization energy) and kinetic (molecular orbital coefficients) factors. (**b**) Previous approaches have been delineated wherein 1,4-selectivity could be achieved by deactivation of the B-ring either by electronic and steric maneuvers at the 9,10-positions or by exerting topochemical control by carrying out the reactions inside metal-organic cage-like structures. (**c**) The present approach entails direct activation of the A-ring by installing one or two electron-donating groups at 1- or 1,5-positions. Molecular orbital coefficients at 9,10-positions and 1,4,5,8-positions have been shown in vermillion and blue spheres, respectively (not to scale). The bluish-green spheres denote the steric shielding of the 9,10-positions by substituents R^3^. EWG, electron-withdrawing group; Fc, ferrocenyl, EDG, electron-donating group.

The [4 + 2]-cycloaddition of a conjugated diene with 4 π-electrons and a dienophile (an alkene or an alkyne) with 2 π-electrons—commonly known as the Diels–Alder reaction—is one of the most emblematic pericyclic reactions and is deservedly celebrated due to its synthetic reliability and atom-economic approach for the facile construction of various complex 6-membered ring systems in a regio- and stereoselective fashion. Being an easily available conjugated π-electron-rich carbocyclic system, anthracene (**1a**) has been widely exploited as a classic diene in Diels–Alder reactions wherein its chemical reactivity and transformational effectiveness are subsidized by the partial loss of aromaticity. Extensive synthetic and mechanistic studies reveal that the natural preference for [4 + 2]-cycloadditions of unsubstituted anthracene is at its 9,10-positions^1,2^. The selectivity is governed by thermodynamic factors given that the twofold stabilization energy of the benzene moieties (2 × 35 = 70 kcal/mol) is greater compared to that of the naphthalene moiety (50 kcal/mol) as well as the kinetic factor for having the largest frontier molecular orbital coefficients at the 9,10-positions in the Highest Occupied Molecular Orbitals (HOMO) of **1a** (Fig. 1a).

Considering the significance of the transformation, tuning the regioselectivity of the Diels–Alder reaction of anthracenes is an attractive problem. However, until today, only very few approaches have been successful in indirectly circumventing the inherent 9,10-preference of anthracenes in [4 + 2]-cycloaddition reactions and almost all of these studies relied on the premeditated engagement of the 9,10-positions either sterically or electronically. Installation of voluminous and/or electron-withdrawing substituents at the 9- and 10-positions indirectly deactivates the central B-ring by steric and electronic means, thereby allowing the formation of the corresponding cycloadducts with the terminal A-ring (Fig. 1b). For example, 9,10-dicyanoanthracene in the reaction with benzyne furnishes a 1:1 mixture of the corresponding 1,4- and 9,10-adducts, albeit only in low yield (8%)^3^. Moreover, in the total synthesis of molecular gyroscopes with triptycyl frames, Garcia-Garibay et al. observed the formation of 1,4-cycloadducts as byproducts (maximal ratio 9,10/1,4 = 1:1) during the Diels-Alder reaction of anthracenes bearing alkyne groups in 9-position with benzynes^4^. Likewise, sterically more demanding 9,10-diphenyl- or 9,10-diferrocenyl-substituted anthracenes were found to yield exclusively the corresponding A-ring adducts with dimethyl acetylenedicarboxylate (DMAD) in 50% and 38% yield, respectively^5,6^. More recently, a 1,4-selective cycloaddition with sterically bulky *N-*2,6-difluorophenylmaleimide^7^ or with *N*-substituted maleimides activated by superstoichiometric amounts of AlCl_3_ was reported with anthracene derivatives^8^. However, yet again blocking of both, the 9- and/or the 10-position by ester, halide, and/or phenyl groups was necessary.

The only example so far that is reported to be exclusively 1,4-selective with an anthracene moiety unsubstituted at the 9,10-positions is the reaction between anthracene (**1a**) itself and *N*-cyclohexylmaleimimde confined as an inclusion complex of a supramolecular octahedral organopalladium host^9^. It was assumed that the unusual regioselectivity stemmed from external topochemical control by the way the substrates bind to the host which made it geometrically impossible to attack the 9,10-positions with the dienophile (Fig. 1b). More recently, an anthracene derivative with fused carbocyclic moieties in 2,3- and 6,7-positions was found to undergo a cycloaddition at both 9,10- and 1,4-positions (ratio 9,10/1,4 = 1:2) with 4,5-dimethoxybenzyne in 33% yield^10^. The unusual reactivity was explained by the electronic activation of the outer rings that outweighs the steric hindrance.

Motivated by this literature void, we wondered whether the A-ring of the anthracene moiety could be sufficiently electronically enriched by placing donor substituents on it so that the corresponding 1,4-cycloadducts would directly form by the reaction with electron-deficient dienophiles without the necessity of blocking the 9,10-positions. Gratifyingly, we could successfully achieve regioselective functionalization of the terminal rings of 9,10-unsubstituted anthracenes by placing sufficiently strong electron-donating substituents at the 1- or 1,5-positions which exert their effects by imposing largest orbital coefficients in the HOMO at the 1,4-positions of the anthracene moiety making the transformations kinetically favored with highly asynchronous transition states leading to the corresponding 1,4-cycloadducts and substitution products. Synthetic valorization of the 1,4-cycloadducts has also been demonstrated. Herein, we describe our results in detail.

## Results and Discussion

### [4 + 2]-Cycloaddition with olefins

**Fig. 2:**
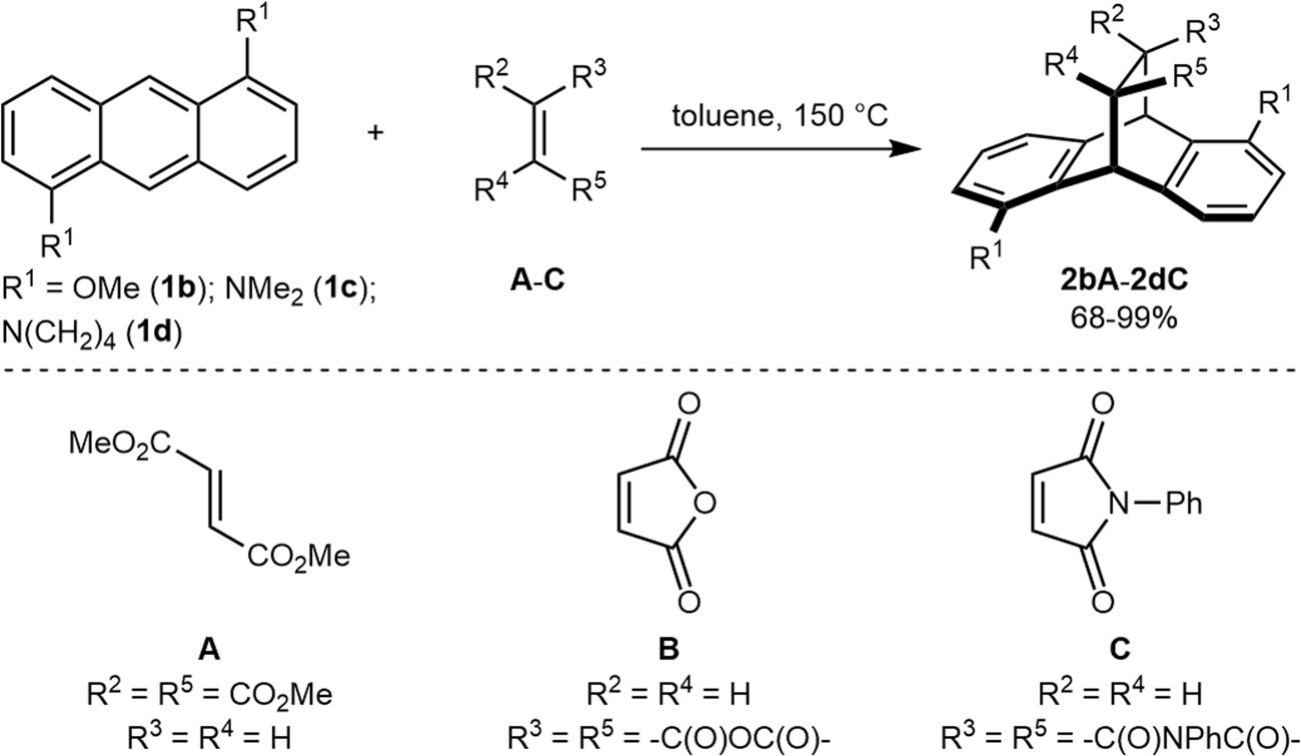
[4 + 2]-Cycloadditions of anthracenes with olefins. [4 + 2]-Cycloaddition reactions of electron-rich disubstituted anthracenes **1b**–**1d** with dimethyl fumarate (**A**), maleic anhydride (**B**) and *N*-phenylmaleimide (**C**) take place at 9,10-positions.

To assess the validity of our hypothesis, we synthesized (for details see Supplementary Methods) and employed 1,5-dimethoxyanthracene (**1b**) in a series of thermal [4 + 2]-cycloaddition reactions with a range of olefinic dienophiles such as dimethyl fumarate (**A**), maleic anhydride (**B**) and *N*-phenylmaleimide (**C**). However, in every case, we only observed the formation of 9,10-cycloadducts **2bA**–**C** in 95–99% yields (Supplementary Table 1). Intending to further increase the π-electron density on the A-ring, we subsequently synthesized dimethylamino or dipyrrolidino 1,5-disubstituted anthracenes (**1c**,**d**, for details, see Supplementary Methods) and inspected the outcome of the [4 + 2]-cycloaddition reactions with **A**–**C** under thermal conditions. Again, in all the cases, only the 9,10-cycloadducts **2cA**–**C** and **2dA**–**C** formed exclusively in 68–98% yields (Fig. 2, Supplementary Table 1).

### [4 + 2]-Cycloaddition with alkynes

**Fig. 3:**
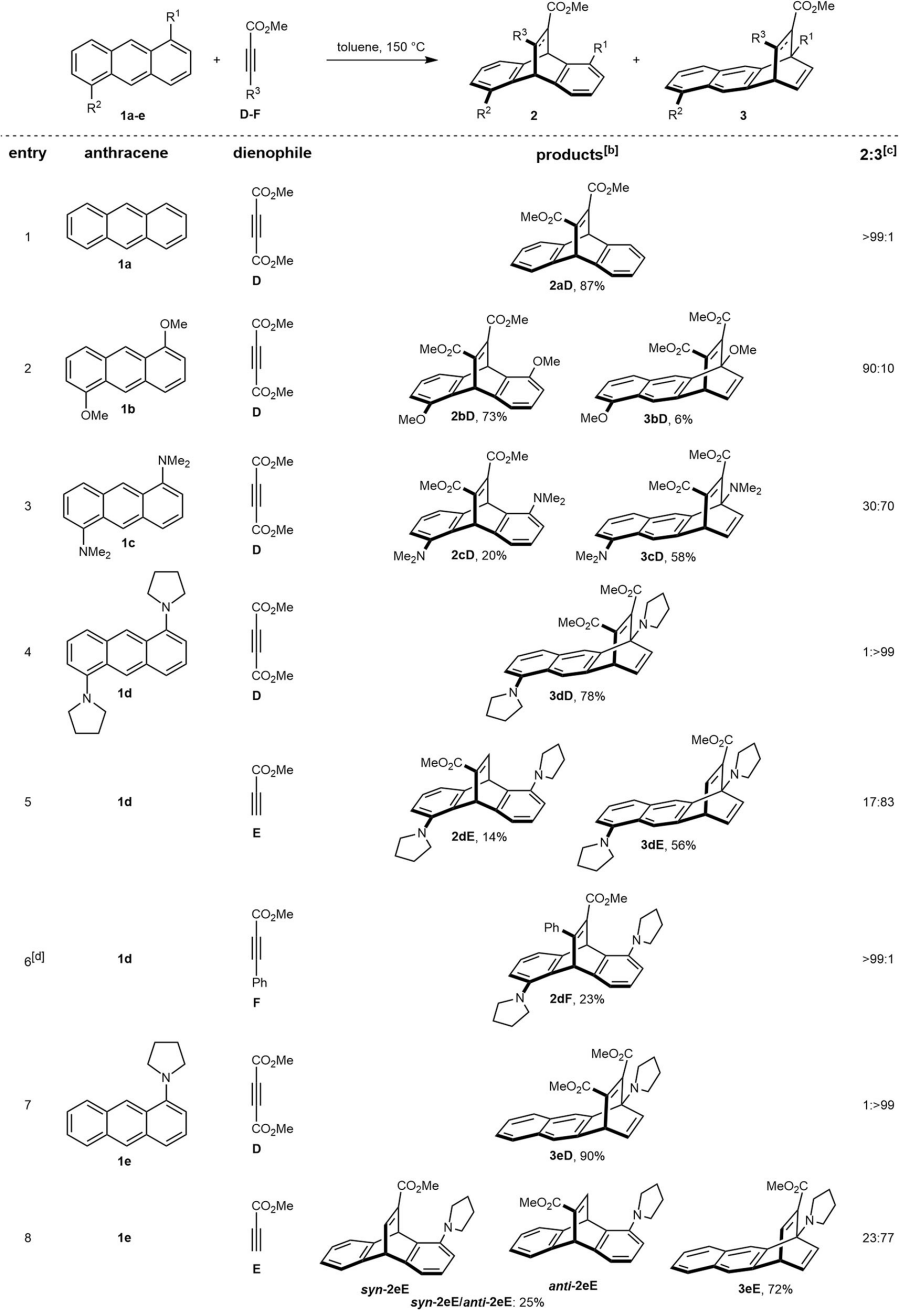
Substrate scope. Diels-Alder reactions between 1,5-disubstituted anthracenes and alkynes **D-F**.^[a]^ [a] Anthracene (**1a–e**, 1.0 equiv) and dienophile (**D–E**, 1.1 equiv) in toluene (0.5 M) in a sealed tube at 150 °C. [b] Isolated yield of **2** and **3**. [c] Determined by ^1^H NMR. [d] 5.0 equiv of dienophile **F** was used.

**Fig. 4:**
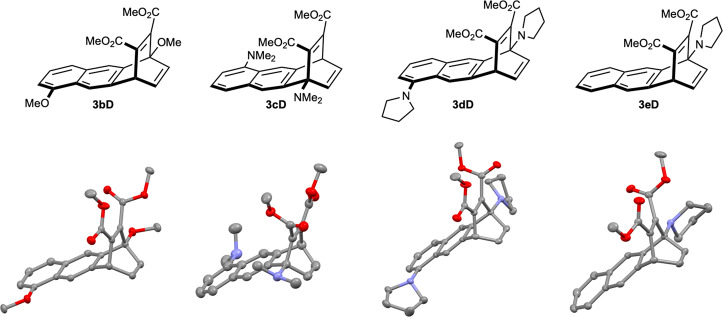
X-ray crystallographic studies. Crystal structures of **3bD**, **3cD**, **3dD** and **3eD** (50% thermal probability).

Next, we turned our attention to evaluating alkyne dienophiles such as dimethylacetylene dicarboxylate (DMAD, **D**) which are also known for being exclusively 9,10-selective for unsubstituted anthracene^11^ (**1a**, Fig. 3, entry 1). When **1b** was reacted with **D** under thermal reaction conditions, we pleasingly observed the first indication of the change in the trend of the regioselectivity of the transformation, as 6% of the corresponding 1,4-cycloadduct (**3bD**) formed alongside the formation of the usual 9,10-cycloadduct in 73% yield (Fig. 3, entry 2). The 1,4-cycloadduct **3bD** was isolated and unambiguously characterized by spectroscopic and single-crystal X-ray analysis (Fig. 4). Subsequently, when dimethylamino-group bearing 1,5-disubstituted anthracene **1c** was subjected to the [4 + 2]-cycloaddition with DMAD (**D**) under similar thermal reaction conditions, the corresponding 1,4-cycloadduct **3cD** turned out to be the major product (58% yield) dominating over the usual 9,10-cycloadduct **2cD** which was obtained in only 20% yield (Fig. 3, entry 3). The structure of the 1,4-cycloadduct **3cD** was again confirmed by single-crystal X-ray analysis (Fig. 4). Inspired by the observation that the donor strength of the substitutions on the A-ring indeed had an effect on the regioselectivity of [4 + 2]-cycloaddition reactions of anthracene derivatives, we next investigated the dipyrrolidino 1,5-disubstituted anthracene (**1d**) following the lead of Zipse et al.^12,13^ who demonstrated that a pyrrolidine substituted pyridine is more nucleophilic than dimethylaminopyridine (DMAP). When **1d** was engaged in the thermal [4 + 2]-cycloaddition with **D** under similar reaction conditions, we were pleased to find that the reaction yielded the 1,4-adduct **3dD** exclusively in 78% yield (Fig. 3, entry 4), representing, to our knowledge, the first example for a 1,4-selective [4 + 2]-cycloaddition of an anthrancene derivative in which the 9,10-positions are not blocked with substituents or shielded by any other means. Interestingly, when methyl propiolate (**E**) bearing a single ester group on the alkyne functionality was employed as the dienophile and reacted with **3d**, a drop in regioselectivity was observed and the corresponding 1,4-cycloadduct **3dE** was obtained as a single isomer in 56% yield along with the formation of the corresponding 9,10-cycloadduct **2dE** in 14% yield (Fig. 3, entry 5). The regioselectivity was again found to be entirely reversed when methyl phenylpropiolate (**F**) was reacted with **1d** and the corresponding 9,10-cycloadduct **2dF** formed exclusively, albeit with poor yield even after 72 h (23%, Fig. 3, entry 6).

Having validated our initial hypothesis and after establishing a definite relationship between the donor strength of the substituents at 1,5-positions and the regioselective outcomes of the [4 + 2]-cycloadditions of anthracenes with alkene and alkyne dienophiles, we next intended to investigate the extent of the electron-donating effect on the regioselectivity of the transformations. Accordingly, we synthesized the monosubstituted pyrrolidino anthracene derivative **1e** (for details see Supplementary Methods). Again, when **1e** was subsequently employed in the thermal [4 + 2]-cycloaddition with alkene dienophiles **A–C**, the corresponding 9,10-cycloadducts **2eA-C** exclusively formed in every case in 95–99% yields (Supplementary Table 1). Satisfyingly, when DMAD (**D**) was employed as the dienophile, we observed the selective formation of the 1,4-cycloadduct **3eD** in excellent yield (90%, Fig. 3, entry 7). Likewise, methyl propiolate (**E**) yielded preferentially the corresponding 1,4-cycloadduct **3eE** as a single regioisomer in 72% yield along with the formation of the corresponding 9,10-cycloadducts as a *syn*/*anti* isomeric mixture (25%, Fig. 3, entry 8). In contrast, methyl phenylpropiolate (**F**) did not undergo any cycloaddition reaction with **1e**. The structures of the 1,4-cycloadducts **3b–eD** were unambiguously assigned by nuclear magnetic resonance (NMR) spectroscopy and were confirmed by single-crystal X-ray diffraction analysis (Fig. 4, for more details, see Supplementary Data 2–5). It is noteworthy to mention that all 1,4- and 9,10-cycloadducts were examined at high-temperature reaction conditions (reflux, 24 h, 160 °C); however, no crossover between the products was observed in any case indicating no retro-Diels-Alder reactions from these products.

### Electrophilic substitutions

**Fig. 5:**
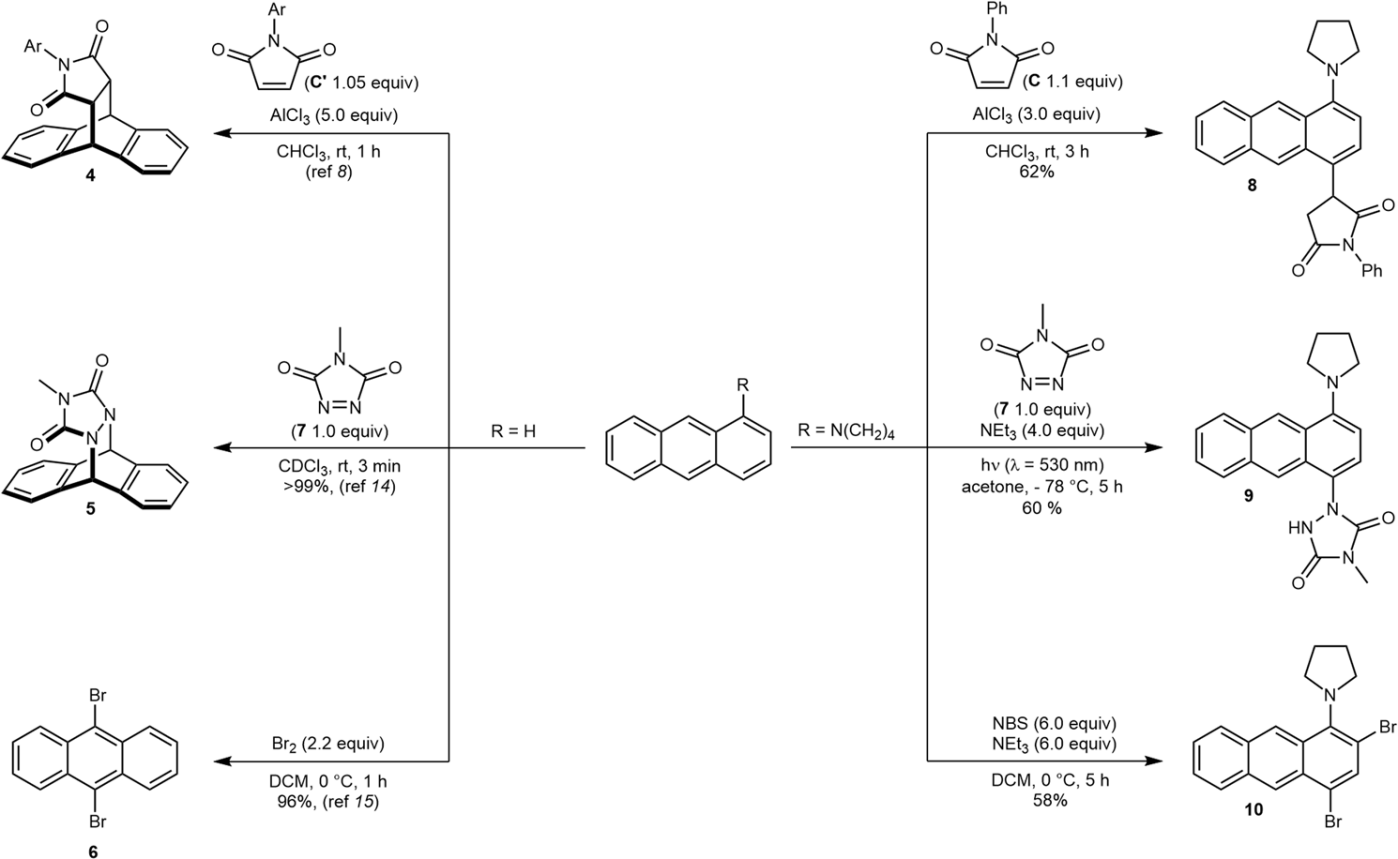
Comparison of electrophilic substitutions of anthracenes. Electron-donating substitution effect on the reactivities of anthracenes was also evident when electrophilic substitution reactions were compared between unsubstituted anthracene (**1a**) and pyrrolidine anthracene **1e** with different electrophiles such as *N*-arylmaleimides, MTAD, and bromine.

The effect of the pronounced electronic perturbations exerted by the amine substitution in 1/5-position favoring their terminal ring functionalization was also evident for the reactions with various electrophiles (Fig. 5). While unsubstituted anthracene is known to undergo a [4 + 2]-cycloaddition reaction at the 9,10-positions with *N*-arylmaleimides (**C**′) in the presence of superstoichiometric amounts of AlCl_3_^8^, anthracene **1e** exclusively furnished the Friedel-Crafts type product **8** in 62% yield under similar reaction conditions. *N*-Methyl-1,2,4-triazoline-3,5-dione (MTAD, **7**) has been reported to react with anthracene **1a** in the dark to produce the corresponding 9,10-cycloadduct **5** within three minutes quantitatively^14^, while under the same reaction conditions no conversion was observed with **1e** even after 24 h. In contrast, activation of MTAD by irradiation with a green light-emitting diode (LED, λ = 530 nm) and subsequent reaction with **1e** led to the formation of a *para*-Friedel-Crafts type product **9** in 60% yield wherein again the terminal ring was functionalized exclusively. Analogous to the cycloaddition reactions, electrophilic aromatic substitution of anthracenes generally takes place in the central B ring as a consequence of maximizing the aromatic stabilization energies in the transition state^15–17^. In contrast, bromination of anthracene **1e**, using an excess of *N*-bromosuccinamide (NBS) and NEt_3_ as an acid scavenger, only occurred at the terminal A ring giving rise to *ortho-para* brominated anthracene **10** in 58% yield.

### Functionalizations of 1,4-cycloadduct

**Fig. 6:**
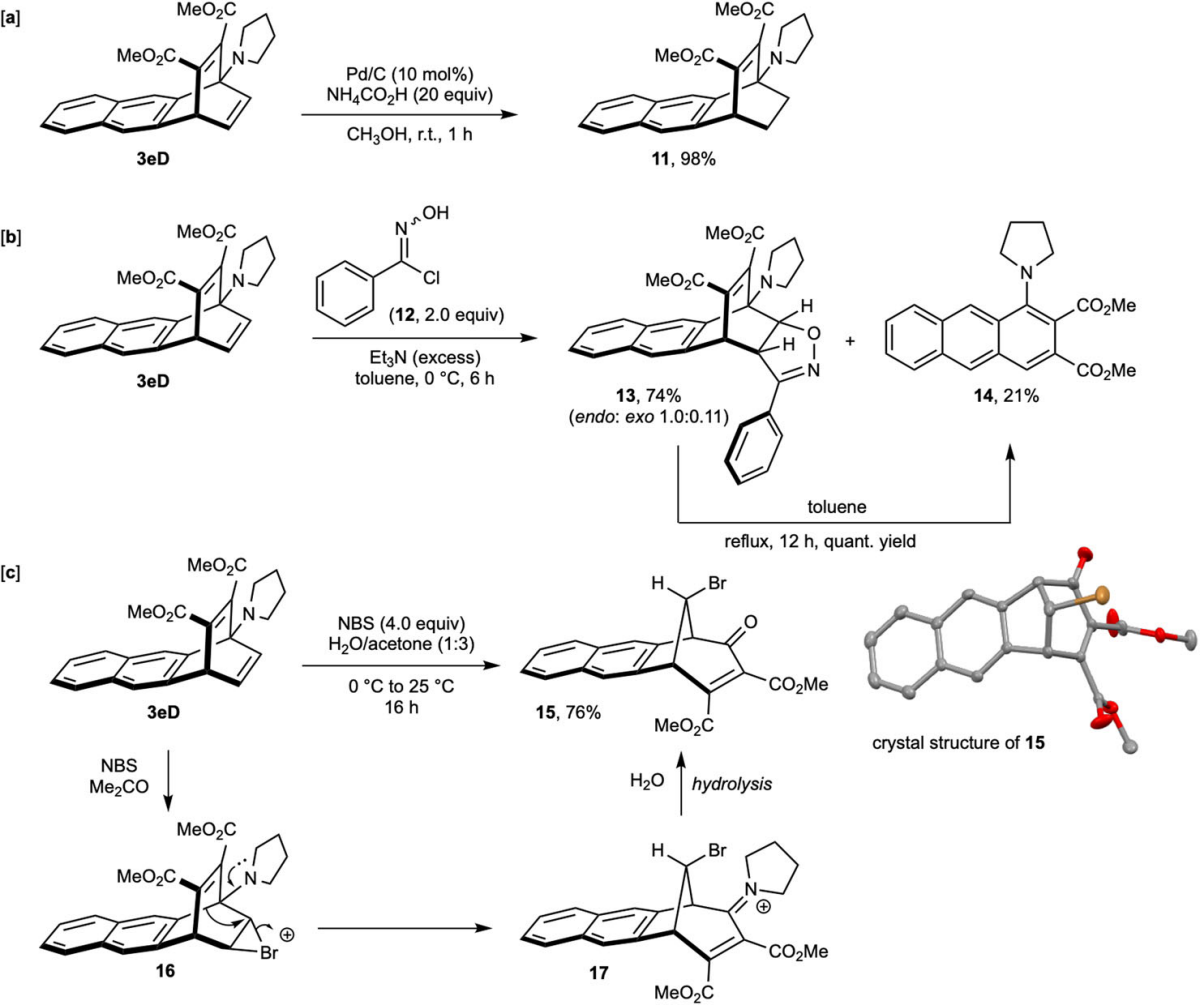
Synthetic explorations of the 1,4-cycloadduct, 3eD. (**a**) Hydrogenation with Pd/C and ammonium formate. (**b**) Formal 1,3-dipolar cycloaddition with nitrile oxide. (**c**) Wagner–Meerwein-type rearrangement with NBS in acetone-water.

The practical benefits of our developed methodology could be gleaned from the results of the synthetic exploration of the 1,4-cycloadducts particularly focusing on the exploitation of the isolated, unsubstituted olefinic moiety at the 11,12-positions in various types of reactions. For example, the olefin functionality at the 11,12-position of **3eD** could be effortlessly reduced by using ammonium formate as the hydrogen transfer agent in the presence of a catalytic amount of palladium on charcoal in methanol^18^ at room temperature and the corresponding hydrogenated product **11** was obtained in 98% yield (Fig. 6a). Next, a formal 1,3-dipolar cycloaddition reaction was performed on the same olefin functionality employing *N*-hydroxybenzimidoyl chloride (**12**) as the precursor for the corresponding *N*-phenyl nitrile oxide^19^. Accordingly, when **3eD** was reacted with 2.0 equivalents of **12** in the presence of excess triethylamine in toluene at 0 °C for 6 h, the corresponding [3 + 2]-cycloadduct product **13** was obtained in 74% yield as a mixture of *endo* and *exo* isomers in 10:1 ratio (Fig. 6b) along with 21% of 1-pyrrolidinyl-2,3-dicarboxylate anthracene derivative **14** - the formation of which could be explained by a retro-Diels-Alder reaction of **13** under thermal conditions. To provide evidence for this retro-Diels-Alder reaction, **13** was exposed to refluxing conditions in toluene and in 12 h we observed a complete conversion of **13** to **14** (Fig. 6b). Considering the easy functionalizability of the ester groups in **14**, this route could provide an expedient access to varied 2,3-disubstituted anthracene derivatives which might otherwise be difficult to obtain despite of being useful in a number of transformations^20,21^. Lastly, an exciting Wagner-Meerwein-type rearrangement was observed when **3eD** was treated with a superstoichiometric amount of *N*-bromosuccinamide in acetone-water (3:1) at 0–25 °C for 16 h, leading to a cycloheptanone-fused bridged naphthalene derivative **15** in 76% yield (Fig. 6c, for more details see Optimization Studies for 1,3-Dipolar Cycloaddition in the Supplementary Information). We propose that the reaction proceeds via the initial formation of a cyclic bromonium ion intermediate **16** at the 11,12-positions of **3eD** and then with the anchimeric assistance from the pyrrolidine nitrogen lone pair, a [1,2]-sigmatropic shift takes place to form the iminium intermediate **17** that upon hydrolysis furnishes the skeletally rearranged product **15** in good yield^22,23^. The structure of **15** was unambiguously determined by spectroscopic data and single-crystal X-ray analysis (see Supplementary X-Ray Crystallographic Studies and Supplementary Data 6).

### Computational studies

With a view to completely comprehending the atypical regioselectivity demonstrated by the anthracenes **1b**–**e** in above-described [4 + 2]-cycloaddition reactions with various dienophiles and aromatic substitution reactions with various electrophiles, we performed extensive *in silico* theoretical calculations.

The fundamental question arises, which governs the 9,10- vs. 1,4-selectivity in the reaction of the various dienophile and anthracene combinations. The two dienophiles **B** and **D** were picked as they show a stark contrast in their position-selectivity in the reactions with the dienes **1a**-**e**. Whereas **D** followed a smooth trend from 9,10- to 1,4-selectivity with the variation of the electronics of the dienes, **B** apparently was insensitive to this and showed expected 9,10-selectivity only.

The presented analyses go beyond the simple comparison of calculated activation barriers (ΔG^≠^) and driving forces (Δ_R_G) to achieve a fundamental understanding of the variation in product selectivities. On the same note, the concepts by Fukui (frontier molecular orbital analysis, FMO) and Bell-Evans-Polanyi (BEP) that are commonly used to predict relative reactivities, i.e., selectivities in Diels-Alder reactions, were re-evaluated in their applicability for the observed peculiar position-selectivities.

A judgment about the minimum energy pathways (MEP, concerted vs. stepwise) was based on intrinsic reaction coordinate (IRC) calculations and analysis of the root mean square (RMS) gradient. In a subsequent step, the Marcus theory was employed to differentiate the kinetic (intrinsic barrier) and thermodynamic contributions to the overall activation barrier (ΔG^≠^). To understand why the intrinsic barriers of the (first) σ-bond formation govern product ratios we then analyzed the electronics of the starting materials and transition state structures by natural bond orbital (NBO) analysis. The calculations have been run with the Gaussian09.E01 suite of programs using the B3LYP-D3/6-31 G** level of theory. For further details and citations see Supplementary Computational Studies.

The combination of dienophile DMAD (**D**) with five different anthracenes (**1a-e**, R = H, OMe, NMe_2_, bis-pyrrolidine, mono-pyrrolidine) was used to establish the choice of the level of theory (LOT). The calculated activation barriers reproduce the experimentally observed trend of 9,10- to 1,4-cycloaddition selectivity as a function of the electron donor substituent (ED) at the diene (Table 1). Therefore, this LOT was used for a more detailed analysis.

**Table 1:** Summary of calculated vs experimental selectivities of the reaction between anthracenes **1a**–**e** and DMAD (**D**).

entry	anthracene	ΔΔG^≠^_9,10–1,4_ [kcal/mol]	calculated selectivity 9,10: 1,4	experimental selectivity 9,10: 1,4
1	**1a**	−6.2	9,10 only	>99: 1
2	**1b**	−1.4	10: 1	9: 1
3	**1c**	0	1: 1	1: 2.5
4	**1d**	1.5	1: 13	<1: 99
5	**1e**	6	1,4 only	<1: 99

**Fig. 7:**
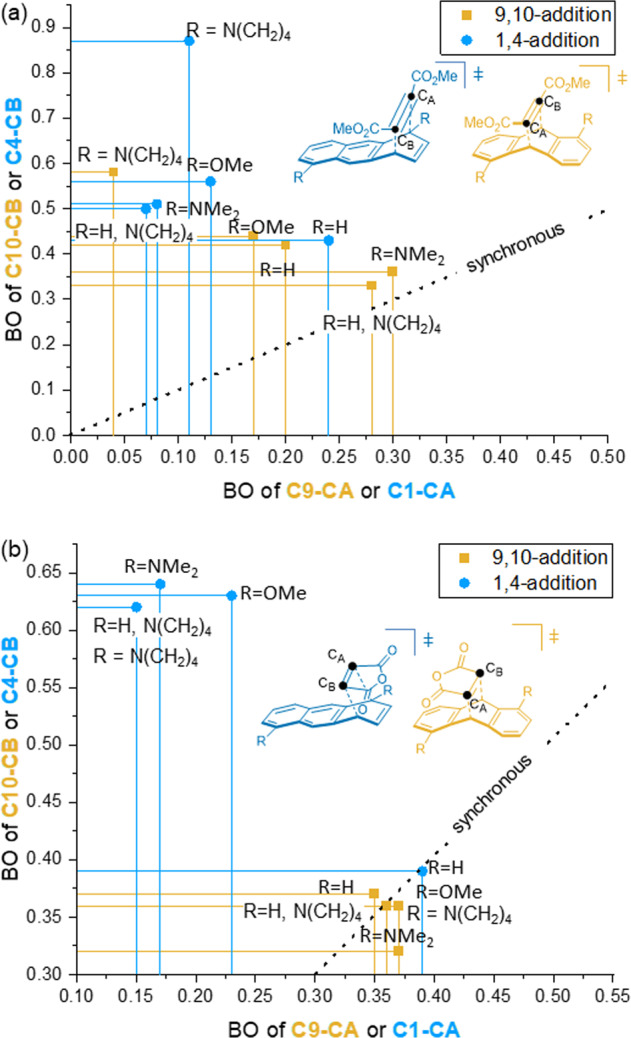
Bond order calculation. Bond orders (BOs) according to Pauling characterizing the mechanisms in the computationally analyzed Diels-Alder reactions (fully optimized structures obtained at the B3LYP-D3/6-31 G** level of theory, for further details, see Supplementary Computational Studies): a) BOs of transition state structures of reactions of **D** with **1a-e**. b) BOs of transition state structures of **B** with **1a-e**. Color coding: yellow squares C9-C10 product, blue circles C1-C4 product.

To determine the extent of concertedness, transition state (TS) structures were analyzed with respect to the extent of the bond reorganization (Fig. 7a, b) and most importantly the minimum energy pathway (MEP) of the respective transformations were scanned by IRC calculations and the RMS gradient was analyzed (see Supplementary IRC Plots).

Interestingly, the cycloaddition of **D** even with the parent anthracene (**1a**) the TS structures of the 9,10-addition, as well as the 1,4-addition, are asynchronous, with the 1,4-addition slightly more so (Fig. 7a). This is contrary to the TS structures with maleic anhydride **B** (Fig. 7b) that remained insensitive to changes in the electronics for the 9,10-additions and only showed only asynchronicity for the 1,4-addition process.

Adding electron donor groups (EDs) in 1- and/or 5-position of the anthracenes a trend towards higher asynchronicity is consistently observed for reactions with **D**. Based on the analysis of the RMS gradient along the calculated IRC a stepwise mechanism was identified to be in operation for the 1,4-additions of **D** with **1d** and **B** with **1b**-**1d**. And for the 9,10-addition in case of **D** with **1d** (see Supplementary IRC Plots for full details). With the help of this energy and force related differentiation of concerted vs. two-step mechanism in the reactions of **D,** we could define also a geometric parameter of the transition state structures as an indicator for a change in mechanism: A difference in bond orders (ΔBO^24^) of the two-forming σ-bonds of ΔBO ≥ 0.63 is characteristic for the stepwise pathway. (A definition of step-wise vs. concerted based only on bond length and corresponding bond orders of the transition state structure of interest is not trivial and not free from bias. Therefore, we opted for an approach based on the RMS gradient analysis.).

Based on the MEP-analysis the comparison of the Gibbs free activation barriers of the 9,10- vs. 1,4-addition were conducted with respect to either the concerted pathways or for the first σ-bond formation step that is occurring.

In case of the addition reaction with D the 1,4 addition is consistently facilitated by increasingly electron-donating substituents, whereas the 9,10-addition barrier is significantly less sensitive to changes in the diene electronics (see Supplementary Tables 2–4) and in addition, shows no consistent correlation.

**Fig. 8:**
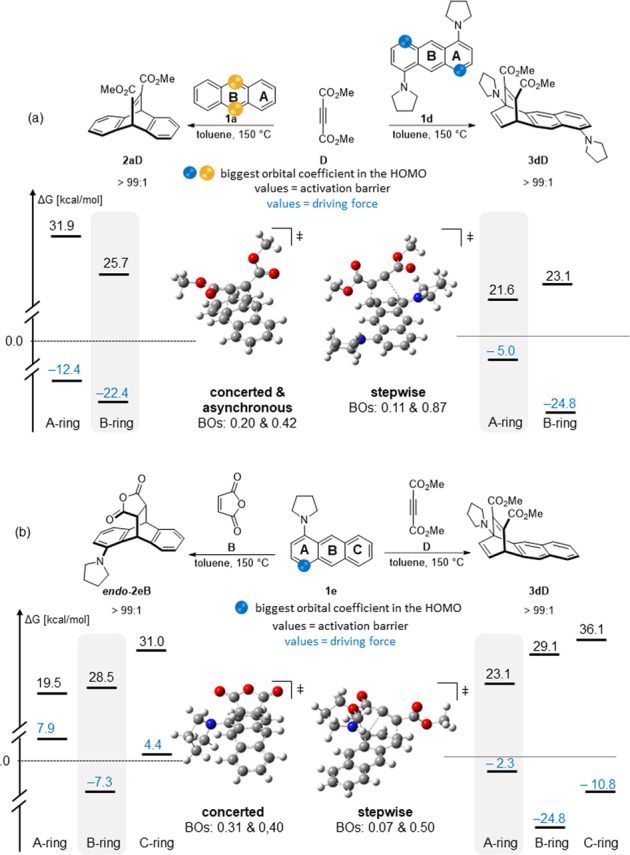
Summary of the experimental results and their computational rational based on FMO-analysis, activation barriers, mechanism (concerted vs. stepwise), and thermodynamics. (A) Variation of the anthracene (exemplified for **1a** and **1d**) in presence of **D** leads to changes in the regioselectivity that are driven by kinetic parameters. (B) Based on the FMO analysis this regioselectivity is intrinsic to the anthracene (biggest orbital coefficients). On the contrary, changing the dieneophile from **D** to **B** this intrinsic and kinetic preference of the 1,4-product is overwritten by its unfavorable thermodynamics. The computational data was obtained at the B3LYP-D3/6-31 G** level of theory. Energies were calculated using the harmonic oscillator approach and include zero-point energy corrections (for further details see Supplementary Computational Studies). Color coding: yellow circles indication of biggest orbital coefficients for 9,10 addition, blue circles for 1,4 addition; blue numbers: Δ_R_G-values (driving forces), black numbers: ΔG^≠^ (activation barriers).

The relative thermodynamic contributions (Δ_R_G) to the activation barrier of the reaction with **D** based on the Marcus analysis^25^ do not explain the observed product selectivities. On the contrary, the 1,4-addition product is thermodynamically significantly less stable than the 9,10-addition product and moreover, the driving force for the 1,4-product formation decreases with increasing strength of the electron donor (ED; see Fig. 8 and Supplementary Tables 2–4). Clearly, there is no linear free energy relationship in operation. In addition, analysis of intrinsic activation barriers ΔG^≠°^ according to Marcus confirmed this conclusion: the observed product selectivities are kinetic in nature (see Supplementary Tables 11–13).

Kinetic control of the product formation may be rationalized by a maximal orbital overlap in the (rate-determining) step. Therefore, we focused our analysis on the understanding of how the amino substituents change the electronic (FMO) and nuclear structure of the transition states (TSs) and the dienes in favor of the 1,4-addition process. This analysis is based on the frontier molecular orbitals (FMOs), and the natural bond orbital (NBO) analysis (partial charges).

Although the 1,4-addition pathway is shifting from a concerted to a step-wise bond reorganization in the series **1a** to **1e** the FMO analysis of the anthracenes turned out to correctly predict the position selectivity in the reactions with **D**. The relative size of the orbital coefficients in the anthracenes provided a mean for a quick estimate where the first bonding interaction will take place (Fig. 8 and Supplementary FMO Analysis). On the other hand, the HOMO-LUMO gap Δε of the reactants reflects the increased ease of the 1,4-addition in the series R = H to N(CH_2_)_4,_ surprisingly well (see Supplementary Tables 14 and 15). Whereas, on the other hand, there is no correlation between the activation barriers of the 9,10-addition and Δε. This observation can be read in support of the results obtained by the Marcus-analysis, i.e., only the 1,4-addition is a kinetically preferred process in the reactions of **D**.

Since the bond reorganization is approaching the stepwise limit in the reaction of **D** and **1d**, **e** charge separation and its stabilization by the substituents is deemed to be the second important player in the kinetically controlled Diels-Alder reaction in anthracene derivatives **1b-e**. With an increasing asymmetry, one would expect a built-up of partial positive charge at C1 and partial negative charge at CA (see Fig. 7 for nomenclature). Indeed, a significant charge separation in the TS structures was confirmed by NBO analysis (see Supplementary Tables 16–18).

The comparison of the aforementioned parameters also provided a clue for the apparent insensitivity of maleic anhydride (**B**) to the electronic changes in **1a**-**e**.

The computed activation barriers and driving forces indicated that in the reaction of **C** with all five analyzed dienes, the 1,4-addition is still kinetically favored, but the transformation is thermodynamically inhibited (endergonic; Fig. 8B). Hence, the 9,10-adduct is predicted to be the only product being formed in the case of **B** independent of the electronic situation in the diene (Fig. 8, for more details, see Supplementary Computational Studies).

## Conclusion

To conclude we have addressed a longstanding problem in controlling and accessing atypical regioselectivity in Diels-Alder reaction of anthracenes by a simple yet highly effective electronic tweaking of the terminal rings by installing substituents of varying electron-donating abilities at the 1- or 1,5-positions. Consequently, a functionally diverse range of 1,4-cycloadducts could be obtained in up to excellent yields and regioselectivity. Synthetic values of such products have also been demonstrated with diverse functionalization of a 1,4-cycloadduct. Computational studies revealed the origin of the atypical regioselectivity to be of kinetic nature, being reflected by the largest orbital coefficient in the HOMO at the 1,4-positions imposed by the electron-donating ability of substituents in the terminal rings and the highly asynchronous transition states leading to the products.

## Methods

### General information

For more details, see Supplementary Methods.

### X-ray crystallographic structures of compounds 3bD, 3cD, 3dD, 3eD, and 15

For the CIF files, see Supplementary Data 2–6, respectively. For more details, see Supplementary X-Ray Crystallographic Studies.

### Synthesis and characterization

See Supplementary Methods (general information about chemicals and analytical methods, and synthetic procedures), Spectral Data of Products (^1^H and ^13^C NMR data, and HRMS data), Supplementary Figs. 1–89 (^1^H and ^13^C NMR spectra).

### Detailed results of Diels-Alder reactions of anthracenes and alkenes

For more details, see Supplementary Table 1.

### Computational studies

See Supplementary Computational Studies (Supplementary Tables 2–18), Calculated Thermodynamic Data (Supplementary Tables 2–4), Calculation of Bond Orders (Supplementary Tables 5–10), Marcus analysis (Supplementary Tables 11–13), FMO Analysis (Supplementary Tables 14+15), NBO Analysis (Partial Charges; Supplementary Tables 16–18), All obtained IRC plots (Supplementary Figs. 92–107) and all calculated structures in Supplementary Data 1.

### General procedure for [4 + 2]-cycloaddition reactions

In a sealed pressure tube anthracene substrate (1.0 equiv) and dienophile (1.1 equiv) were dissolved in toluene (0.5 M) and stirred at 150 °C until complete conversion of starting material was observed (monitored by TLC). The solvent was removed under reduced pressure and the crude product was purified by flash column chromatography.

## Supplementary information

Supporting Information

Description of Additional Supplementary Files

Supplementary Data 1

Supplementary Data 2

Supplementary Data 3

Supplementary Data 4

Supplementary Data 5

Supplementary Data 6

## Data Availability

The X-ray crystallographic coordinates for structures reported in this Article have been deposited at the Cambridge Crystallographic Data Centre (CCDC), under deposition numbers CCDC 1979830 (**3bD**), CCDC 1979832 (**3cD**), 1979833 (**3dD**), 1979834 (**3eD**), and 2017368 (**15**). These data can be obtained free of charge from The Cambridge Crystallographic Data Centre via www.ccdc.cam.ac.uk/data_request/cif. The data supporting the findings of this study are available within the paper and its Supplementary Information (Supplementary Data 2–6 – crystallographic information files for **3bD**, **3cD**, **3dD**, **3eD**, and **15**). All other relevant data are available from the authors.
