# Supplementary material for: Diels–Alder reactions and electrophilic substitutions with atypical regioselectivity enable functionalization of terminal rings of anthracene
Source: Commun Chem. 2020 Nov 6;3:158. doi: 10.1038/s42004-020-00407-9 (PMC9814106; doi:10.1038/s42004-020-00407-9)
Supplement: Supplementary file 1 — Supporting Information [file 42004_2020_407_MOESM1_ESM.pdf]

Supplementary Materials

for

Diels-Alder Reactions and Electrophilic Substitutions with Atypical Regioselectivity Enable Functionalization of Terminal Rings of Anthracene

Vinh Ngoc Huynh,[a,b] Michael Leitner,[a] Aditya Bhattacharyya,[a] Lisa Uhlstein,[a] Peter Kreitmeier,[a] Patrick Sakrausky,[a] Julia Rehbein,[a,*] Oliver Reiser[a,*]

[a]*Institut für Organische Chemie, Universität Regensburg, Universitätsstr. 31, 93053 Regensburg, Germany; email: Oliver.Reiser@chemie.uni-regensburg.de; Julia.Rehbein@chemie.uni-regensburg.de*

[b]*University of Science, Vietnam National University, 227 Nguyễn Văn Cừ street, district 5, Ho Chi Minh City, Vietnam*

Table of Contents

Sl. No.	Contents	Page No.
1.	Supplementary Methods……………………………………………..	2
2.	Supplementary Table 1…..……………………………………………	6
3.	Spectral Data of Products……………………………………………..	7
4.	Optimization Studies for 1,3-Dipolar Cycloaddition………………….	20
5.	NMR spectra…………………………………………………………..	23
6.	X-Ray Crystallographic Studies………………………………………	70
7.	Computational Studies………………………………………………..	75
8.	Calculated Thermodynamic Data (Supplementary Tables 2–4)………	76
9.	Calculation of Bond Orders (Supplementary Tables 5–10)…………...	77
10.	Marcus analysis (Supplementary Tables S11–S13)…………………...	80
11.	FMO Analysis (Supplementary Tables S14 + S15)………………..….	81
12.	NBO Analysis (Partial Charges; Supplementary Tables 16–18)….….	82
13.	All obtained IRC Plots……………………………………………...	84
14.	Supplementary References…………….………...…………………….	93

1. Supplementary Methods

1.1. General Information

All moisture sensitive reactions were performed in flame-dried glassware under nitrogen atmosphere. All reactions were performed in pressure tube apparatus. Commercially available chemicals were purchased in high quality and used without any further purification. DCM, ethyl acetate and hexanes (petroleum ether, PE (60/40)) were distilled prior to use. Anhydrous solvents were prepared according to standard procedures. All the reactions were monitored by TLC and visualized by a dual short (254 nm) / long (366 nm) wavelength UV lamp and staining was performed with vanillin or potassium permanganate solutions followed by heating. Analytical thin layer chromatography was performed on Merck TLC aluminium sheets silica gel 60 F 254. Column chromatographic purifications were performed using Merck flash silica gel 60 (0.040–0.063 mm). ^{1}H- and ^{13}C-NMR spectra were recorded on BRUKER Avance III 400 Nanobay (400 MHz) and BRUKER Avance 300 (300 MHz) spectrometers at ambient temperature. Chemical shifts are reported as δ (ppm) relative to the signal of the solvent. Characterization of the signals: s = singlet, d = doublet, t = triplet, quin = quintet, m = multiplet, br s= broad singlet, dd = doublet of a doublet, ddd = doublet of doublet of doublet, dt = doublet of a triplet, dq = doublet of a quartet, td = triplet of a doublet, qt = quartet of a triplet. Integration is determined as the relative number of atoms, and the coupling constants (*J*) are given in Hertz (Hz). FT-IR spectroscopy was carried out on a Cary 630 spectrometer, equipped with a Diamond Single Reflection ATR-System and compounds were measured neatly, and wave numbers are reported in cm^{-1}. Mass spectrometry was performed in the Central Analytical Department of the University of Regensburg on a Jeol AccuTOF GCX, Agilent Q-TOF 6540 UHD, Finnigan MAT SSQ 710 A or a ThermoQuest Finnigan TSQ 7000 and are reported in *m/z*. The melting points were measured on an SRS MPA 100 OptiMelt in a silicon oil bath and are reported uncorrected. Single crystal X-ray analyses were performed on Agilent Technologies SuperNova, Agilent Technologies Gemini R Ultra or Stoe IPDS I.

1.2. Synthetic Procedures and Spectral Data of Starting Materials

1,5-Dimethoxy-9,10-anthraquinone

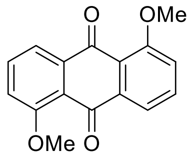

To a stirred solution of 1,5-dihydroxy-9,10-anthraquinone (9.61 g, 40.0 mmol, 1.0 equiv) and K_2CO_3 (15.8 g, 114 mmol, 2.9 equiv) in acetone (50.0 mL) dimethyl sulfate (8.27 mL, 87.2 mmol, 2.2 equiv) was added. The reaction mixture was refluxed for 48 h. After being cooled to room temperature, the solvent was removed under reduced pressure. The crude product was purified by flash chromatography (DCM) and 1,5-dimethoxy-9,10-anthraquinone was obtained as yellow needles in 79% yield (8.43 g, 31.4 mmol). The spectroscopic data is in accordance with the literature[1].

^{1}H NMR (300 MHz, $CDCl_3$) δ 8.01–7.78 (m, 2H), 7.77–7.51 (m, 2H), 7.38–7.09 (m, 2H), 4.13–3.82 (m, 6H); **^{13}C NMR** (75 MHz, $CDCl_3$) δ 182.8, 159.8, 137.5, 135.1, 120.9, 119.8, 116.8, 56.6.

1,5-Bis(dimethoxy)anthracene (1b)

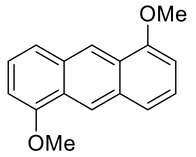

1b

This compound **1b** was synthesized from 1,5-dimethoxy-9,10-anthraquinone following the literature procedure[2] in 64% yield (1.48 g, 6.21 mmol). The spectroscopic data is in accordance with the literature.

^{1}H NMR (300 MHz, $CDCl_3$) δ 8.79 (s, 2H), 7.64 (d, J = 8.8 Hz, 2H), 7.36 (dd, J = 8.5, 7.4 Hz, 2H), 6.75 (d, J = 7.3 Hz, 2H), 4.08 (s, 6H); **^{13}C NMR** (75 MHz, $CDCl_3$) δ 155.5, 132.3, 125.3, 121.3, 120.6, 102.1, 55.7.

1,5-Diaminoanthracene

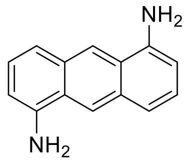

1,5-Diaminoanthracene was synthesized from 1,5-diamino-9,10-anthraquinone following the literature procedure[3] in 78% yield (4.50 g, 21.6 mmol). The spectroscopic data is in accordance with the literature.

^{1}H NMR (300 MHz, DMSO-d_6) δ 8.57 (s, 2H), 7.40–6.97 (m, 4H), 6.61 (d, J = 6.9 Hz, 2H), 5.83 (s, 4H); **^{13}C NMR** (75 MHz, DMSO-d_6) δ 144.2, 131.3, 125.8, 123.0, 120.5, 116.0, 104.7.

1,5-Bis(*N,N*-dimethylamino)anthracene (1c)

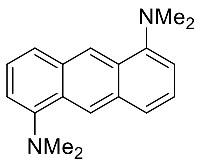

1c

1,5-Diaminoanthracene (4.00 g, 19.2 mmol, 1.0 equiv) and methyl iodide (12.0 mL, 192 mmol, 10 equiv) were dissolved in dry THF (200 mL) under a nitrogen atmosphere. The mixture was cooled to 0 °C and a 60% dispersion of NaH in mineral oil (7.68 g, 192 mmol, 10 equiv) was added portionwise. The reaction mixture was stirred at room temperature for 12 h and then refluxed for additional 12 h. The mixture was cooled with an ice/water bath and water was slowly added to it. After the removal of THF under reduced pressure, the resulting mixture was extracted with DCM (3 × 40 mL). The combined extracts were washed with brine, dried over anhydrous $MgSO_4$ and the solvent was removed under reduced pressure. The black crude reaction mixture was purified by flash column chromatography (hexanes:DCM = 3:1) to afford the pure product **1c** as bright yellow solid in 64% yield (3.20 g, 12.1 mmol).

Mp.: 144–145 °C; **^{1}H NMR** (300 MHz, $CDCl_3$) δ 8.78 (d, J = 3.5 Hz, 2H), 7.75 (d, J = 8.5 Hz, 2H), 7.39 (dd, J = 10.4, 5.2 Hz, 2H), 7.02 (d, J = 7.2 Hz, 2H), 3.01 (s, 12H); **^{13}C NMR** (75 MHz, $CDCl_3$) δ 150.6, 132.8, 127.5, 125.2, 123.6, 112.6, 45.3; **IR** (ν/cm^{-1}): 3049, 2981, 2944, 2862, 2832, 2788, 1610, 1543, 1453, 1349, 1189, 1140, 1077, 916, 793, 730; **HRMS** (ESI-TOF) *m/z* calculated for $C_{18}H_{21}N_2$ [MH^+]: 265.1699 found: 265.1702.

1,5-Bis(dipyrrolidin-1-yl)anthracene (1d)

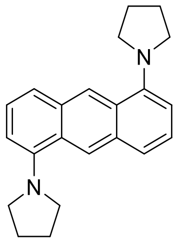

1d

To a suspension of 1,5-diaminoanthracene (2.00 g, 9.60 mmol, 1.0 equiv) and K_2CO_3 (5.31 g, 38.4 mmol, 4.0 equiv) in dioxane/water (1:1 v/v, 40.0 mL) 1,4-dibromobutane (3.49 mL, 28.8 mmol, 3.0 equiv) was added at room temperature. After heating the mixture under refluxing condition for 24 h, additional 1,4-dibromobutane (3.49 mL, 28.8 mmol, 3.0 equiv) was added. The mixture was refluxed again for 24 h before it was allowed to cool down to room temperature. Cold water was added and the aqueous phase was extracted with DCM (3 × 25 mL). The combined organic phases were dried over anhydrous $MgSO_4$ and the solvent was removed under reduced pressure to give rise to a black crude reaction mixture which was purified by flash column chromatography (hexanes:DCM = 7:3 to 1:1) affording the pure product **1d** as bright yellow solid in 51% yield. (1.55 g, 4.90 mmol).

Mp.: 194–195 °C; **^{1}H NMR** (300 MHz, $CDCl_3$) δ 8.74 (s, 2H), 7.65 (d, J = 8.4 Hz, 2H), 7.45–7.28 (m, 2H), 6.91 (d, J = 7.3 Hz, 2H), 3.50 (t, J = 6.4 Hz, 8H), 2.27–1.91 (m, 8H); **^{13}C NMR** (75 MHz, $CDCl_3$) δ 147.3, 132.4, 127.1, 125.1, 123.9, 122.1, 109.7, 52.8, 25.0;

IR (ν/cm^{-1}): 3067, 2948, 2873, 2821, 1610, 1539, 1453, 1353, 1110, 920, 793, 730; **HRMS** (ESI-TOF) *m/z* calculated for $C_{22}H_{25}N_2$ [MH$^+$]: 317.2012, found: 317.2013.

1-Aminoanthracene

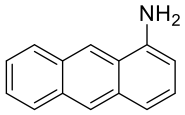

1-Aminoanthracene was synthesized from 1-amino-9,10-anthraquinone following the literature procedure[4] in 69% yield (11.3 g, 58.5 mmol). The spectroscopic data is in accordance with the literature.

^{1}H NMR (300 MHz, $CDCl_3$) δ 8.40 (s, 2H), 8.06 – 7.95 (m, 2H), 7.52 (dd, J = 8.5, 1.0 Hz, 1H), 7.50 – 7.43 (m, 2H), 7.31 (dd, J = 8.5, 7.1 Hz, 1H), 6.76 (dd, J = 7.1, 1.0 Hz, 1H), 4.29 (s, 2H); **^{13}C NMR** (75 MHz, $CDCl_3$) δ 141.9, 132.6, 131.7, 131.0, 128.5, 128.0, 126.7, 125.9, 125.6, 125.2, 123.9, 119.7, 119.4, 107.7.

1-(Pyrrolidine-1-yl)anthracene (1e)

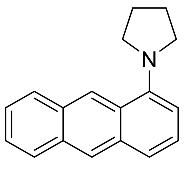

1e

To a suspension of 1-aminoanthracene (9.79 g, 50.7 mmol, 1.0 equiv) and K_2CO_3 (14.0 g, 101.3 mmol, 2.0 equiv) in dioxane/water (1:1 v/v, 212 mL) 1,4-dibromobutane (8.97 mL, 76.0mmol, 1.5 equiv) was added at room temperature. After heating the mixture under reflux condition for 24 h, additional 1,4-dibromobutane (8.97 mL, 76.0 mmol, 1.5 equiv) was added. The mixture was refluxed again for 24 h before it was allowed to cool down to room temperature. Cold water was added and the aqueous phase was extracted with DCM (3 × 40 mL). The combined organic phases were dried over anhydrous $MgSO_4$ and the solvent was removed under reduced pressure to give a black crude reaction mixture which was purified by flash column chromatography (hexanes:DCM = 7:3 to 1:1) to afford the pure product **1e** as bright yellow solid in 57% yield (7.14 g, 28.9 mmol).

Mp.: 60–61 °C; **^{1}H NMR** (400 MHz, $CDCl_3$) δ 8.85 (s, 1H), 8.41 (s, 1H), 8.13–7.96 (m, 2H), 7.66 (d, J = 8.4 Hz, 1H), 7.54–7.45 (m, 2H), 7.39 (dd, J = 8.4, 7.3 Hz, 1H), 6.95 (d, J = 7.3 Hz, 1H), 3.56–3.44 (m, 4H), 2.17–2.06 (m, 4H); **^{13}C NMR** (101 MHz, $CDCl_3$) δ 147.5, 133.3, 131.4, 130.8, 128.8, 127.7, 126.3, 125.5, 125.0, 123.7, 121.7, 109.9, 52.9, 24.9; **IR** (ν/cm^{-1}): 3049, 2996, 2967, 2870, 2810, 1613, 1535, 1453, 1394, 1349, 1230, 1129, 887, 749; **HRMS** (EI-TOF) *m/z* calculated for $C_{18}H_{17}N$ [M$^+$]: 247.1355, found: 247.1350.

2. Supplementary Table 1. Diels-Alder reactions between substituted anthracenes **1b-1e** and dienophiles **A-C**.

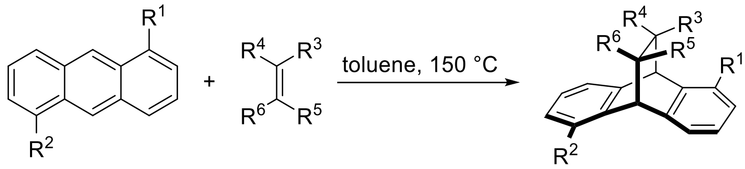

1b = R^1, R^2 = OMe
1c = R^1, R^2 = NMe_2
1d = R^1, R^2 = $N(CH_2)_4$
1e = R^1 = H, R^2 = $N(CH_2)_4$

A-C **2A-C**

entry[a]	anthracene	dienophile	product[b]	
**1**	**1b**	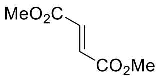 **A**	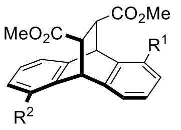	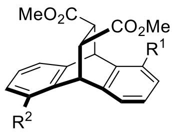
**2**	**1c**		***syn*-2bA: R^1, R^2 = OMe = 95%**	
**3**	**1d**		***syn*-2cA: R^1, R^2 = NMe_2 = 40%**	***anti*-2bA: R^1, R^2 = NMe_2 = 28%**
**4**	**1e**		***syn*-2dA: R^1, R^2 = $N(CH_2)_4$ = 55%**	***anti*-2cA: R^1, R^2 = $N(CH_2)_4$ = 31%**
			***syn*-2eA: R^1 = $N(CH_2)_4$, R^2 = H = 46%**	***anti*-2eA: R^1 = $N(CH_2)_4$, R^2 = H = 50%**
**3**	**1b**	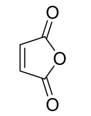 **B**	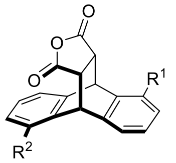	
**4**	**1c**		**2bB: R^1, R^2 = OMe = 97%**	
**5**	**1d**		**2cB: R^1, R^2 = NMe_2 = 88%**	
**6**	**1e**		**2dB: R^1, R^2 = $N(CH_2)_4$ = 75%**	
			**2eB: R^1 = H, R^2 = $N(CH_2)_4$ = 99%[c, d]**	
**5**	**1b**	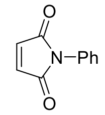 **C**	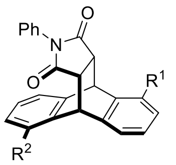	
**6**	**1c**		**2bC: R^1, R^2 = OMe = 99%**	
**7**	**1d**		**2cC: R^1, R^2 = NMe_2 = 98%**	
**8**	**1e**		**2dC: R^1, R^2 = $N(CH_2)_4$ = 92%**	
			**2eC: R^1 = H, R^2 = $N(CH_2)_4$ = 95%[d]**	

[a] anthracene (**1b,c**, 1.0 equiv) and dienophile (**A-C**, 1.1 equiv) in toluene (0.5 M) in a sealed tube at 150 °C (for details see SI), [b] Isolated yield, [c] determined by ^{1}H-NMR, [d] also *exo*-product with R^1 = $N(CH_2)_4$, R^2 = H obtained (ratio *endo*/*exo* = 67:33).

3. Spectral Data of Products

Dimethyl 9,10-dihydro-9,10-ethenoanthracene-11,12-dicarboxylate (2aD)

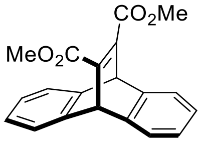

2aD

Following the general procedure, reaction of anthracene (**1a**) (200 mg, 1.12 mmol, 1.0 equiv) and DMAD (**D**) (175 mg, 1.23 mmol, 1.1 equiv) afforded after 24 h **2aD** as white solid in 87% yield (314 mg, 0.98 mmol) after purification by flash column chromatography (hexanes:DCM = 1:1). The spectroscopic data is in accordance with the literature[5].

^{1}H NMR (300 MHz, $CDCl_3$) δ 7.48–7.32 (m, 4H), 7.10–6.94 (m, 4H), 5.48 (s, 2H), 3.79 (s, 6H).

Syn-dimethyl-1,5-dimethoxy-9,10-dihydro-9,10-ethanoanthracene-11,12-dicarboxylate (*syn*-2bA)

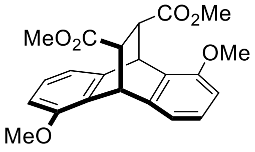

***syn*-2bA**

Following the general procedure, reaction of 1,5-bis(dimethoxy)anthracene (**1b**) (80.0 mg, 0.34 mmol, 1.0 equiv) and dimethyl fumarate (**A**) (53.0 mg, 0.37 mmol, 1.1 equiv) afforded after 24 h *syn*-**2bA** as white solid in 95% yield (122 mg, 0.32 mmol) after purification by flash column chromatography (DCM to DCM:EA = 99:1).

***syn*-2bA**: **Mp.**: 162–163 °C; **^{1}H NMR** (400 MHz, $CDCl_3$) δ 7.10–6.99 (m, 3H), 6.91 (d, J = 7.3 Hz, 1H), 6.68 (d, J = 8.2 Hz, 2H), 5.22–5.17 (m, 2H), 3.85 (s, 3H), 3.82 (s, 3H), 3.64 (s, 3H), 3.63 (s, 3H), 3.37 (dt, J = 5.5, 1.3 Hz, 2H); **^{13}C NMR** (101 MHz, $CDCl_3$) δ 173.18, 173.15, 155.1, 154.0, 144.2, 142.5, 129.9, 128.2, 127.2, 127.0, 117.5, 116.6, 109.1, 108.9, 55.7, 55.5, 52.2, 52.1, 47.5, 47.4, 40.0, 39.5; **IR** (ν/cm^{-1}): 3000, 2952, 2840, 1729, 1587, 1483, 1438, 1263, 1200, 1095, 1021, 909, 782, 730; **HRMS** (ESI-TOF) *m/z* calculated for $C_{22}H_{22}O_6$ [MH^+]: 383.1489, found: 383.1490.

1,5-Dimethoxy-9,10-dihydro-9,10-[3,4]furanoanthracene-12,14-dione (2bB)

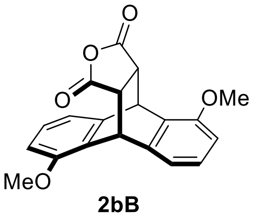

2bB

Following the general procedure, reaction of 1,5-bis(dimethoxy)anthracene (**1b**) (80.0 mg, 0.34 mmol, 1.0 equiv) and maleic anhydride (**B**) (36.0 mg, 0.37 mmol, 1.1 equiv) afforded after 2 h **2bB** as white solid in 97% yield (109 mg, 0.32 mmol) after purification by recrystallization from DCM.

Mp.: 263–264 °C; **^{1}H NMR** (400 MHz, $CDCl_3$) δ 7.14 (ddd, J = 8.3, 7.4, 4.2 Hz, 2H), 7.03 (d, J = 7.4 Hz, 1H), 6.98 (d, J = 7.4 Hz, 1H), 6.75 (ddd, J = 8.4, 5.7, 0.9 Hz, 2H), 5.33 (dd, J = 17.1, 3.0 Hz, 2H), 3.86 (s, 3H), 3.84 (s, 3H), 3.50–3.41 (m, 2H).; **^{13}C NMR** (101 MHz, $CDCl_3$) δ 170.7, 170.6, 155.0, 154.6, 142.8, 140.2, 128.6, 128.5, 127.8, 126.4, 117.9, 117.0, 109.5, 55.9, 55.6, 47.8, 47.7, 38.5, 38.4; **IR** (ν/cm^{-1}): 3011, 2959, 2922, 2840, 1863, 1781, 1587, 1483, 1267, 1215, 1099, 939, 786, 726; **HRMS** (EI-TOF) *m/z* calculated for $C_{20}H_{16}O_5$ [M$^+$]: 336.0992, found: 336.0998.

1,5-Dimethoxy-13-phenyl-9,10-dihydro-9,10-[3,4]epipyrroloanthracene-12,14-dione (2bC)

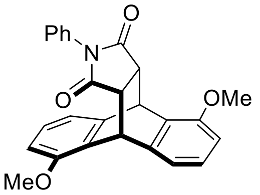

2bC

Following the general procedure, reaction of 1,5-bis(dimethoxy)anthracene (**1b**) (80.0 mg, 0.34 mmol, 1.0 equiv) and *N*-phenylmaleimide (**C**) (64.0 mg, 0.37 mmol, 1.1 equiv) afforded **2bC** in 2 h as white solid in 99% yield (137 mg, 0.33 mmol) after purification by flash column chromatography (DCM:EA = 99:1).

Mp.:274–275 °C; **^{1}H NMR** (400 MHz, $CDCl_3$) δ 7.35–7.27 (m, 3H), 7.16 (ddd, J = 8.1, 7.3, 4.6 Hz, 2H), 7.08 (d, J = 7.3 Hz, 1H), 7.01 (d, J = 7.3 Hz, 1H), 6.77 (ddd, J = 8.2, 5.8, 0.9 Hz, 2H), 6.60–6.52 (m, 2H), 5.42 (d, J = 3.3 Hz, 1H), 5.38 (d, J = 3.3 Hz, 1H), 3.88 (s, 3H), 3.81 (s, 3H), 3.32 (dq, J = 8.4, 3.3 Hz, 2H); **^{13}C NMR** (101 MHz, $CDCl_3$) δ 176.3, 176.1, 155.1, 154.6, 143.5, 140.8, 131.7, 129.3, 129.1, 128.7, 127.8, 127.5, 127.1, 126.6, 118.0, 117.0, 109.8, 109.2, 56.1, 55.6, 46.9, 46.7, 38.8; **IR** (ν/cm^{-1}): 3067, 2996, 2937, 2899, 2836, 2255, 1777, 1707, 1584, 1479, 1382, 1263, 1095, 909, 723; **HRMS** (ESI-TOF) *m/z* calculated for $C_{26}H_{21}NO_4$ [MH$^+$]: 412.1543, found: 412.1547.

Dimethyl 1,5-dimethoxy-9,10-dihydro-9,10-ethenoanthracene-11,12-dicarboxylate (2bD)

Dimethyl 1,5-dimethoxy-1,4-dihydro-1,4-ethenoanthracene-2,3-dicarboxylate (3bD)

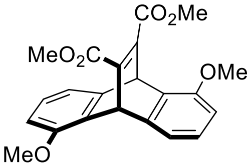

2bD

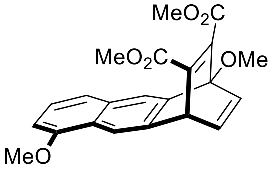

3bD

Following the general procedure, reaction of 1,5-bis(dimethoxy)anthracene (**1b**) (300 mg, 1.26 mmol, 1.0 equiv) and DMAD (**D**) (197 mg, 1.38 mmol, 1.1 equiv) afforded after 48 h **2bD** as white solid in 73% yield (351 mg, 0.92 mmol) and **3bD** as white solid in 6% yield (29.0 mg, 0.08 mmol) after purification by flash column chromatography (hexanes:DCM = 1:1).

2bD: **Mp.**: 238–239 °C; **^{1}H NMR** (300 MHz, $CDCl_3$) δ 7.07 (d, J = 7.2 Hz, 2H), 7.02–6.91 (m, 2H), 6.60 (d, J = 8.0 Hz, 2H), 5.93 (s, 2H), 3.83 (s, 6H), 3.79 (s, 6H); **^{13}C NMR** (75 MHz, $CDCl_3$) δ 166.1, 154.6, 147.8, 146.3, 131.9, 126.3, 116.8, 108.7, 55.8, 52.4, 45.9; **IR** (ν/cm^{-1}):

2996, 2955, 2836, 1714, 1632, 1587, 1479, 1435, 1334, 1259, 1211, 1088, 775, 741, 670; **HRMS** (ESI-TOF) *m/z* calculated for $C_{22}H_{21}O_6$ [MH$^+$]: 381.1333, found: 381.1337.

3bD: **Mp.**: 143–144 °C; **^{1}H NMR** (300 MHz, $CDCl_3$) δ 8.09 (s, 1H), 7.81 (s, 1H), 7.45–7.29 (m, 2H), 7.16 (dd, J = 7.5, 1.6 Hz, 1H), 7.02 (dd, J = 7.4, 6.1 Hz, 1H), 6.81 (dd, J = 5.9, 2.8 Hz, 1H), 5.44 (dd, J = 6.1, 1.4 Hz, 1H), 3.97 (s, 3H), 3.92 (s, 3H), 3.83 (s, 3H), 3.73 (s, 3H); **^{13}C NMR** (75 MHz, $CDCl_3$) δ 166.5, 163.3, 155.2, 141.7, 139.0, 138.2, 135.6, 132.0, 126.1, 122.8, 120.5, 119.1, 115.6, 104.7, 89.3, 55.6, 52.5, 46.9; **IR** (ν/cm^{-1}): 3075, 3008, 2952, 2840, 1736, 1640, 1595, 1431, 1259, 1103, 902, 793, 708; **HRMS** (ESI-TOF) *m/z* calculated for $C_{22}H_{21}O_6$ [MH$^+$]: 381.1333, found: 381.1338.

***Syn*-dimethyl 1,5-bis(dimethylamino)-9,10-dihydro-9,10-ethanoanthracene-11,12-dicarboxylate (*syn*-2cA)**

***Anti*-dimethyl 1,5-bis(dimethylamino)-9,10-dihydro-9,10-ethanoanthracene-11,12-dicarboxylate (*anti*-2cA)**

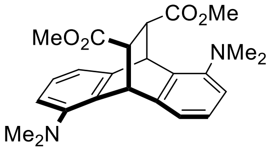

***syn*-2cA**

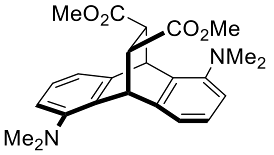

***anti*-2cA**

Following the general procedure, reaction of 1,5-bis(*N,N*-dimethylamino)anthracene (**1c**) (200 mg, 0.76 mmol, 1.0 equiv) and dimethyl fumarate (**A**) (118 mg, 0.83 mmol, 1.1 equiv) afforded after 24 h *syn*-**2cA** as white solid in 40% yield (125 mg, 0.31 mmol) and *anti*-**2cA** as white solid in 28% yield (87.0 mg, 0.21 mmol) after purification by flash column chromatography (hexanes:EA = 4:1).

***syn*-2cA**: **Mp.**: 159–160 °C; **^{1}H NMR** (300 MHz, $CDCl_3$) δ 7.19–6.96 (m, 4H), 6.96–6.68 (m, 2H), 5.21 (s, 2H), 3.60 (s, 6H), 3.38 (s, 2H), 2.73 (s, 12H); **^{13}C NMR** (75 MHz, $CDCl_3$) δ 173.1, 150.4, 143.0, 134.7, 126.8, 118.5, 116.5, 52.0, 47.5, 45.1, 42.6; **IR** (ν/cm^{-1}): 2981, 2940, 2858, 2825, 2776, 1729, 1584, 1479, 1435, 1304, 1174, 1010, 883, 793, 745; **HRMS** (ESI-TOF) *m/z* calculated for $C_{24}H_{29}N_2O_4$ [MH$^+$]: 409.2122, found: 409.2125.

***anti*-2cA**: **Mp.**: 139–140 °C; **^{1}H NMR** (300 MHz, $CDCl_3$) δ 7.15–6.96 (m, 4H), 6.87 (d, J = 7.6 Hz, 2H), 5.18 (s, 2H), 3.67 (s, 6H), 3.39 (s, 2H), 2.79 (s, 12H); **^{13}C NMR** (75 MHz, $CDCl_3$) δ 173.2, 149.2, 141.5, 135.9, 126.5, 119.4, 116.1, 52.2, 47.5, 45.0, 42.1; **IR** (ν/cm^{-1}): 3056, 2985, 2948, 2825, 2780, 1729, 1584, 1479, 1435, 1375, 1259, 1192, 1039, 1010, 920, 864, 749; **HRMS** (ESI-TOF) *m/z* calculated for $C_{24}H_{29}N_2O_4$ [MH$^+$]: 409.2122, found: 409.2127.

1,5-Bis(dimethylamino)-9,10-dihydro-9,10-[3,4]furanoanthracene-12,14-dione (2cB)

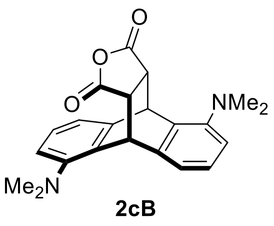

2cB

Following the general procedure, reaction of 1,5-bis(*N,N*-dimethylamino)anthracene (**1c**) (100 mg, 0.38 mmol, 1.0 equiv) and maleic anhydride (**B**) (41.0 mg, 0.42 mmol, 1.1 equiv)

afforded after 0.5 h **2cB** as white solid in 88% yield (120 mg, 0.33 mmol) after purification by recrystallization from DCM.

Mp.: 265–266 °C; **^{1}H NMR** (300 MHz, $CDCl_3$) δ 7.21–7.00 (m, 4H), 7.00–6.84 (m, 2H), 5.37 (d, J = 3.0 Hz, 1H), 5.25 (d, J = 2.8 Hz, 1H), 3.57–3.30 (m, 2H), 2.77 (s, 6H), 2.74 (s, 6H); **^{13}C NMR** (75 MHz, $CDCl_3$) δ 170.9, 170.4, 150.5, 150.1, 141.8, 138.8, 134.6, 132.9, 128.0, 127.3, 119.8, 118.6, 117.9, 116.9, 47.8, 47.8, 45.0, 44.9, 41.2, 40.8; **IR** (ν/cm^{-1}): 2993, 2862, 2832, 2784, 1863, 1777, 1580, 1483, 1319, 1140, 1080, 924, 756; **HRMS** (EI-TOF) *m/z* calculated for $C_{22}H_{22}N_2O_3$ [M$^+$]: 362.1630, found: 362.1627.

1,5-Bis(dimethylamino)-13-phenyl-9,10-dihydro-9,10-[3,4]epipyrroloanthracene-12,14-dione (2cC)

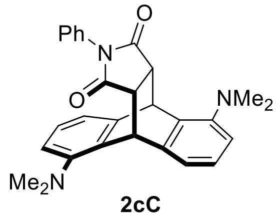

2cC

Following the general procedure, reaction of 1,5-bis(*N,N*-dimethylamino)anthracene (**1c**) (132 mg, 0.50 mmol, 1.0 equiv) and *N*-phenylmaleimide (**C**) (95.2 mg, 0.55 mmol, 1.1 equiv) afforded **2cC** in 0.5 h as white solid in 98% yield (219 mg, 0.49 mmol) after purification by flash column chromatography (DCM:EA = 99:1).

Mp.:209–210 °C; **^{1}H NMR** (400 MHz, $CDCl_3$) δ 7.43–7.37 (m, 3H), 7.25 (dd, J = 7.0, 3.2 Hz, 3H), 7.17 (dd, J = 7.3, 1.1 Hz, 1H), 7.07 (dd, J = 8.1, 1.1 Hz, 1H), 7.03 (dd, J = 6.4, 2.8 Hz, 1H), 6.68–6.60 (m, 2H), 5.54 (d, J = 3.1 Hz, 1H), 5.41 (d, J = 3.0 Hz, 1H), 3.45–3.37 (m, 2H), 2.90 (s, 6H), 2.84 (s, 6H); **^{13}C NMR** (101 MHz, $CDCl_3$) δ 176.3, 175.9, 150.6, 150.1, 142.6, 139.6, 135.2, 133.5, 131.7, 129.0, 128.6, 127.3, 126.9, 126.6, 119.8, 118.6, 117.2, 116.5, 47.0, 46.8, 45.1, 44.9, 41.5, 41.2; **IR** (ν/cm^{-1}): 3063, 2985, 2944, 2862, 2784, 2251, 1777, 1710, 1584, 1483, 1382, 1319, 1189, 909, 790, 726; **HRMS** (ESI-TOF) *m/z* calculated for $C_{28}H_{27}N_3O_2$ [MH$^+$]: 438.2176, found: 438.2181.

Dimethyl 1,5-bis(dimethylamino)-9,10-dihydro-9,10-ethenoanthracene-11,12-dicarboxylate (2cD)

Dimethyl 1,5-bis(dimethylamino)-1,4-dihydro-1,4-ethenoanthracene-2,3-dicarboxylate (3cD)

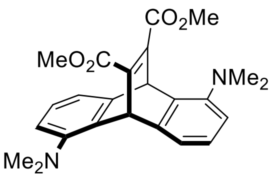

2cD

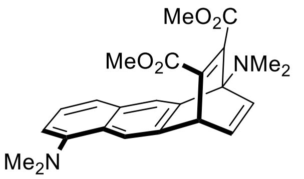

3cD

Following the general procedure, reaction of 1,5-bis(*N,N*-dimethylamino)anthracene (**1c**) (200 mg, 0.76 mmol, 1.0 equiv) and DMAD (**D**) (118 mg, 0.83 mmol, 1.1 equiv) afforded after 24 h **2cD** as white solid in 20% yield (61 mg, 0.15 mmol) and **3cD** as white solid in 58% yield (178 mg, 0.44 mmol) after purification by flash column chromatography (DCM:EA = 24:1).

2cD: **Mp.**: 182–183 °C; **^{1}H NMR** (300 MHz, $CDCl_3$) δ 7.14 (d, J = 7.1 Hz, 2H), 6.95 (t, J = 7.7 Hz, 2H), 6.76 (d, J = 8.0 Hz, 2H), 5.95 (s, 2H), 3.79 (s, 6H), 2.77 (s, 12H); **^{13}C NMR** (75 MHz, $CDCl_3$) δ 166.4, 150.0, 147.8, 145.3, 137.8, 125.8, 118.8, 115.8, 52.4, 48.5, 45.1; **IR** (ν/cm^{-1}): 3056, 2989, 2940, 2870, 2836, 2791, 1736, 1714, 1636, 1587, 1476, 1438, 1271, 1066, 939, 782; **HRMS** (ESI-TOF) *m/z* calculated for $C_{24}H_{27}N_2O_4$ [MH$^+$]: 407.1965, found: 407.1966.

3cD: **Mp.**: 155–156 °C; **^{1}H NMR** (300 MHz, $CDCl_3$) δ 8.03 (s, 1H), 7.86 (s, 1H), 7.46 (d, J = 8.1 Hz, 1H), 7.35 (t, J = 7.8 Hz, 1H), 7.08–7.04 (m, 3H), 5.40 (t, J = 3.9 Hz, 1H), 3.79 (s, 3H), 3.74 (s, 3H), 2.90 (s, 6H), 2.87 (s, 6H); **^{13}C NMR** (75 MHz, $CDCl_3$) δ 168.5, 163.7, 154.2, 150.5, 142.7, 142.1, 141.7, 139.0, 136.5, 132.2, 126.0, 123.5, 122.74, 117.6, 114.8, 78.6, 52.5, 47.7, 45.3, 42.4; **IR** (ν/cm^{-1}): 2996, 2940, 2899, 2829, 1714, 1632, 1587, 1435, 1326, 1259, 1215, 1058, 902, 801, 745, 715; **HRMS** (ESI-TOF) *m/z* calculated for $C_{24}H_{27}N_2O_4$ [MH$^+$]: 407.1965, found: 407.1966.

***Syn*-dimethyl 1,5-di(pyrrolidin-1-yl)-9,10-dihydro-9,10-ethanoanthracene-11,12-dicarboxylate (*syn*-2dA)**

***Anti*-dimethyl 1,5-di(pyrrolidin-1-yl)-9,10-dihydro-9,10-ethanoanthracene-11,12-dicarboxylate (*anti*-2dA)**

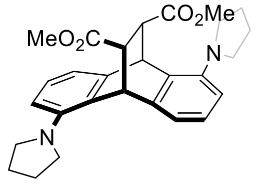

***syn*-2dA**

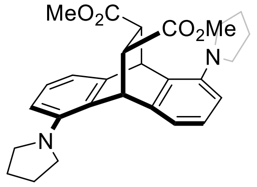

***anti*-2dA**

Following the general procedure, reaction of 1,5-bis(dipyrrolidin-1-yl)anthracene (**1d**) (200 mg, 0.63 mmol, 1.0 equiv) and dimethyl fumarate (**A**) (99.0 mg, 0.70 mmol, 1.1 equiv) afforded after 24 h *syn*-**2dA** as white solid in 55% yield (160 mg, 0.35 mmol) and *anti*-**2dA** as white solid in 31% yield (89.0 mg, 0.19 mmol) after purification by flash column chromatography (DCM:EA = 9:1).

***syn*-2dA**: **Mp.**: 188–189 °C; **^{1}H NMR** (300 MHz, $CDCl_3$) δ 7.03 (t, J = 7.7 Hz, 2H), 6.91 (d, J = 7.0 Hz, 2H), 6.63 (d, J = 8.2 Hz, 2H), 5.29 (s, 2H), 3.71–3.47 (m, 10H), 3.42 (s, 2H), 3.23–3.21 (brs, 4H), 2.06–2.05 (brs, 4H), 1.93–1.89 (brs, 4H); **^{13}C NMR** (75 MHz, $CDCl_3$) δ 173.3, 146.3, 144.2, 128.6, 126.8, 114.9, 113.4, 51.8, 51.8, 47.5, 44.2, 25.7; **IR** (ν/cm^{-1}): 2959, 2866, 1729, 1587, 1476, 1304, 1177, 1088, 1013, 879, 786; **HRMS** (ESI-TOF) *m/z* calculated for $C_{28}H_{33}N_2O_4$ [MH$^+$]: 461.2435, found: 461.2441.

***anti*-2dA**: **Mp.**: 208–209 °C; **^{1}H NMR** (300 MHz, $CDCl_3$) δ 6.98 (t, J = 7.7 Hz, 2H), 6.83 (d, J = 7.0 Hz, 2H), 6.68 (d, J = 8.0 Hz, 2H), 5.18 (s, 2H), 3.66 (s, 6H), 3.53–3.17 (m, 10H), 2.00– (brs, 8H); **^{13}C NMR** (75 MHz, $CDCl_3$) δ 173.3, 145.9, 142.3, 131.3, 126.3, 116.6, 113.6, 52.2, 52.0, 47.7, 43.6, 25.5; **IR** (ν/cm^{-1}): 3060, 3004, 2948, 2851, 1729, 1580, 1476, 1345,1185, 1092, 775; **HRMS** (ESI-TOF) *m/z* calculated for $C_{28}H_{33}N_2O_4$ [MH$^+$]: 461.2435, found: 461.2437.

1,5-Di(pyrrolidin-1-yl)-9,10-dihydro-9,10-[3,4]furanoanthracene-12,14-dione (2dB)

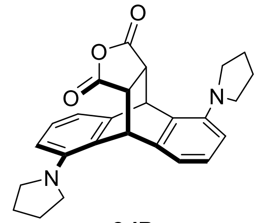

2dB

Following the general procedure, reaction of 1,5-bis(dipyrrolidin-1-yl)anthracene (**1d**) (100 mg, 0.32 mmol, 1.0 equiv) and maleic anhydride (**B**) (34.0 mg, 0.35 mmol, 1.5 equiv) afforded after 0.5 h **2dB** as white solid in 75% yield (98.0 mg, 0.24 mmol) after purification by recrystallization from DCM.

Mp.: 220–221 °C; ^{1}**H NMR** (400 MHz, DMSO-*d*$_6$) δ 7.03–6.97 (m, 3H), 6.75 (d, J = 7.0 Hz, 1H), 6.69 (dd, J = 6.4, 3.0 Hz, 1H), 6.62 (d, J = 7.8 Hz, 1H), 5.31 (d, J = 3.1 Hz, 1H), 5.01 (d, J = 2.7 Hz, 1H), 3.64–3.53 (m, 2H), 3.51-3.39 (m, 2H), 3.36–3.28 (m, 2H), 3.28–3.12 (m, 4H), 2.03–1.78 (m, 8H); ^{13}**C NMR** (101 MHz, DMSO-*d*$_6$) δ 171.8, 171.7, 146.1, 145.8, 143.3, 140.6, 129.2, 127.2, 126.8, 126.1, 115.8, 115.4, 114.0, 113.8, 51.6, 51.3, 48.0, 47.7, 42.2, 41.4, 25.2, 25.0; **IR** (ν/cm^{-1}): 2955, 2862, 1707, 1584, 1476, 1423, 1312, 1222, 1088, 920, 849; **HRMS** (EI-TOF) *m/z* calculated for $C_{26}H_{26}N_2O_3$ [M$^+$]: 414.1943, found: 414.1939.

13-Phenyl-1,5-di(pyrrolidin-1-yl)-9,10-dihydro-9,10-[3,4]epipyrroloanthracene-12,14-dione (2dC)

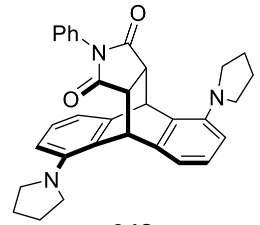

2dC

Following the general procedure, reaction of 1,5-bis(dipyrrolidin-1-yl)anthracene (**1d**) (158 mg, 0.50 mmol, 1.0 equiv) and *N*-phenylmaleimide (**C**) (95.2 mg, 0.55 mmol, 1.1 equiv) afforded after 0.5 h **2dC** as brownish solid in 92% yield (223 mg, 0.46 mmol) after purification by flash column chromatography (DCM:EA = 99:1).

Mp.: 225–226 °C; ^{1}**H NMR** (400 MHz, $CDCl_3$) δ 7.27 (m, 3H), 7.03 (dt, J = 9.5, 7.6 Hz, 2H), 6.94 (d, J = 7.2 Hz, 1H), 6.79 (d, J = 7.2 Hz, 1H), 6.71 (d, J = 8.2 Hz, 1H), 6.64 (d, J = 8.3 Hz, 1H), 6.60–6.51 (m, 2H), 5.44 (d, J = 2.7 Hz, 1H), 5.22 (d, J = 2.3 Hz, 1H), 3.52 (q, J = 7.8 Hz, 2H), 3.46–3.38 (m, 2H), 3.31–3.17 (m, 6H), 1.97 (m, 6H), 1.90–1.77 (m, 2H); ^{13}**C NMR** (101 MHz, $CDCl_3$) δ 176.6, 176.4, 146.7, 146.4, 143.7, 140.5, 131.8, 130.4, 129.0, 128.5, 127.2, 126.8, 126.69, 126.65, 115.8, 115.6, 113.94, 113.91, 52.0, 51.8, 47.1, 47.0, 43.2, 42.8, 25.6, 25.4; **IR** (ν/cm^{-1}): 3063, 2967, 2870, 2825, 2251, 1774, 1710, 1580, 1476, 1382, 1356, 1185, 909, 726; **HRMS** (ESI-TOF) *m/z* calculated for $C_{32}H_{31}N_3O_2$ [MH$^+$]: 490.2489, found: 490.2496.

Dimethyl 1,5-di(pyrrolidin-1-yl)-1,4-dihydro-1,4-ethenoanthracene-2,3-dicarboxylate (3dD)

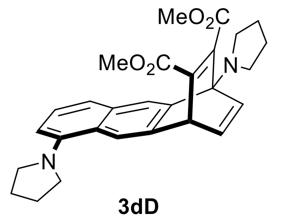

3dD

Following the general procedure, reaction of 1,5-bis(dipyrrolidin-1-yl)anthracene (**1d**) (200 mg, 0.63 mmol, 1.0 equiv) and DMAD (**D**) (99.0 mg, 0.70 mmol, 1.1 equiv) afforded after 24 h **3dD** as white solid in 78% yield (226 mg, 0.49 mmol) after purification by flash column chromatography (DCM:EA = 19:1).

Mp.: 199–200 °C; **^{1}H NMR** (300 MHz, $CDCl_3$) δ 7.98 (s, 1H), 7.87 (s, 1H), 7.39–7.28 (m, 2H), 7.08–7.04 (m, 1H), 7.01–6.93 (m, 2H), 5.40 (dd, J = 6.0, 1.6 Hz, 1H), 3.80 (s, 3H), 3.73 (s, 3H), 3.53-3.29 (m, 6H), 3.29-3.16 (m, 2H), 2.03 (brs, 8H); **^{13}C NMR** (75 MHz, $CDCl_3$) δ 168.1, 163.8, 154.9, 147.4, 142.3, 142.0, 140.6, 139.3, 132.4, 125.9, 125.5, 122.6, 122.1, 118.1, 112.7, 79.0, 52.8, 52.7, 52.4, 51.4, 47.8, 26.3, 24.7; **IR** (ν/cm^{-1}): 2952, 2870, 2840, 1729, 1625, 1591, 1453, 1319, 1252, 1107, 1062, 749; **HRMS** (ESI-TOF) *m/z* calculated for $C_{28}H_{31}N_2O_4$ [MH$^+$] 459.2278, found: 459.2280.

Methyl 1,5-di(pyrrolidin-1-yl)-9,10-dihydro-9,10-ethenoanthracene-11-carboxylate (2dE)

Methyl 1,5-di(pyrrolidin-1-yl)-1,4-dihydro-1,4-ethenoanthracene-2-carboxylate (3dE)

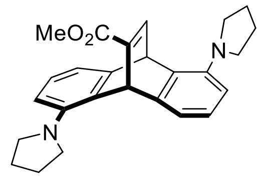

2dE

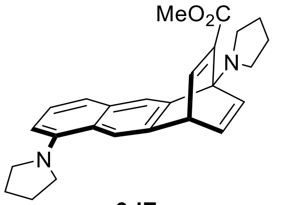

3dE

Following the general procedure, reaction of 1,5-bis(dipyrrolidin-1-yl)anthracene (**1d**) (200 mg, 0.63 mmol, 1.0 equiv) and methyl propiolate (**E**) (62.0 µL, 0.70 mmol, 1.1 equiv) afforded after 24 h **2dE** as white solid in 14% yield (35.0 mg, 0.09 mmol) and **3cE** as white solid in 56% yield (142 mg, 0.35 mmol) after purification by flash column chromatography (DCM:EA = 19:1 to 3:2).

2dE: **Mp.**: 118–119 °C; **^{1}H NMR** (400 MHz, $CDCl_3$) δ 7.87 (dd, J = 6.3, 1.9 Hz, 1H), 6.98 (d, J = 7.2 Hz, 1H), 6.91–6.83 (m, 3H), 6.55 (dd, J = 8.3, 1.0 Hz, 1H), 6.51 (d, J = 8.2 Hz, 1H), 6.20 (d, J = 1.9 Hz, 1H), 5.69 (d, J = 6.4 Hz, 1H), 3.70 (s, 3H), 3.49–3.38 (m, 6H), 3.31–3.24 (m, 2H), 2.03–1.95 (m, 8H); **^{13}C NMR** (101 MHz, $CDCl_3$) δ 165.57, 150., 147.7, 146.6, 146.2, 146.0, 144.9, 133.58 132.9, 125.4, 125.3, 116.0, 114.9, 112.7, 112.5, 52.2, 51.9, 51.7, 49.1, 47.8, 25.8, 25.5; **IR** (ν/cm^{-1}): 3034, 2944, 2866, 1710, 1625, 1580, 1461, 1330, 1237, 1092, 730; **HRMS** (ESI-TOF) *m/z* calculated for $C_{26}H_{28}N_2O_2$ [MH$^+$]: 401.2224, found: 401.2233.

3dE: **Mp.**: 201–202 °C; **^{1}H NMR** (400 MHz, $CDCl_3$) δ 7.89 (s, 2H), 7.43 (d, J = 6.3 Hz, 1H), 7.39 (d, J = 8.1 Hz, 1H), 7.29 (t, J = 7.8 Hz, 1H), 7.00 (d, J = 3.8 Hz, 2H), 6.96 (d, J = 7.5 Hz, 1H), 4.90 (dt, J = 7.2, 3.9 Hz, 1H), 3.70 (s, 3H), 3.37–3.21 (m, 8H), 2.08–1.98 (m, 8H); **^{13}C NMR** (101 MHz, $CDCl_3$) δ 166.7, 148.7, 147.4, 146.0, 143.8, 141.2, 139.6, 138.5, 132.5,

125.7, 125.4, 122.2, 121.7, 117.5, 112.5, 52.8, 52.0, 51.3, 48.0, 26.0, 24.7; **IR** (ν/cm^{-1}): 3052, 2948, 2866, 2817, 1714, 1621, 1580, 1457, 1364, 1289, 1230, 1073, 894, 738; **HRMS** (ESI-TOF) *m/z* calculated for $C_{26}H_{28}N_2O_2$ [MH$^+$]: 401.2224, found: 401.2232.

Methyl 12-phenyl-1,5-di(pyrrolidin-1-yl)-9,10-dihydro-9,10-ethenoanthracene-11-carboxylate (2dF)

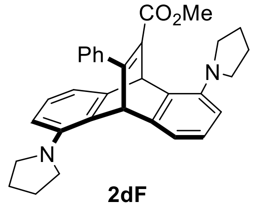

2dF

Following the general procedure, reaction of 1,5-bis(dipyrrolidin-1-yl)anthracene (**1d**) (200 mg, 0.63 mmol, 1.0 equiv) and methyl phenylpropiolate (**F**) (506 mg, 3.16 mmol, 5.0 equiv) afforded after 72 h **2dF** as white solid in 23% yield (69.0 mg, 0.14 mmol) after purification by flash column chromatography (hexanes:DCM = 2:3).

Mp.: 188–189 °C; **^{1}H NMR** (300 MHz, $CDCl_3$) *δ* 7.32 (d, *J* = 5.3 Hz, 5H), 7.04 (d, *J* = 6.5 Hz, 1H), 7.00–6.76 (m, 3H), 6.58 (d, *J* = 8.0 Hz, 2H), 6.34 (s, 1H), 5.81 (s, 1H), 3.70–3.42 (m, 7H), 3.42–3.03 (m, 4H), 2.59–1.53 (m, 8H); **^{13}C NMR** (75 MHz, $CDCl_3$) *δ* 166.5, 162.1, 147.6, 146.6, 146.2, 145.8, 138.8, 136.9, 133.0, 132.3, 127.9, 127.7 (2C), 125.6, 125.5, 115.4, 114.5, 112.6, 112.5, 57.6, 52.1, 51.8, 51.4, 49.6, 25.9, 25.6; **IR** (ν/cm^{-1}): 3049, 2948, 2836, 1714, 1621, 1584, 1476, 1334, 1192, 1088, 775; **HRMS** (ESI-TOF) *m/z* calculated for $C_{32}H_{33}N_2O_2$ [MH$^+$] 477.2537, found: 477.2538.

***Syn*-dimethyl 1-(pyrrolidin-1-yl)-9,10-dihydro-9,10-ethanoanthracene-11,12-dicarboxylate (*syn*-2eA)**

***Anti*-dimethyl 1-(pyrrolidin-1-yl)-9,10-dihydro-9,10-ethanoanthracene-11,12-dicarboxylate (*anti*-2eA)**

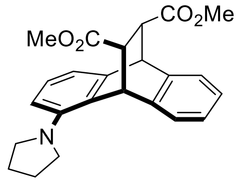

***syn*-2eA**

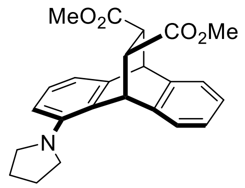

***anti*-2eA**

Following the general procedure, reaction of 1-(pyrrolidine-1-yl)anthracene (**1e**) (200 mg, 0.81 mmol, 1.0 equiv) and dimethyl fumarate (**A**) (128 mg, 0.89 mmol, 1.1 equiv) afforded after 24 h *syn*-**2eA** as white solid in 46% yield (147 mg, 0.37 mmol) and *anti*-**2eA** as white solid in 50% yield (157 mg, 0.40 mmol) after purification by flash column chromatography (DCM to DCM:EA = 19:1).

***syn*-2eA**: **Mp.**: 138–139 °C; **^{1}H NMR** (400 MHz, $CDCl_3$) *δ* 7.52–7.47 (m, 1H), 7.42–7.39 (m, 1H), 7.31–7.22 (m, 2H), 7.16 (dd, *J* = 8.2, 7.2 Hz, 1H), 7.03 (d, *J* = 7.1 Hz, 1H), 6.78 (dd, *J* = 8.3, 1.0 Hz, 1H), 5.46 (d, *J* = 2.9 Hz, 1H), 4.84 (d, *J* = 2.8 Hz, 1H), 3.76 (s, 3H), 3.74 (s, 3H), 3.69–3.60 (m, 3H), 3.51 (dd, *J* = 4.7, 2.9 Hz, 1H), 3.32 (td, *J* = 8.0, 4.6 Hz, 2H), 2.25–2.13 (m, 2H), 2.10–1.98 (m, 2H); **^{13}C NMR** (101 MHz, $CDCl_3$) *δ* 173.1, 173.0, 146.3, 142.5, 140.8, 128.6, 126.9, 126.3, 126.2, 124.7, 123.2, 115.5, 113.7, 52.2, 51.9, 51.8, 47.8, 47.6, 47.4, 43.5,

25.7; **IR** (ν/cm^{-1}): 3052, 3022, 2952, 2870, 1729, 1580, 1479, 1435, 1356, 1259, 1192, 1006, 868, 771; **HRMS** (ESI-TOF) *m/z* calculated for $C_{24}H_{25}NO_4$ [MH$^+$]: 392.1856, found: 392.1858.

***anti*-2eA**: **Mp.**: 106–107 °C; **^{1}H NMR** (400 MHz, $CDCl_3$) δ 7.51–7.46 (m, 1H), 7.44–7.41 (m, 1H), 7.30–7.22 (m, 2H), 7.13 (dd, *J* = 8.2, 7.2 Hz, 1H), 6.96 (d, *J* = 7.1 Hz, 1H), 6.84 (dd, *J* = 8.3, 1.0 Hz, 1H), 5.37 (d, *J* = 2.6 Hz, 1H), 4.85 (d, *J* = 2.6 Hz, 1H), 3.81 (s, 3H), 3.78 (s, 3H), 3.61 (dd, *J* = 5.0, 2.6 Hz, 1H), 3.58–3.43 (m, 5H), 2.21–2.09 (m, 4H); **^{13}C NMR** (101 MHz, $CDCl_3$) δ 173.0, 172.9, 146.0, 142.6, 141.8, 140.7, 130.9, 126.4, 126.2, 126.1, 124.3, 123.6, 116.8, 113.8, 52.2, 52.0, 47.9, 47.6, 47.3, 42.9, 25.4; **IR** (ν/cm^{-1}): 3019, 2955, 2577, 2814, 1729, 1587, 1476, 1435, 1304, 1177, 1010, 872, 782, 738; **HRMS** (ESI-TOF) *m/z* calculated for $C_{24}H_{25}NO_4$ [MH$^+$]: 392.1856, found: 392.1863.

***Exo*-1-(pyrrolidin-1-yl)-9,10-dihydro-9,10-[3,4]furanoanthracene-12,14-dione (*exo*-2eB)**

***Endo*-1-(pyrrolidin-1-yl)-9,10-dihydro-9,10-[3,4]furanoanthracene-12,14-dione (*endo*-2eB)**

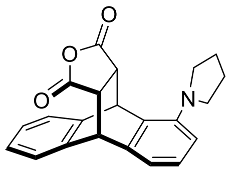

***exo*-2eB**

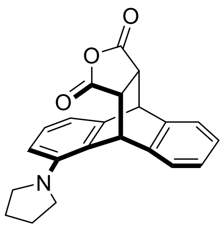

***endo*-2eB**

Following the general procedure, reaction of 1-(pyrrolidine-1-yl)anthracene (**1e**) (200 mg, 0.81 mmol, 1.0 equiv) and maleic anhydride (**B**) (128 mg, 0.89 mmol, 1.1 equiv) afforded after 0.5 h a crude mixture of *exo*-**2eB** and *endo*-**2eB** (NMR yield > 99%, ratio *endo*-**2eB**/*exo*-**2eB** = 67:33) as white solid. During purification by flash column chromatography (DCM to DCM:EA = 19:1) decomposition of both products was observed and only ***endo*-2eB** could be isolated as white solid in 34% yield (94.0 mg, 0.27 mmol).

***endo*-2eB**: **Mp.**: 218–219 °C; **^{1}H NMR** (400 MHz, $CDCl_3$) δ 7.34 (dt, *J* = 8.5, 4.0 Hz, 2H), 7.20 (dd, *J* = 5.5, 3.2 Hz, 2H), 7.06 (t, *J* = 7.7 Hz, 1H), 6.93 (d, *J* = 7.2 Hz, 1H), 6.74 (d, *J* = 8.2 Hz, 1H), 5.29 (d, *J* = 3.1 Hz, 1H), 4.77 (d, *J* = 3.2 Hz, 1H), 3.57–3.37 (m, 4H), 3.26 (dt, *J* = 8.6, 5.1 Hz, 2H), 2.08–1.92 (m, 4H); **^{13}C NMR** (101 MHz, $CDCl_3$) δ 170.9, 170.8, 146.7, 142.4, 138.6, 129.3, 127.6, 127.4, 125.2, 124.8, 116.3, 114.6, 52.2, 48.1, 48.0, 46.1, 42.1, 25.5; **IR** (ν/cm^{-1}): 3026, 2970, 2825, 1859, 1774, 1580, 1476, 1356, 1222, 1077, 928, 756; **HRMS** (EI-TOF) *m/z* calculated for $C_{22}H_{19}NO_3$ [M$^+$]: 345.1359, found: 345.1353.

***Exo*-13-phenyl-4-(pyrrolidin-1-yl)-9,10-dihydro-9,10-[3,4]epipyrroloanthracene-12,14-dione (*exo*-2eC)**

***Endo*-13-phenyl-4-(pyrrolidin-1-yl)-9,10-dihydro-9,10-[3,4]epipyrroloanthracene-12,14-dione (*endo*-2eC)**

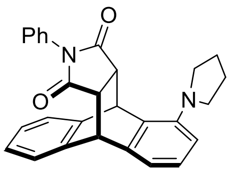

***exo*-2eC**

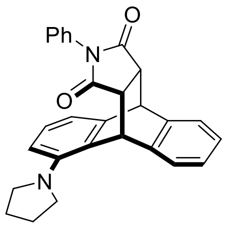

***endo*-2eC**

Following the general procedure, reaction of 1-(pyrrolidine-1-yl)anthracene (**1e**) (200 mg, 0.81 mmol, 1.0 equiv) and *N*-phenylmaleimide (**C**) (154 mg, 0.89 mmol, 1.1 equiv) afforded after 0.5 h a mixture of *exo*-**2eC** and *endo*-**2eC** (ratio *endo*-**2eC**/*exo*-**2eC** = 67:33) as white solid after purification by flash column chromatography (DCM:EA = 99:1) in 95% yield (324 mg, 0.77 mmol).

Characteristic signals of minor compound ***exo*-2eC** are marked in the proton NMR.

***exo*-2eC and *endo*-2eC**: **Mp.**:177–178 °C; **^{1}H NMR** (400 MHz, $CDCl_3$) δ 7.50–7.13 (m, 13H), 7.05 (q, J = 7.5 Hz, 2H), 6.96 (d, J = 7.2 Hz, 1H), 6.81^{minor} (t, J = 3.7 Hz, 2H), 6.74 (d, J = 8.2 Hz, 1H), 6.67^{minor} (d, J = 8.3 Hz, 1H), 6.64–6.54 (m, 1H), 6.49 (dd, J = 7.0, 2.7 Hz, 2H), 5.51^{minor} (d, J = 3.2 Hz, 1H), 5.32 (d, J = 3.1 Hz, 1H), 4.82 (d, J = 3.3 Hz, 1H), 4.80^{minor} (d, J = 3.1 Hz, 1H), 3.54 (q, J = 7.8 Hz, 1H), 3.44 (q, J = 6.9 Hz, 2H), 3.40–3.15 (m, 6H), 2.06–1.92 (m, 5H), 1.87 (td, J = 10.6, 8.3, 5.7 Hz, 1H).; **^{13}C NMR** (101 MHz, $CDCl_3$) δ 176.47, 176.42, 176.3, 176.1, 146.7, 146.5, 143.1, 141.9, 140.0, 139.1, 134.2, 131.7, 131.5, 130.0, 129.1, 129.09, 129.05, 128.7, 128.6, 127.9, 127.3, 127.05, 127.00, 126.66, 126.60, 126.5, 126.4, 126.0, 125.1, 124.7, 124.3, 123.6, 116.3, 116.2, 114.2, 114.1, 52.1, 51.8, 47.2, 47.1, 47.0, 46.5, 46.4, 42.4, 42.1, 25.6, 25.4; **IR** (ν/cm^{-1}): 3067, 2967, 2870, 2825, 2251, 1774, 1707, 1587, 1498, 1382, 1185, 909, 723; **HRMS** (ESI-TOF) *m/z* calculated for $C_{28}H_{24}N_2O_2$ [MH^+]: 421.1911, found: 421.1913 and 421.1916.

Dimethyl 1-(pyrrolidin-1-yl)-1,4-dihydro-1,4-ethenoanthracene-2,3-dicarboxylate (3eD)

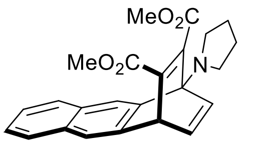

3eD

Following the general procedure, reaction of 1-(pyrrolidine-1-yl)anthracene (**1e**) (282 mg, 1.14 mmol, 1.0 equiv) and DMAD (**D**) (178 mg, 1.25 mmol, 1.1 equiv) afforded after 24 h **3eD** as white solid in 90% yield (403 mg, 1.03 mmol) after purification by flash column chromatography (DCM:EA = 19:1).

Mp.: 159–160 °C; **^{1}H NMR** (400 MHz, $CDCl_3$) δ 7.89 (s, 1H), 7.77–7.68 (m, 2H), 7.63 (s, 1H), 7.45–7.39 (m, 2H), 7.07–6.99 (m, 2H), 5.38 (dd, J = 5.9, 1.9 Hz, 1H), 3.80 (s, 3H), 3.74 (s, 3H), 3.39 (dt, J = 10.1, 6.2, 3.0 Hz, 4H), 2.06–1.98 (m, 4H); **^{13}C NMR** (101 MHz, $CDCl_3$) δ 168.1, 163.8, 155.2, 142.6, 142.0, 141.8, 139.5, 139.0, 131.05 , 131.02, 128.3, 127.2, 126.2, 125.9,

122.0, 121.1, 79.1, 52.7, 52.5, 51.4, 47.3, 26.4; **IR** (ν/cm^{-1}): 3034, 2952, 2870, 2836, 1722, 1628, 1595, 1427, 1312, 1237, 1203, 1110, 1051, 883, 749; **HRMS** (ESI-TOF) *m/z* calculated for $C_{24}H_{23}NO_4$ [MH$^+$] 390.1700, found: 390.1700.

Methyl 4-(pyrrolidin-1-yl)-9,10-dihydro-9,10-ethenoanthracene-11-carboxylate (*syn*-2eE)

Methyl 1-(pyrrolidin-1-yl)-9,10-dihydro-9,10-ethenoanthracene-11-carboxylate (*anti*-2eE)

Methyl 1-(pyrrolidin-1-yl)-1,4-dihydro-1,4-ethenoanthracene-2-carboxylate (3eE)

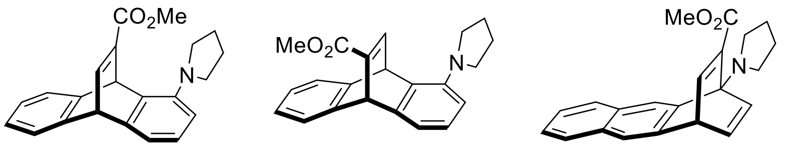

***syn*-2eE** ***anti*-2eE** **3eE**

Following the general procedure, reaction of 1-(pyrrolidine-1-yl)anthracene (**1e**) (200 mg, 0.81 mmol, 1.0 equiv) and methyl propiolate (**E**) (79.0 µL, 0.89 mmol, 1.1 equiv) afforded after 24 h a mixture of *syn*-**2eE** and *anti*-**2eE** (ratio: *syn*-**2eE**/ *anti*-**2eE** = 55:45) as a white solid in 25% yield (67.0 mg, 0.20 mmol) and **3eE** as a white solid in 72% yield (193 mg, 0.58 mmol) after purification by flash column chromatography (DCM:EA = 19:1 to 3:2).

Characteristic signals of minor compound *anti*-**2eE** are marked in the proton NMR.

***syn*-2eE and *anti*-2eE**: **Mp.**: 64–65 °C; **^{1}H NMR** (400 MHz, $CDCl_3$) δ 7.91 (dd, J = 6.2, 1.8 Hz, 1H), 7.87^{minor} (dd, J = 6.3, 1.8 Hz, 1H), 7.42–7.31 (m, 4H), 7.00 (qd, J = 5.3, 4.7, 2.3 Hz, 5H), 6.94–6.86 (m, 3H), 6.60^{minor} (d, J = 8.1 Hz, 1H), 6.54 (quin, J = 3.5 Hz, 1H), 6.27 (d, J = 1.8 Hz, 1H), 5.77^{minor} (d, J = 6.3 Hz, 1H), 5.66^{minor} (d, J = 1.9 Hz, 1H), 5.21 (d, J = 6.2 Hz, 1H), 3.74 (d, J = 1.5 Hz, 6H), 3.51–3.46 (m, 4H), 3.45–3.38^{minor} (m, 2H), 3.29^{minor} (dq, J = 9.1, 5.4, 4.2 Hz, 2H), 2.01 (qt, J = 7.2, 3.4 Hz, 8H); **^{13}C NMR** (101 MHz, $CDCl_3$) δ 165.5, 165.3, 150.4, 149.7, 147.2, 146.4, 146.3, 146.04, 146.02, 145.7, 145.14, 145.13, 144.5, 144.3, 134.7, 133.0, 132.4, 131.3, 125.58 , 125.51 , 124.95 , 124.94 , 124.8, 124.7, 123.7, 123.4, 123.3, 123.1, 116.4 , 115.3, 112.9, 112.7, 52.6, 52.1, 51.9, 51.84, 51.82, 51.1, 48.1, 47.1, 25.7, 25.4; **IR** (ν/cm^{-1}): 3063, 2948, 2870, 1707, 1617, 1587, 1435, 1330, 1244, 1214, 1066, 946, 745; **HRMS** (ESI-TOF) *m/z* calculated for $C_{22}H_{21}NO_2$ [MH$^+$]: 332.1645, found: 332.1651 and 332.1649.

3eE: **Mp.**: 84–85 °C; **^{1}H NMR** (400 MHz, $CDCl_3$) δ 7.93 (s, 1H), 7.78–7.73 (m, 1H), 7.69–7.64 (m, 1H), 7.52 (s, 1H), 7.44–7.38 (m, 3H), 7.03–6.97 (m, 2H), 4.88 (dt, J = 6.4, 3.8 Hz, 1H), 3.71 (s, 3H), 3.38–3.23 (m, 4H), 2.04 (dt, J = 12.9, 7.6, 6.7, 3.2 Hz, 4H); **^{13}C NMR** (101 MHz, $CDCl_3$) δ 166.7, 148.4, 146.1, 144.1, 142.2, 139.6, 138.4, 131.1, 130.8, 128.2, 127.1, 125.9, 125.8, 121.2, 120.5, 52.0, 51.4, 47.5, 26.0; **IR** (ν/cm^{-1}): 3056, 2948, 2866, 1714, 1621, 1580, 1431, 1289, 1226, 1196, 1077, 883, 745; **HRMS** (ESI-TOF) *m/z* calculated for $C_{22}H_{21}NO_2$ [MH$^+$]: 332.1645, found: 332.1650.

1-Phenyl-3-(4-(pyrrolidin-1-yl)anthracen-1-yl)pyrrolidine-2,5-dione (8)

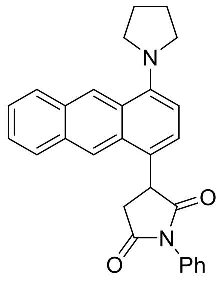

8

A sealed tube was charged with 1-(pyrrolidine-1-yl)anthracene (**1e**) (100 mg, 0.40 mmol, 1.0 equiv), *N*-phenylmaleimide (**C**) (77.0 mg, 0.44 mmol, 1.1 equiv), $AlCl_3$ (162 mg, 1.21 mmol, 3.0 equiv) and $CHCl_3$ (5.00 mL). The mixture was dispersed in an ultrasonic bath for 1 min and afterwards stirred at room temperature for 3 h. The reaction was quenched with water (10 mL) and the aqueous phase was extracted with DCM (3 × 10 mL). The combined organic phases were washed with brine, dried over anhydrous Na_2SO_4 and the solvent was removed under reduced pressure. After purification by flash column chromatography (hexanes:DCM = 1:2 to DCM) product **8** was obtained as a brown solid in 62% yield (106 mg, 0.25 mmol).

Mp.: 94–95 °C; **^{1}H NMR** (400 MHz, $CDCl_3$) δ 8.88 (s, 1H), 8.33 (s, 1H), 8.06–7.96 (m, 2H), 7.56–7.44 (m, 7H), 7.28 (d, *J* = 7.7 Hz, 1H), 6.84 (d, *J* = 7.7 Hz, 1H), 4.95 (dd, *J* = 9.7, 4.8 Hz, 1H), 3.59 (dd, *J* = 18.5, 9.7 Hz, 1H), 3.46 (td, *J* = 9.4, 7.0, 3.0 Hz, 4H), 3.04 (dd, *J* = 18.5, 4.9 Hz, 1H), 2.11–2.05 (m, 4H); **^{13}C NMR** (101 MHz, $CDCl_3$) δ 177.6, 175.4, 148.6, 132.2, 131.6, 130.9, 130.5, 129.4, 128.8, 128.6, 128.0, 127.9, 126.6, 126.2, 125.9, 125.5, 125.3, 121.4, 108.7, 52.9, 43.5, 37.9, 25.0; **IR** (ν/cm^{-1}): 3049, 2963, 2870, 2817, 1777, 1707, 1561, 1498, 1375, 1170, 745, 693; **HRMS** (ESI-TOF) *m/z* calculated for $C_{28}H_{24}N_2O_2$ [MH^+]: 421.1911, found: 421.1918.

4-Methyl-1-(4-(pyrrolidin-1-yl)anthracen-1-yl)-1,2,4-triazolidine-3,5-dione (9)

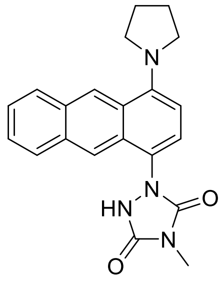

9

Under a nitrogen atmosphere 1-(pyrrolidine-1-yl)anthracene (**1e**) (247 mg, 1.00 mmol, 2.0 equiv), NEt_3 (279 µL, 2.00 mmol, 4.0 equiv) and MTAD (57.0 mg, 0.50 mmol, 1.0 equiv) were dissolved in degassed acetone (5.00 mL) and stirred under irradiation with a green LED (λ = 530 nm) at –78 °C for 5 h. The solvent was removed under reduced pressure and the crude product was purified by flash column chromatography (DCM to DCM:MeOH = 19:1) to afford product **9** as a brown solid in 60% yield (107 mg, 0.30 mmol).

Mp.: 191–192 °C; **^{1}H NMR** (400 MHz, CD_2Cl_2) δ 8.79 (s, 1H), 8.24 (s, 1H), 8.00–7.98 (m, 1H), 7.92–7.88 (m, 1H), 7.48–7.43 (m, 2H), 7.21 (d, *J* = 8.0 Hz, 1H), 6.57 (d, *J* = 8.0 Hz, 1H),

3.51 – 3.45 (m, 4H), 3.12 (s, 3H), 2.07–2.01 (m, 4H); **^{13}C NMR** (101 MHz, CD_2Cl_2) δ 155.3, 153.3, 149.9, 132.1, 130.9, 130.0, 129.1, 128.3, 127.1, 126.9, 126.6, 126.0, 125.6, 123.8, 121.3, 107.1, 53.4, 25.7; **IR** (ν/cm^{-1}): 3049, 2952, 2866, 1766, 1692, 1617, 1453, 1386, 1323, 1148, 1021, 875, 741; **HRMS** (ESI-TOF) *m/z* calculated for $C_{21}H_{20}N_4O_2$ [MH^+]: 361.1659, found: 361.1666.

1-(2,4-Dibromoanthracen-1-yl)pyrrolidine (10)

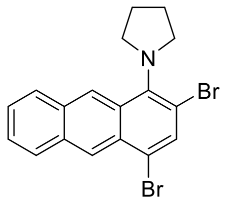

10

1-(pyrrolidine-1-yl)anthracene (**1e**) (200 mg, 0.81 mmol, 1.0 equiv) and NEt_3 (676 µL, 4.85 mmol, 6.0 equiv) were dissolved in DCM (10.0 mL) and the resulting solution was cooled to 0 °C. Then NBS (864 mg, 4.85 mmol, 6.0 equiv) was added portionwise over a period of 15 min and the reaction mixture was stirred at 0 °C for 5 h. The mixture was washed with saturated K_2CO_3 solution (15 mL) and the aqueous phase was extracted with DCM (3 × 10 mL). The combined organic phases were dried over anhydrous K_2CO_3 and the solvent was removed under reduced pressure. The black crude product was purified by flash column chromatography (hexanes) and product **10** was obtained as a bright yellow solid in 58% yield (191 mg, 0.47 mmol).

Mp.: 138–139 °C; **^{1}H NMR** (400 MHz, $CDCl_3$) δ 8.80 (s, 1H), 8.73 (s, 1H), 8.09–8.03 (m, 2H), 7.92 (s, 1H), 7.57–7.50 (m, 2H), 3.53–3.48 (m, 4H), 2.23–2.18 (m, 4H); **^{13}C NMR** (101 MHz, $CDCl_3$) δ 143.9, 133.6, 133.4, 132.4, 132.3, 130.1, 128.6, 128.5, 127.1, 126.6, 126.4, 124.2, 120.2, 119.0, 50.8, 27.0; **IR** (ν/cm^{-1}): 3052, 2967, 2862, 2814, 2668, 1677, 1595, 1558, 1442, 1293, 1140, 939, 838, 872, 715; **HRMS** (EI-TOF) *m/z* calculated for $C_{18}H_{15}NBr_2$ [M^+]: 402.9565, found: 402.9555.

Dimethyl (1*R*,4*S*)-1-(pyrrolidin-1-yl)-1,4-dihydro-1,4-ethanoanthracene-2,3-dicarboxylate (11)

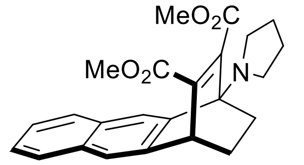

11

Dimethyl (1*R*,4*S*)-1-(pyrrolidin-1-yl)-1,4-dihydro-1,4-ethenoanthracene-2,3-dicarboxylate (**3eD**) (50 mg, 128 µmol, 1.0 equiv) and ammonium formate (163 mg, 2.57 mmol, 20.0 equiv) were dissolved in dry methanol (5 mL) and Pd/C (13.7 mg, 10 w%, 12.8 µmol, 0.1 equiv) was added. The reaction mixture was stirred for one hour until complete conversion of the starting material was observed. The mixture was filtered over celite and washed with ethyl acetate. The organic phase was washed two times with brine and dried over $MgSO_4$. The solvent was removed under reduced pressure. The pure product was obtained as a yellowish oil (49 mg, 125 µmol, 98%).

R$_f$ (PE:EA = 1:1): 0.64; **^{1}H NMR** (400 MHz, $CDCl_3$) δ 7.83–7.77 (m, 4H), 7.70 (s, 1H), 7.48–7.40 (m, 2H), 4.57 (t, J = 2.7 Hz, 1H), 3.79 (s, 3H), 3.79 (s, 3H), 3.59–3.49 (m, 2H), 3.27–3.22 (m, 2H), 2.23–2.16 (m, 1H), 2.08–1.90 (m, 4H), 1.89–1.78 (m, 2H), 1.67–1.60 (m, 1H); **^{13}C NMR** (101 MHz, $CDCl_3$) δ 168.9, 163.7, 152.6, 139.7, 135.9, 132.00, 131.9, 128.2, 127.3, 125.8, 125.4, 121.5, 121.2, 69.8, 52.3, 52.3, 48.9, 39.4, 32.9, 27.3, 26.2; **IR** (ν/cm^{-1}): 3056, 2948, 2870, 2255, 1714, 1621, 1502, 1435, 1334, 1308, 1248, 1196, 1133, 1069, 954, 909, 805, 730; **HRMS** (ESI-TOF) *m/z* calculated for $C_{24}H_{26}NO_4$ [MH^+]: 392.1862 found: 392.1860.

4. Optimization Studies for 1,3-Dipolar Cycloaddition

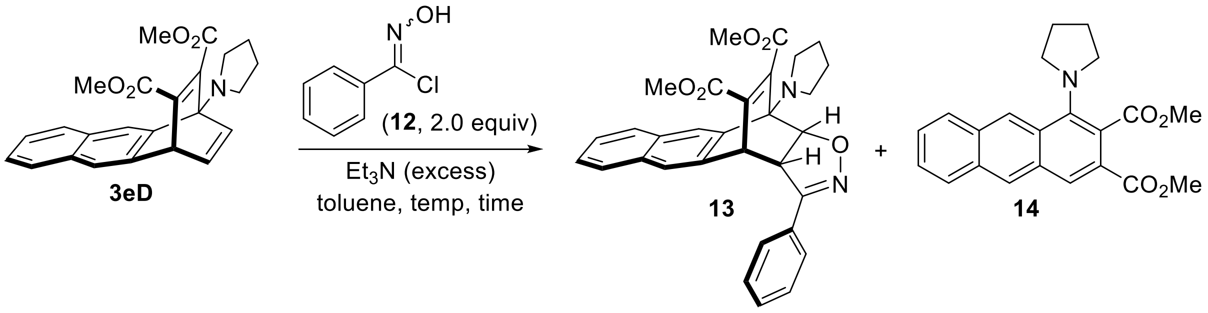

entry	temperature [°C]	time [h]	yield 13 [%]	yield 14 [%]
1	25 to reflux	24	-	41
2	25	17	45	14
3	reflux	17	-	76
4	25	5	60	21
5	0	4	47	22
6	0	6	74	21

Method. Dimethyl (1*R*,4*S*)-1-(pyrrolidin-1-yl)-1,4-dihydro-1,4-ethenoanthracene-2,3-dicarboxylate (**3eD**) (50 mg, 128 µmol, 1.0 equiv) was dissolved in dry toluene (1 mL) and triethylamine (0.5 mL) was added. The solution was cooled down to 0 °C and *N*-hydroxybenzimidoyl chloride (40 mg, 257 µmol, 2.0 equiv) was added slowly over one hour. The reaction mixture was stirred at 0 °C for six hours. Then, toluene was added. The organic phase was washed three times with distilled water and dried over anhydrous $NaSO_4$. The solvent was removed under reduced pressure and the crude product was purified by column chromatography (PE:EA = 95:5, 10% NEt_3). Dimethyl 1-(pyrrolidin-1-yl)anthracene-2,3-dicarboxylate (**14**) was isolated as a red oil (9.7 mg, 26.7 μmol, 21%). Dimethyl-3-phenyl-11-(pyrrolidin-1-yl)-3a,4,11,11a-tetrahydro-4,11-ethenoanthra[2,3-*d*]isoxazole-12,13-dicarboxylate (**13**) was isolated as a yellow oil as a mixture of isomers (48.2 mg, 94.8 μmol, 74%). The major isomer was separated partially by column chromatography (CH_2Cl_2:acetone = 95:5, 10% NEt_3).

Dimethyl (4*S*,11*R*,11a*S*)-3-phenyl-11-(pyrrolidin-1-yl)-3a,4,11,11a-tetrahydro-4,11-ethenoanthra[2,3-*d*]isoxazole-12,13-dicarboxylate (major isomer, 13)

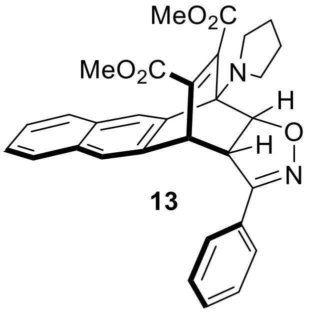

13

R*f* (PE:EA = 1:1): 0.64; **^{1}H NMR** (400 MHz, CDCl3) δ 7.88 (s, 1H), 7.83 (dt, *J* = 7.6, 4.6 Hz, 2H), 7.79 (s, 1H), 7.71 (dd, *J* = 6.6, 3.0 Hz, 2H), 7.52–7.48 (m, 2H), 7.44 (dd, *J* = 5.0, 1.9 Hz, 3H), 5.19 (d, *J* = 9.8 Hz, 1H), 4.93 (d, *J* = 2.9 Hz, 1H), 4.19 (dd, *J* = 9.8, 2.9 Hz, 1H), 3.84 (s, 3H), 3.65 (dd, *J* = 9.1, 5.5 Hz, 2H), 3.35 (s, 1H), 3.35 – 3.30 (m, 4H), 2.14 – 1.99 (m, 4H) ; **^{13}C NMR** (101 MHz, $CDCl_3$) δ 167.5, 162.6, 155.7, 151.1, 137.5, 135.1, 133.1, 132.3, 131.9, 130.1, 128.8, 128.5, 128.4, 127.3, 126.9, 126.7, 126.2, 124.5, 122.4, 88.1, 73.1, 58.3, 52.7, 51.9, 49.3, 40.7, 26.2; **IR** (ν/cm^{-1}): 3056, 2952, 2855, 2251, 1722, 1625, 1498, 1435, 1334, 1252, 1211, 1140, 1058, 1025, 957, 909, 849, 816, 760, 730, 693; **HRMS** (ESI-TOF) *m/z* calculated for $C_{31}H_{29}N_2O_5$ [MH$^+$]: 509.2076 found: 509.2078; **R*f*** (DCM:acetone = 95:5): major: 0.43, minor: 0.62.

Dimethyl 1-(pyrrolidin-1-yl)anthracene-2,3-dicarboxylate (14)

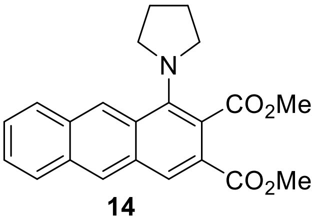

14

R*f* (PE:EA = 1:1): 0.72; **^{1}H NMR** (400 MHz, CDCl3) δ 8.61 (s, 1H), 8.57 (s, 1H), 8.54 (s, 1H), 8.03 (ddd, *J* = 7.3, 5.2, 2.1 Hz, 2H), 7.59–7.47 (m, 2H), 3.98 (s, 3H), 3.96 (s, 3H), 3.51–3.42 (m, 4H), 2.18–2.13 (m, 4H); **^{13}C NMR** (101 MHz, $CDCl_3$) δ 169.9, 166.2, 144.4, 133.0, 132.2, 131.6, 130.9, 130.7, 130.4, 129.9, 128.8, 128.3, 126.9, 126.4, 124.7, 123.5, 52.5, 52.5, 52.4, 26.6. **IR** (ν/cm^{-1}): 3049, 2948, 2847, 2363, 2255, 1718, 1438, 1364, 1326, 1285, 1248, 1196, 1159, 1095, 1051, 984, 946, 913, 790, 730; **HRMS** (ESI-TOF) *m/z* calculated for $C_{22}H_{22}NO_4$ [MH$^+$]: 364.1549 found: 364.1552.

Dimethyl (6*R*,12*S*)-12-bromo-9-oxo-9,10-dihydro-6*H*-6,10-methanocyclohepta-[*b*]naphthalene-7,8-dicarboxylate (15)

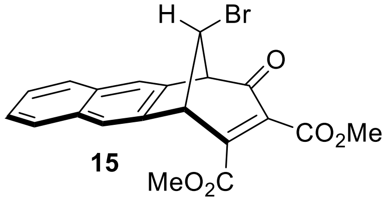

15

Dimethyl (1*R*,4*S*)-1-(pyrrolidin-1-yl)-1,4-dihydro-1,4-ethenoanthracene-2,3-dicarboxylate (**3eD**) (100 mg, 257 µmol, 1.0 equiv) was dissolved in H_2O/acetone (1:3, 4 mL) and cooled down to 0 °C. NBS (183 mg, 1.03 mmol, 4.0 equiv) was added at portions. The mixture was allowed to warm up to room temperature and was stirred for 16 h. Then, saturated aqueous solution of $Na_2S_2O_5$ was added. The aqueous layer was extracted with Et_2O and the combined organic phases were washed with water and dried over anhydrous $NaSO_4$. The solvent was removed under reduced pressure and the crude product was purrified by coloumn chromatography (PE:EA = 95:5) and **15** was obtained as a yellow solid (81 mg, 194 µmol, 76%).

Mp.: 180–182 °C; $\mathbf{R}_f$ (PE:EA = 1:1): 0.56; **^{1}H NMR** (400 MHz, $CDCl_3$) *δ* 7.82 (s, 1H), 7.80 (s, 1H), 7.80–7.75 (m, 2H), 7.54–7.47 (m, 2H), 5.04 (t, *J* = 4.5 Hz, 1H), 4.67 (dd, *J* = 4.6, 1.1 Hz, 1H), 4.29 (dd, *J* = 4.5, 1.1 Hz, 1H), 3.90 (s, 3H), 3.80 (s, 3H); **^{13}C NMR** (101 MHz, $CDCl_3$) *δ* 189.9, 164.4, 164.2, 142.6, 140.2, 133.4, 133.3, 133.2, 132.7, 128.3, 128.1, 127.3, 127.0, 125.4, 123.2, 61.8, 54.4, 53.5, 52.7, 48.0; **IR** (ν/cm^{-1}): 3004, 2952, 2922, 2851, 2363, 2258, 1722, 1625, 1505, 1435, 1341, 1263, 1166, 1043, 961, 909, 797, 726; **HRMS** (ESI-TOF) *m/z* calculated for $C_{20}H_{15}BrO_5$ [MH^+]: 415.0181 found: 415.0184.

5. NMR Spectra

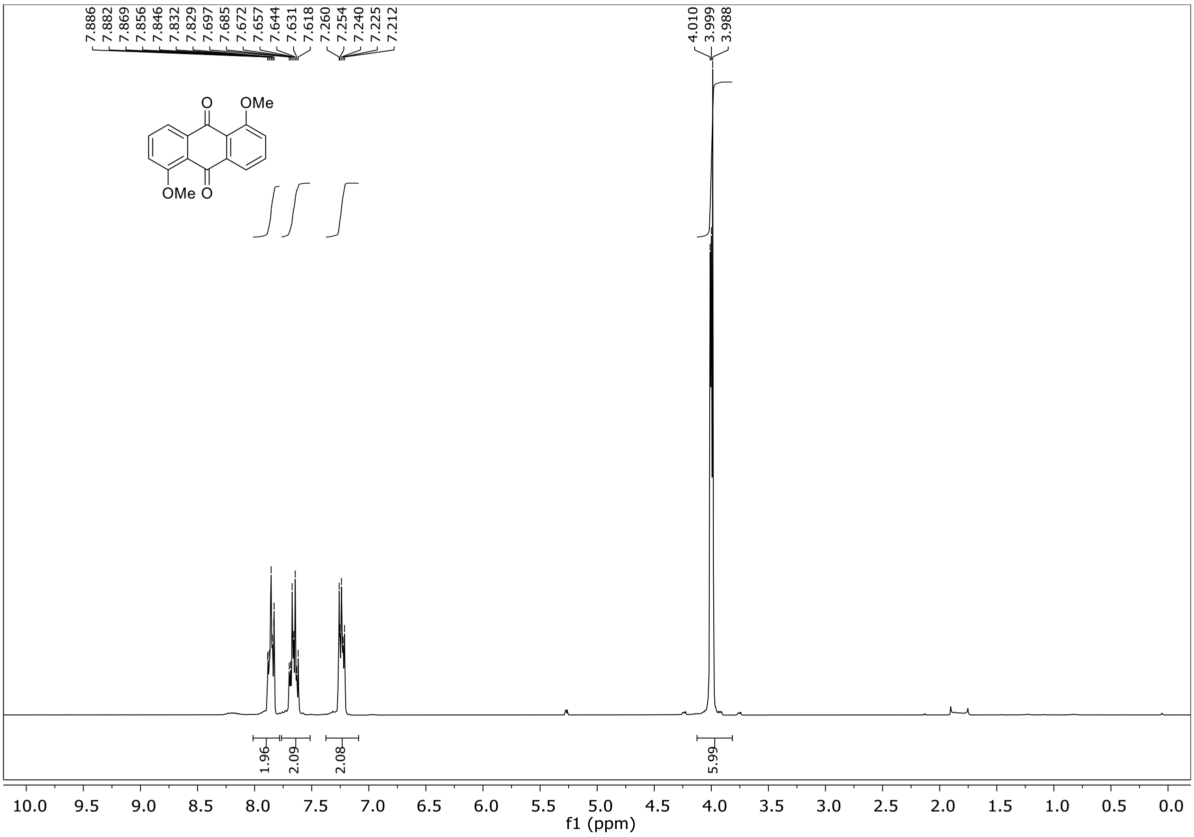

Supplementary Figure 1. ^{1}H NMR of 1,5-dihydroxy-9,10-anthraquinone (300 MHz, $CDCl_3$)

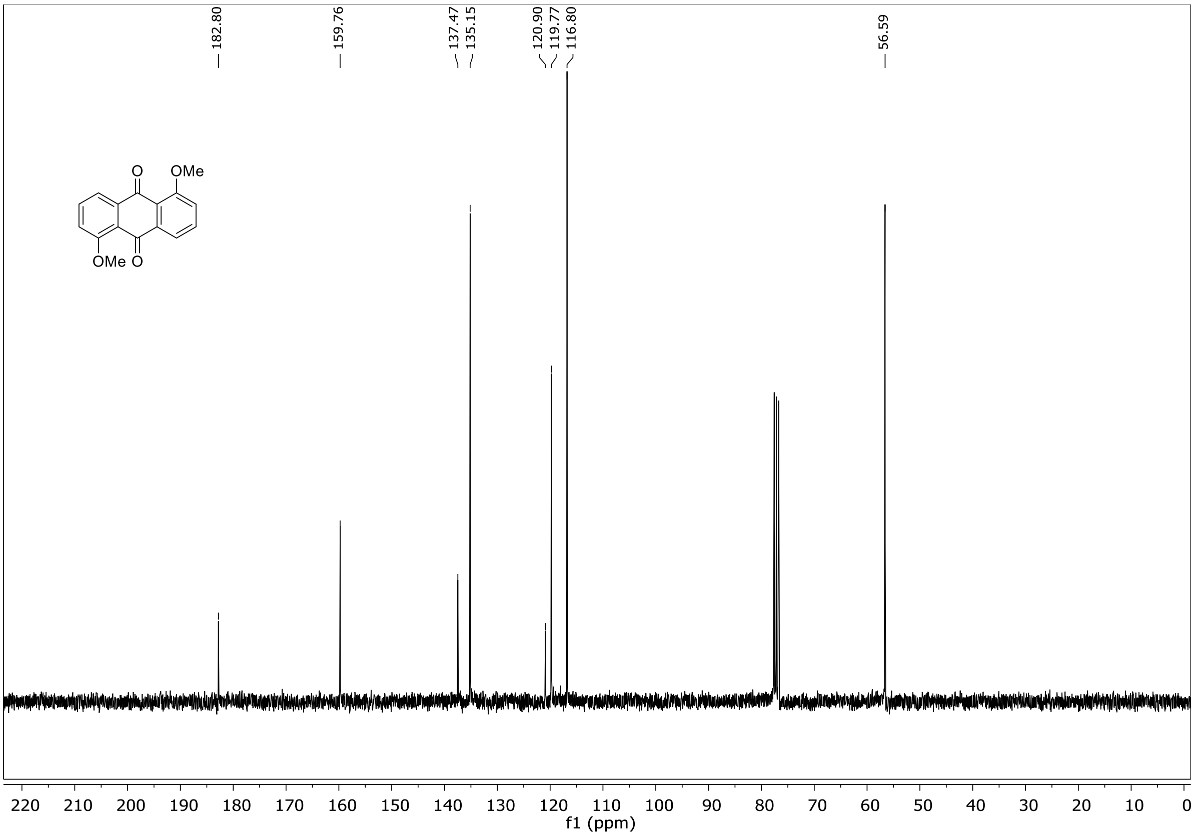

Supplementary Figure 2. ^{13}C NMR of 1,5-dihydroxy-9,10-anthraquinone (75 MHz, $CDCl_3$)

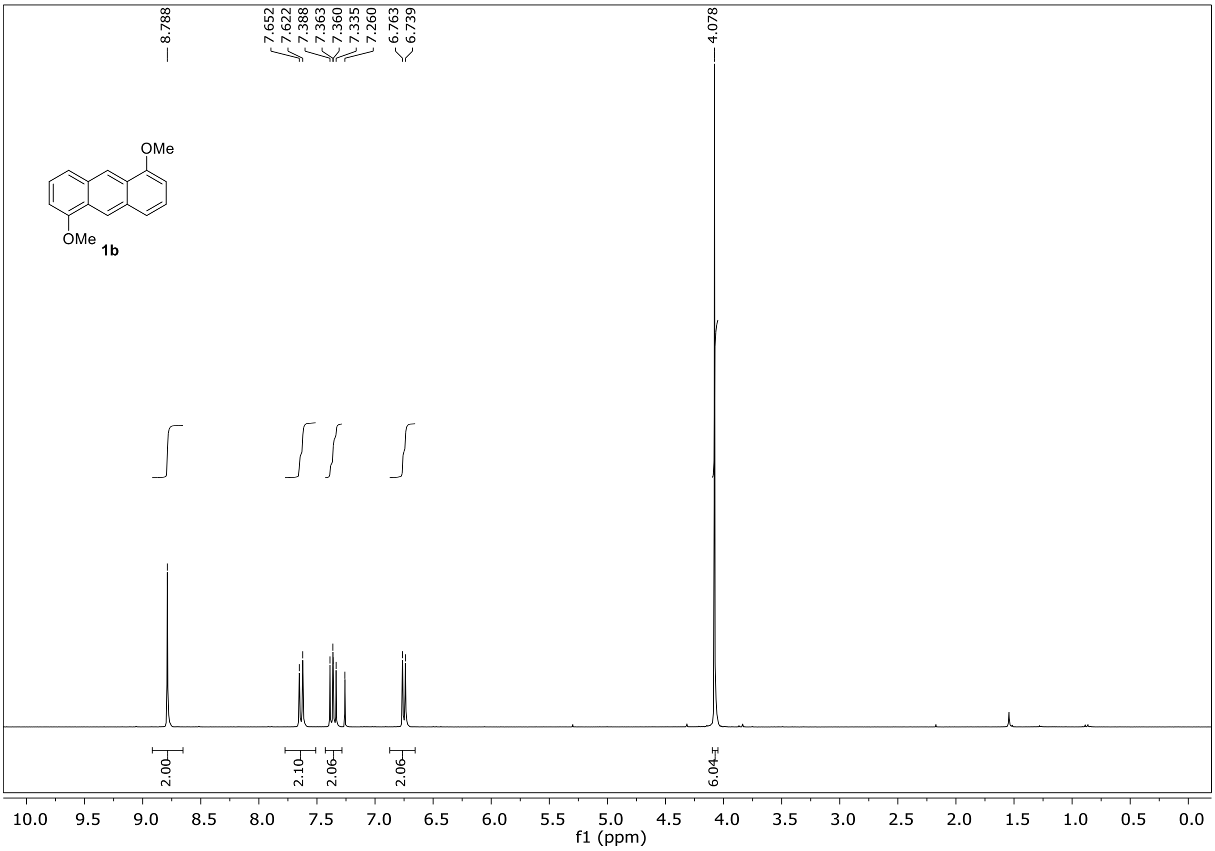

Supplementary Figure 3. ^{1}H NMR of **1b** (300 MHz, $CDCl_3$)

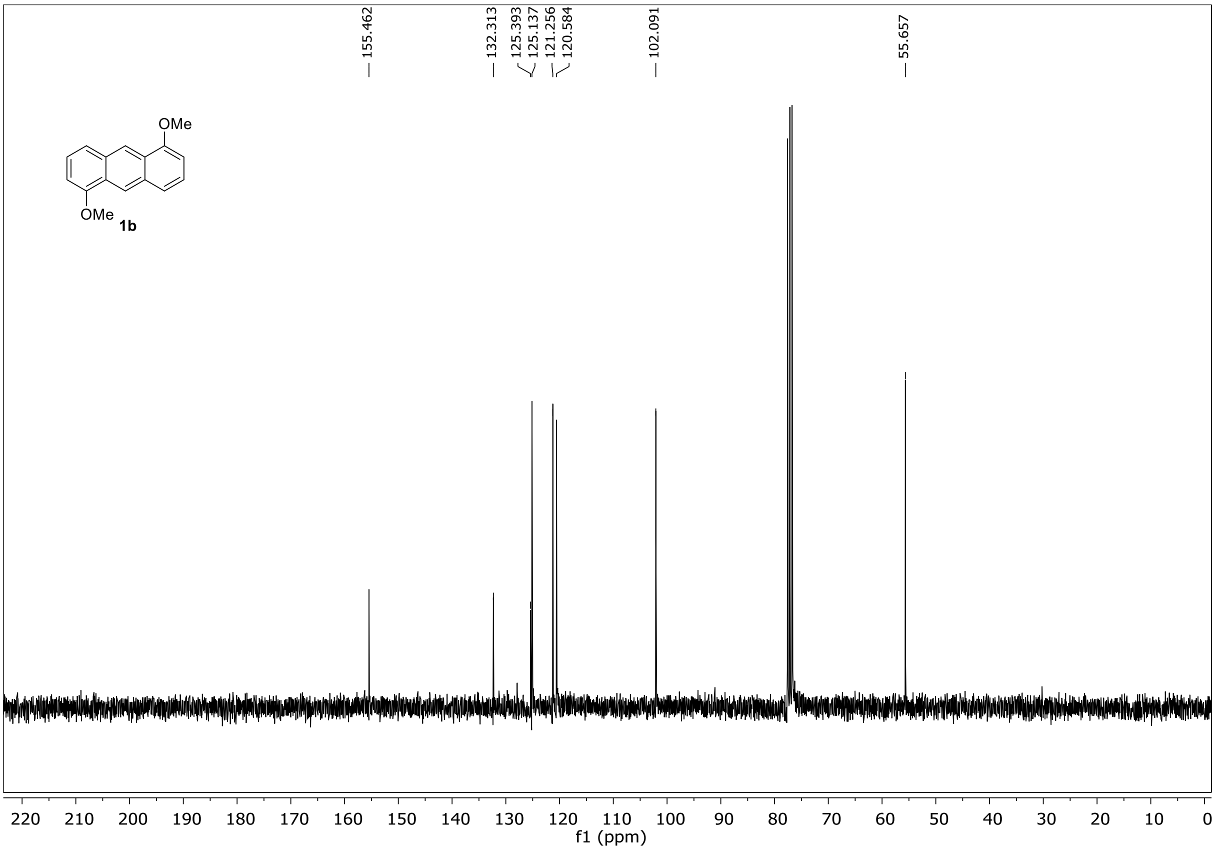

Supplementary Figure 4. ^{13}C NMR of **1b** (75 MHz, $CDCl_3$)

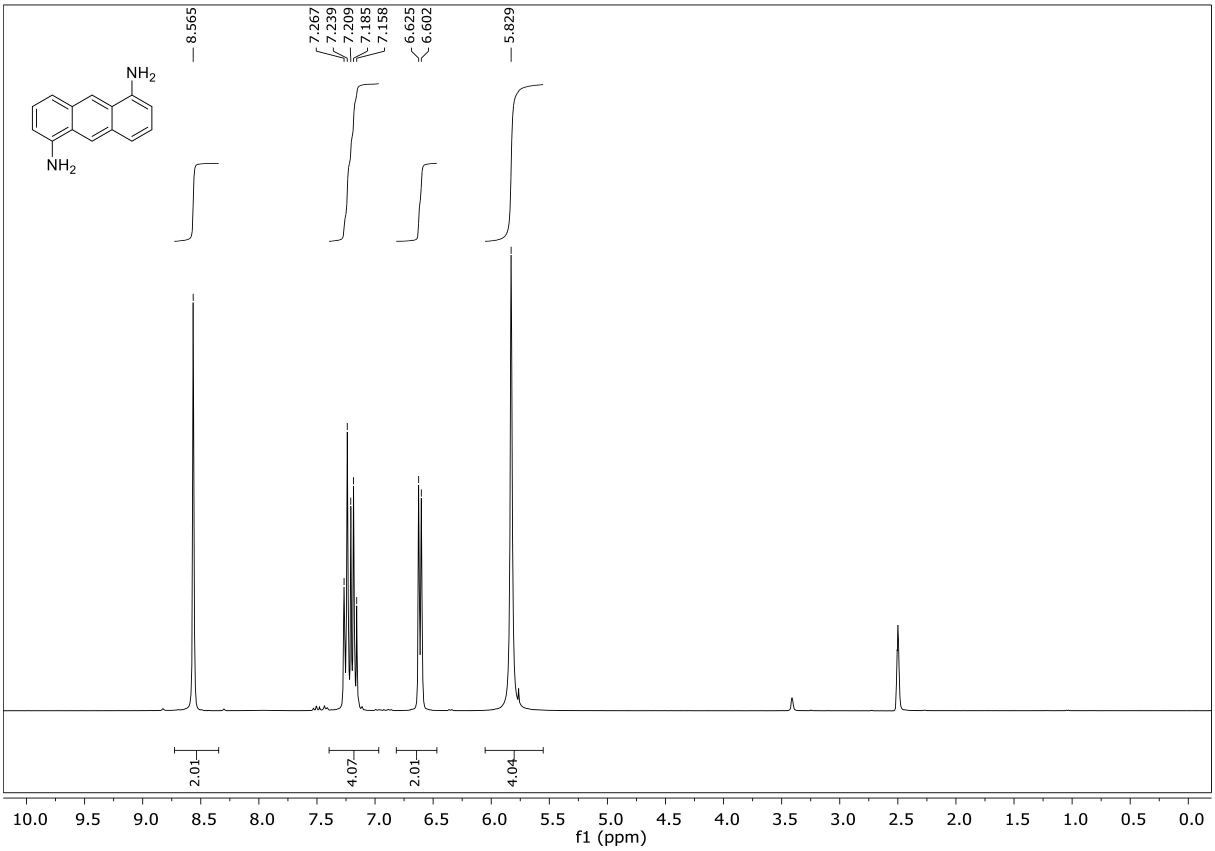

Supplementary Figure 5. ^{1}H NMR of 1,5-diaminoanthracene (300 MHz, $CDCl_3$)

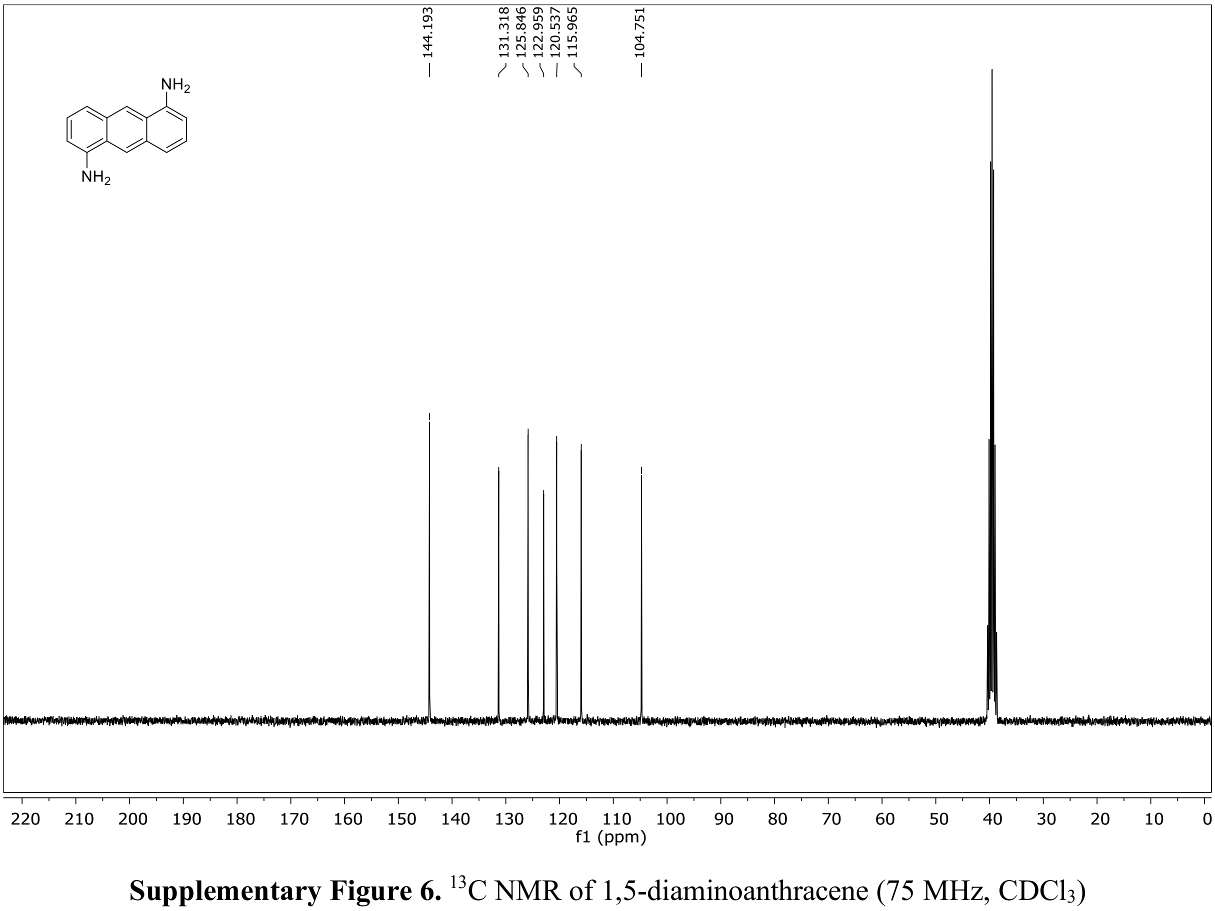

Supplementary Figure 6. ^{13}C NMR of 1,5-diaminoanthracene (75 MHz, $CDCl_3$)

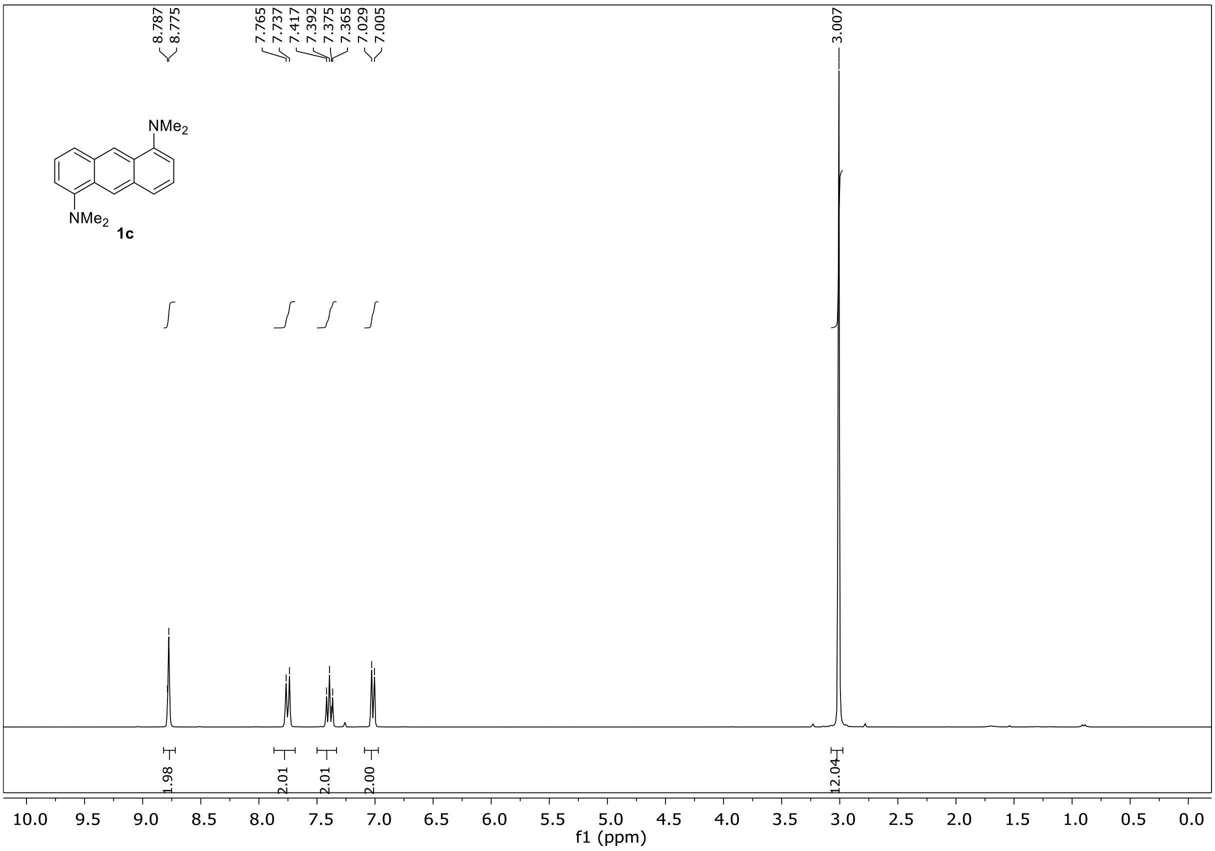

Supplementary Figure 7. ^{1}H NMR of **1c** (300 MHz, $CDCl_3$)

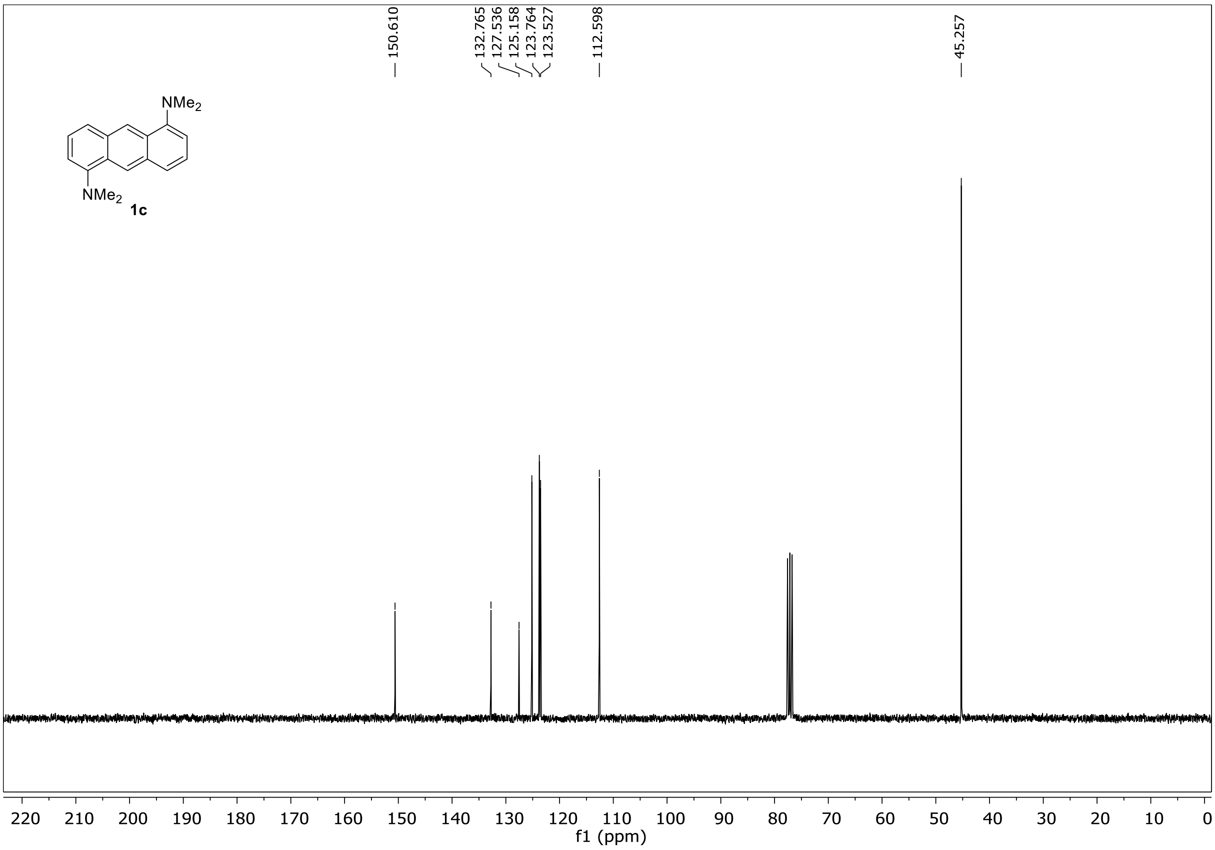

Supplementary Figure 8. ^{13}C NMR of **1c** (75 MHz, $CDCl_3$)

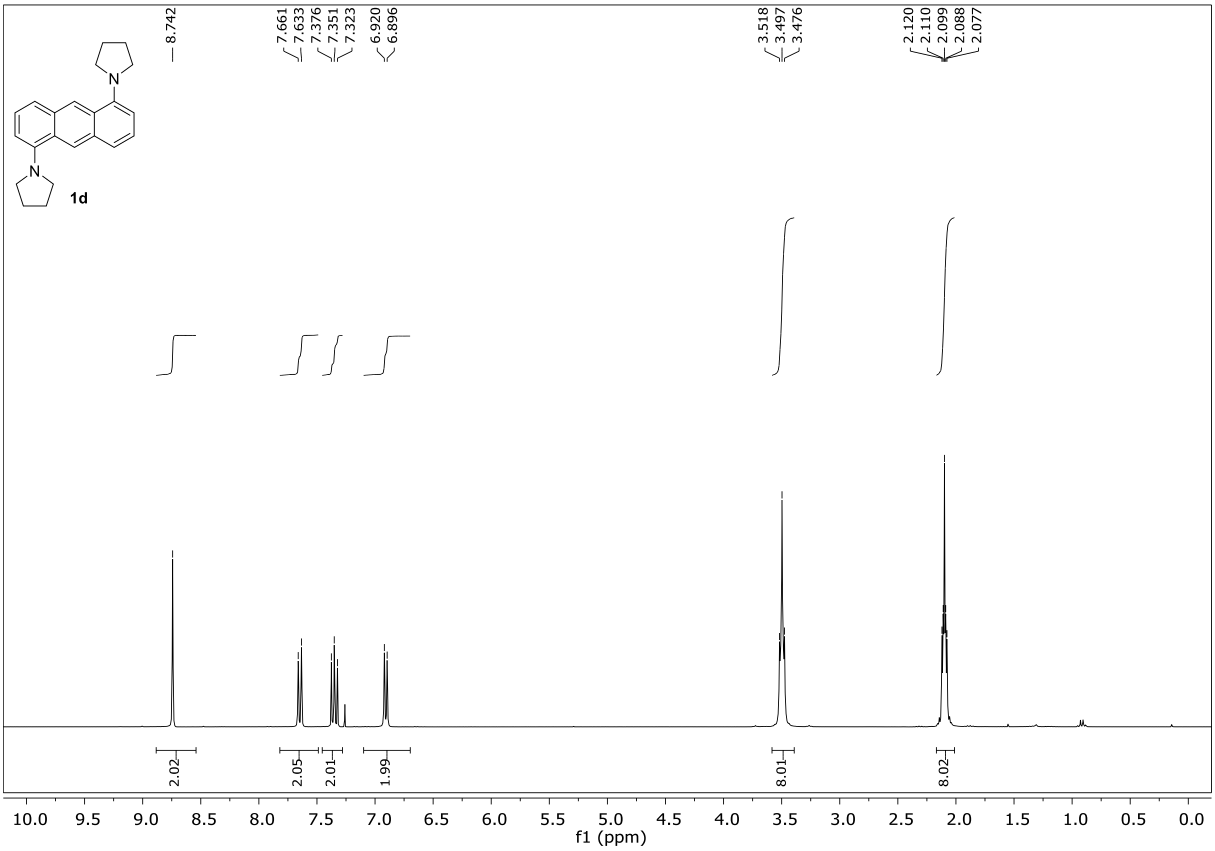

Supplementary Figure 9. ^{1}H NMR of **1d** (300 MHz, $CDCl_3$)

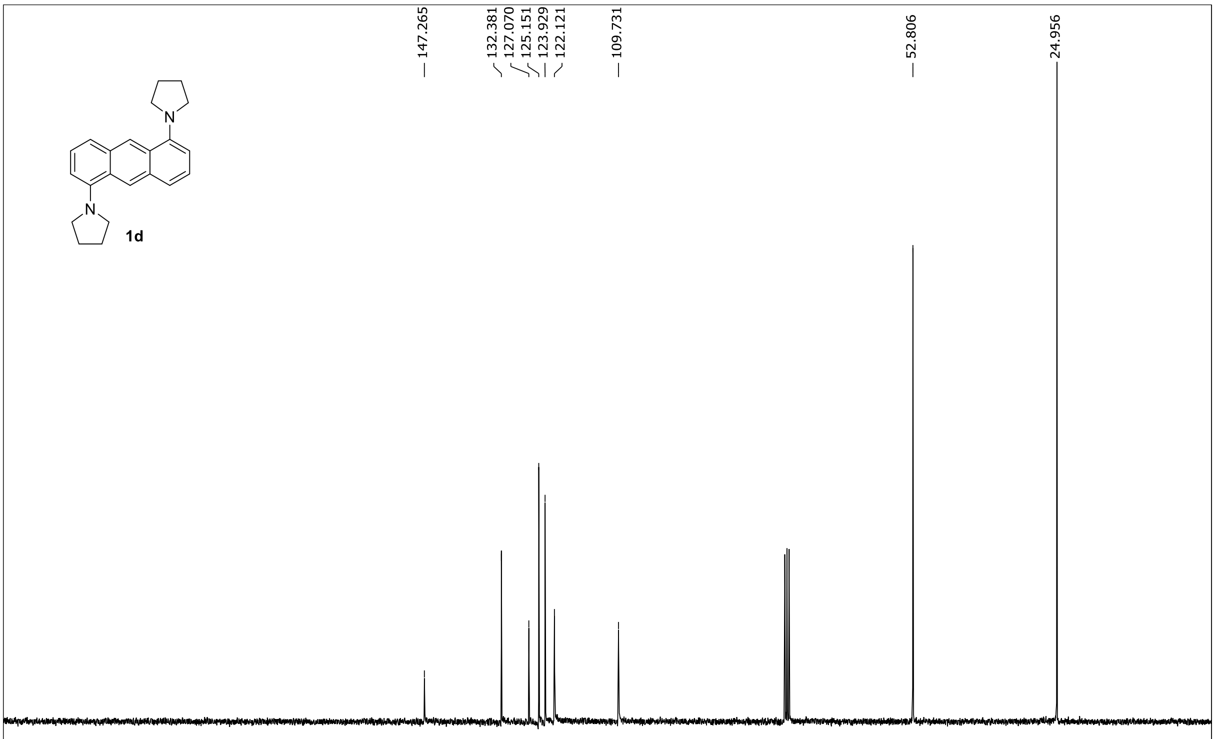

Supplementary Figure 10. ^{13}C NMR of **1d** (75 MHz, $CDCl_3$)

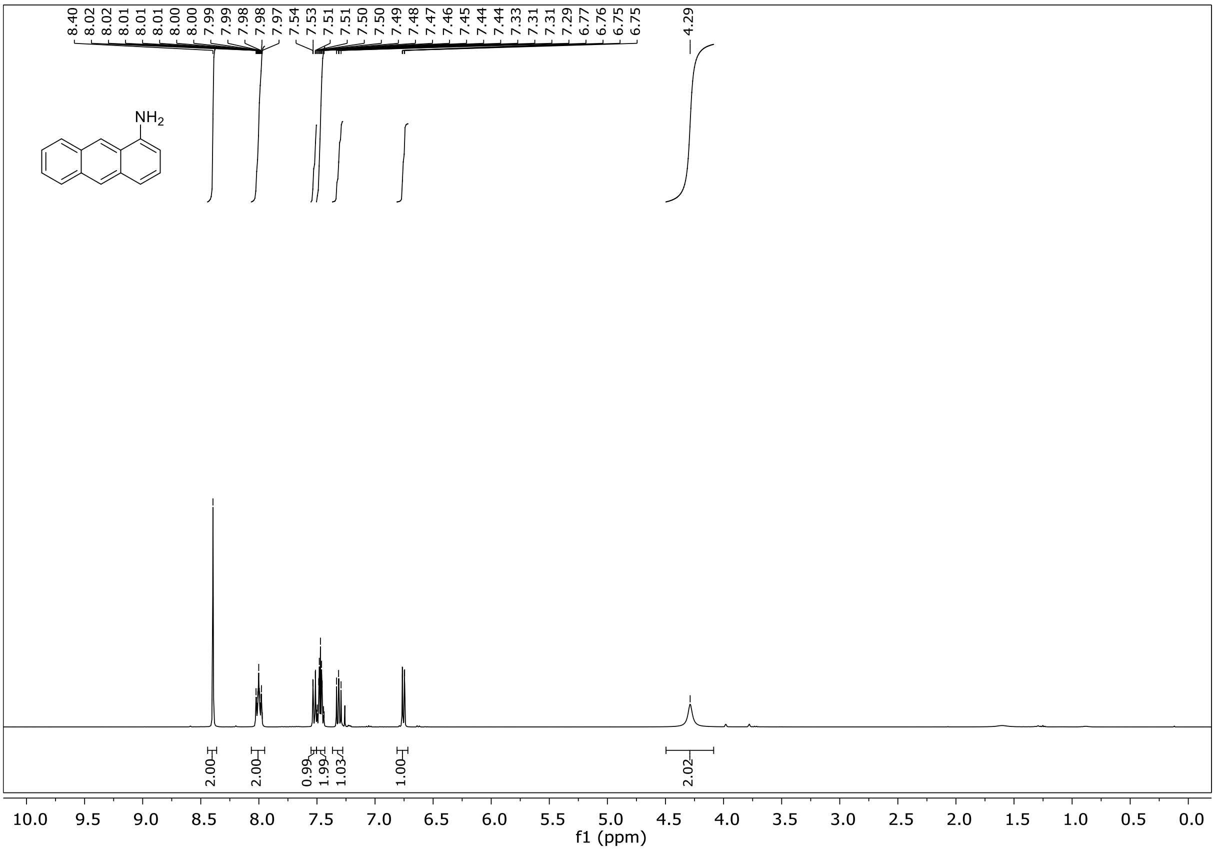

Supplementary Figure 11. ^{1}H NMR of 1-aminoanthracene (300 MHz, $CDCl_3$)

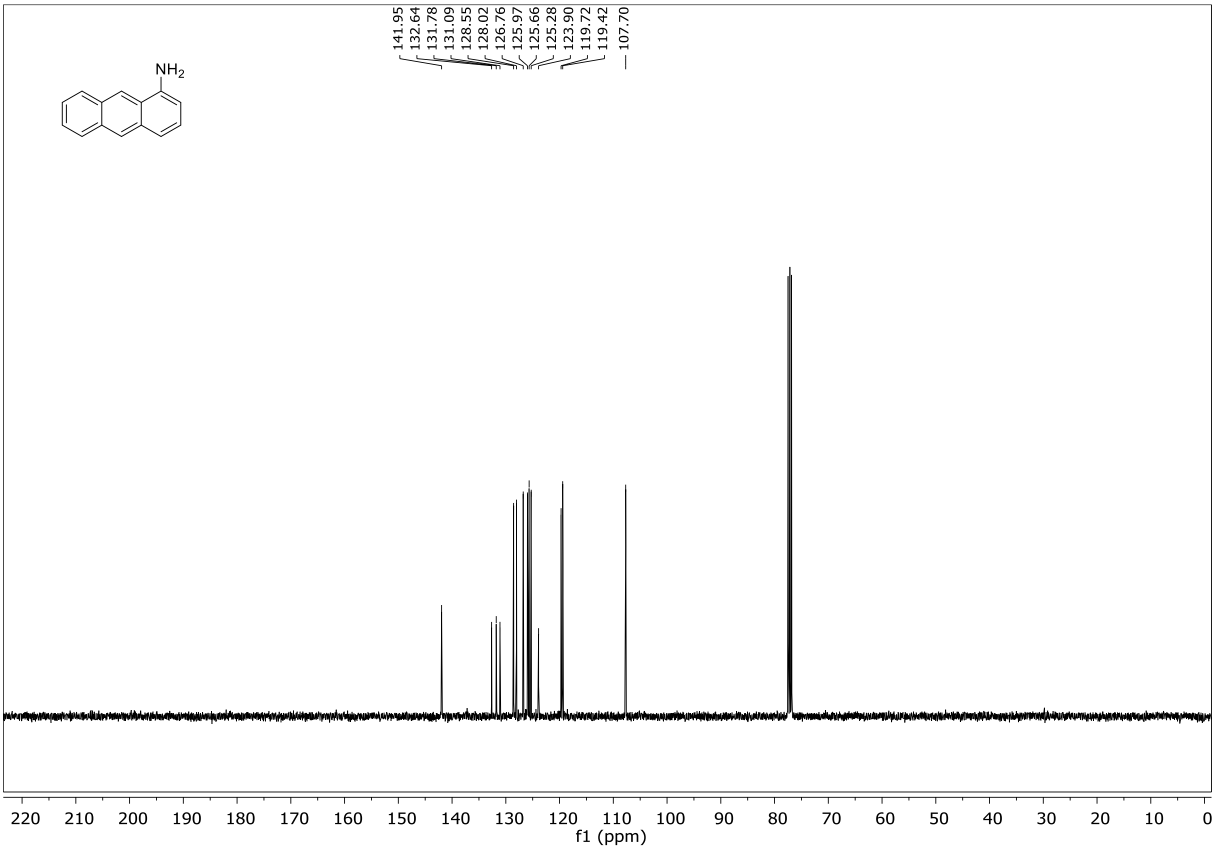

Supplementary Figure 12. ^{13}C NMR of 1-aminoanthracene (75 MHz, $CDCl_3$)

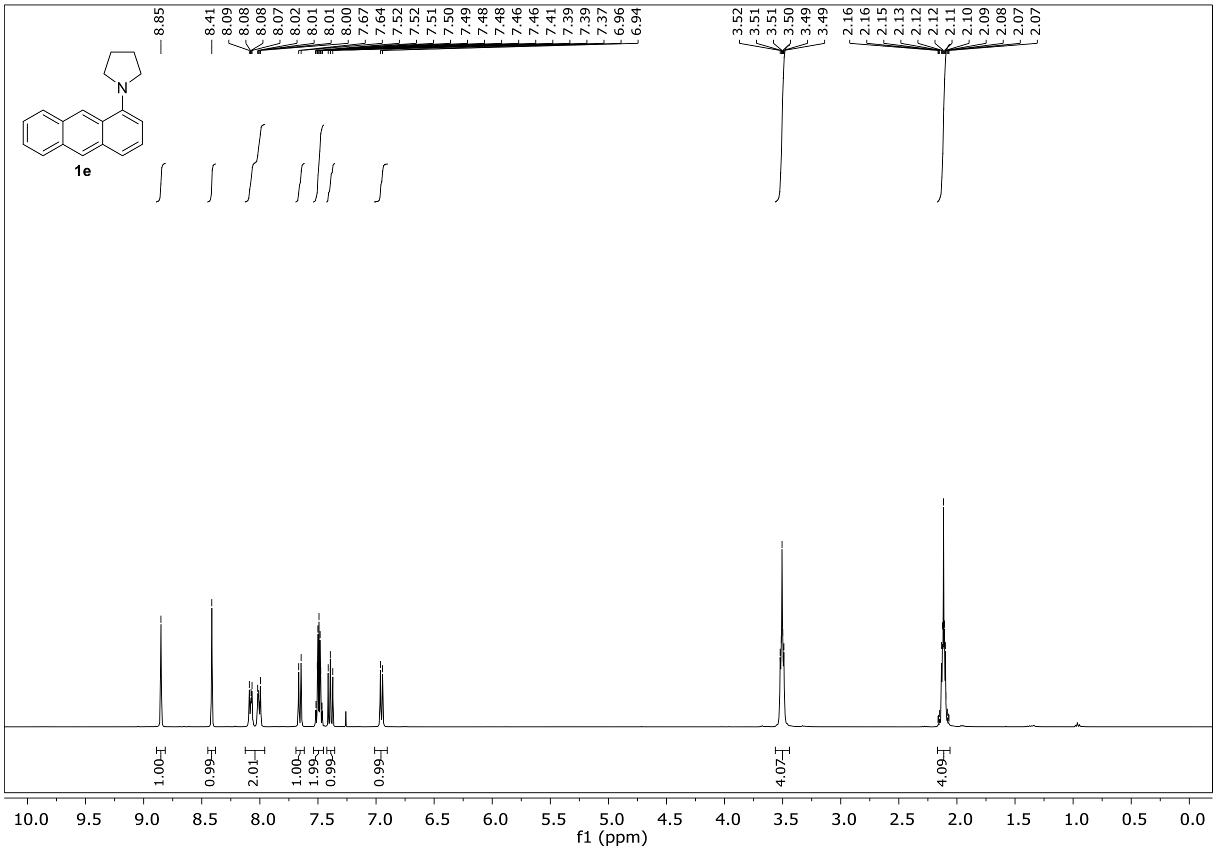

Supplementary Figure 13. ^{1}H NMR of **1e** (400 MHz, $CDCl_3$)

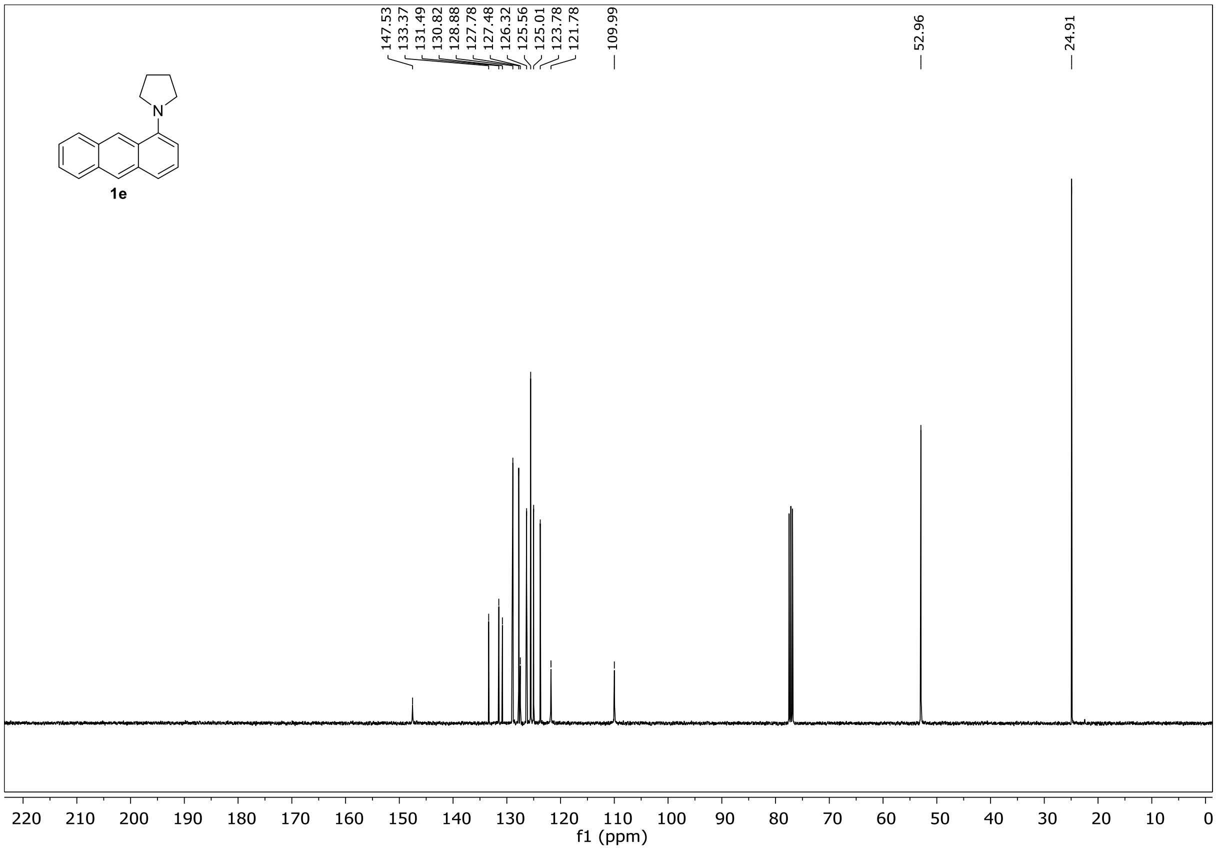

Supplementary Figure 14. ^{13}C NMR of **1e** (101 MHz, $CDCl_3$)

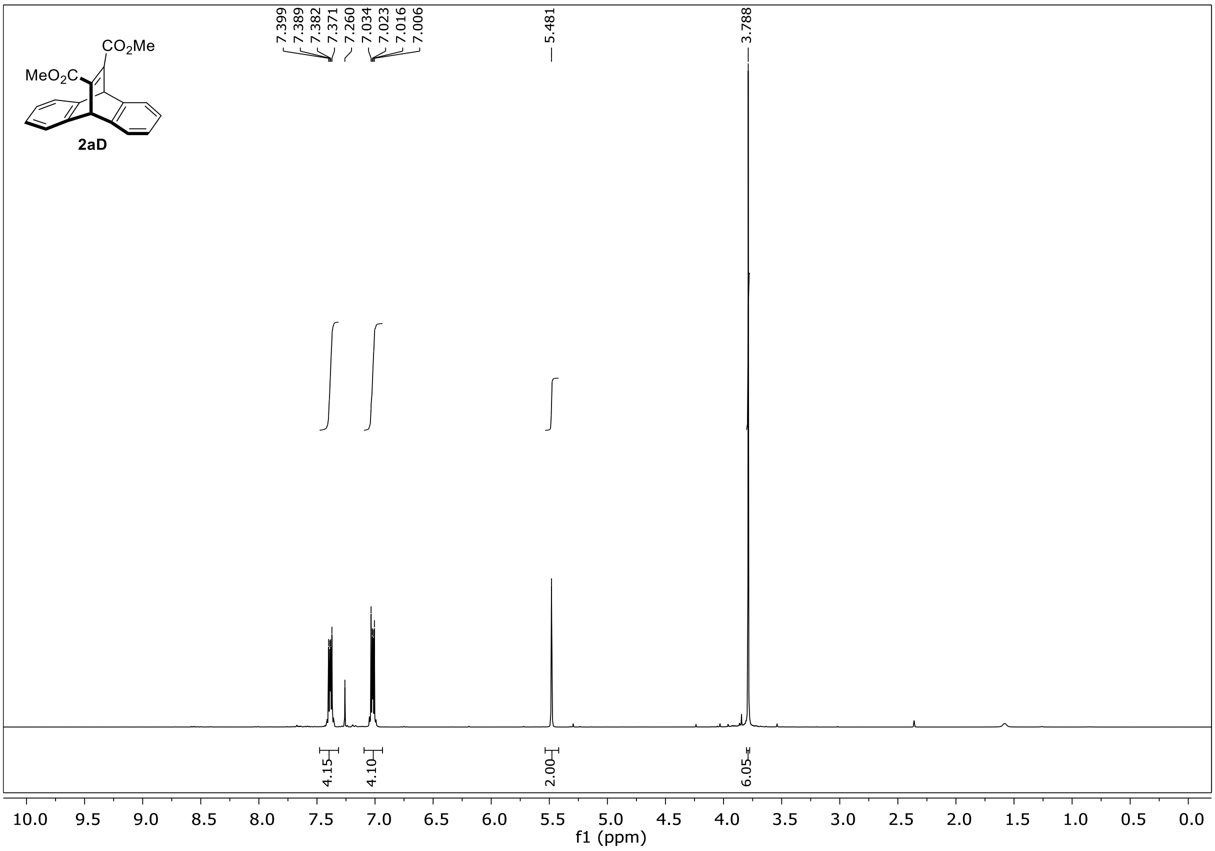

Supplementary Figure 15. ^{1}H NMR of **2aD** (300 MHz, $CDCl_3$)

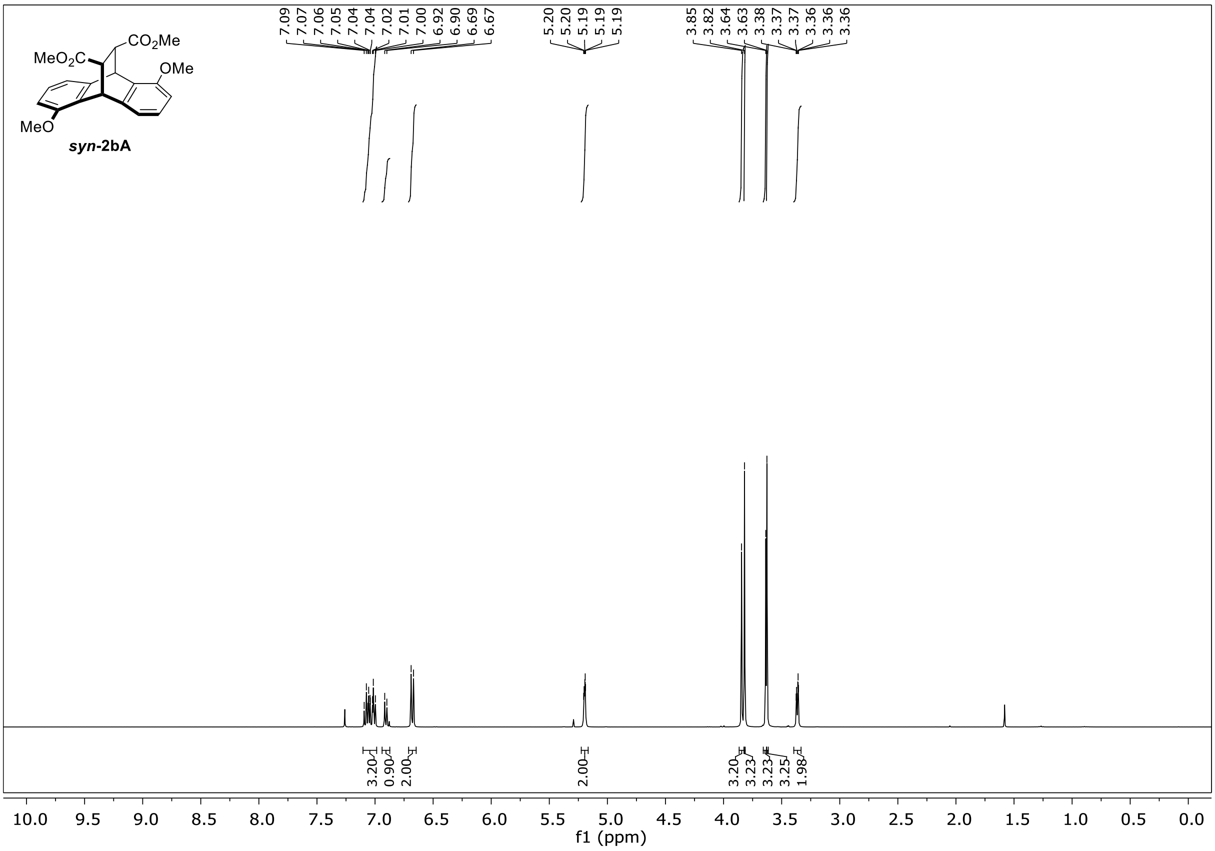

Supplementary Figure 16. ^{1}H NMR of ***syn*-2bA** (400 MHz, $CDCl_3$)

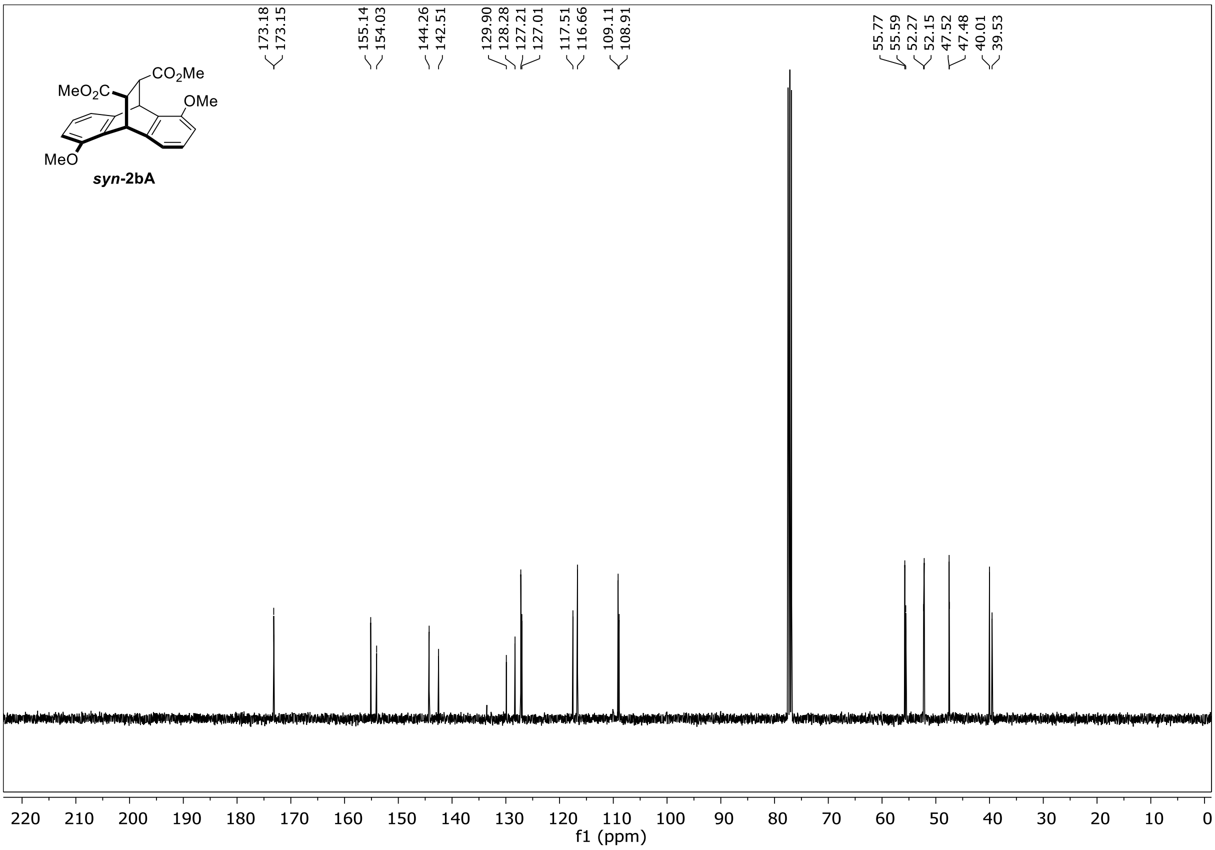

Supplementary Figure 17. ^{13}C NMR of ***syn*-2bA** (101 MHz, $CDCl_3$)

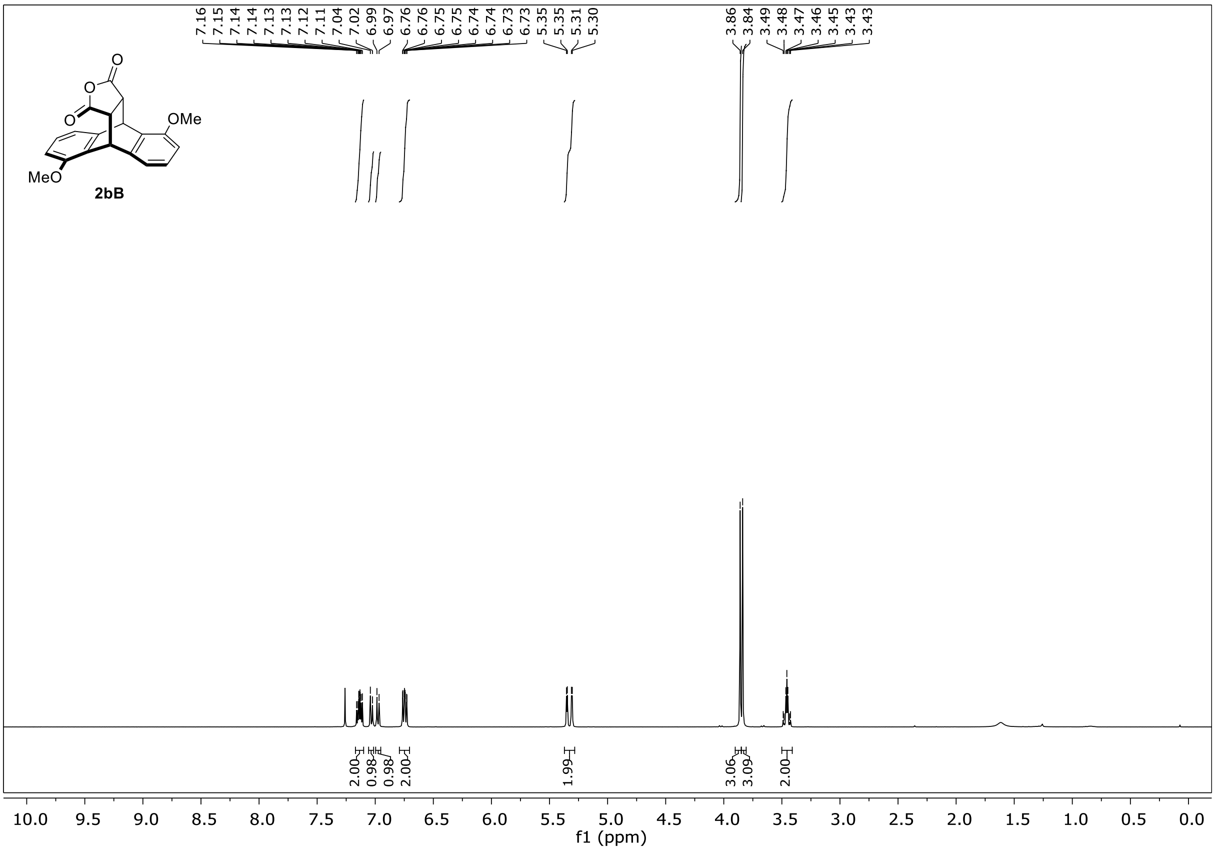

Supplementary Figure 18. ^{1}H NMR of **2bB** (400 MHz, $CDCl_3$)

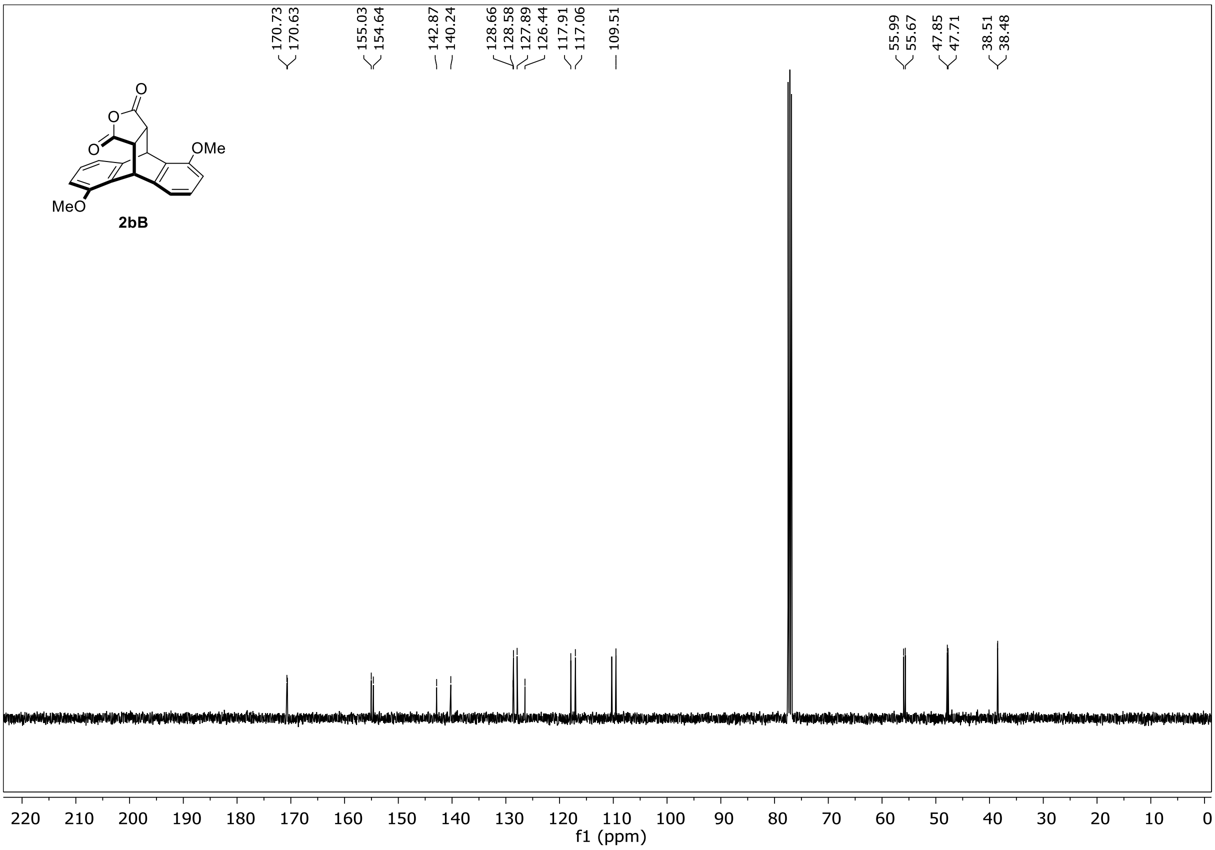

Supplementary Figure 19. ^{13}C NMR of **2bB** (101 MHz, $CDCl_3$)

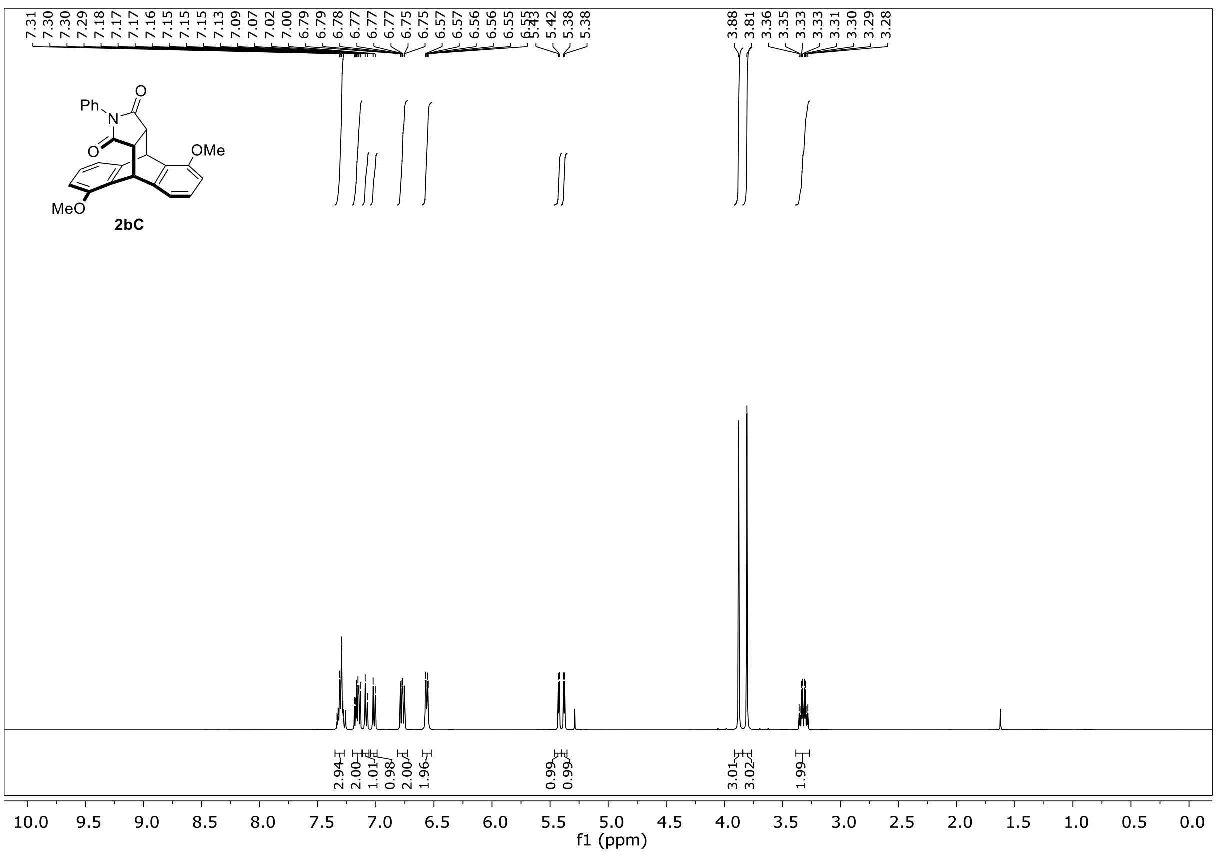

Supplementary Figure 20. ^{1}H NMR of **2bC** (400 MHz, $CDCl_3$)

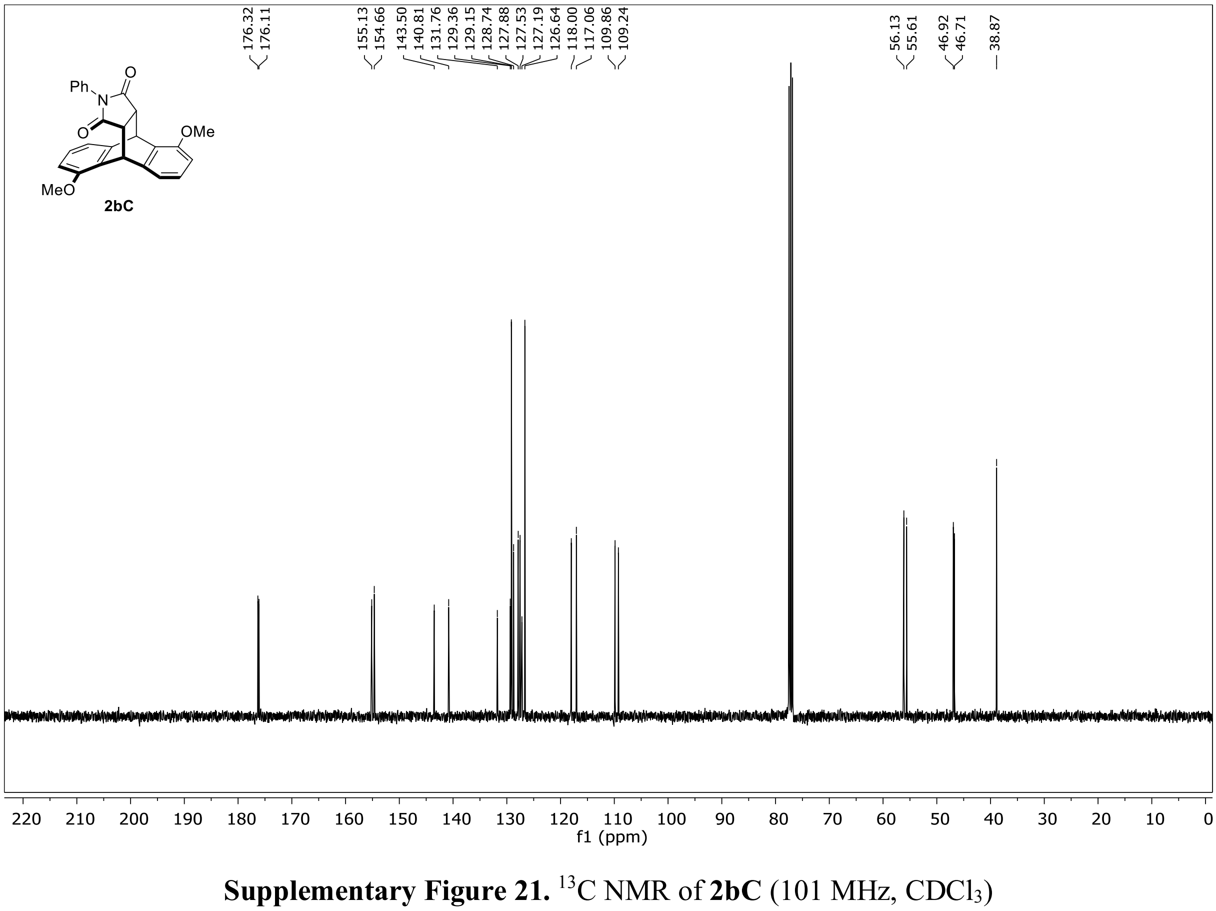

Supplementary Figure 21. ^{13}C NMR of **2bC** (101 MHz, $CDCl_3$)

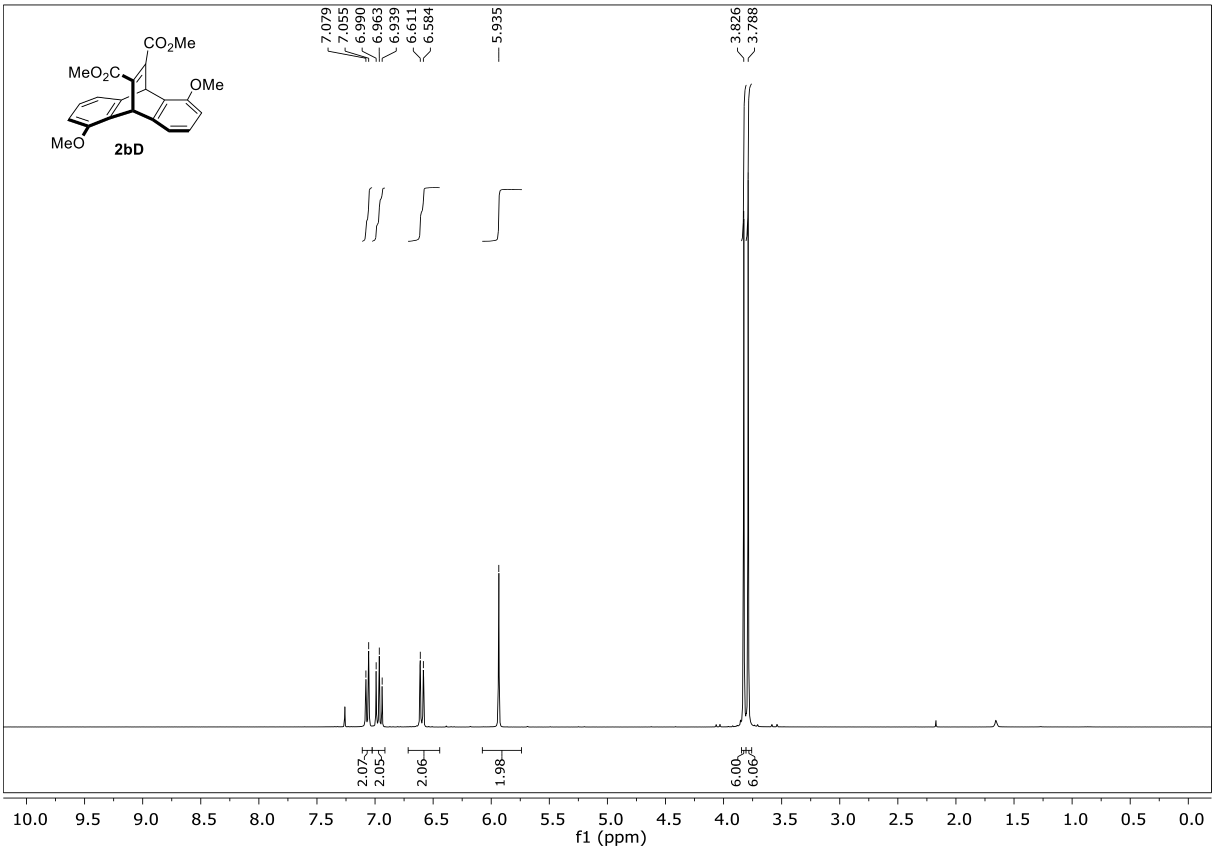

Supplementary Figure 22. ^{1}H NMR of **2bD** (300 MHz, $CDCl_3$)

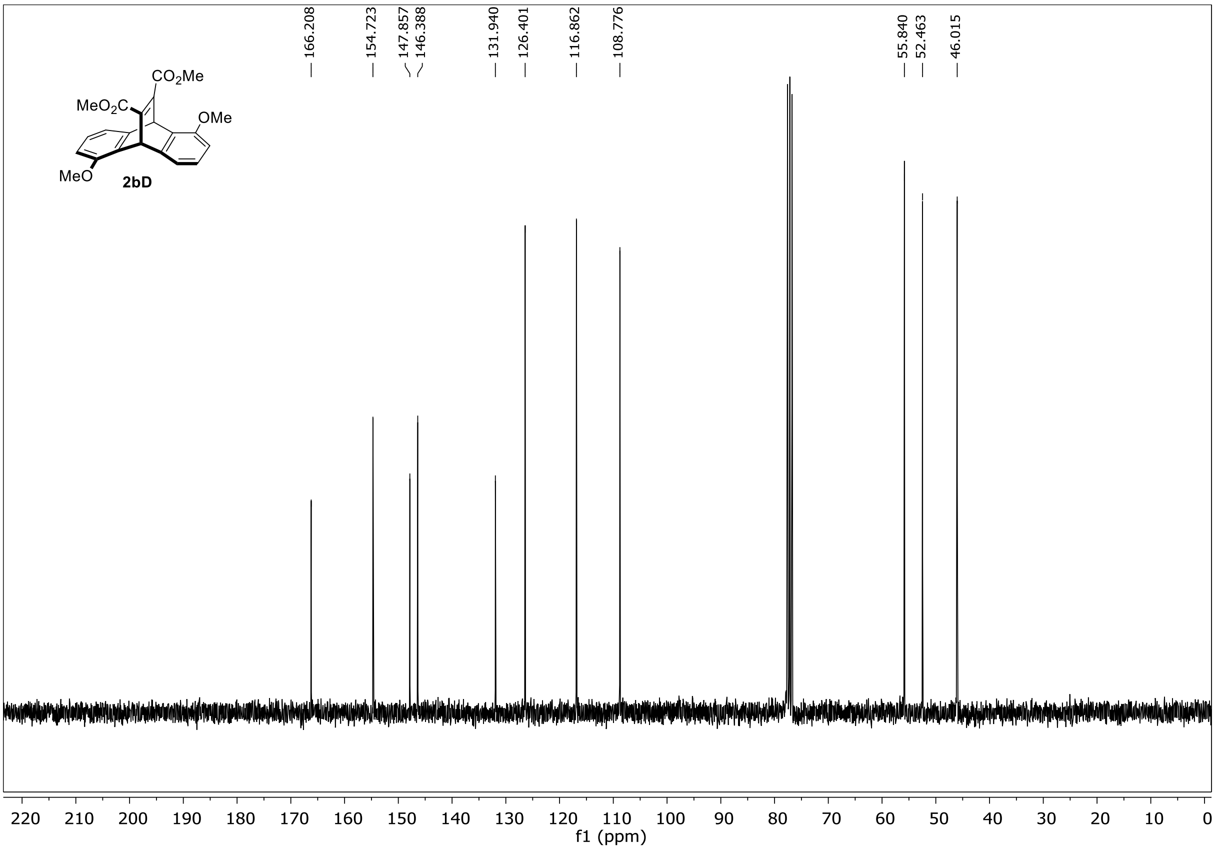

Supplementary Figure 23. ^{13}C NMR of **2bD** (75 MHz, $CDCl_3$)

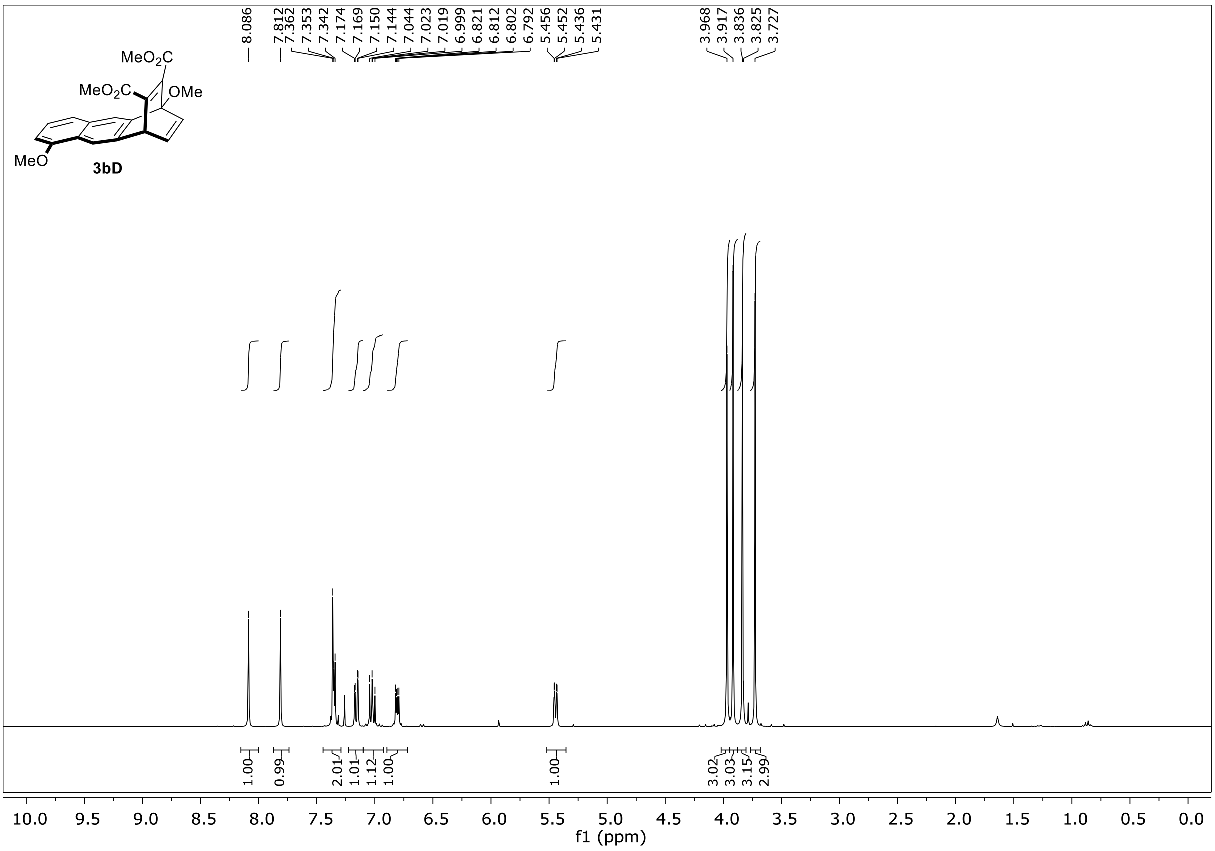

Supplementary Figure 24. ^{1}H NMR of **3bD** (300 MHz, $CDCl_3$)

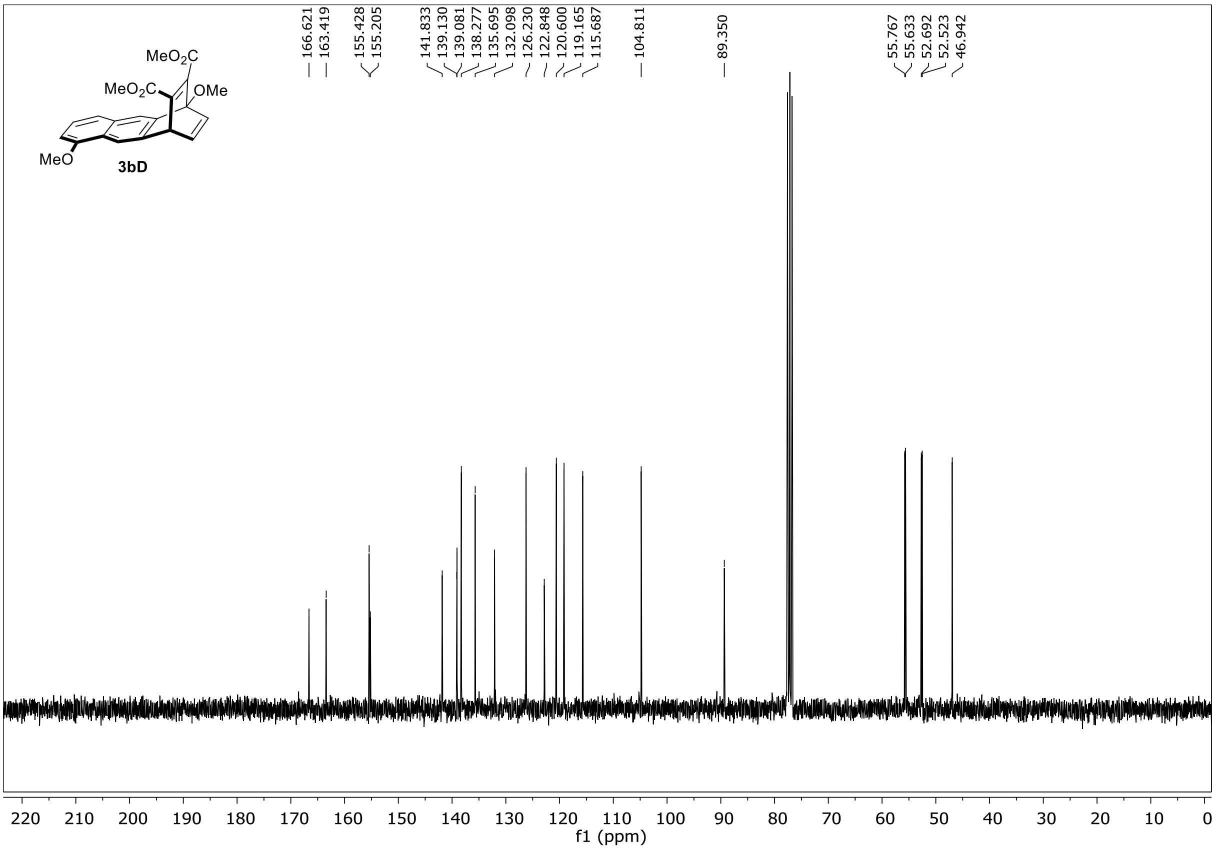

Supplementary Figure 25. ^{13}C NMR of **3bD** (75 MHz, $CDCl_3$)

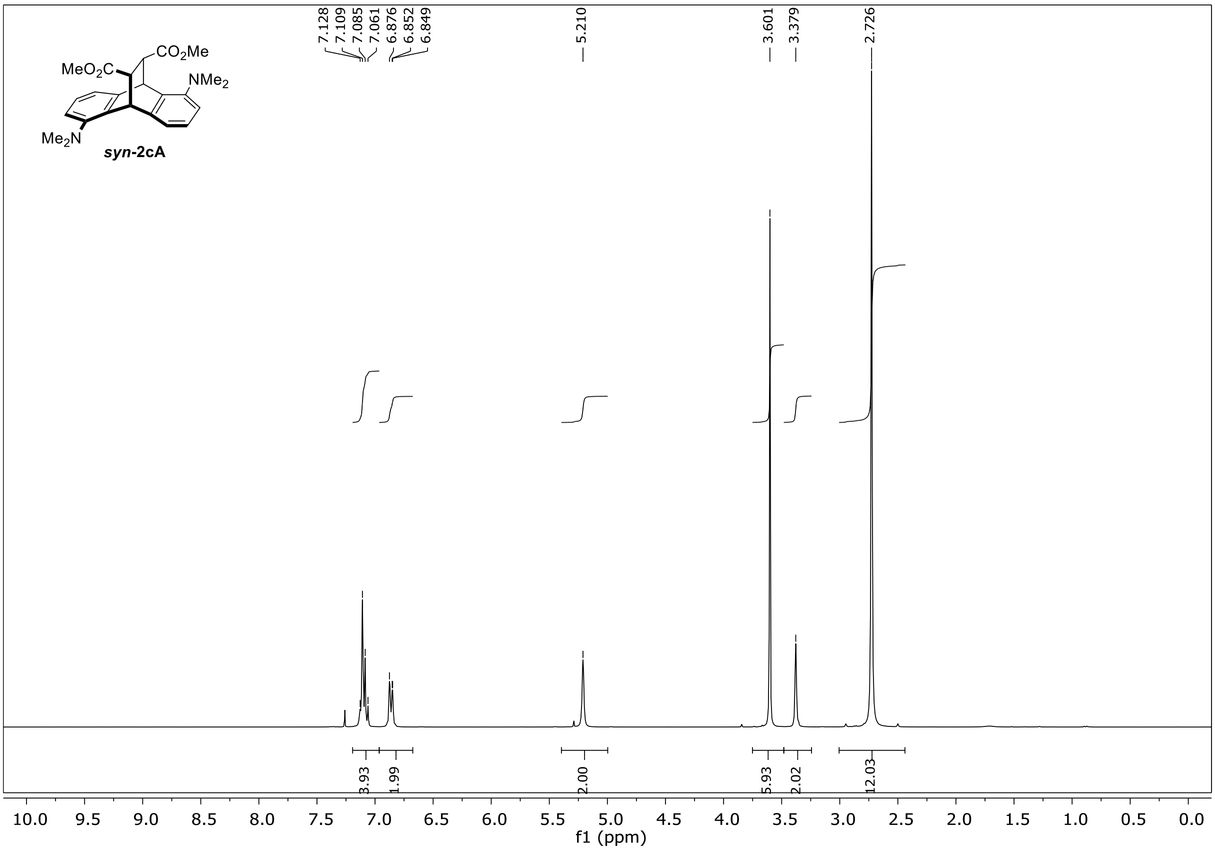

Supplementary Figure 26. ^{1}H NMR of ***syn*-2cA** (300 MHz, $CDCl_3$)

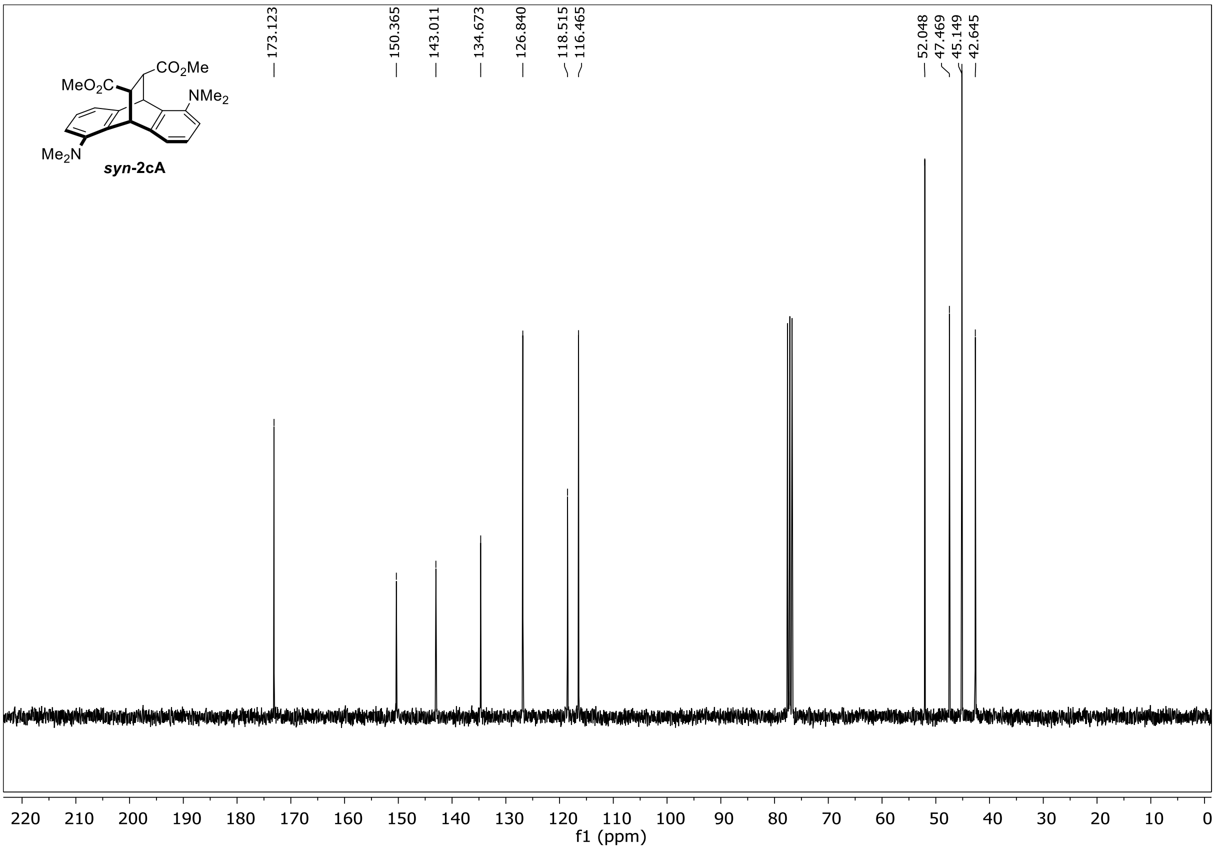

Supplementary Figure 27. ^{13}C NMR of ***syn*-2cA** (75 MHz, $CDCl_3$)

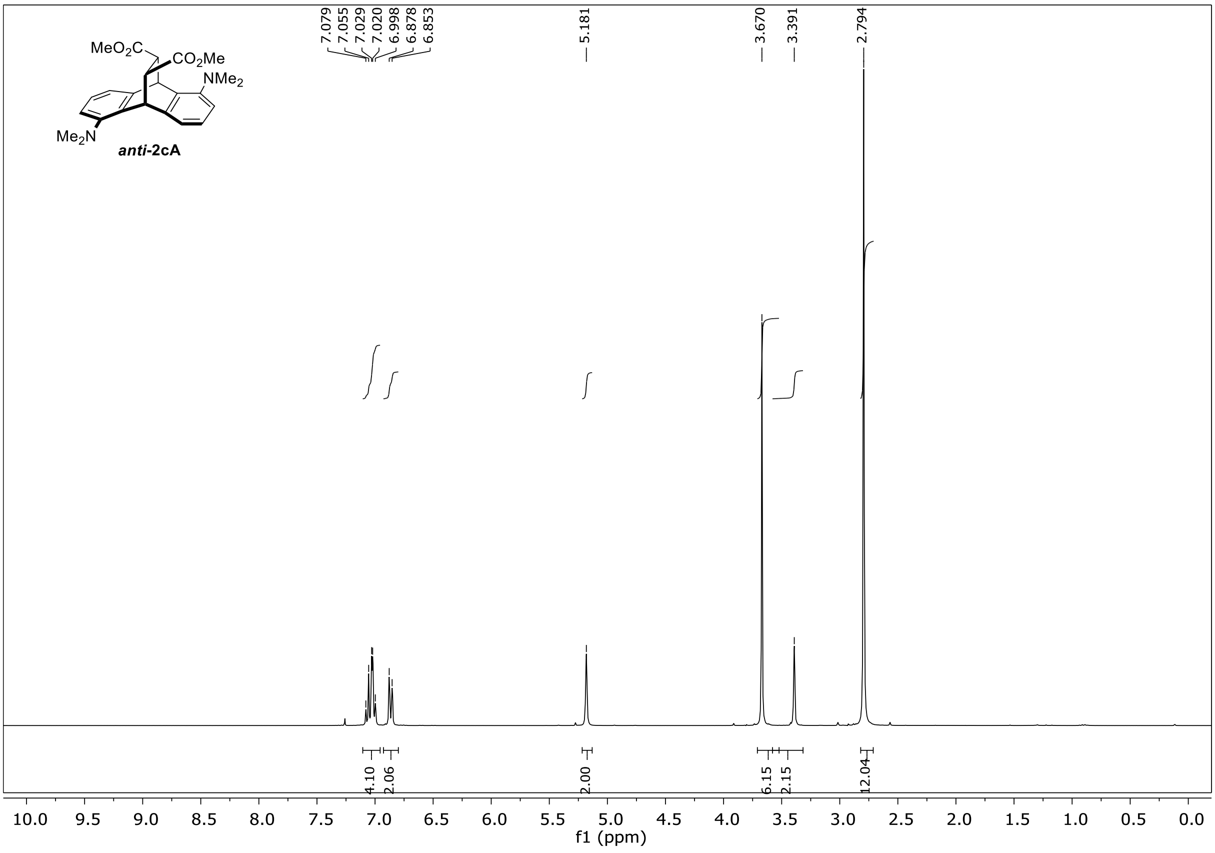

Supplementary Figure 28. ^{1}H NMR of ***anti*-2cA** (300 MHz, $CDCl_3$)

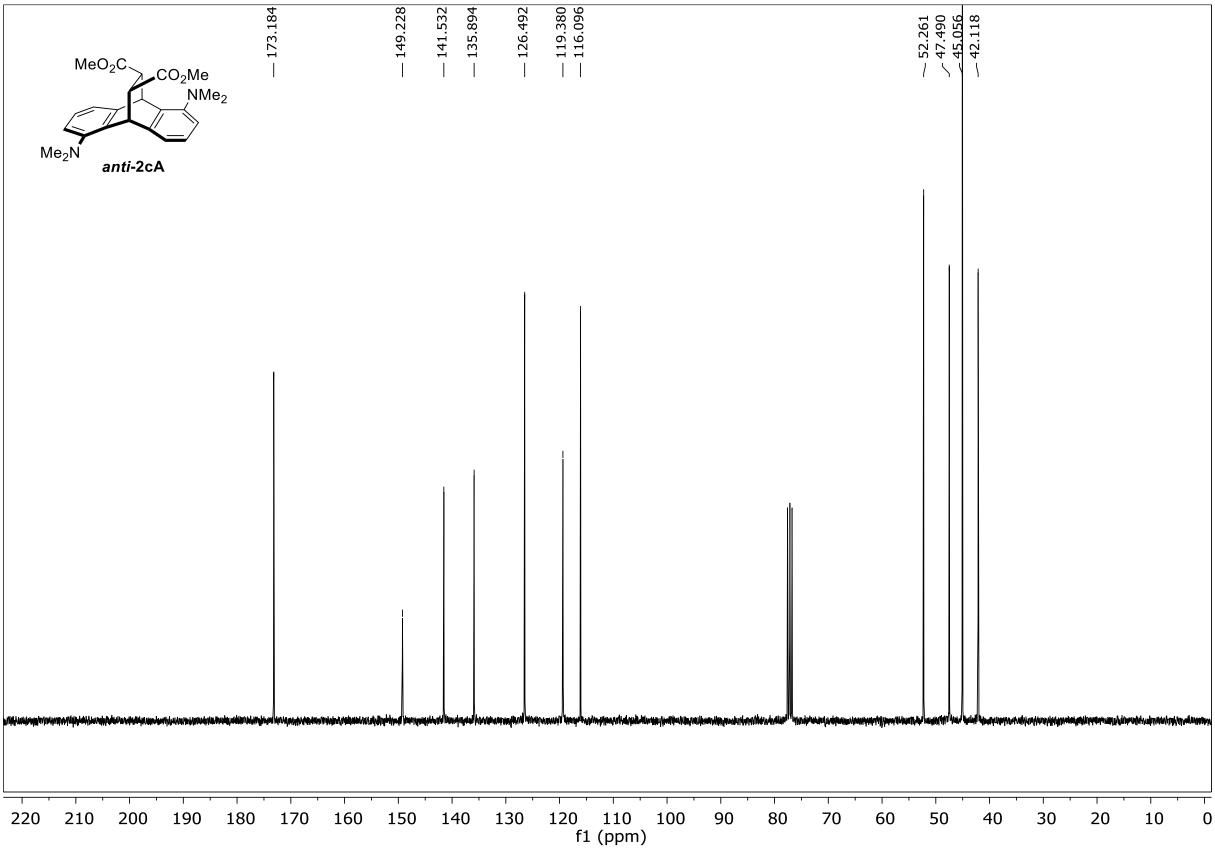

Supplementary Figure 29. ^{13}C NMR of ***anti*-2cA** (75 MHz, $CDCl_3$)

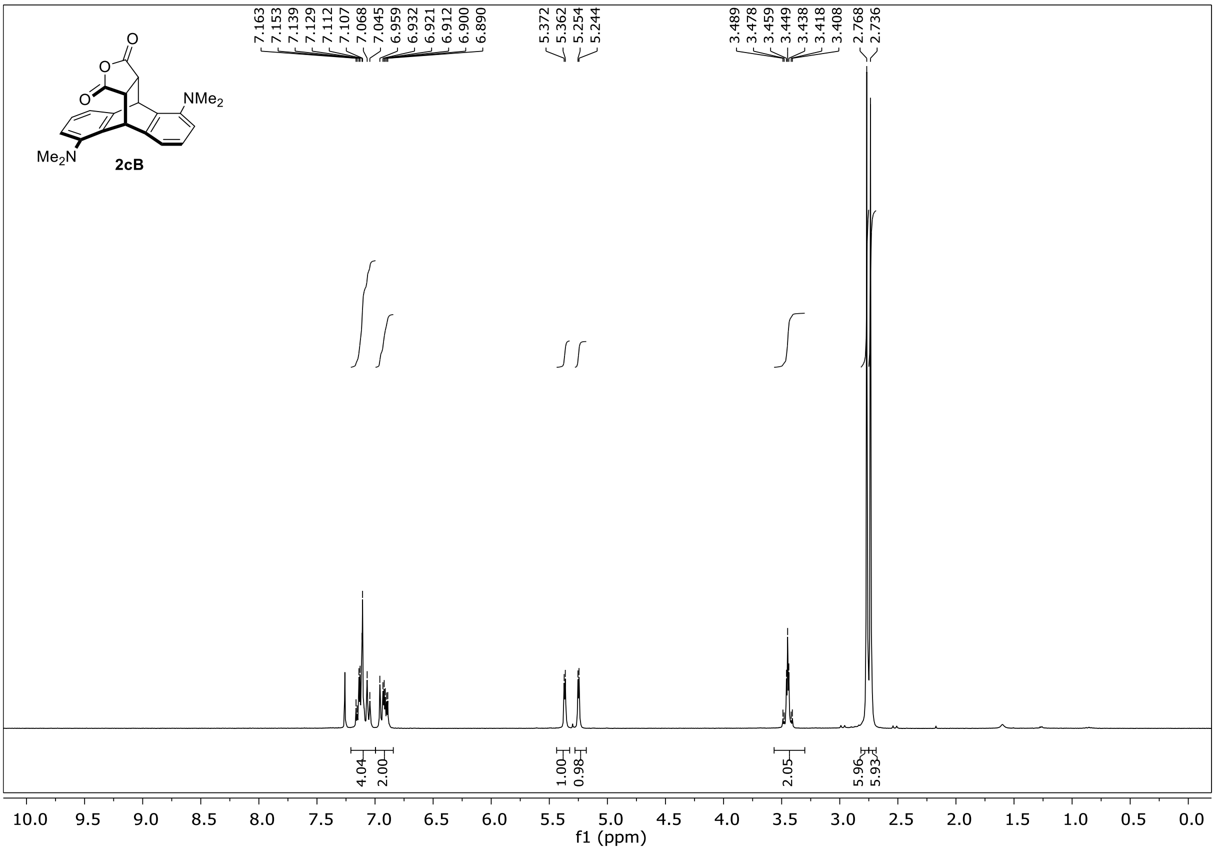

Supplementary Figure 30. ^{1}H NMR of **2cB** (300 MHz, $CDCl_3$)

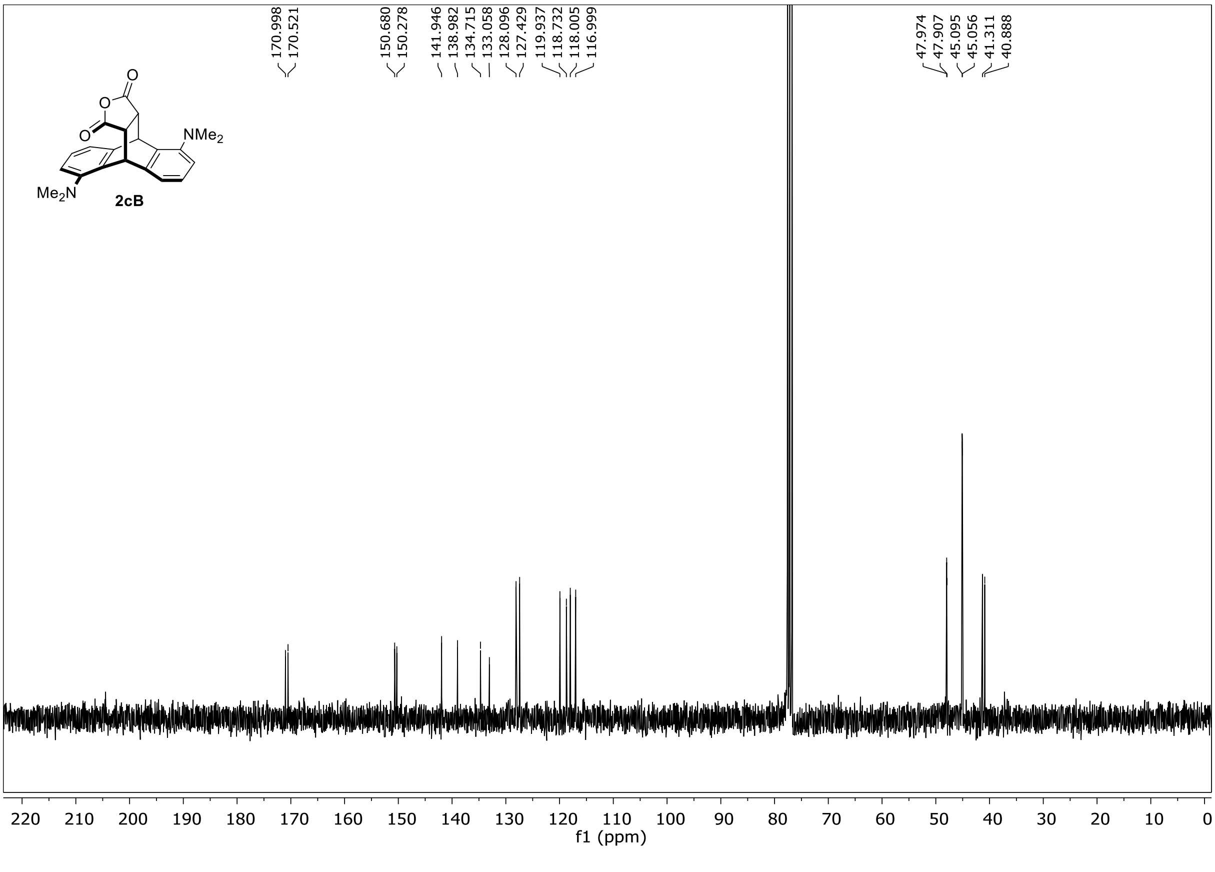

Supplementary Figure 31. ^{13}C NMR of **2cB** (75 MHz, $CDCl_3$)

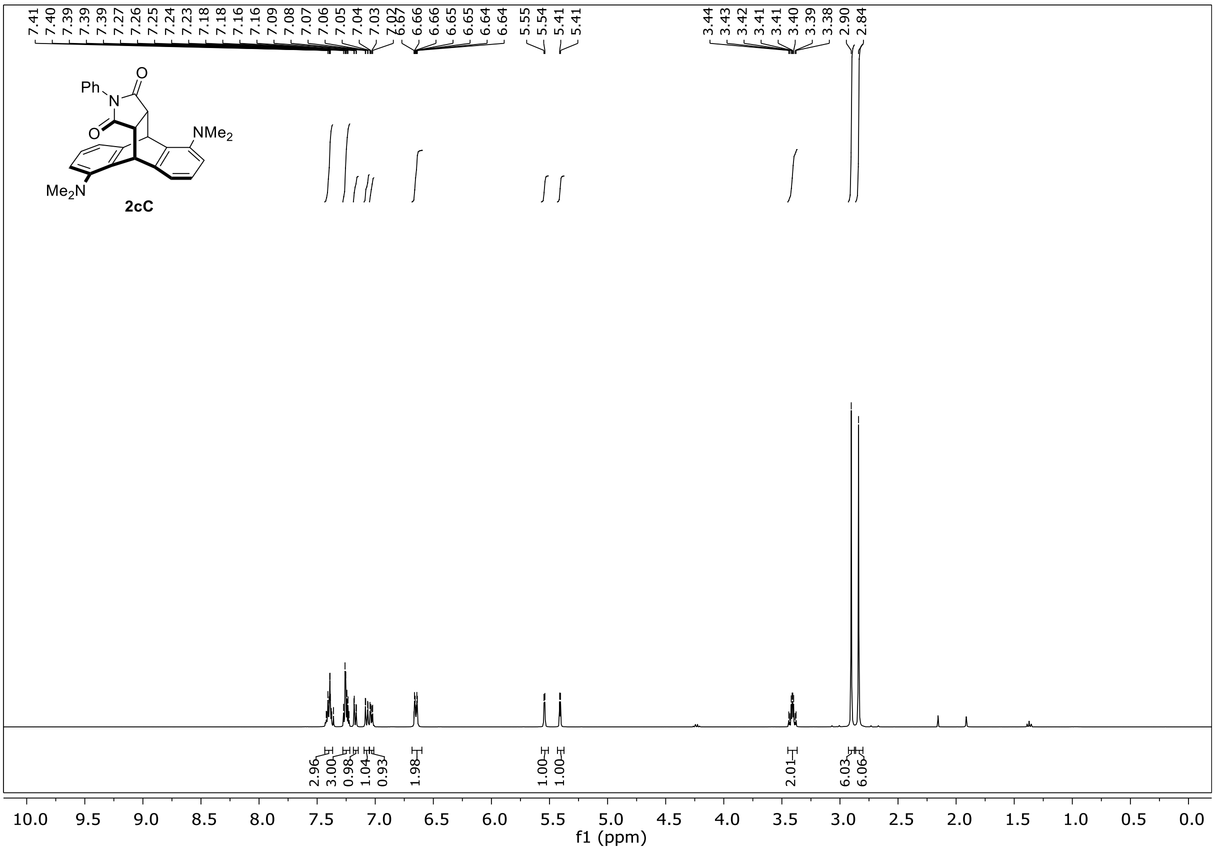

Supplementary Figure 32. ^{1}H NMR of **2cC** (400 MHz, $CDCl_3$)

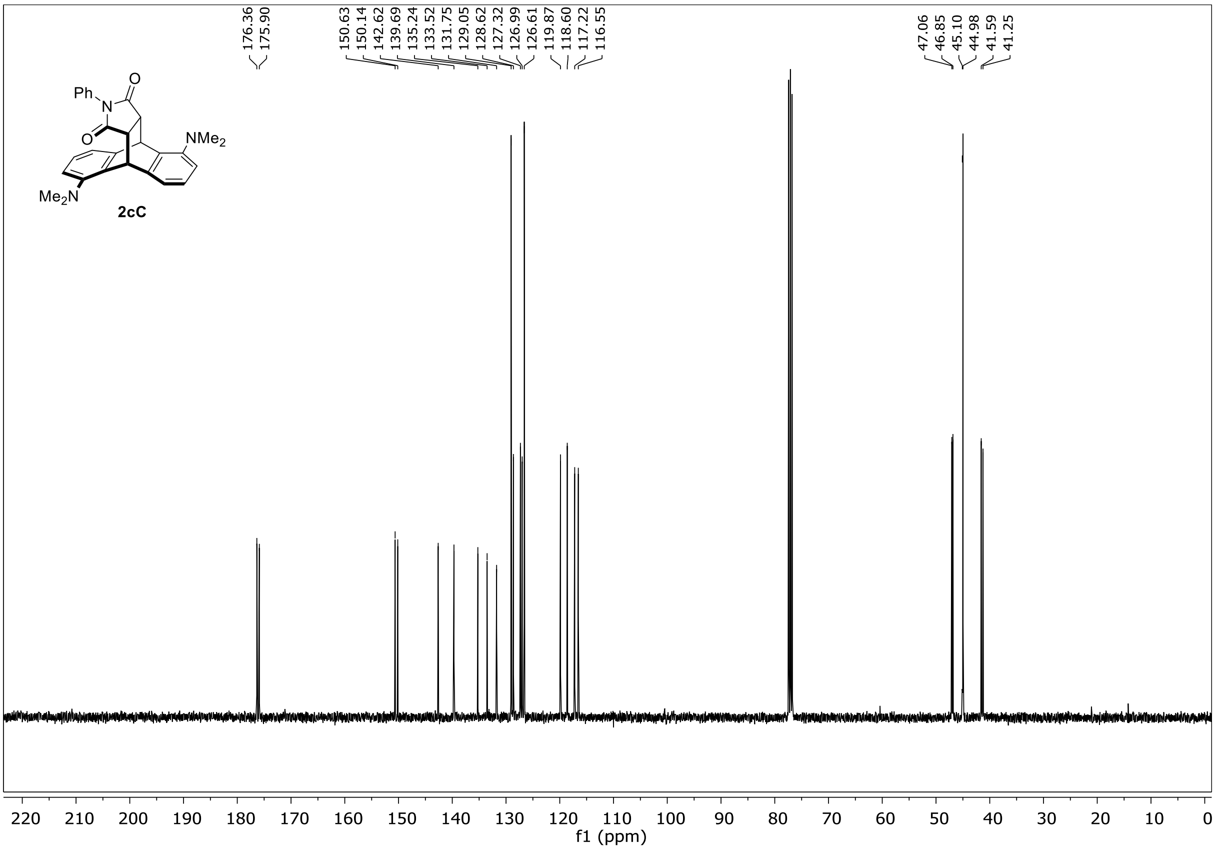

Supplementary Figure 33. ^{13}C NMR of **2cC** (101 MHz, $CDCl_3$)

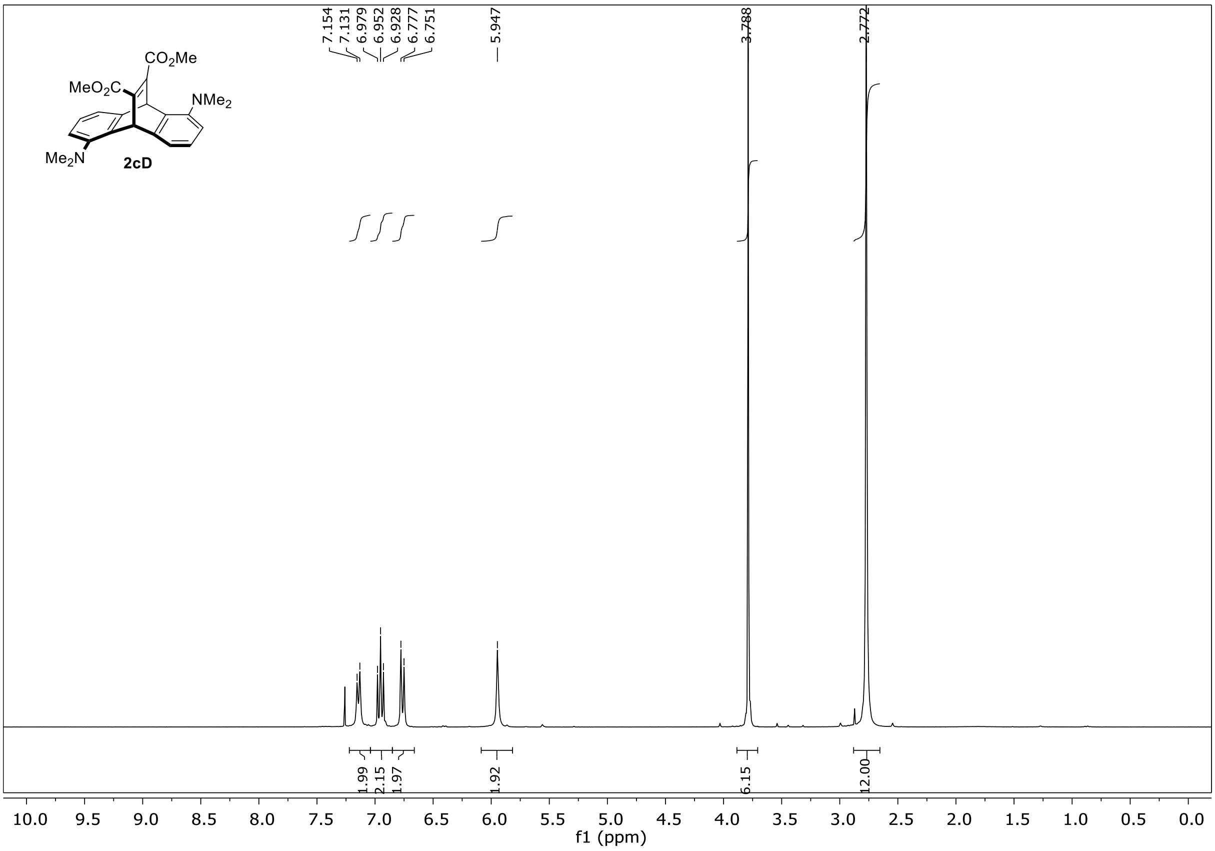

Supplementary Figure 34. ^{1}H NMR of **2cD** (300 MHz, $CDCl_3$)

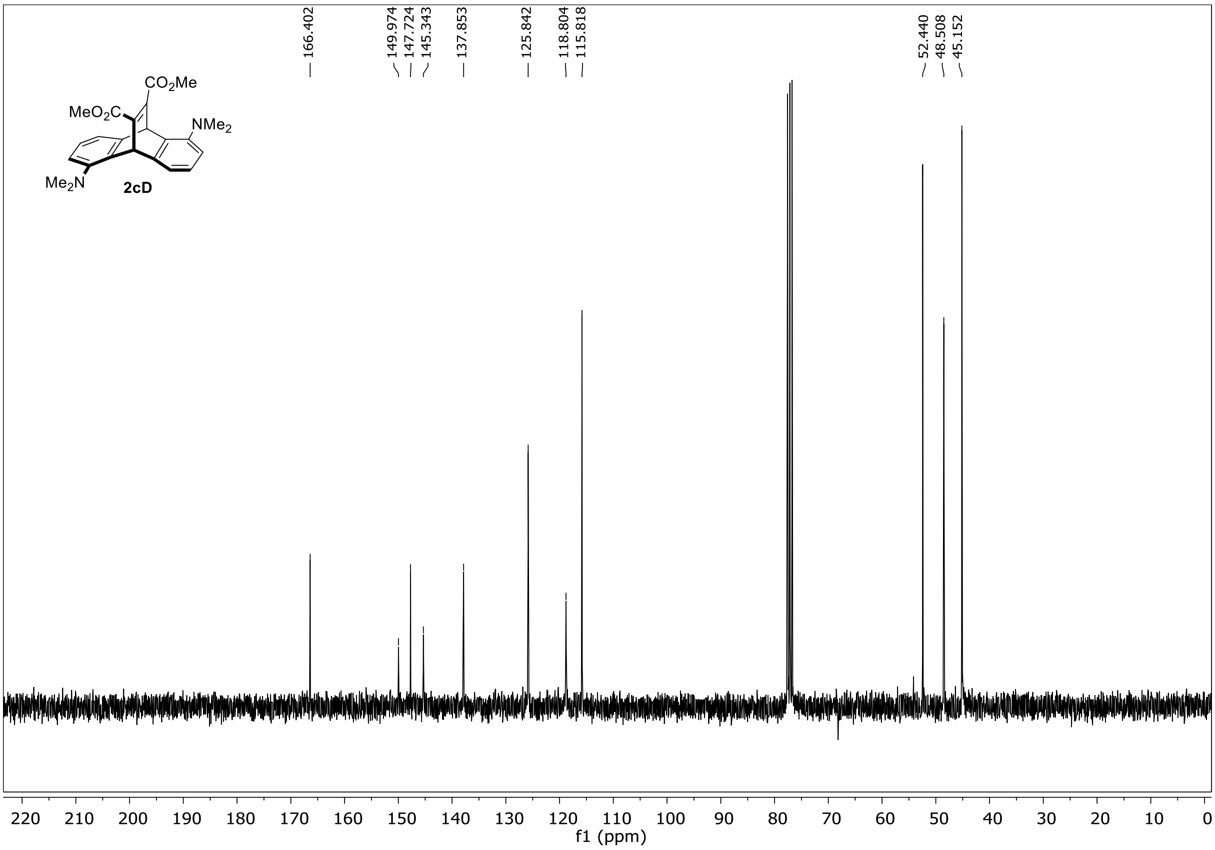

Supplementary Figure 35. ^{13}C NMR of **2cD** (75 MHz, $CDCl_3$)

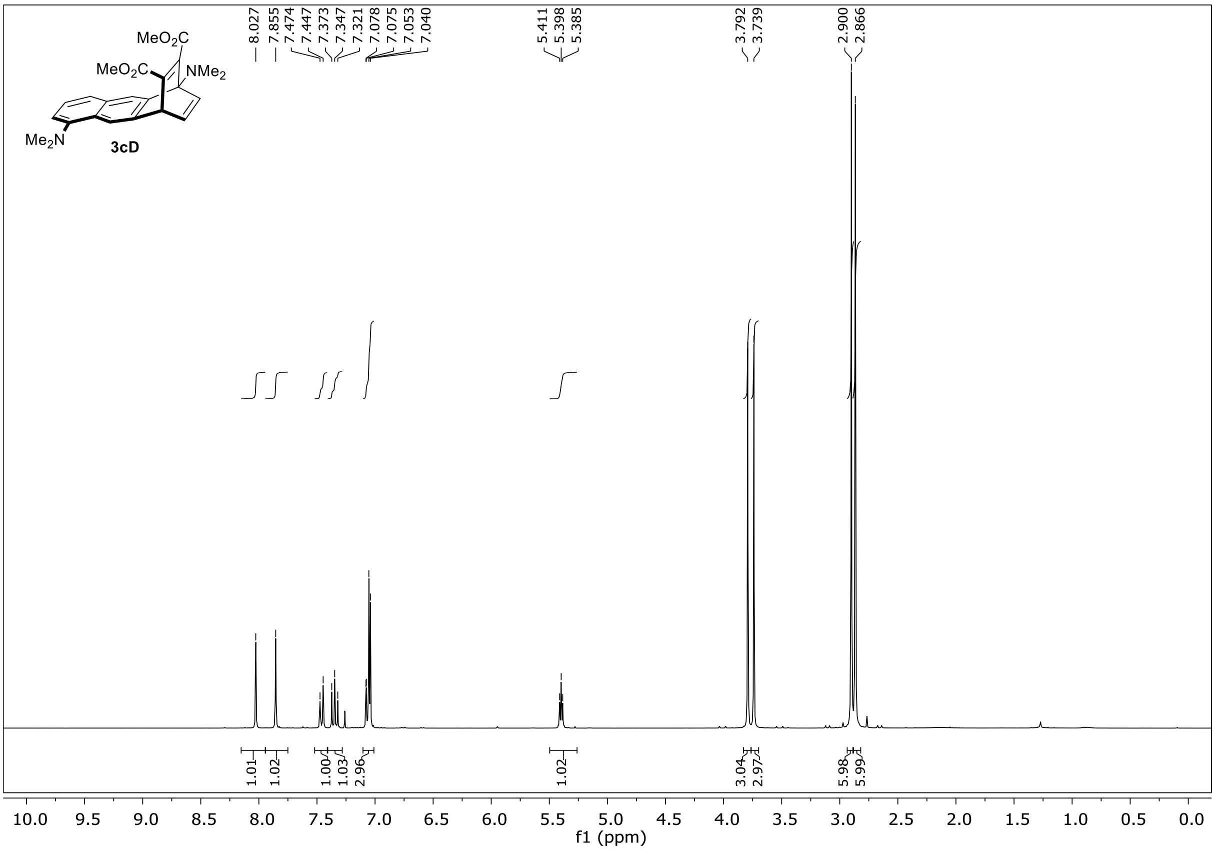

Supplementary Figure 36. ^{1}H NMR of **3cD** (300 MHz, $CDCl_3$)

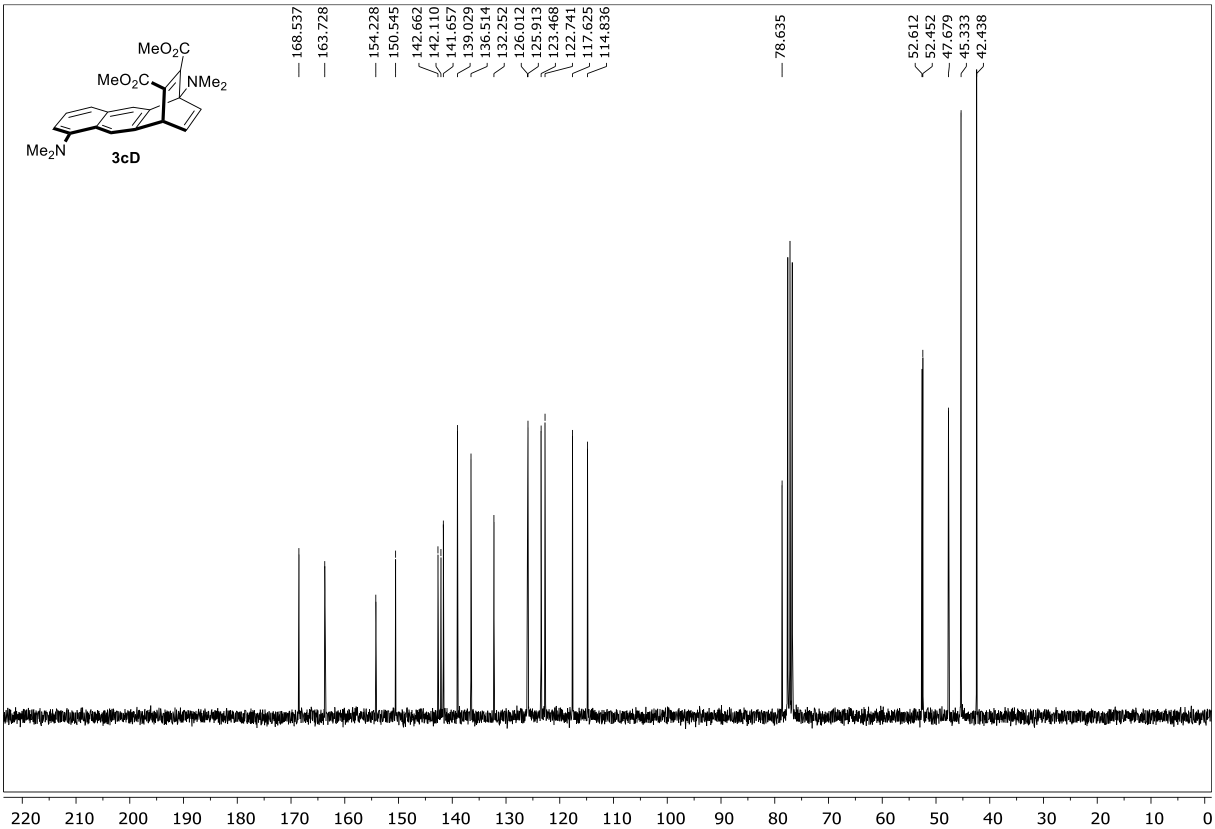

Supplementary Figure 37. ^{13}C NMR of **3cD** (75 MHz, $CDCl_3$)

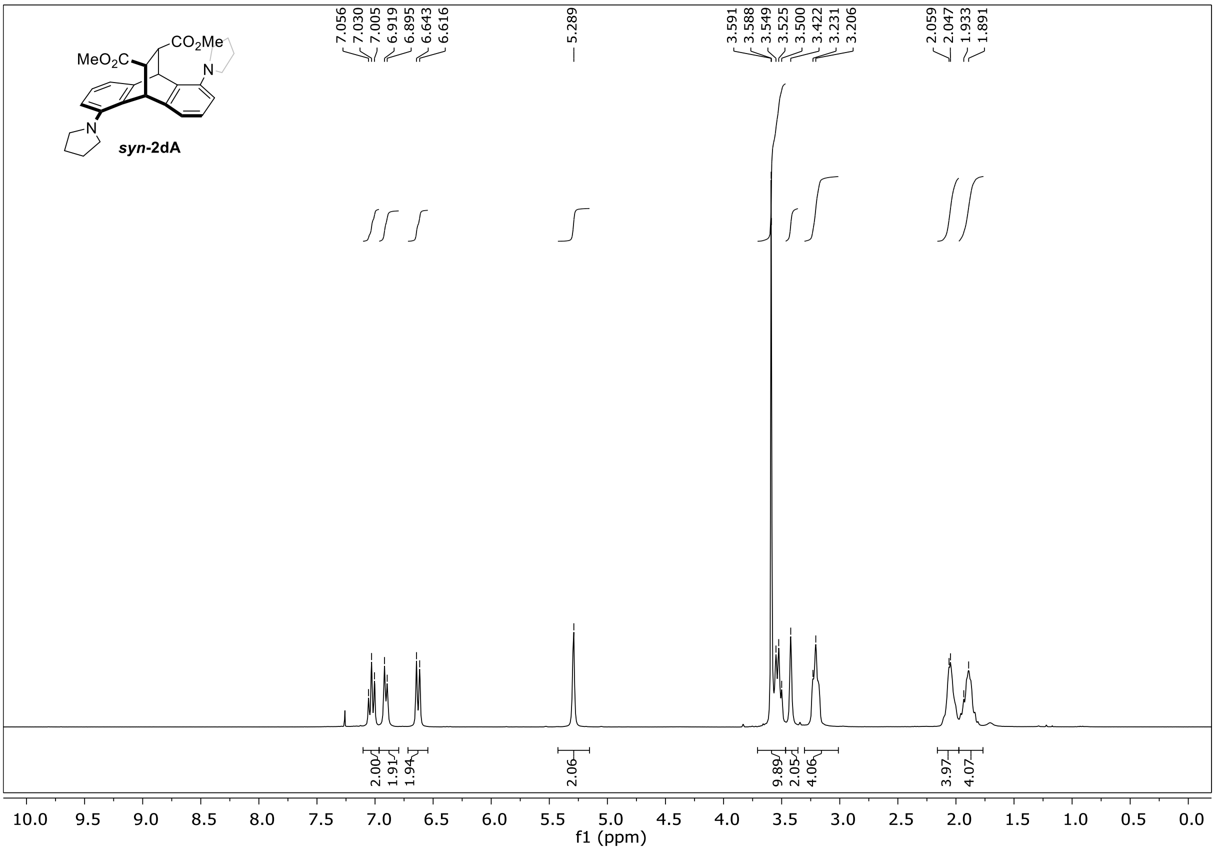

Supplementary Figure 38. ^{1}H NMR of ***syn*-2dA** (300 MHz, $CDCl_3$)

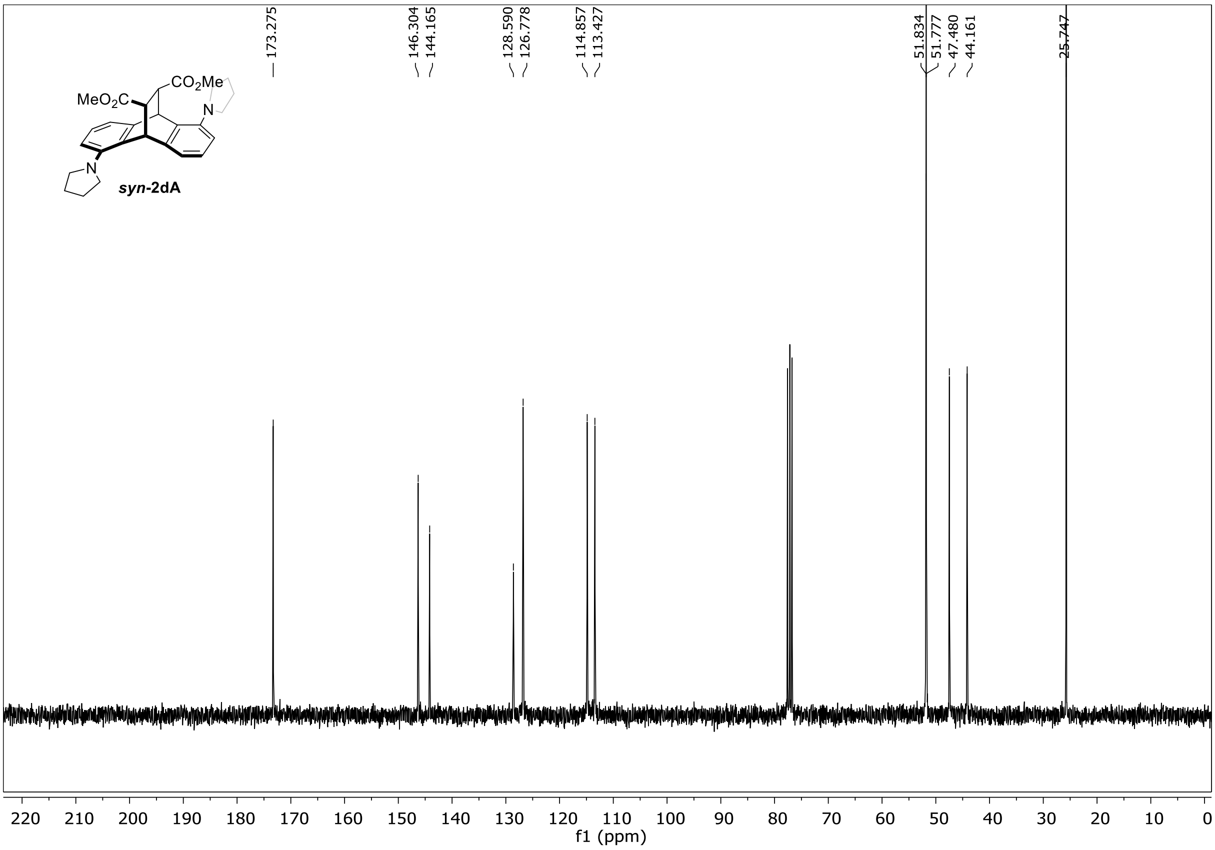

Supplementary Figure 39. ^{13}C NMR of ***syn*-2dA** (75 MHz, $CDCl_3$)

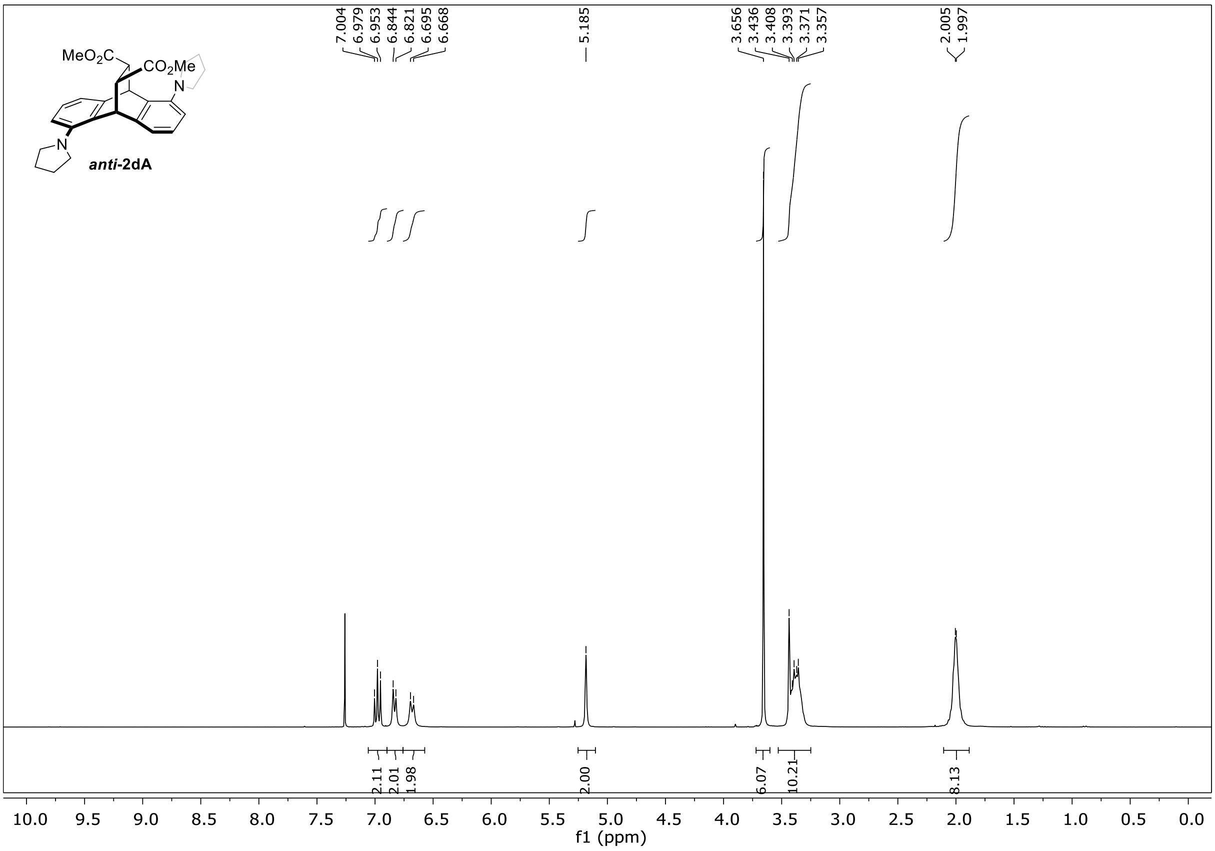

Supplementary Figure 40. ^{1}H NMR of ***anti*-2dA** (300 MHz, $CDCl_3$)

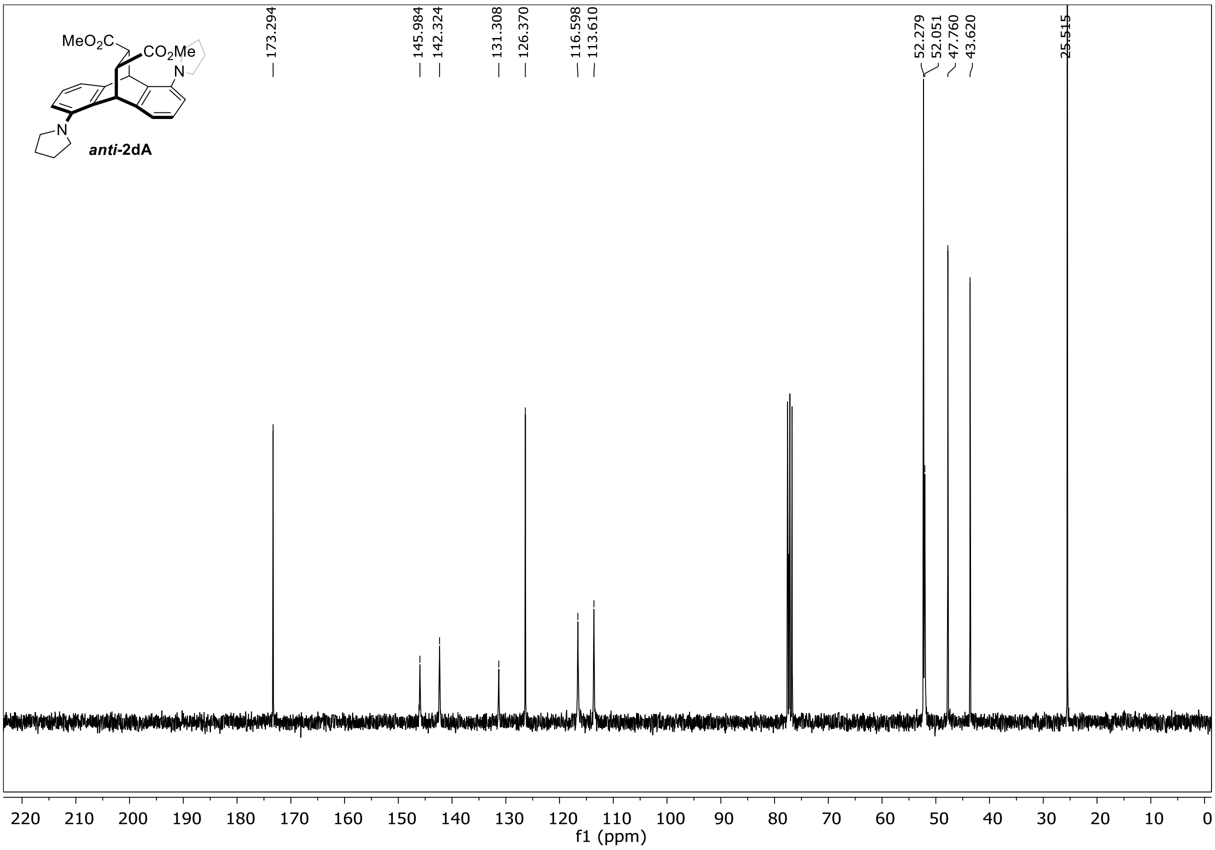

Supplementary Figure 41. ^{13}C NMR of ***anti*-2dA** (75 MHz, $CDCl_3$)

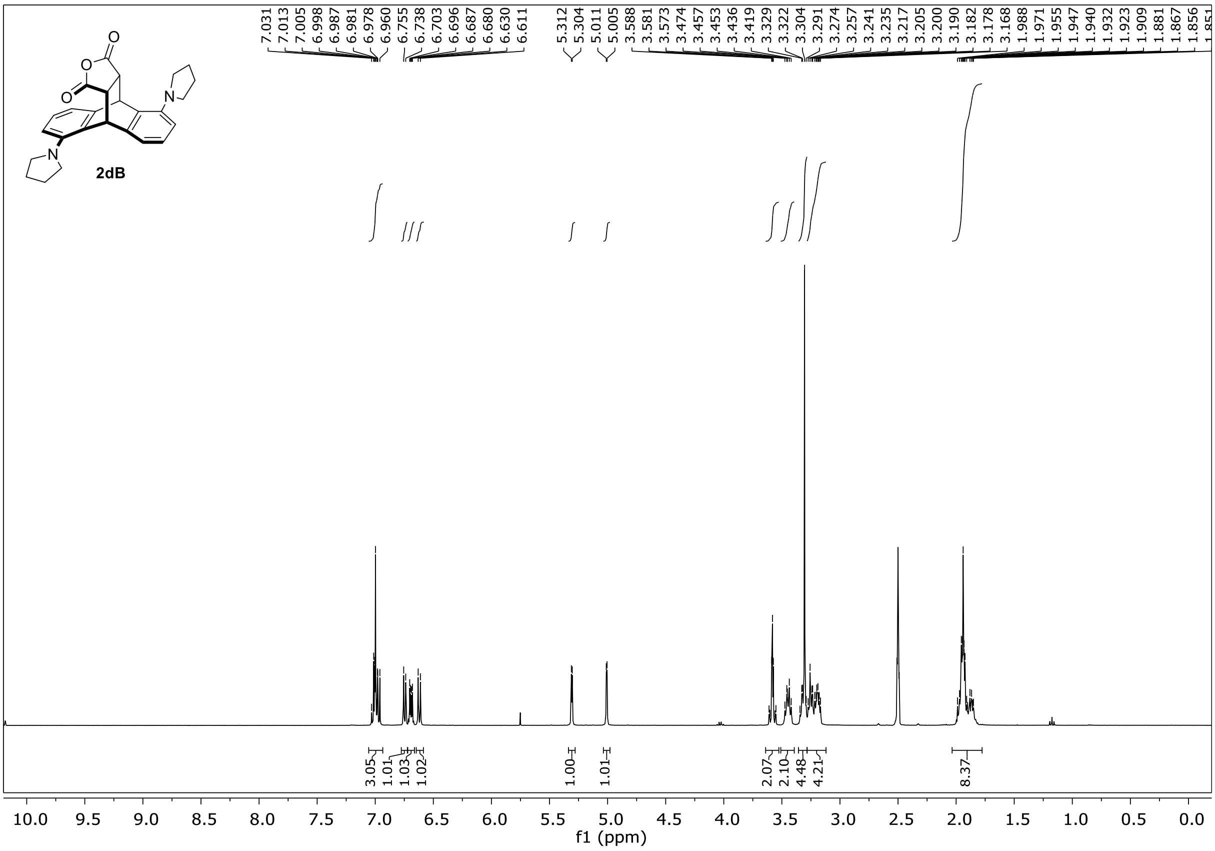

Supplementary Figure 42. ^{1}H NMR of **2dB** (400 MHz, $CDCl_3$)

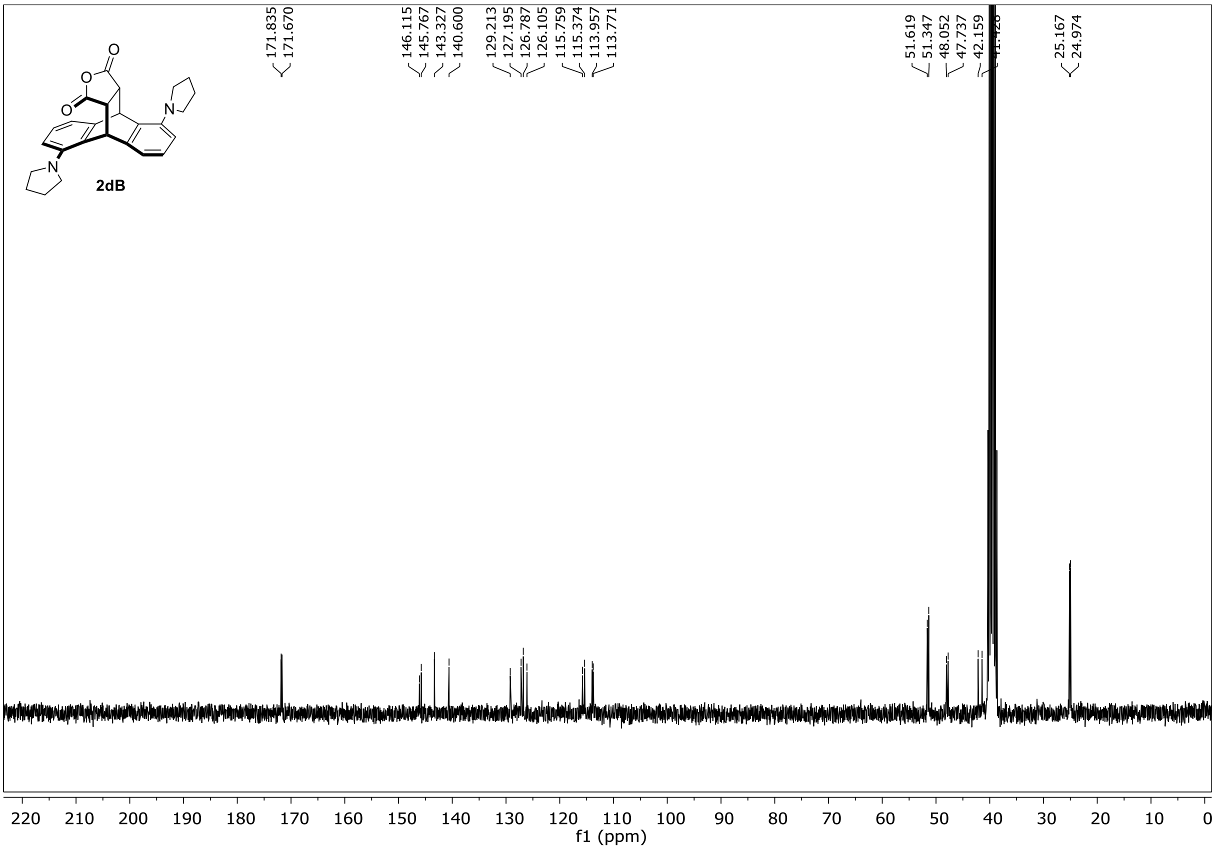

Supplementary Figure 43. ^{13}C NMR of **2dB** (101 MHz, $CDCl_3$)

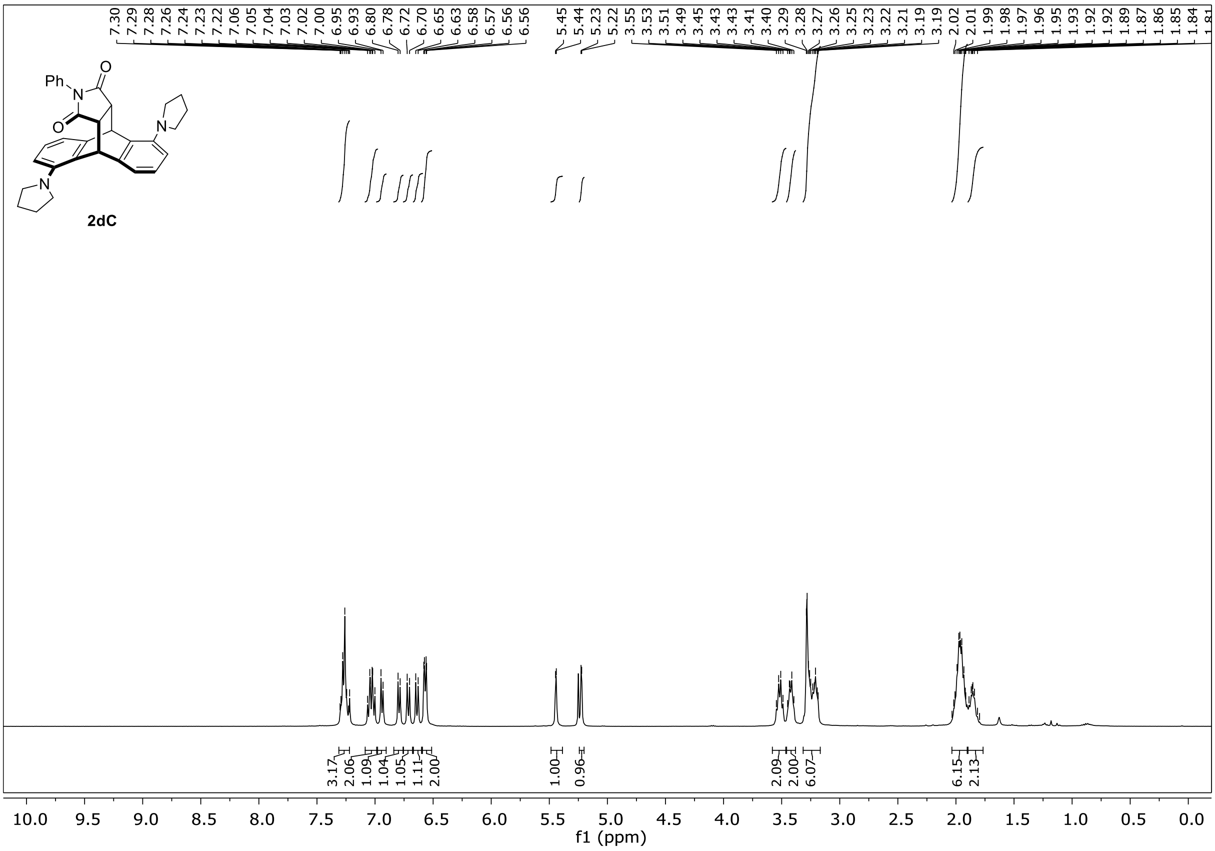

Supplementary Figure 44. ^{1}H NMR of **2dC** (400 MHz, $CDCl_3$)

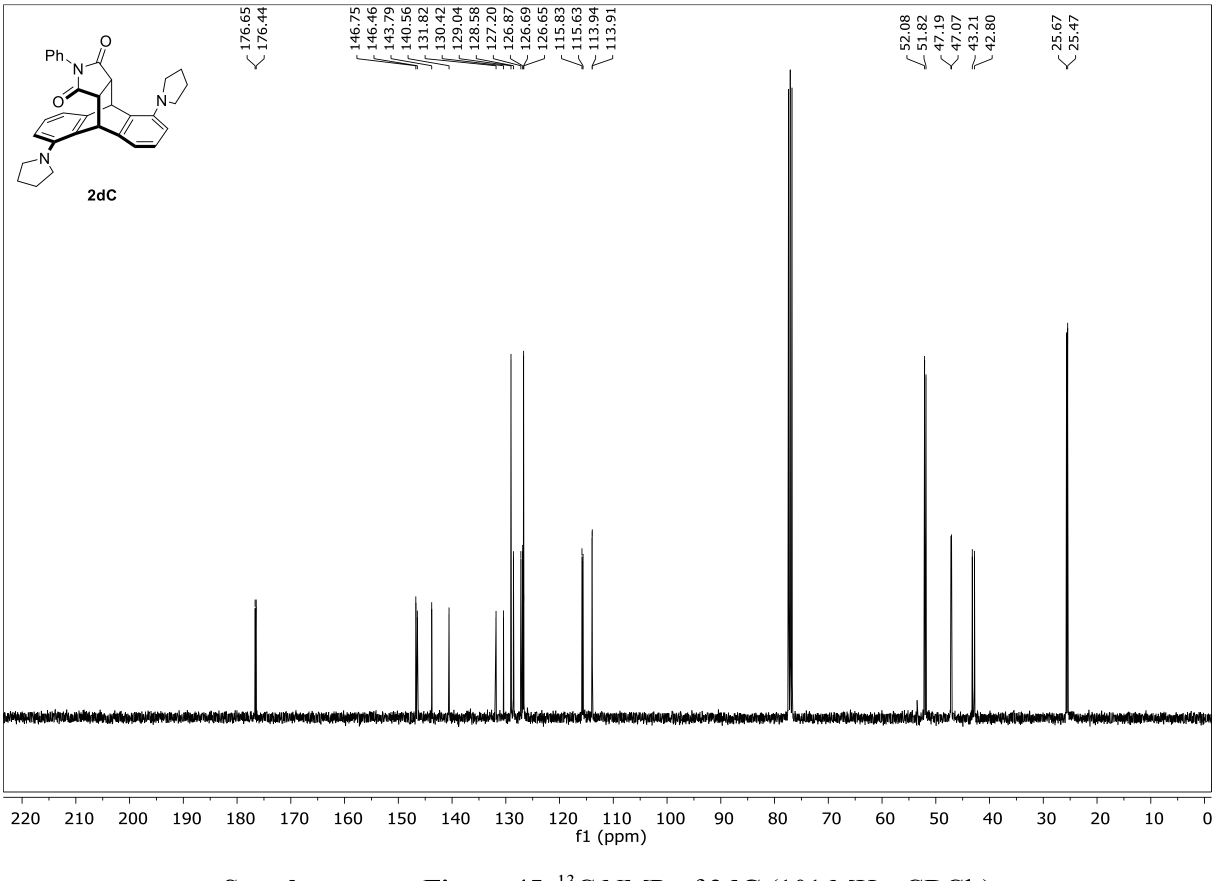

Supplementary Figure 45. ^{13}C NMR of **2dC** (101 MHz, $CDCl_3$)

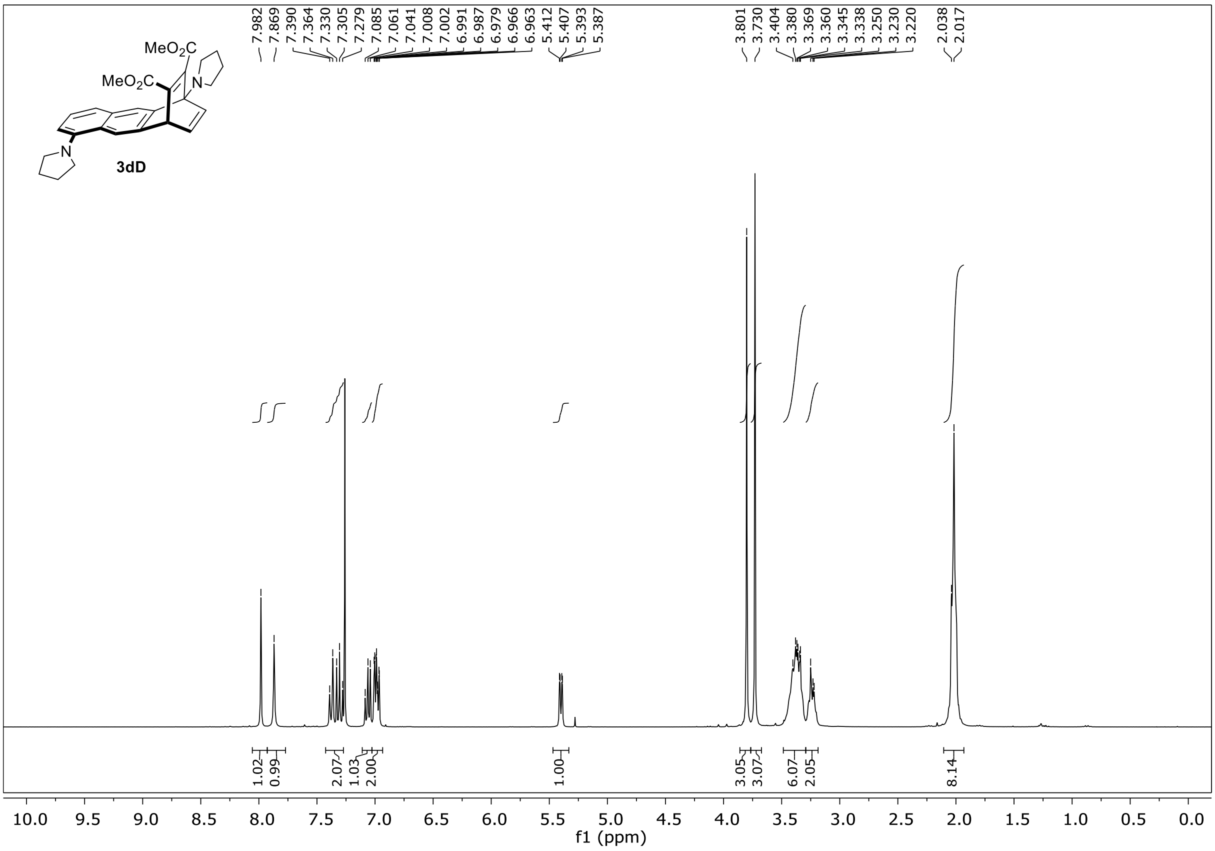

Supplementary Figure 46. ^{1}H NMR of **3dD** (300 MHz, $CDCl_3$)

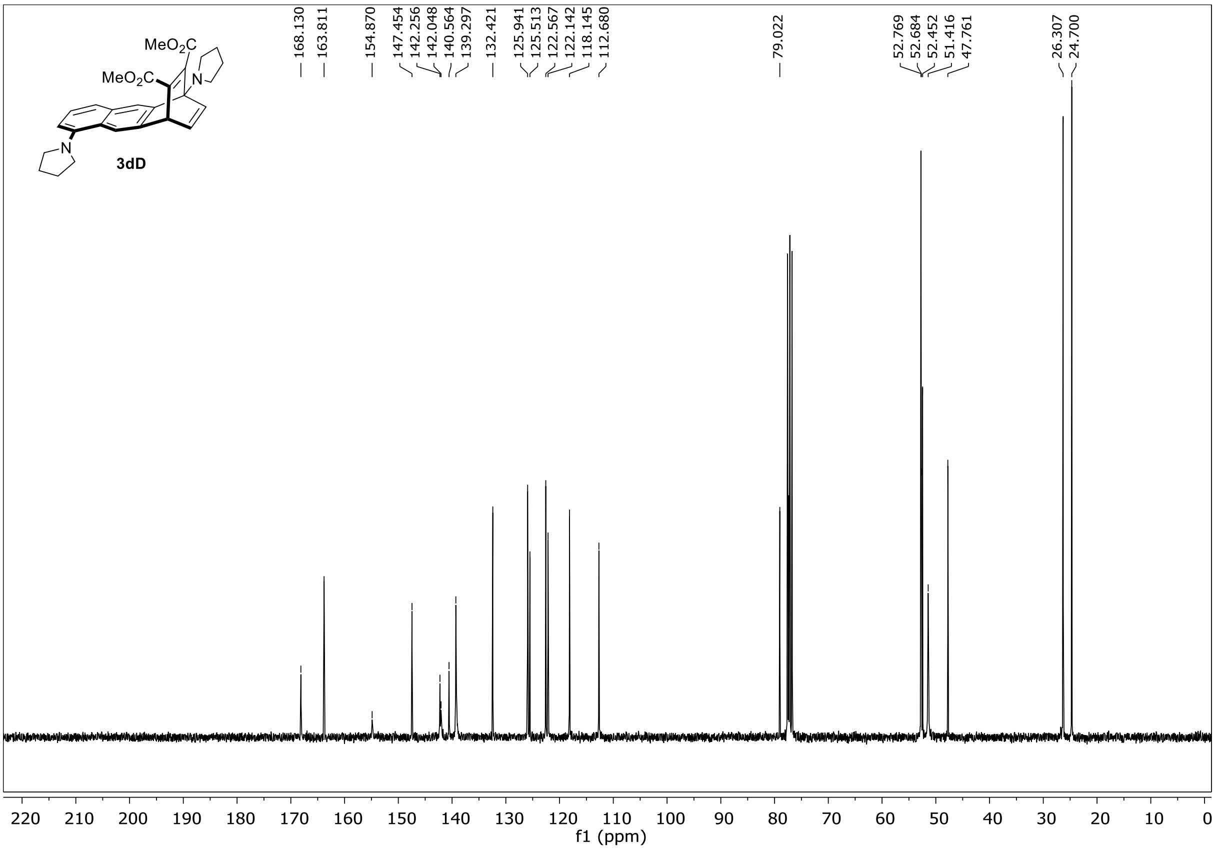

Supplementary Figure 47. ^{13}C NMR of **3dD** (75 MHz, $CDCl_3$)

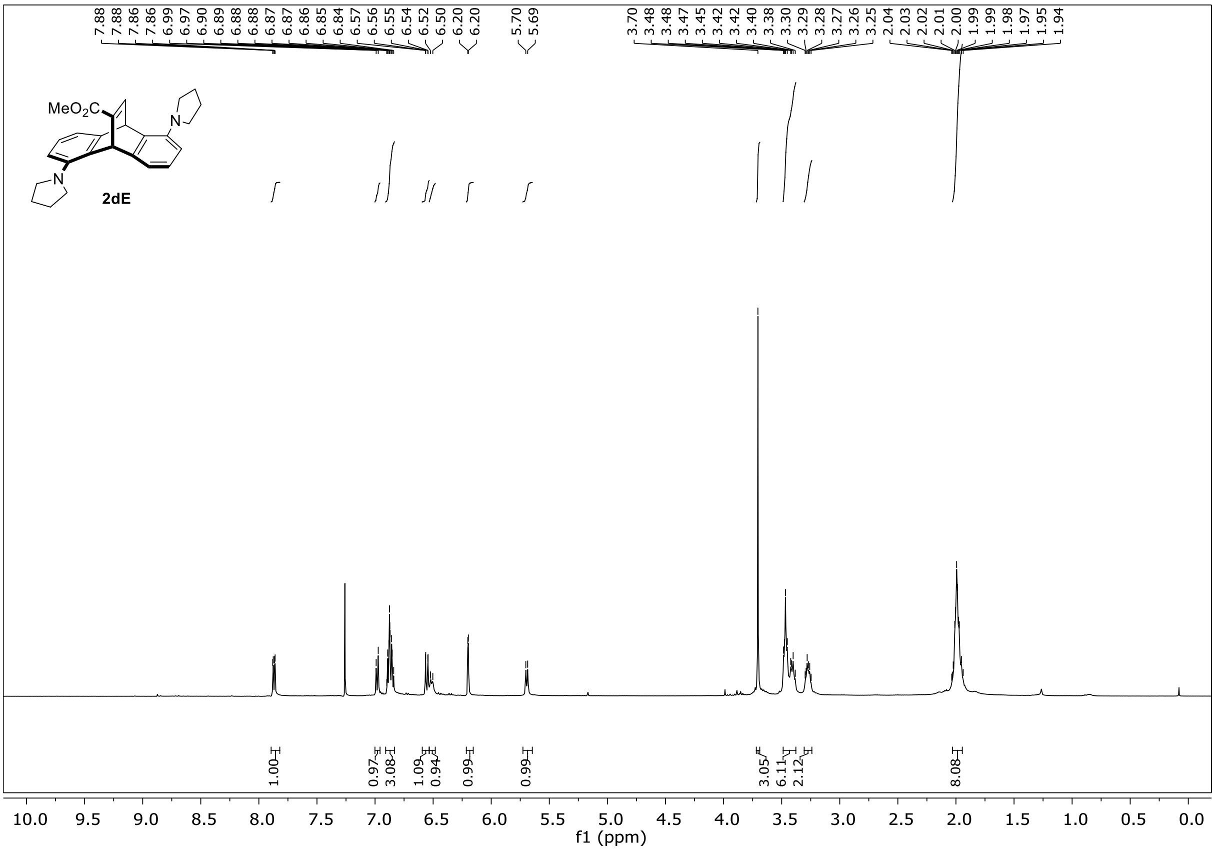

Supplementary Figure 48. ^{1}H NMR of **2dE** (400 MHz, $CDCl_3$)

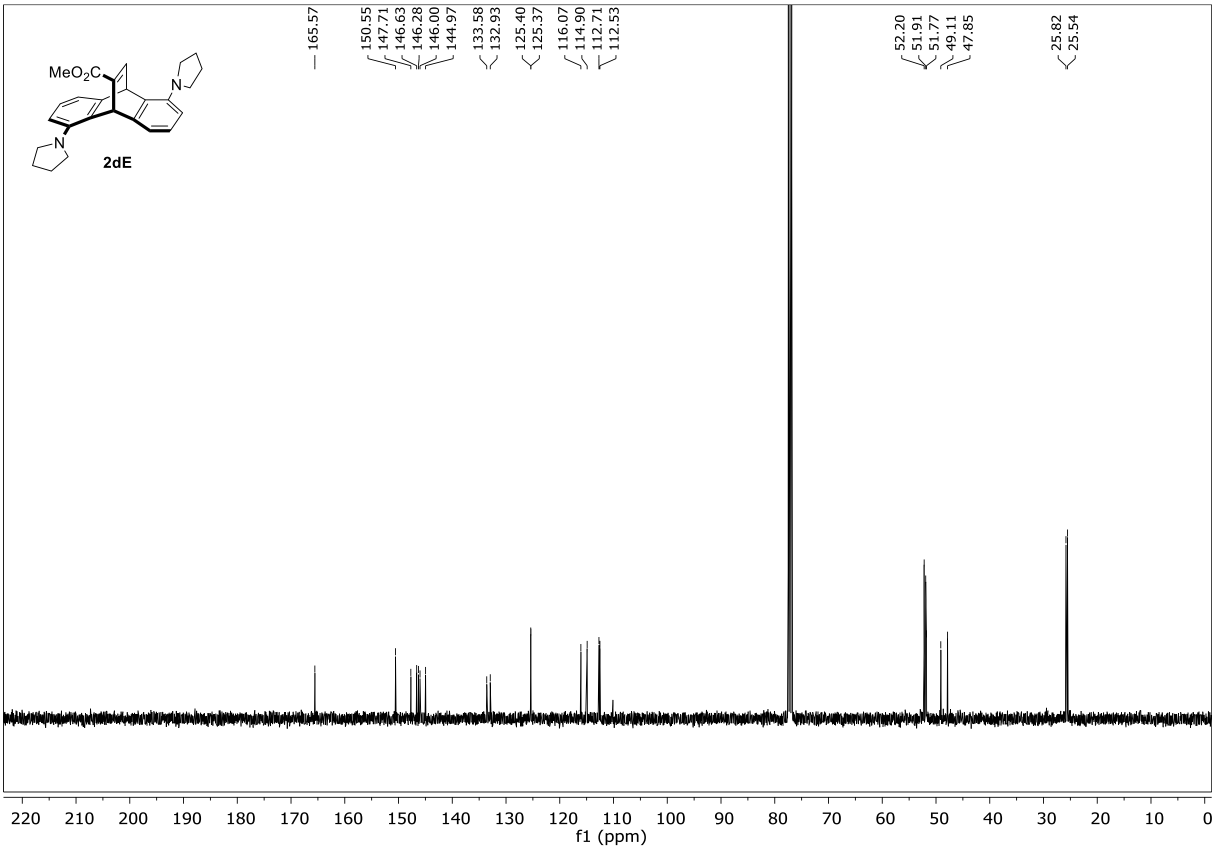

Supplementary Figure 49. ^{13}C NMR of **2dE** (101 MHz, $CDCl_3$)

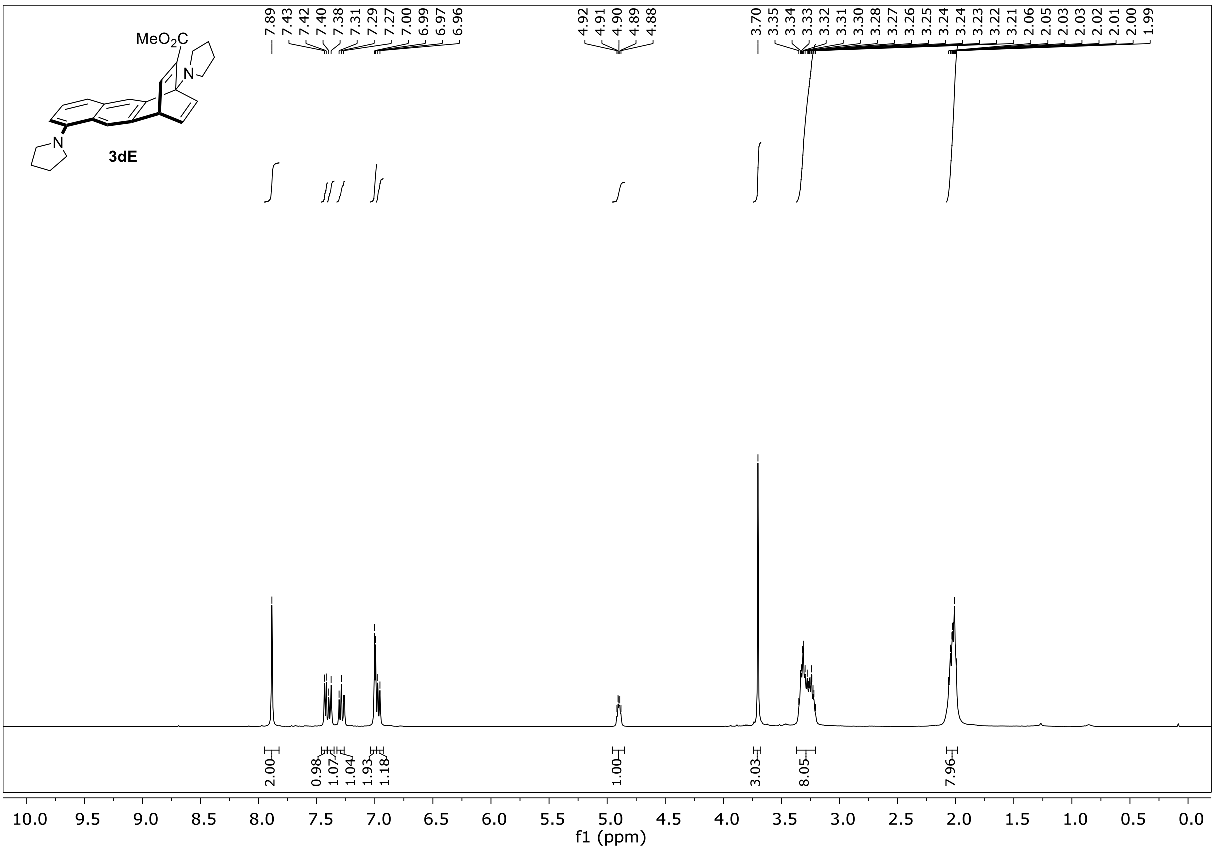

Supplementary Figure 50. ^{1}H NMR of **3dE** (400 MHz, $CDCl_3$)

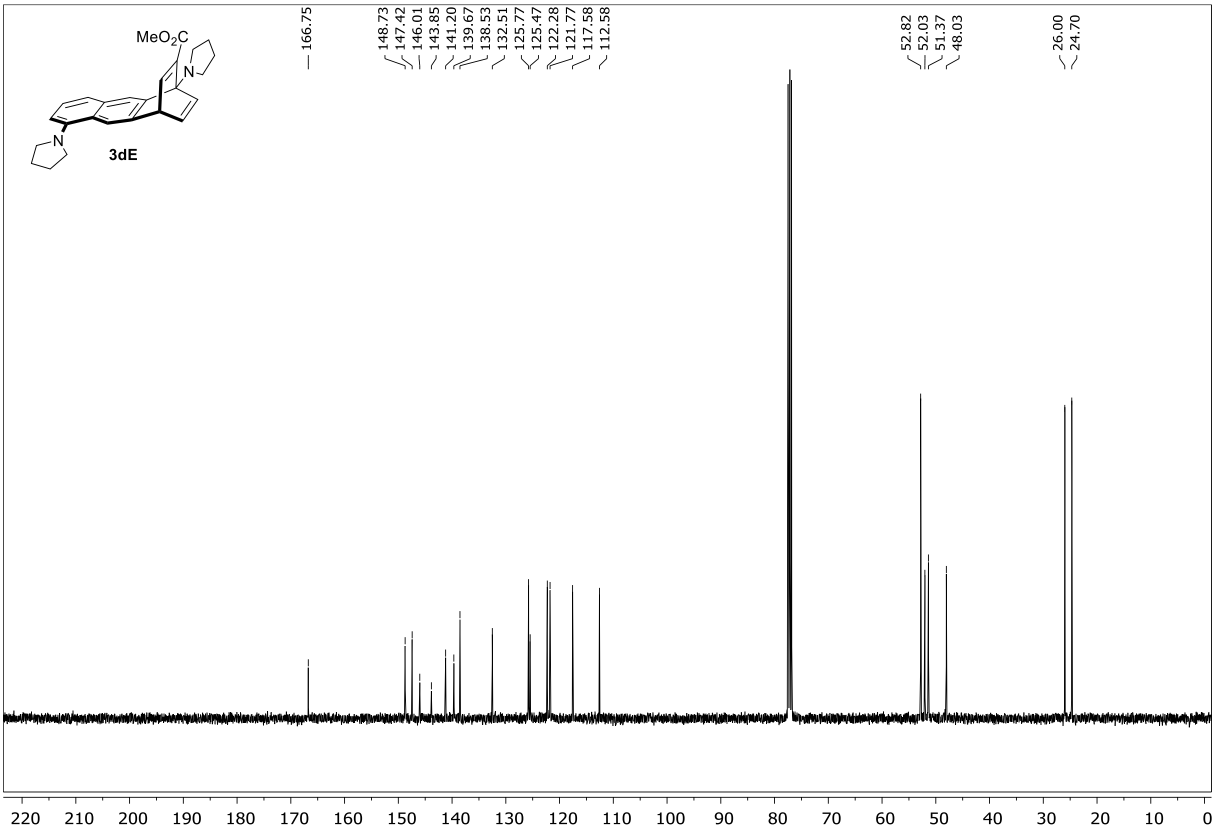

Supplementary Figure 51. ^{13}C NMR of **3dE** (101 MHz, $CDCl_3$)

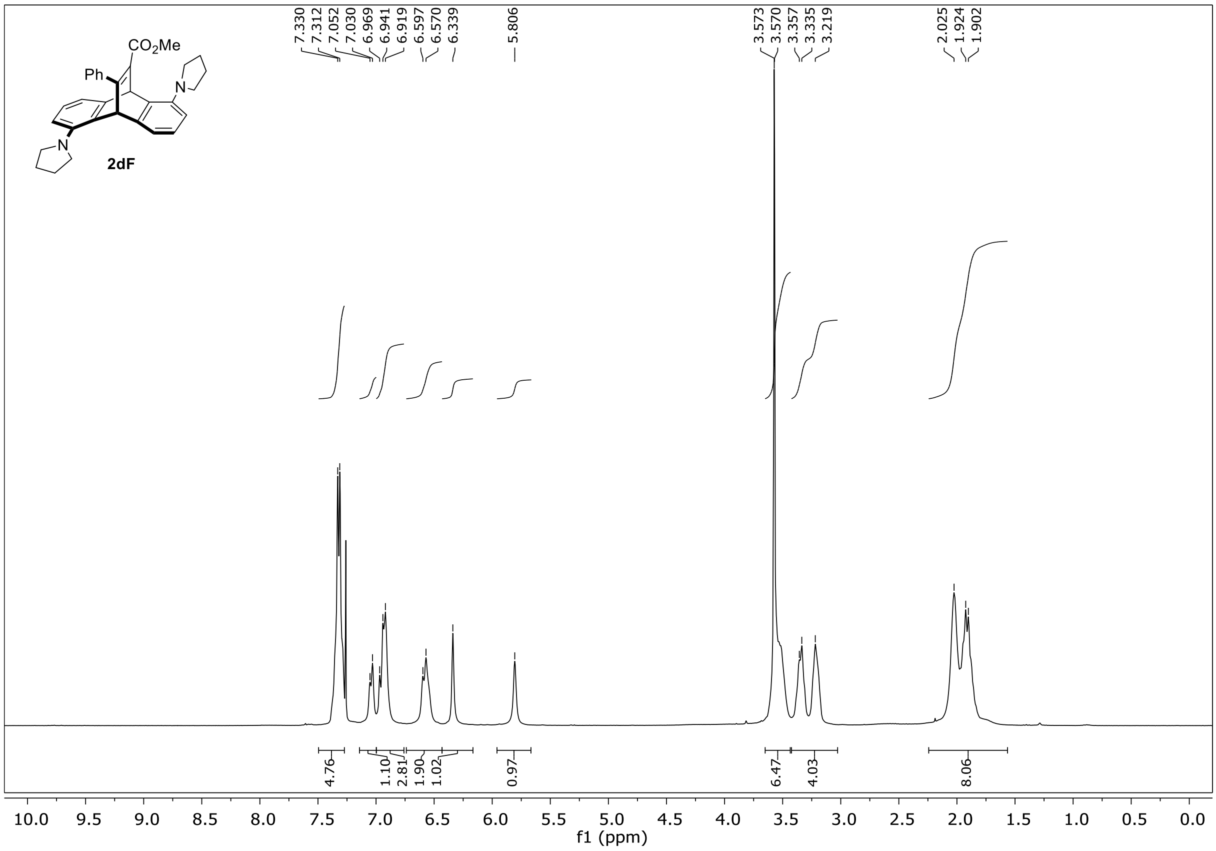

Supplementary Figure 52. ^{1}H NMR of **2dF** (300 MHz, $CDCl_3$)

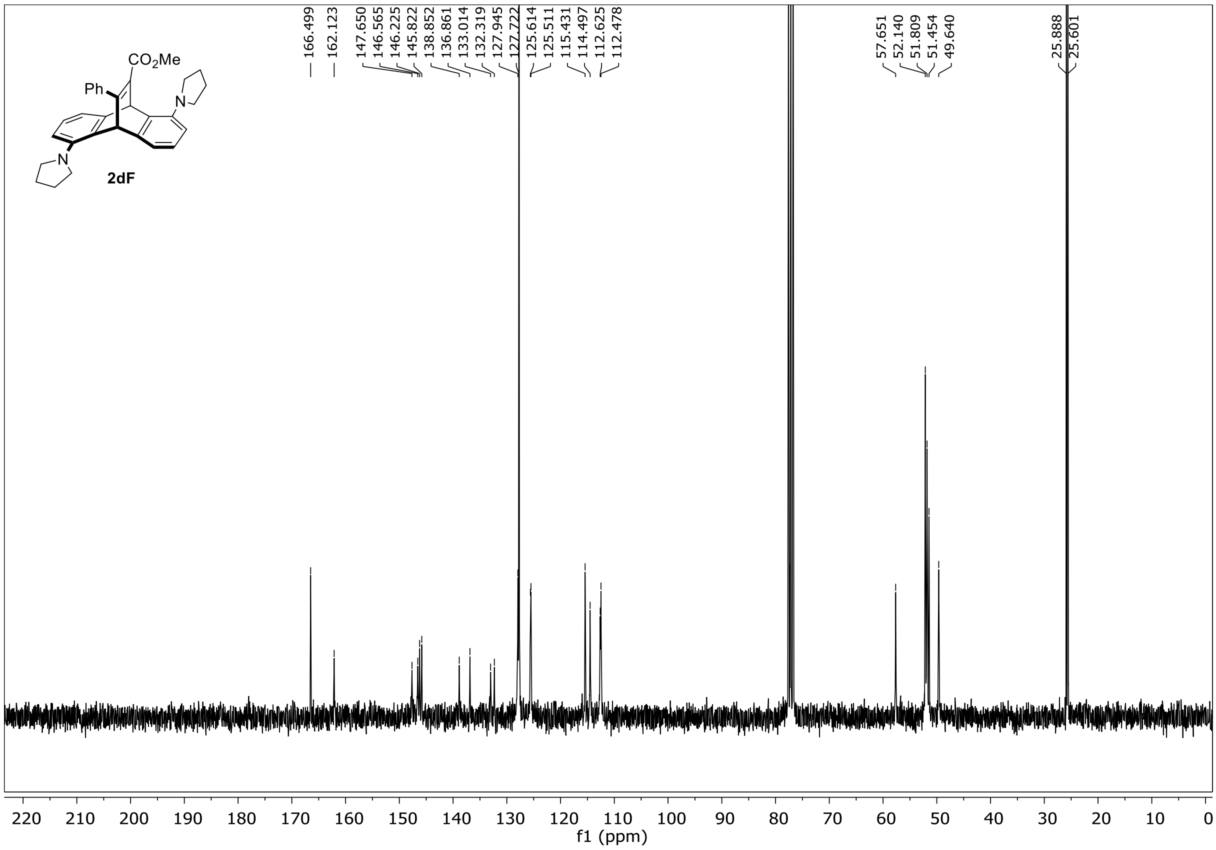

Supplementary Figure 53. ^{13}C NMR of **2dF** (75 MHz, $CDCl_3$)

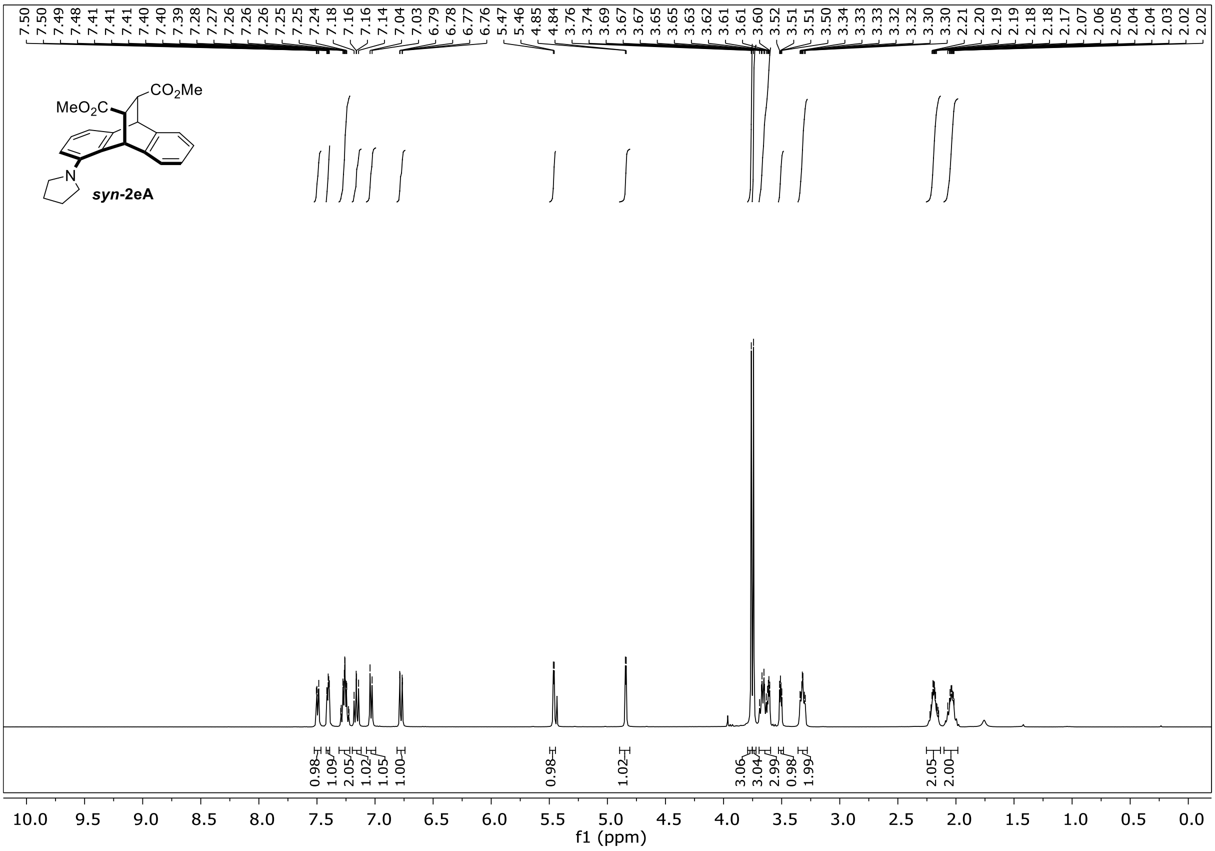

Supplementary Figure 54. ^{1}H NMR of ***syn*-2eA** (400 MHz, $CDCl_3$)

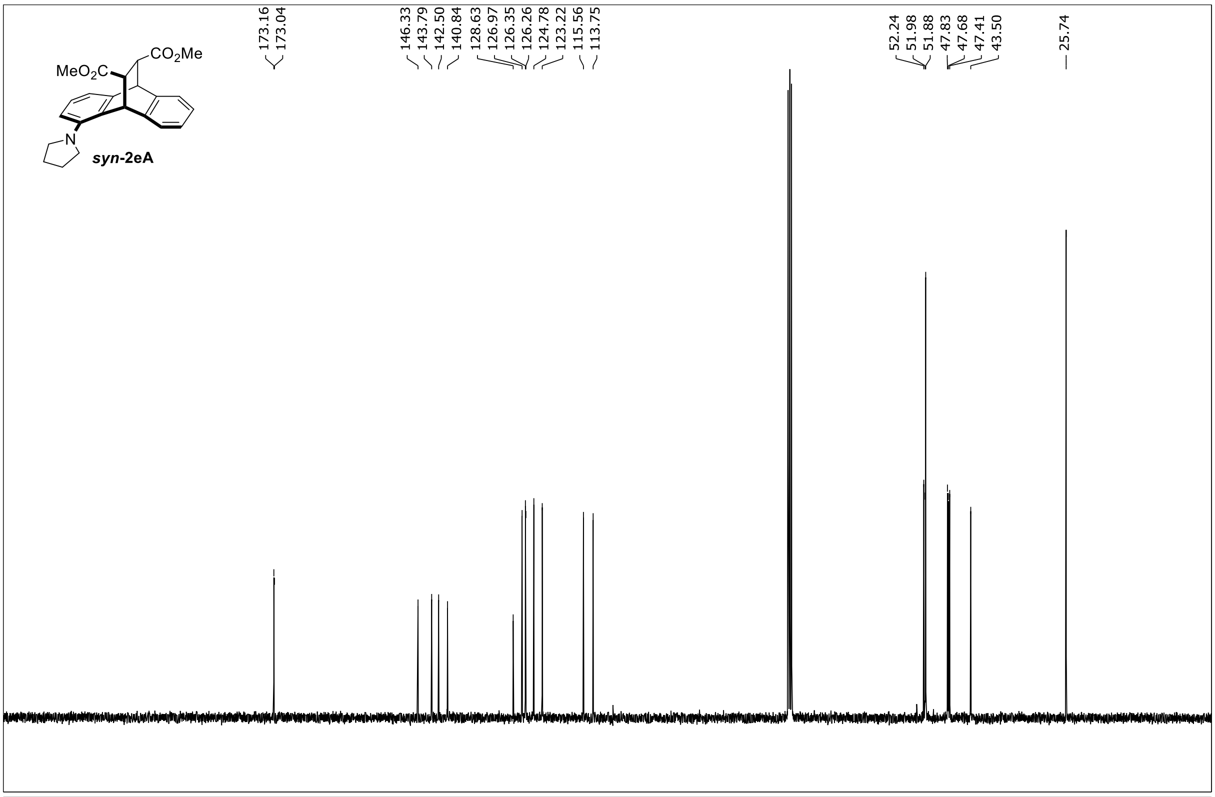

Supplementary Figure 55. ^{13}C NMR of ***syn*-2eA** (101 MHz, $CDCl_3$)

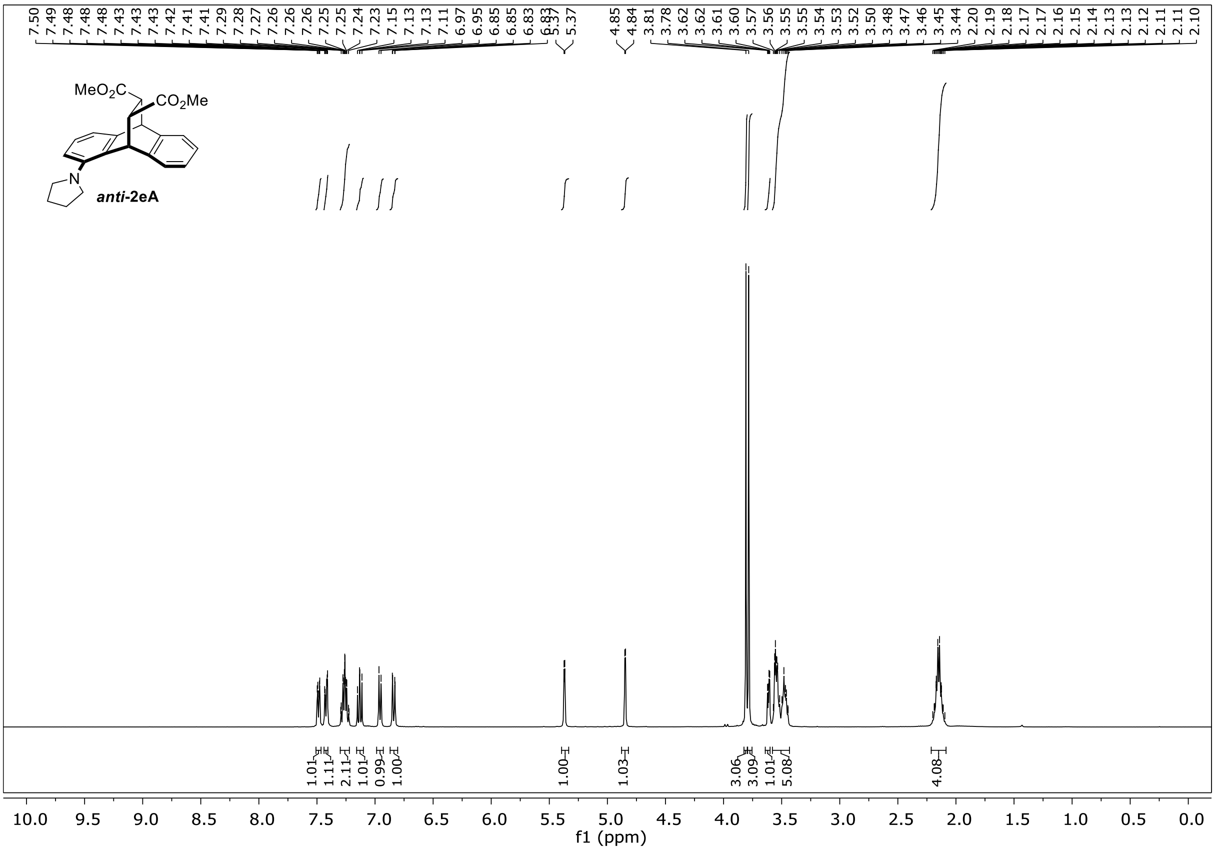

Supplementary Figure 56. ^{1}H NMR of ***anti*-2eA** (400 MHz, $CDCl_3$)

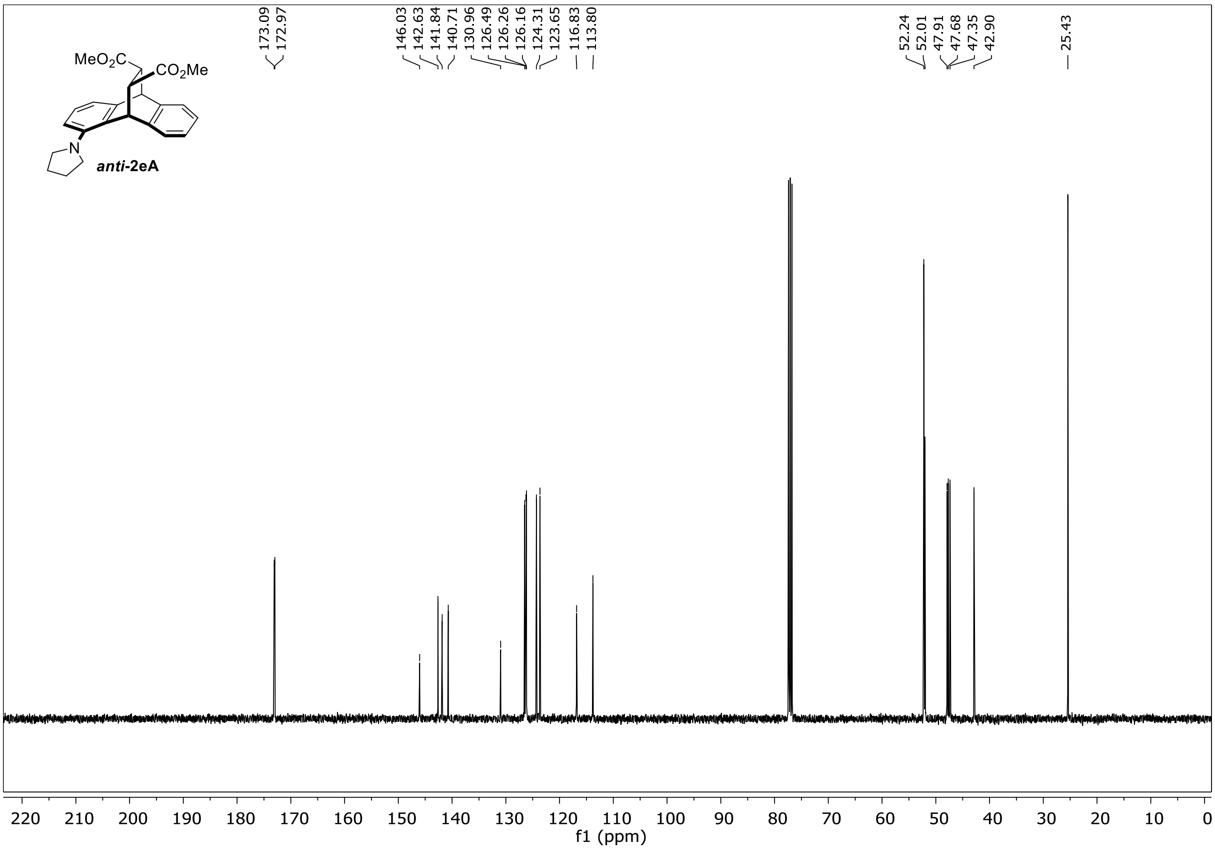

Supplementary Figure 57. ^{13}C NMR of ***anti*-2eA** (101 MHz, $CDCl_3$)

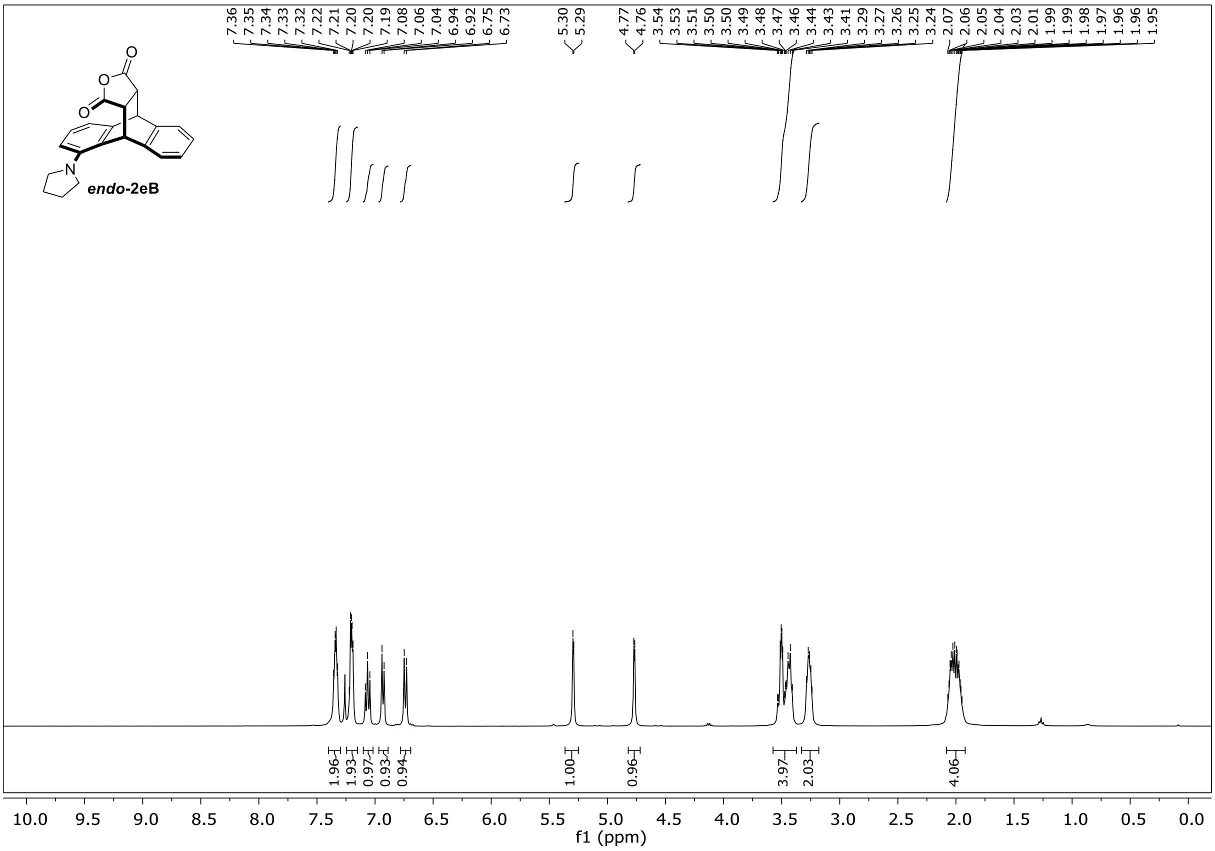

Supplementary Figure 58. ^{1}H NMR of ***endo*-2eB** (400 MHz, $CDCl_3$)

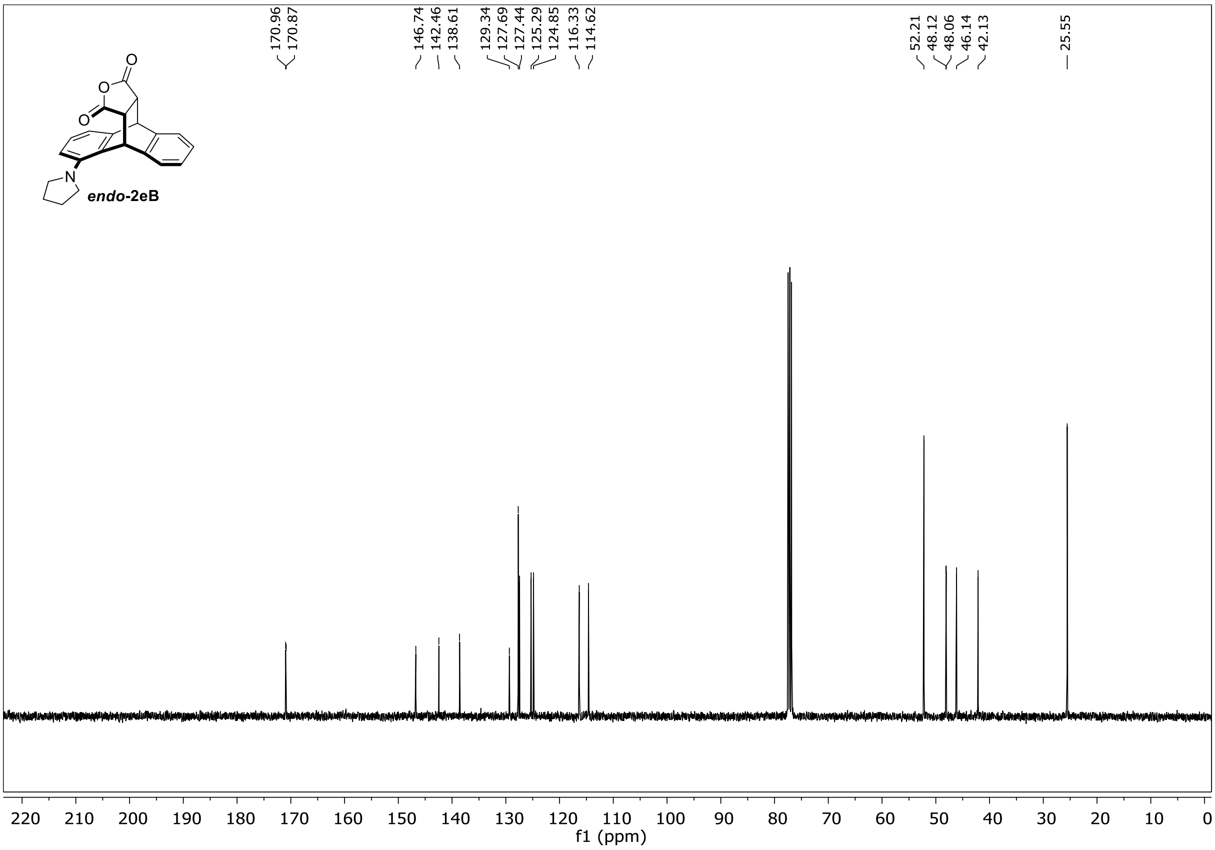

Supplementary Figure 59. ^{13}C NMR of ***endo*-2eB** (101 MHz, $CDCl_3$)

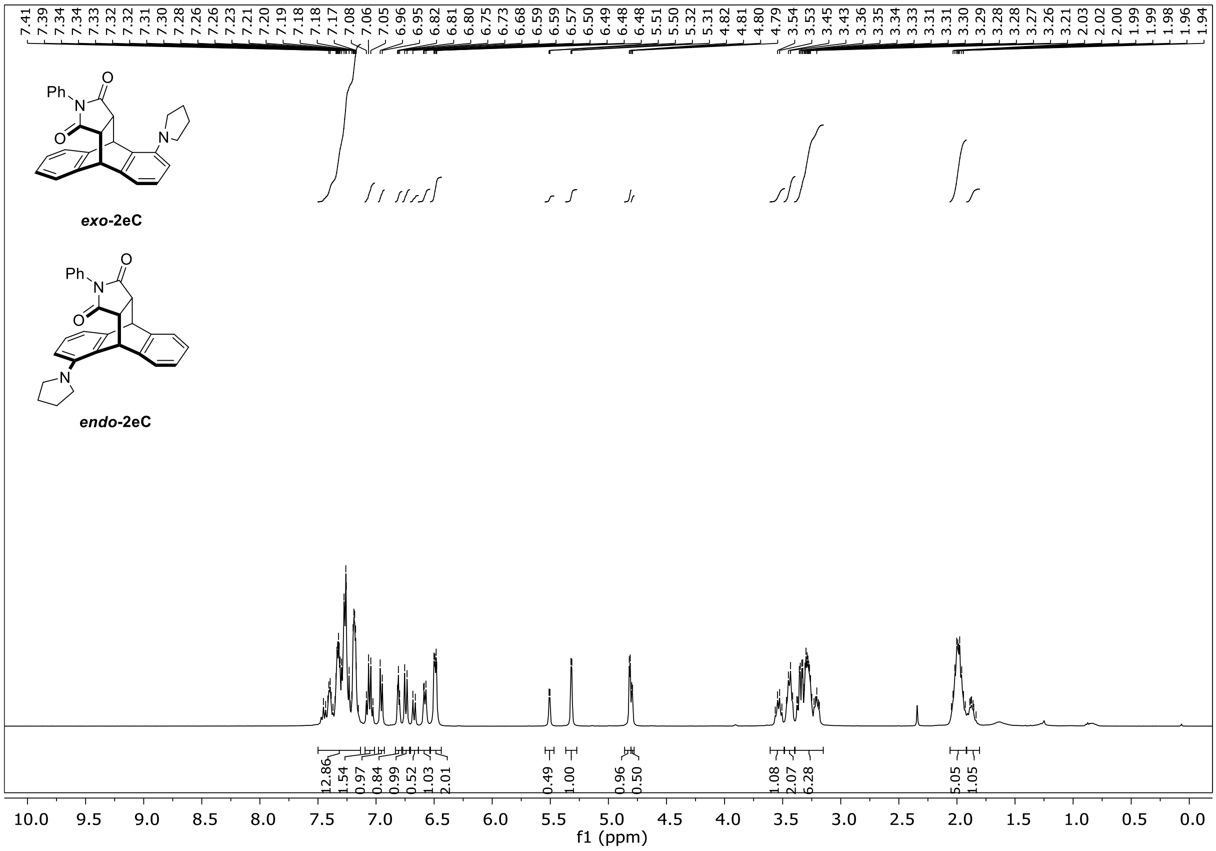

Supplementary Figure 60. ^{1}H NMR of ***exo/endo*-2eC** (400 MHz, $CDCl_3$)

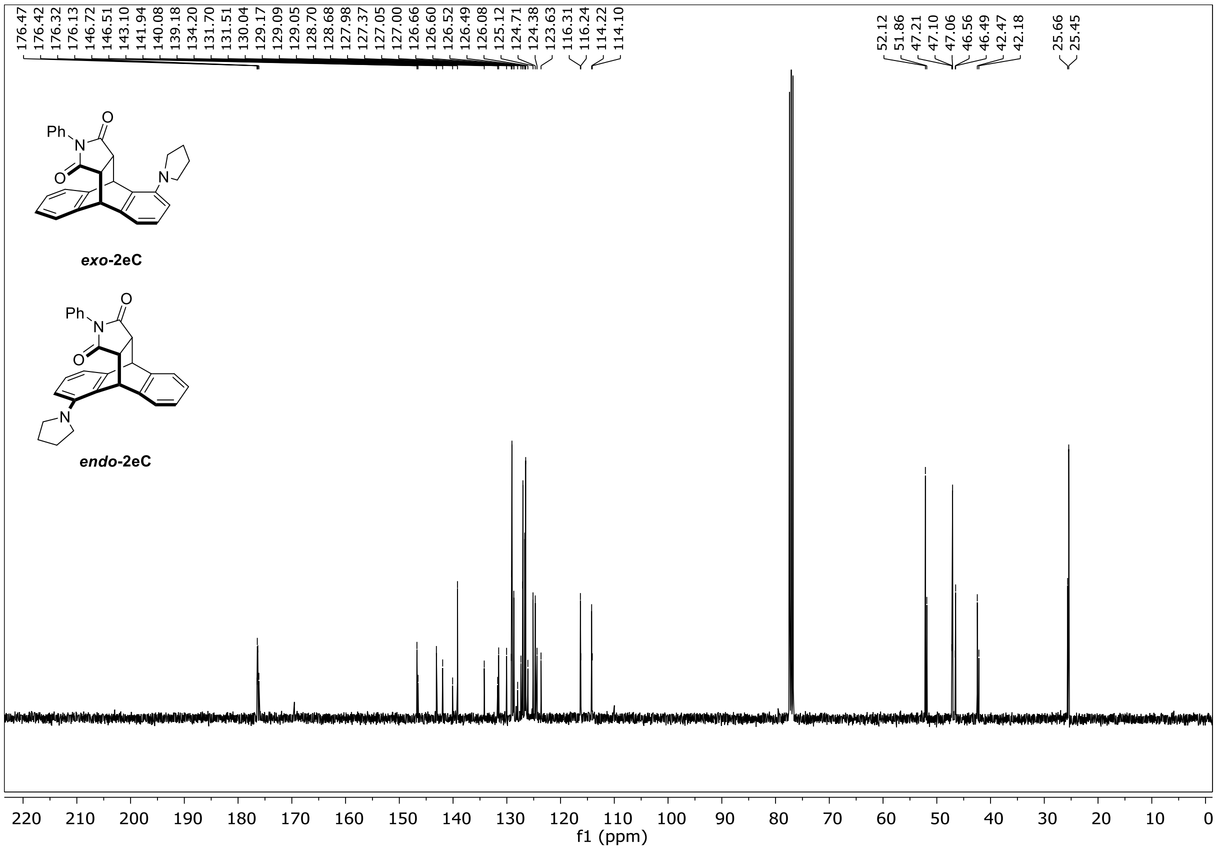

Supplementary Figure 61. ^{13}C NMR of ***exo/endo*-2eC** (101 MHz, $CDCl_3$)

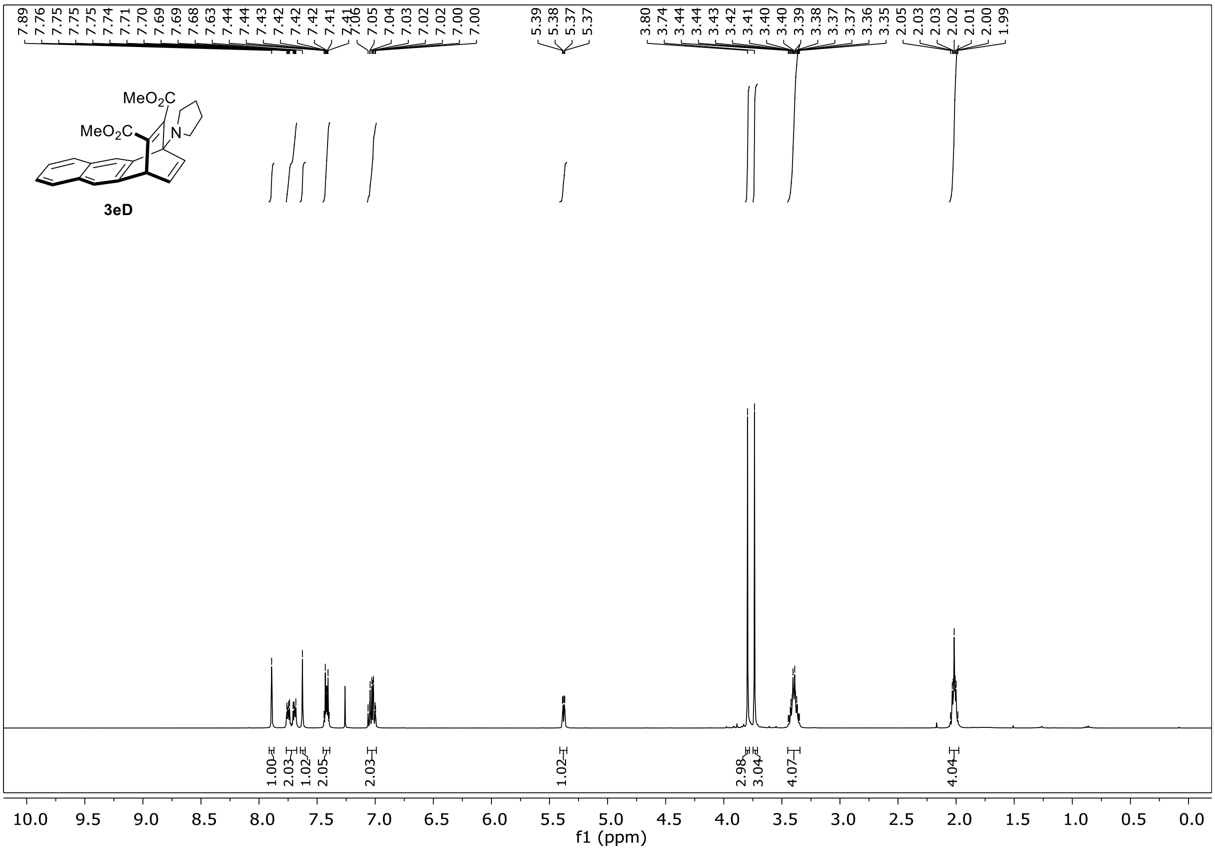

Supplementary Figure 62. ^{1}H NMR of **3eD** (400 MHz, $CDCl_3$)

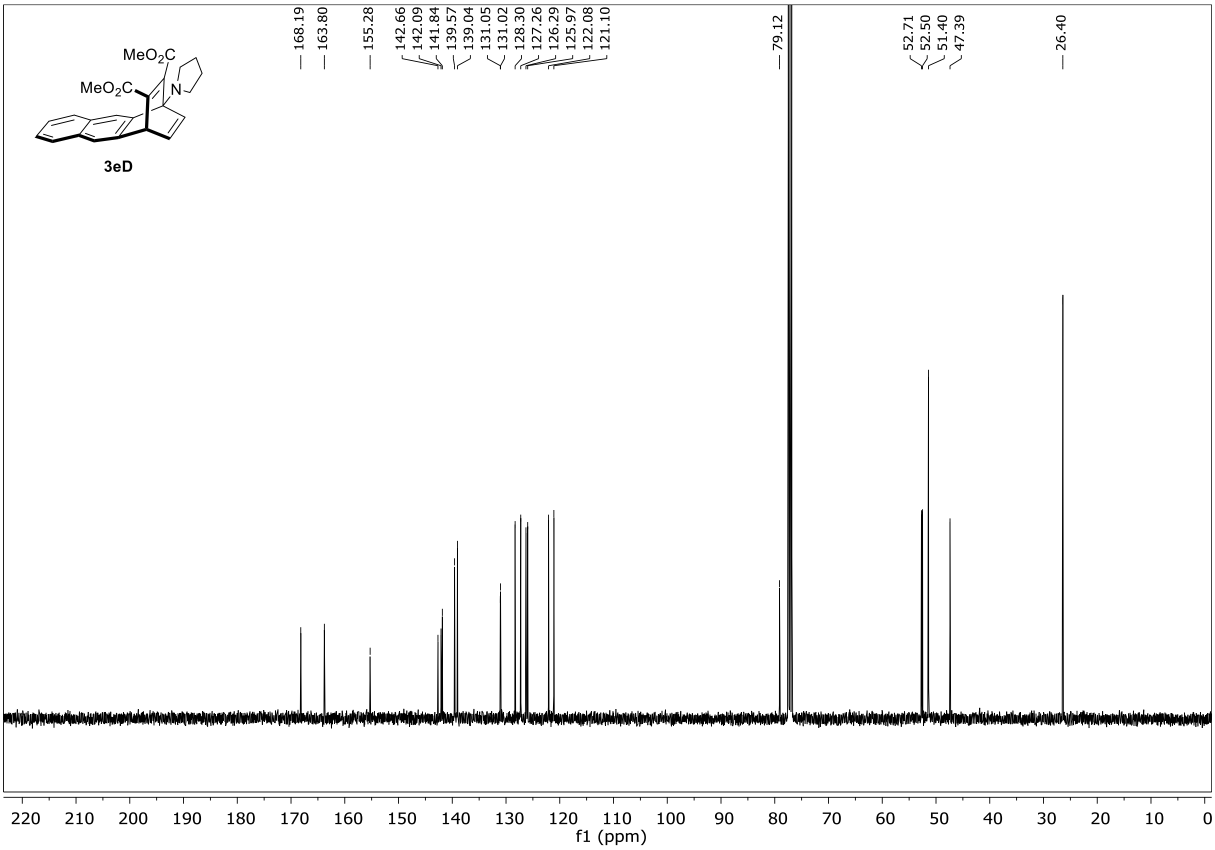

Supplementary Figure 63. ^{13}C NMR of **3eD** (101 MHz, $CDCl_3$)

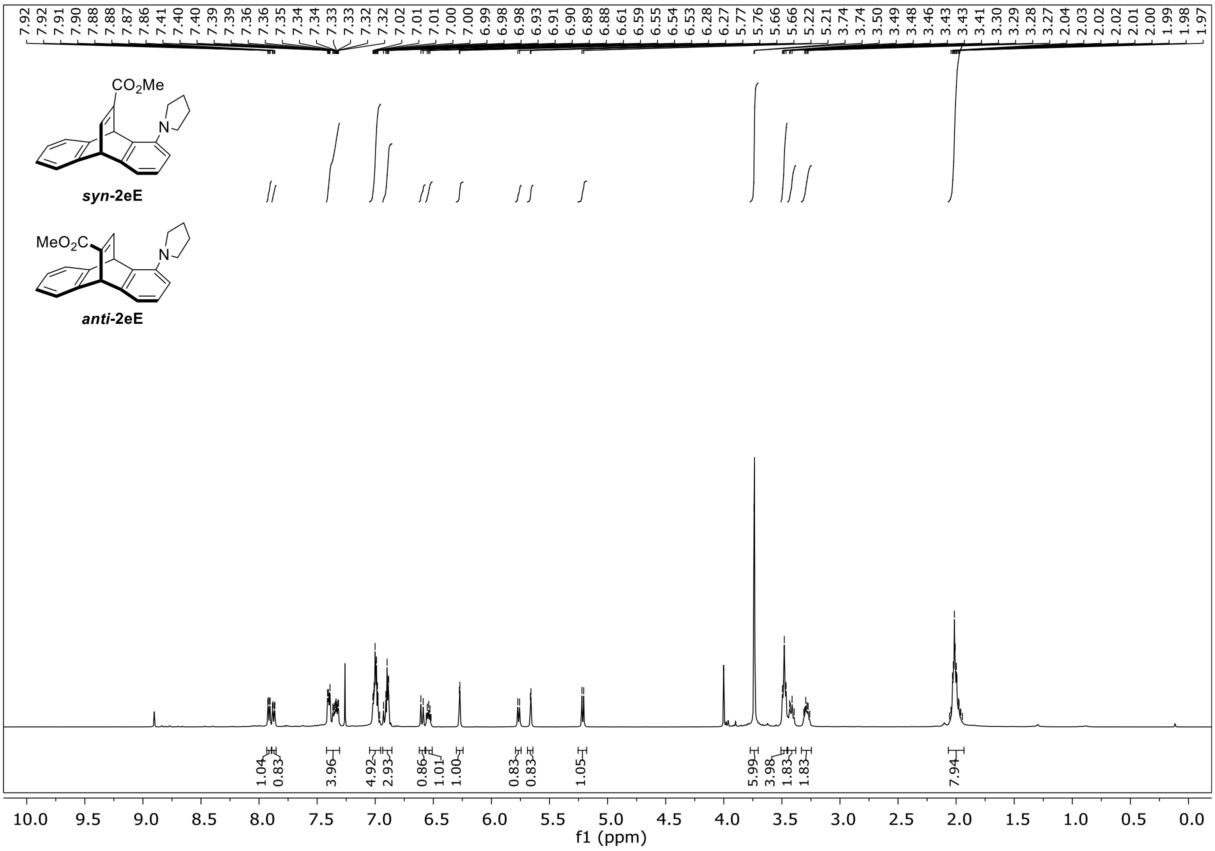

Supplementary Figure 64. ^{1}H NMR of ***anti*-2eE** (400 MHz, $CDCl_3$)

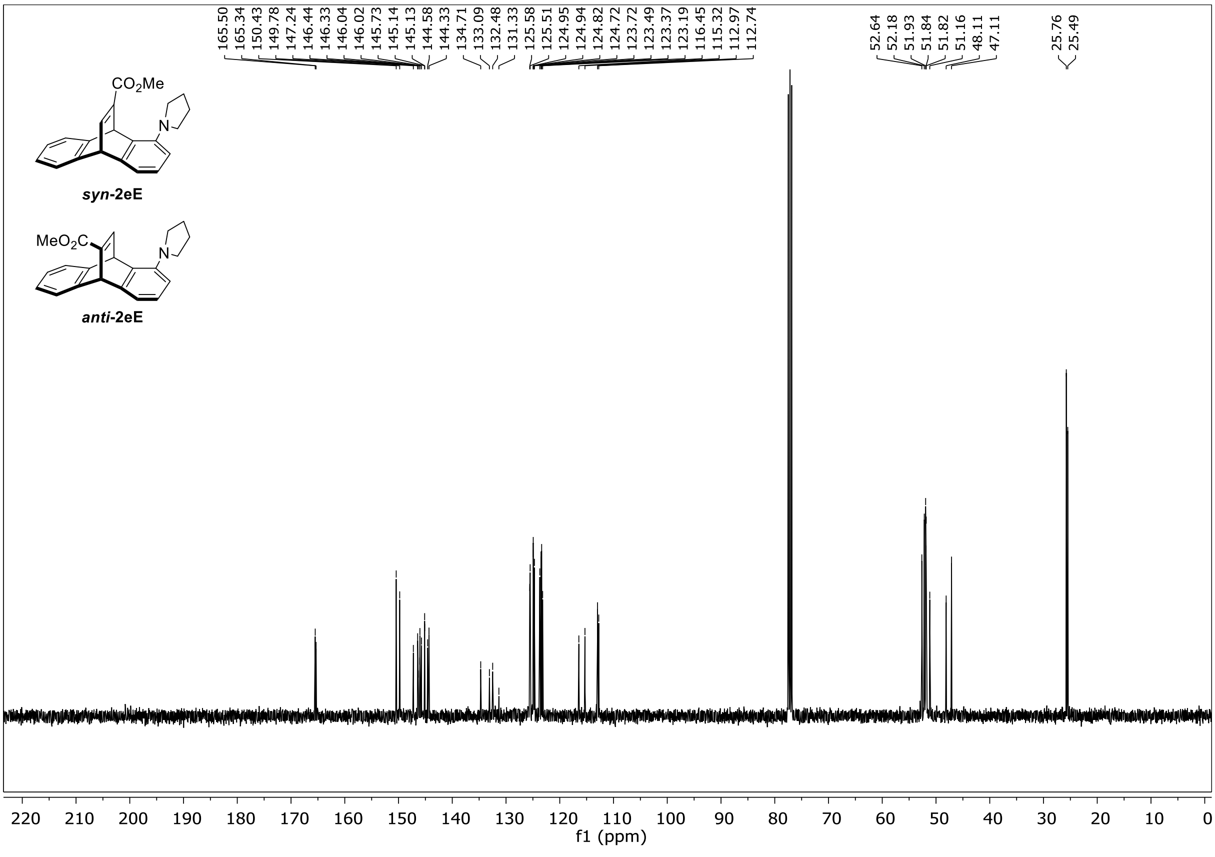

Supplementary Figure 65. ^{13}C NMR of ***anti*-2eE** (101 MHz, $CDCl_3$)

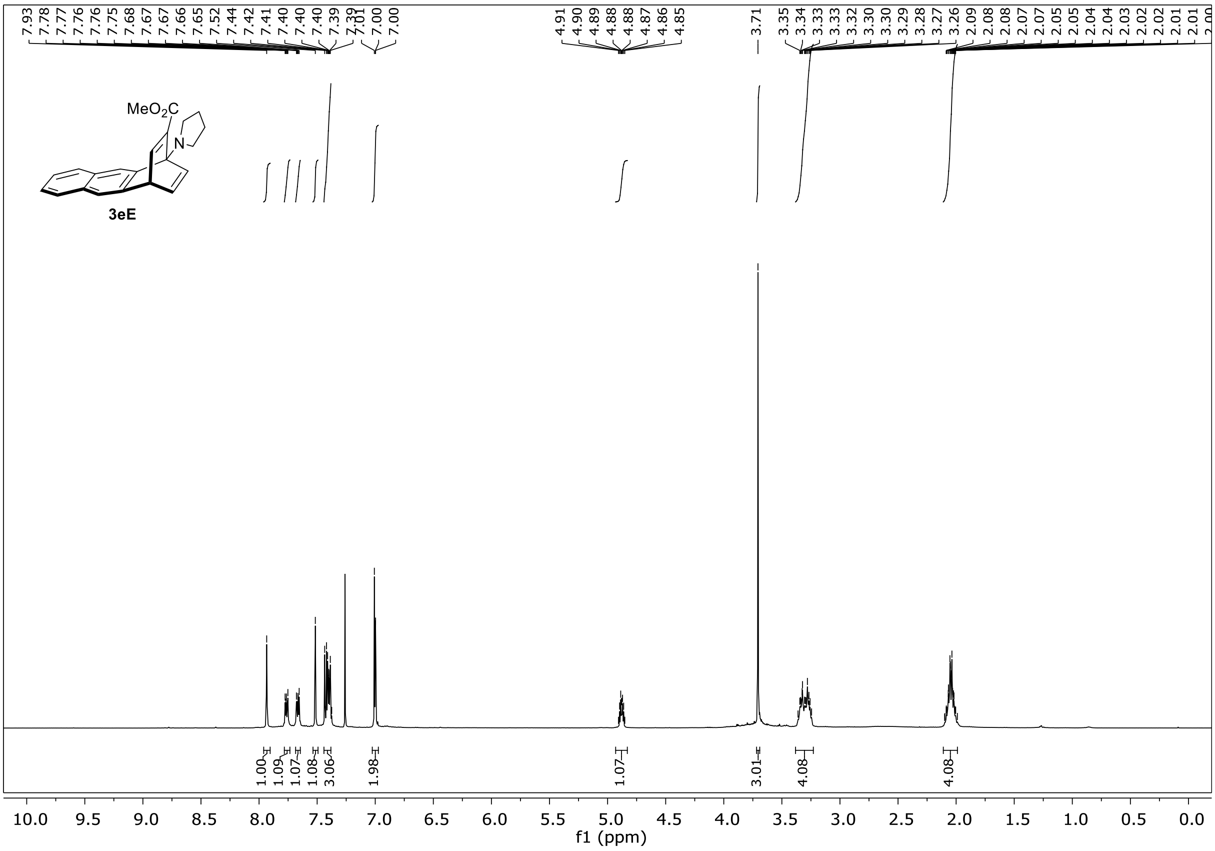

Supplementary Figure 66. ^{1}H NMR of **3eE** (300 MHz, $CDCl_3$)

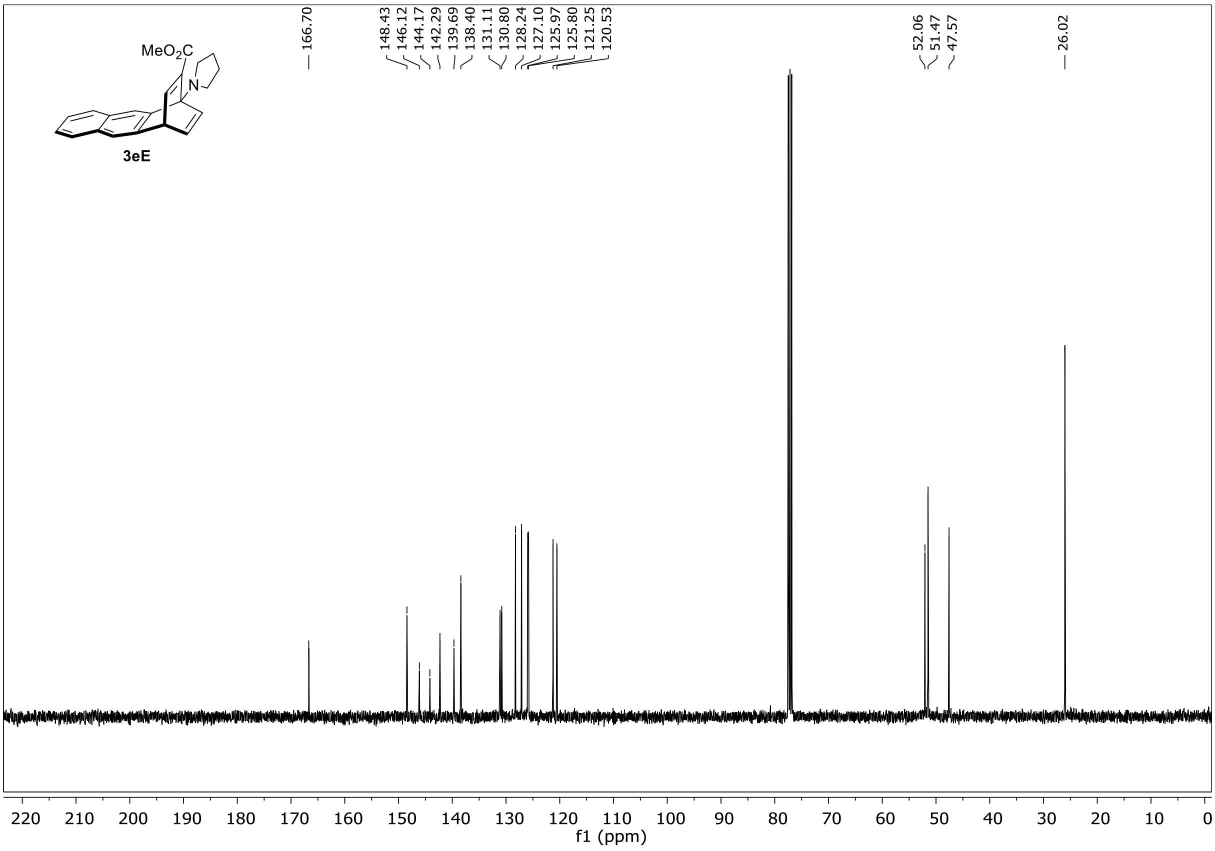

Supplementary Figure 67. ^{13}C NMR of **3eE** (101 MHz, $CDCl_3$)

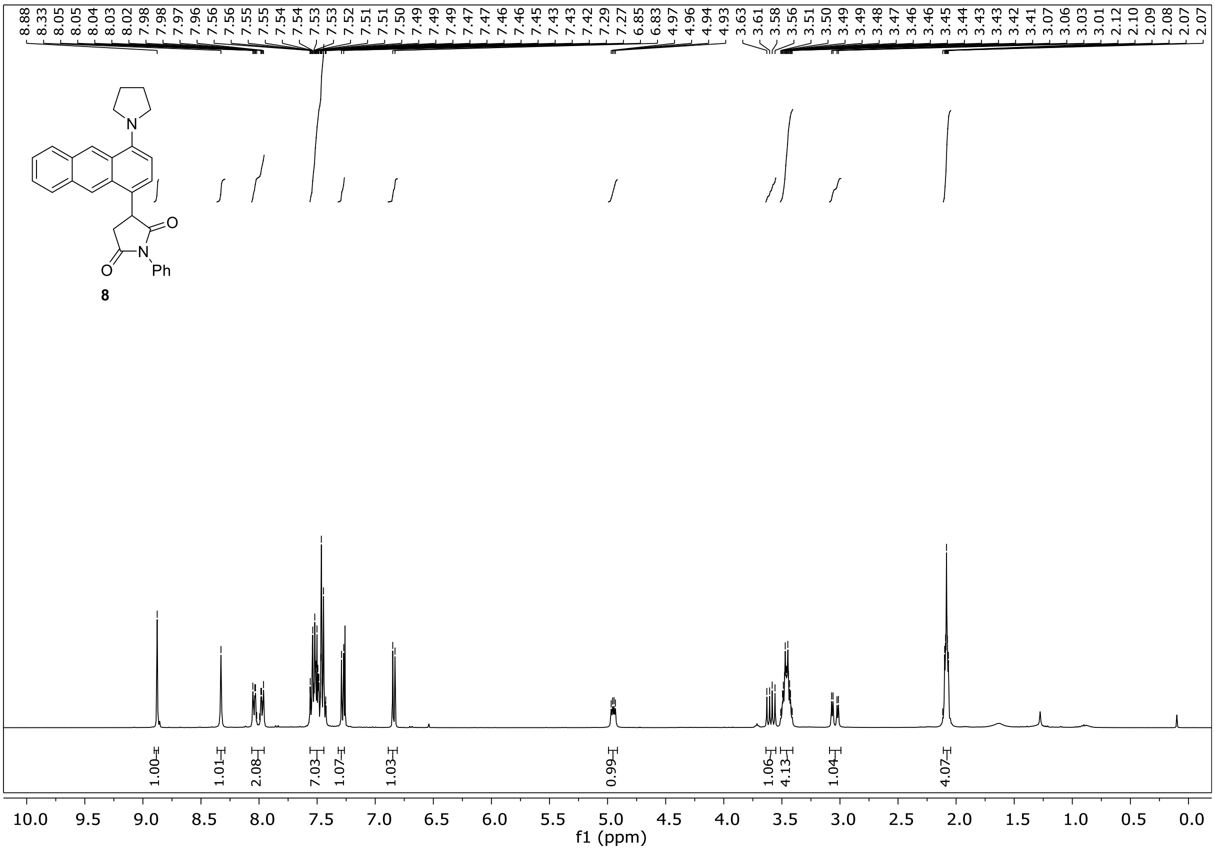

Supplementary Figure 68. ^{1}H NMR of **8** (400 MHz, $CDCl_3$)

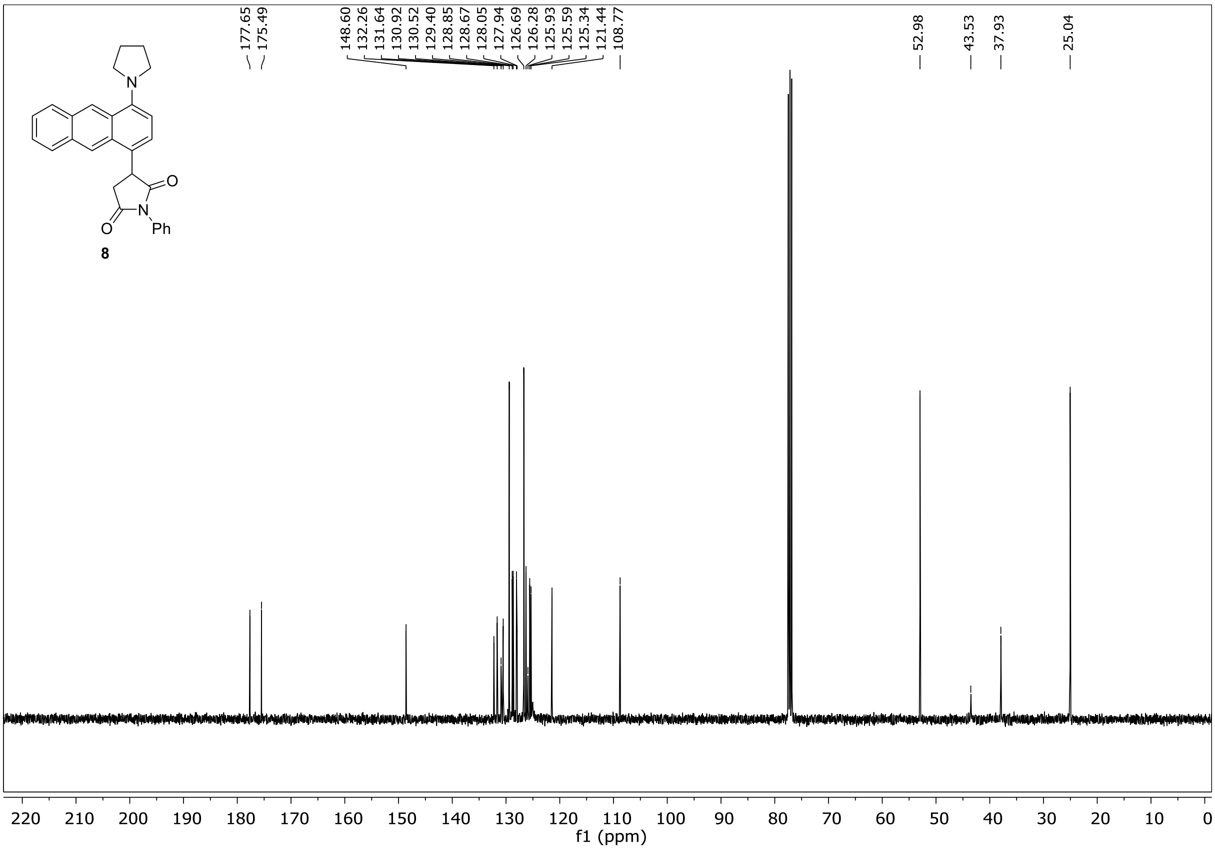

Supplementary Figure 69. ^{13}C NMR of **8** (101 MHz, $CDCl_3$)

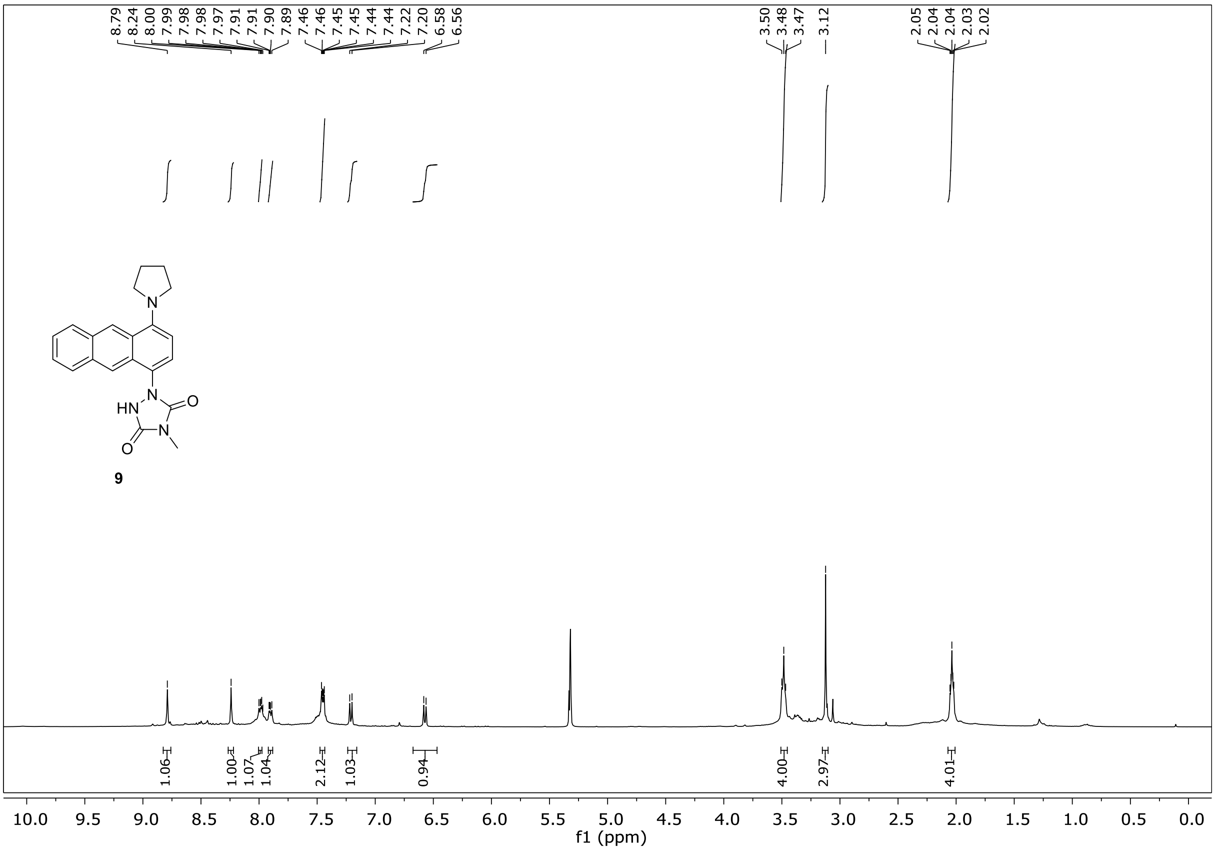

Supplementary Figure 70. ^{1}H NMR of **9** (400 MHz, $CDCl_3$)

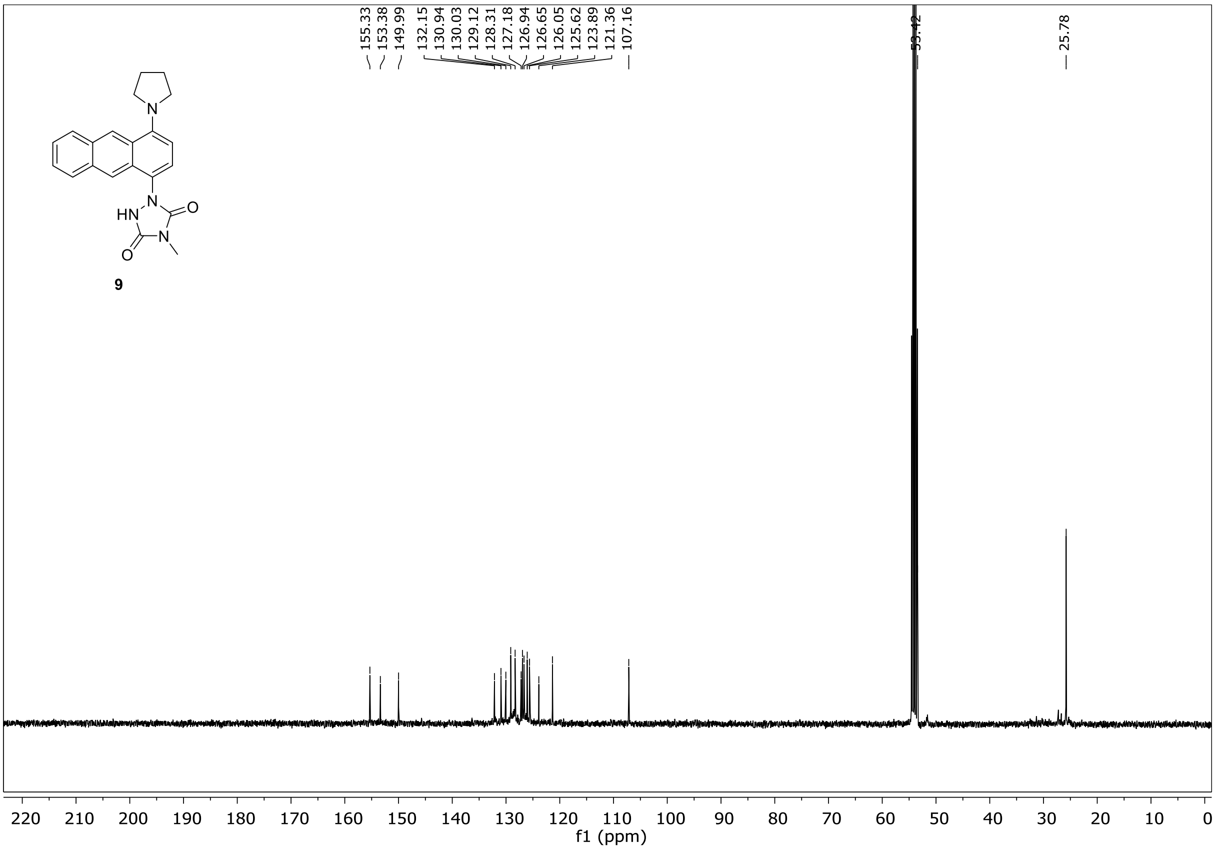

Supplementary Figure 71. ^{13}C NMR of **9** (101 MHz, $CDCl_3$)

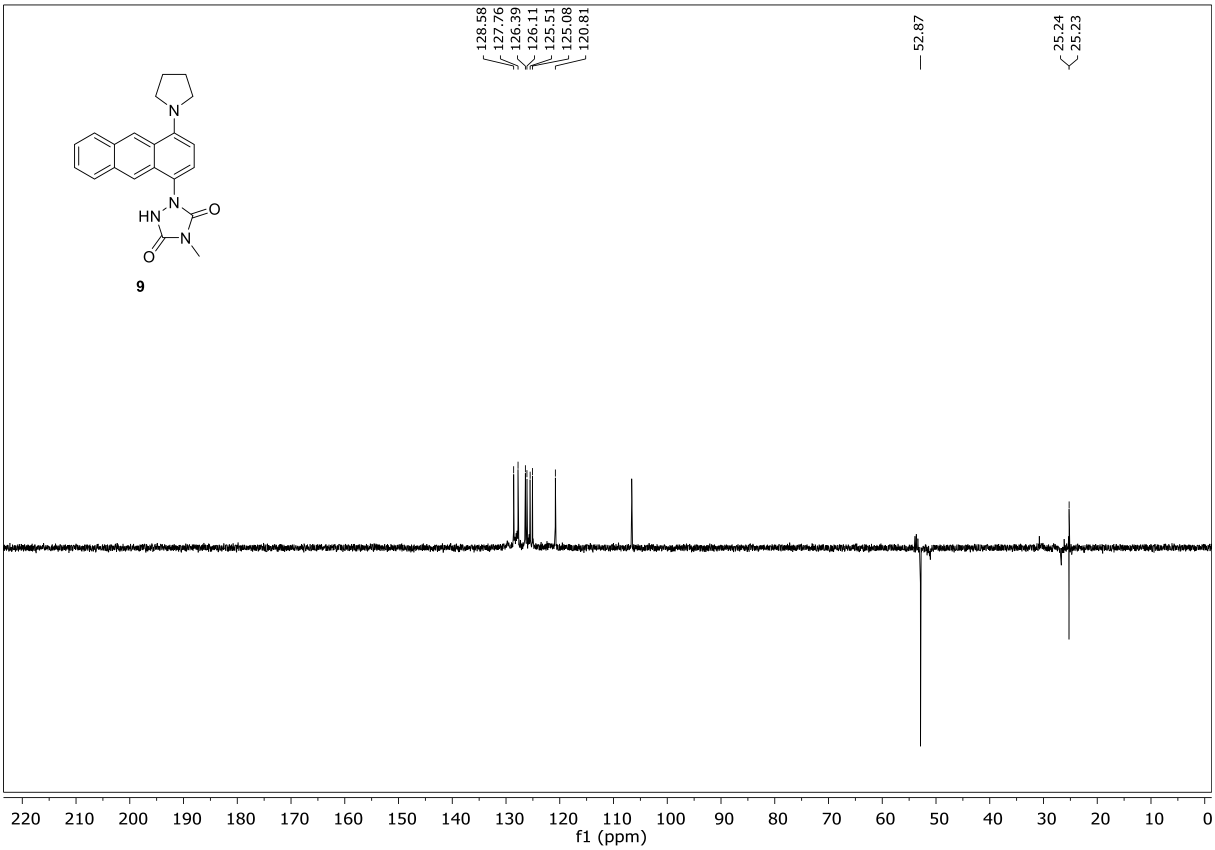

Supplementary Figure 72. DEPT-135 of **9** (101 MHz, $CDCl_3$)

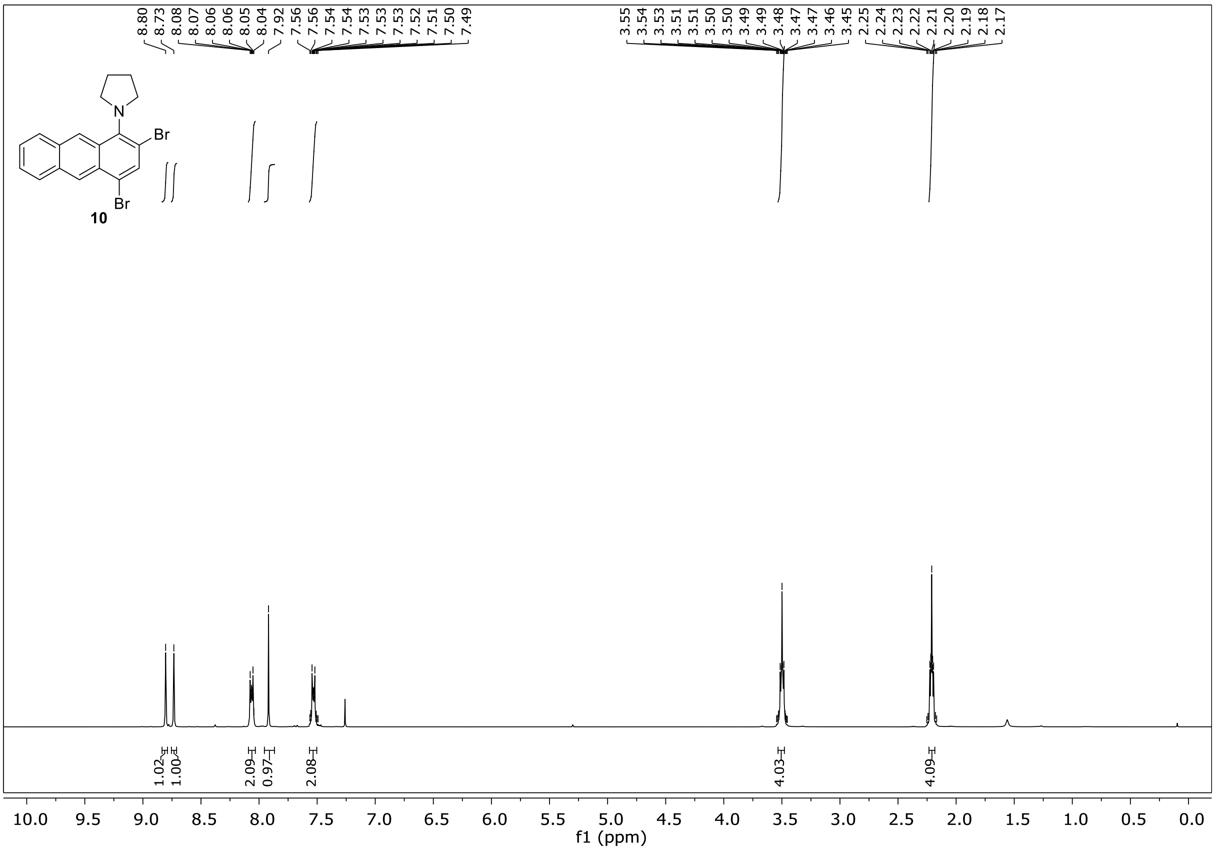

Supplementary Figure 73. ^{1}H NMR of **10** (400 MHz, $CDCl_3$)

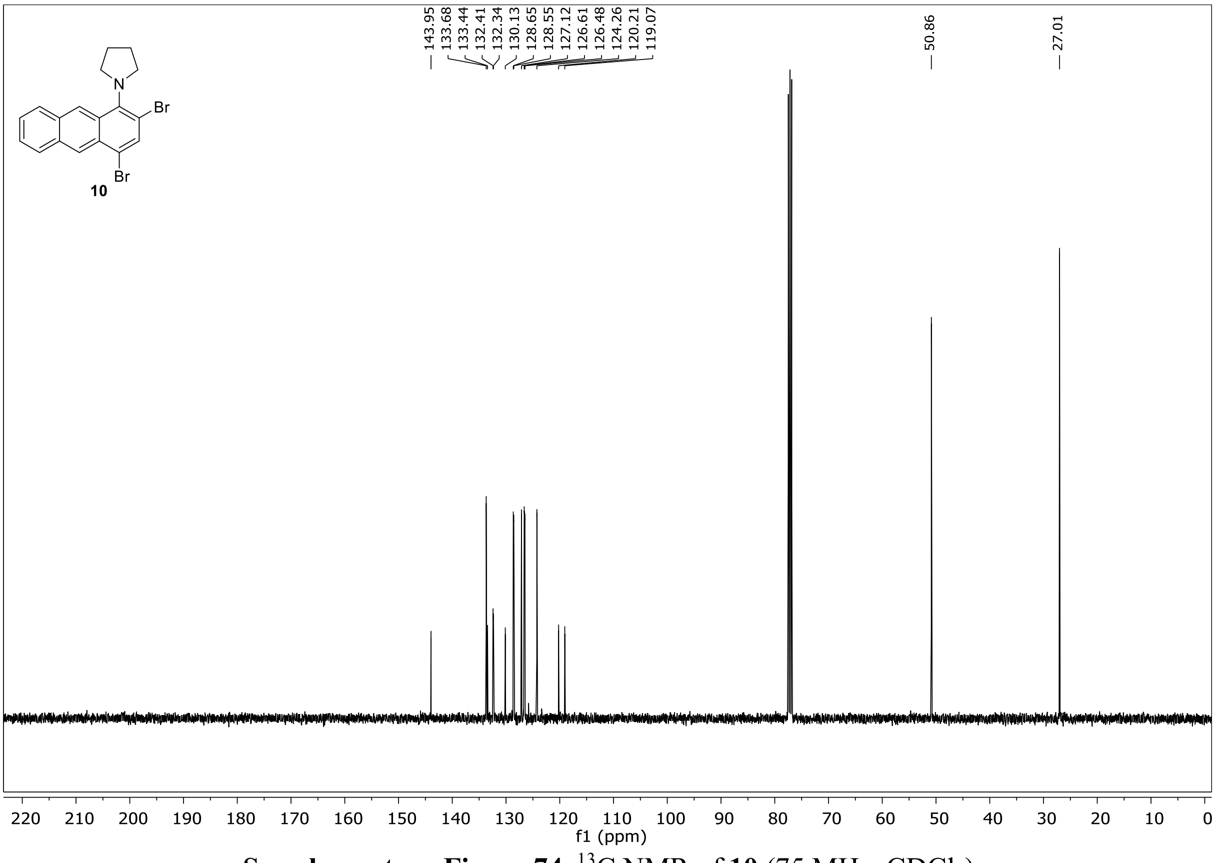

Supplementary Figure 74. ^{13}C NMR of **10** (75 MHz, $CDCl_3$)

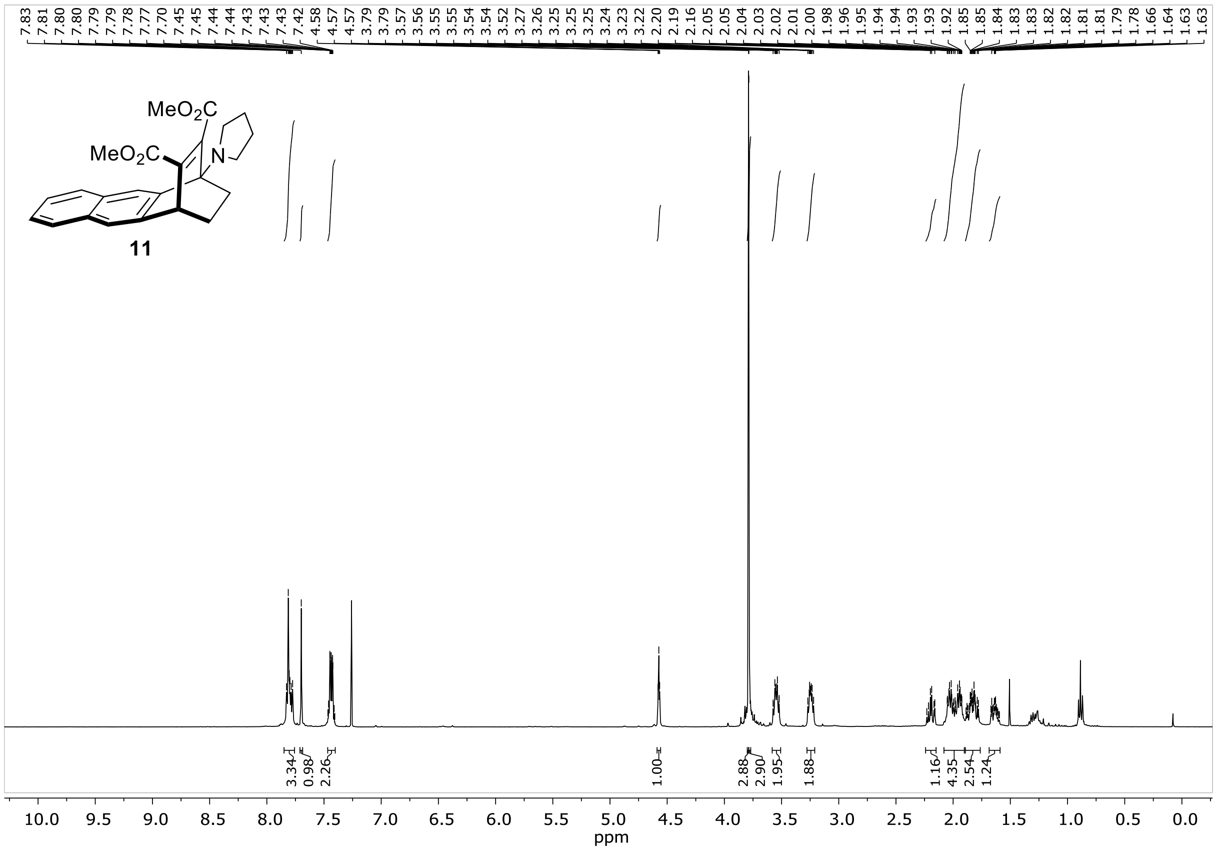

Supplementary Figure 75. ^{1}H NMR of **11** (400 MHz, $CDCl_3$)

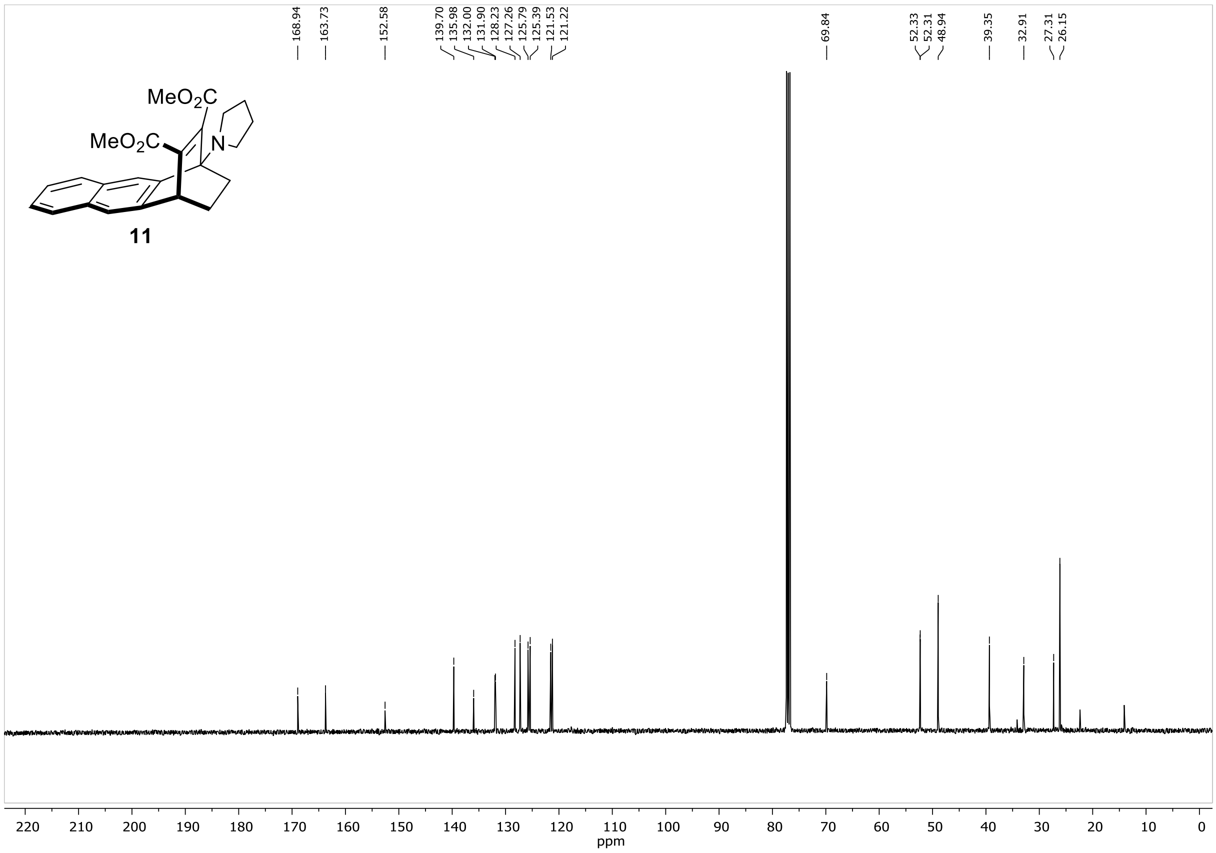

Supplementary Figure 76. ^{13}C NMR of **11** (101 MHz, $CDCl_3$)

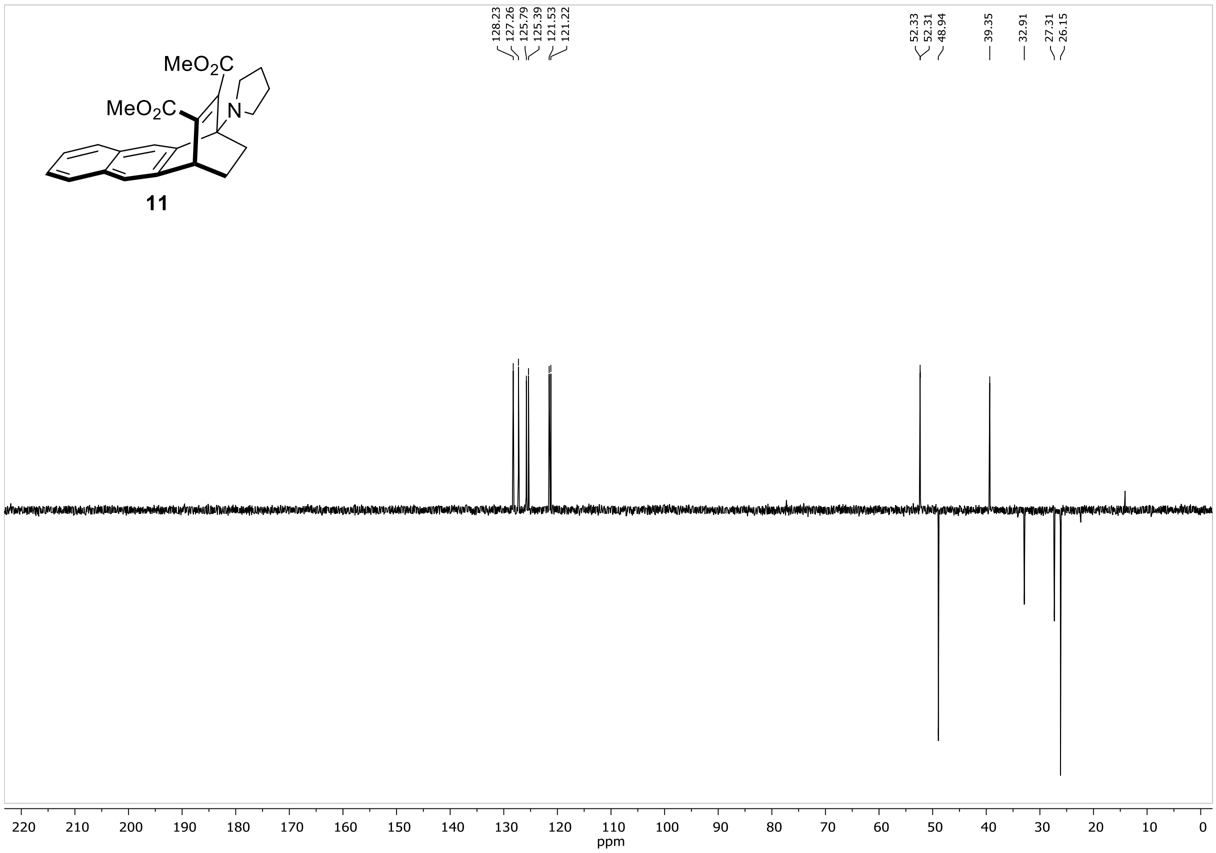

Supplementary Figure 77. DEPT-135 of **11** (101 MHz, $CDCl_3$)

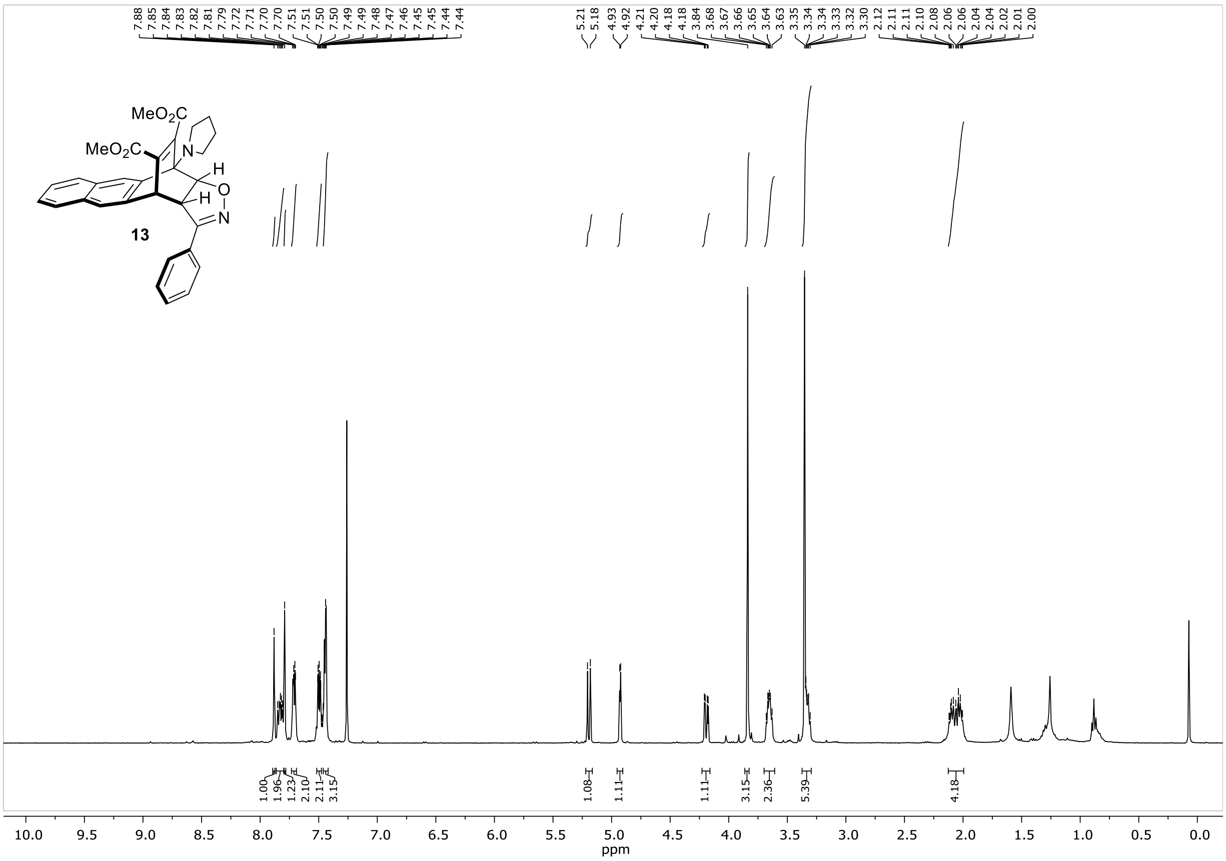

Supplementary Figure 78. ^{1}H NMR of **13** (major isomer, 400 MHz, $CDCl_3$)

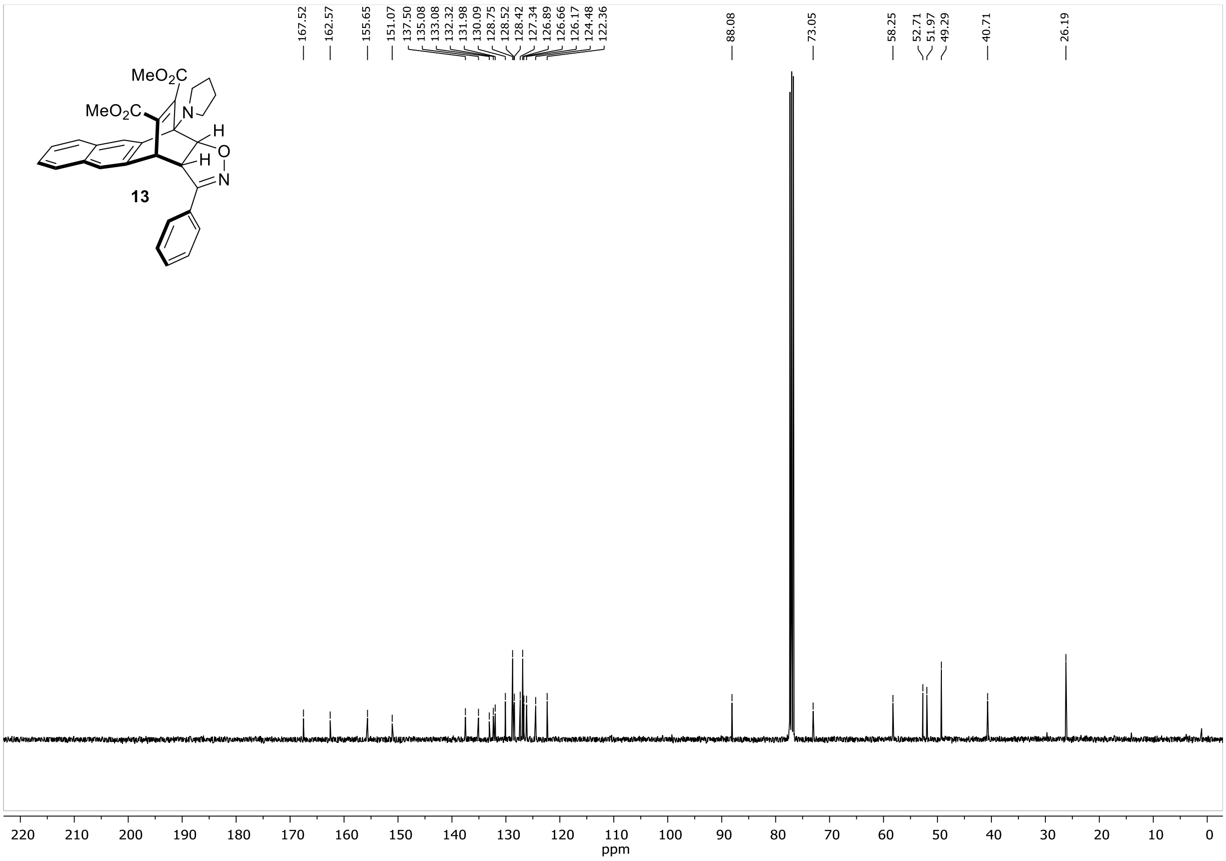

Supplementary Figure 79. ^{13}C NMR of **13** (101 MHz, $CDCl_3$)

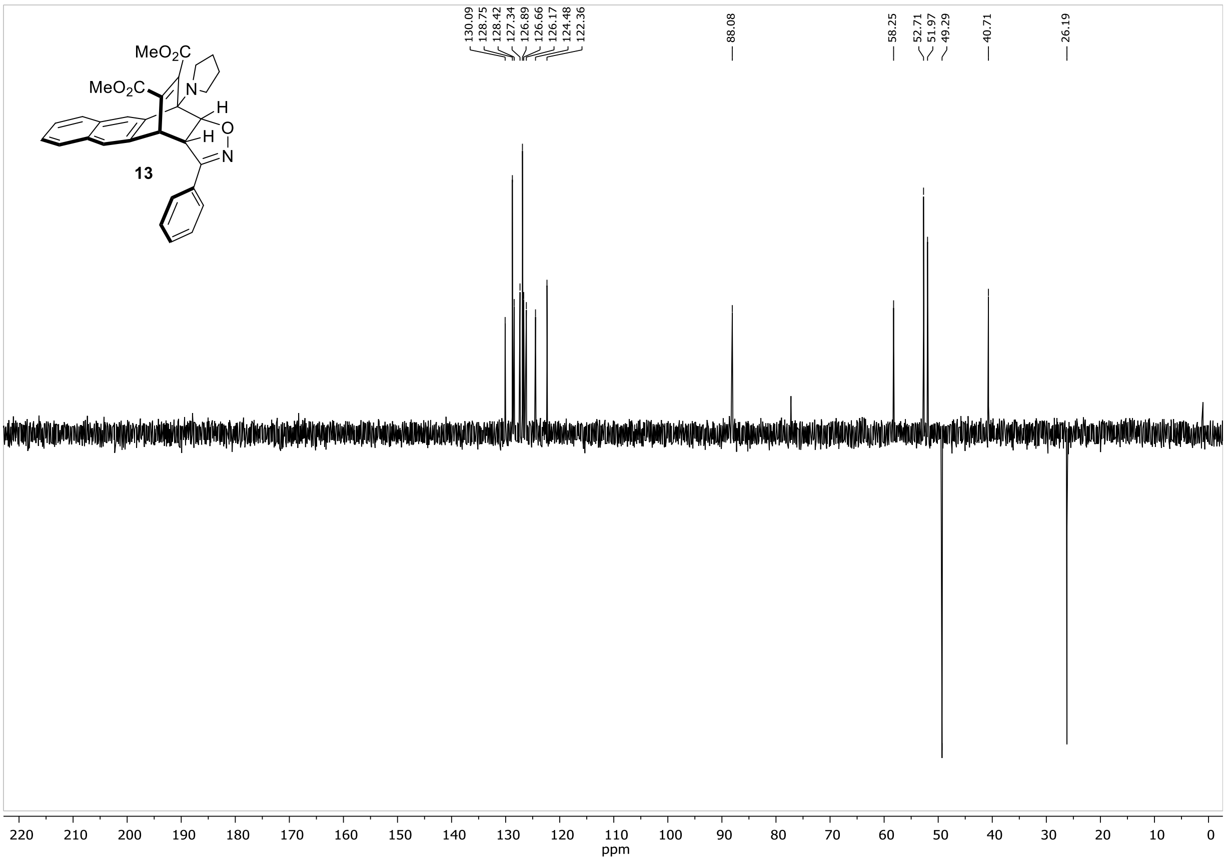

Supplementary Figure 80. DEPT-135 of **13** (101 MHz, $CDCl_3$)

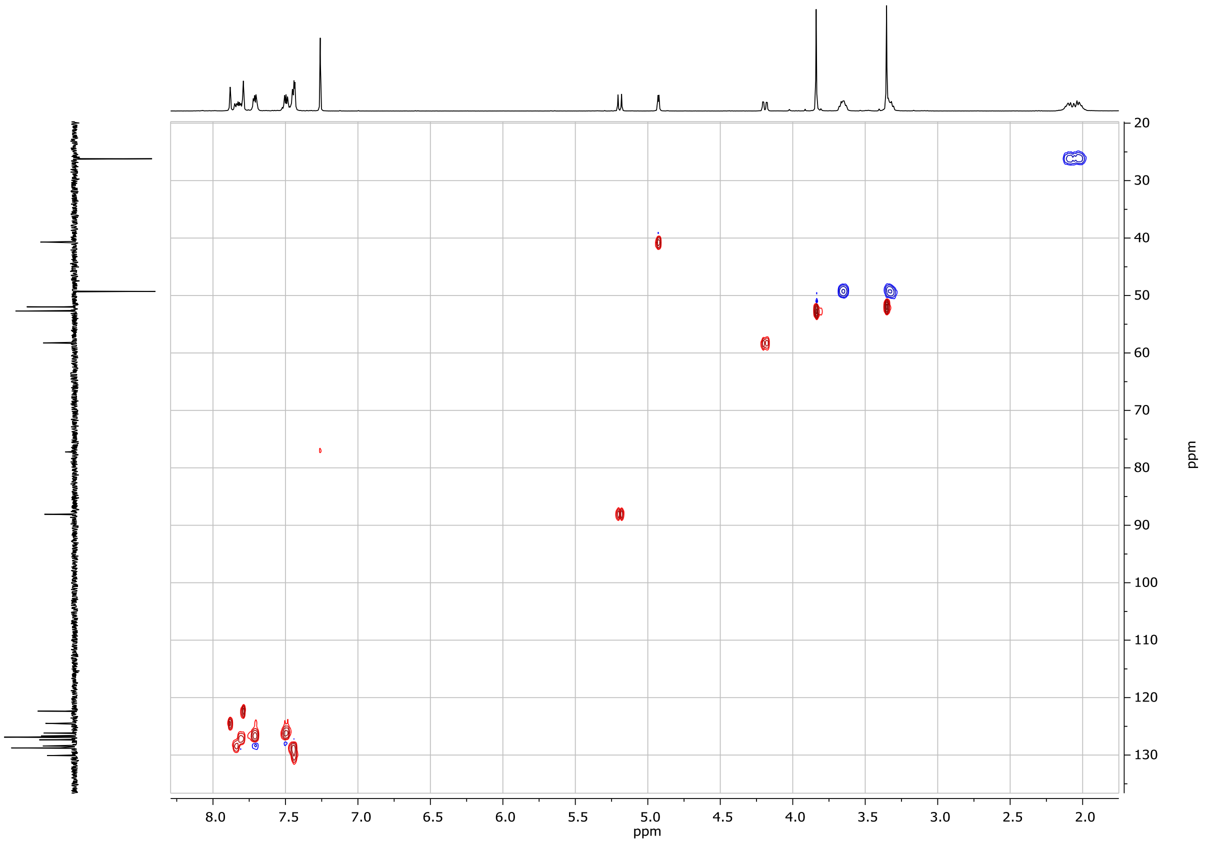

Supplementary Figure 81. HSQC of **13** (400 MHz, $CDCl_3$)

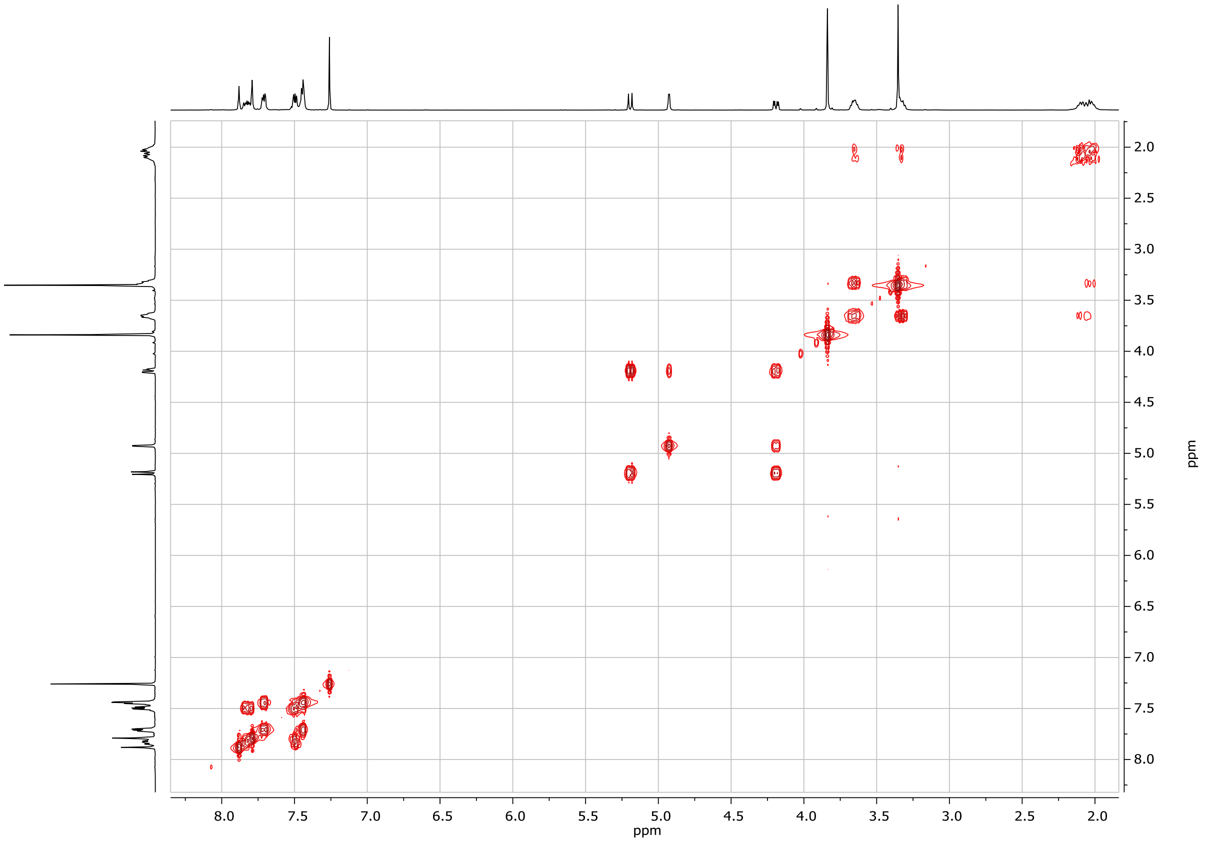

Supplementary Figure 82. COSY of **13** (400 MHz, $CDCl_3$)

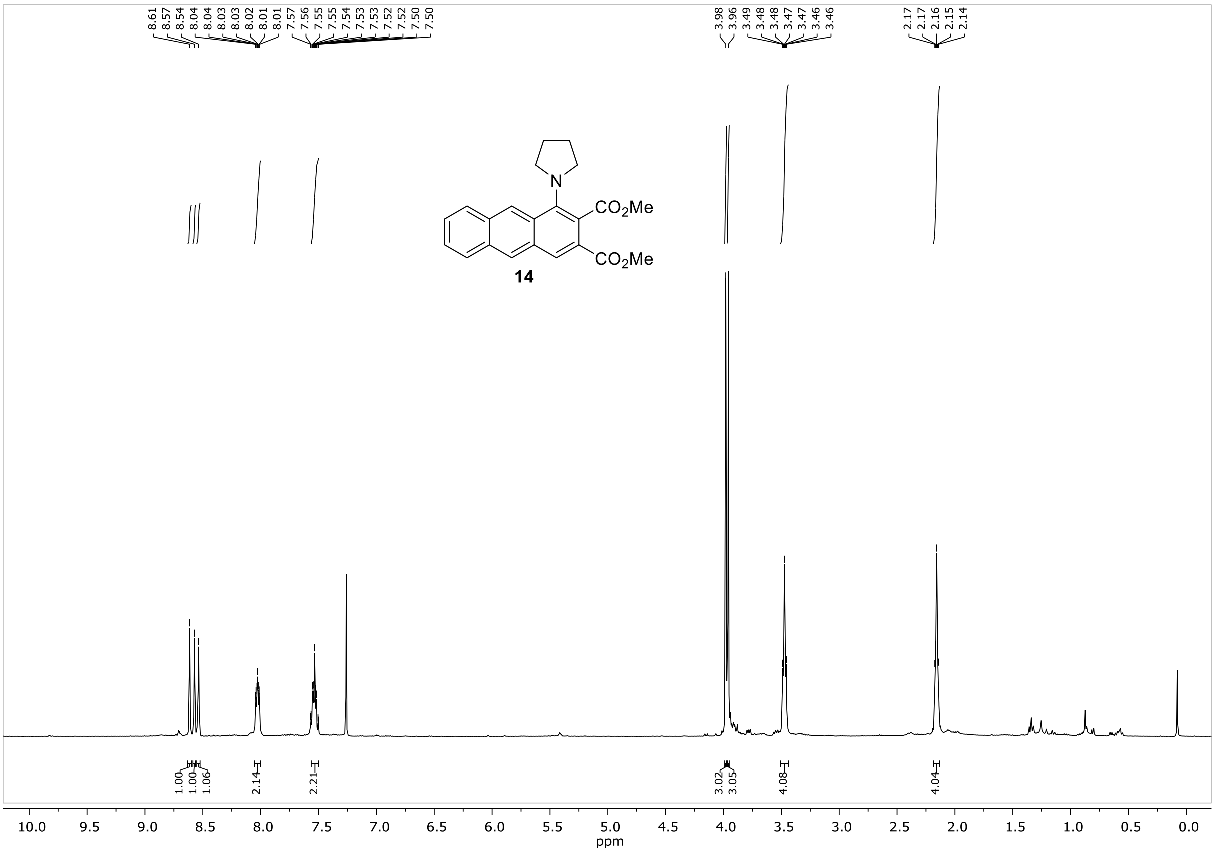

Supplementary Figure 83. ^{1}H NMR of **14** (400 MHz, $CDCl_3$)

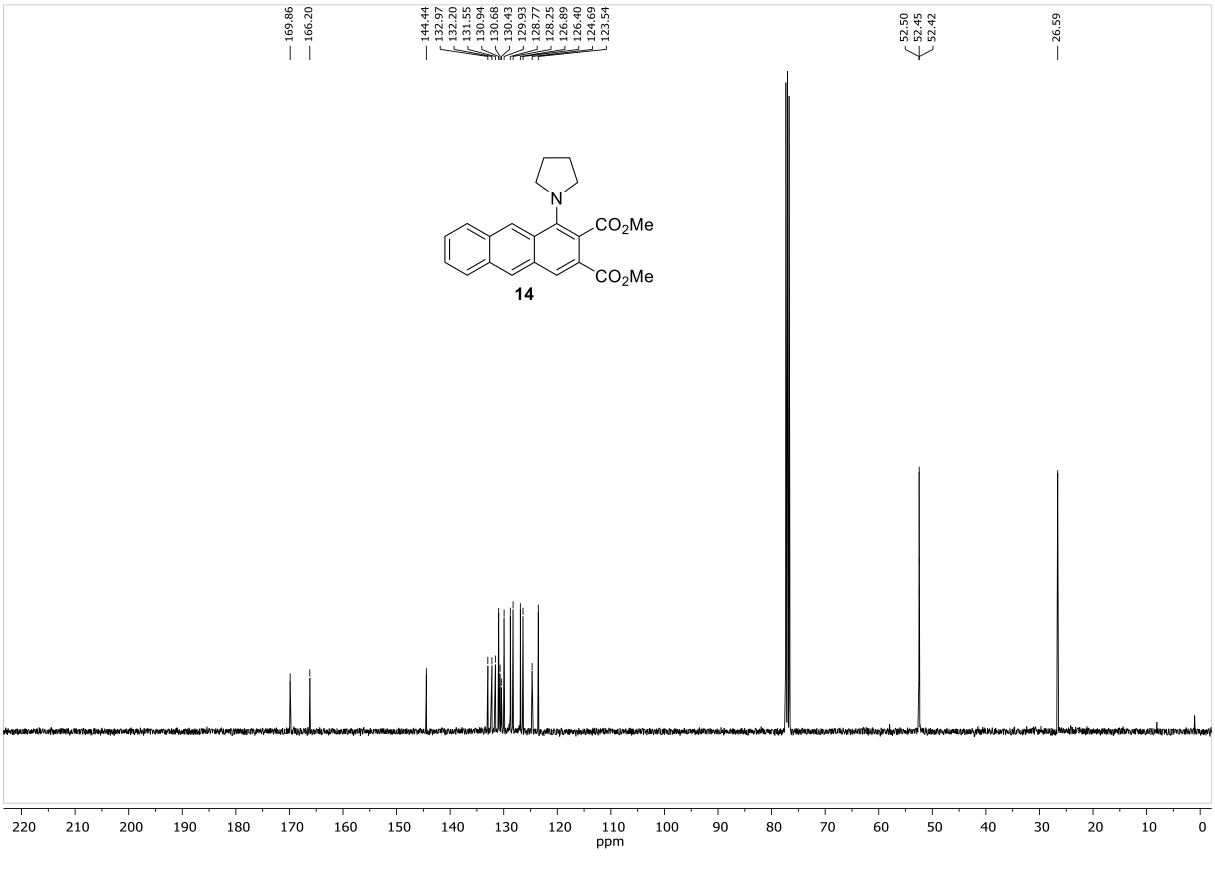

Supplementary Figure 84. ^{13}C NMR of **14** (101 MHz, $CDCl_3$)

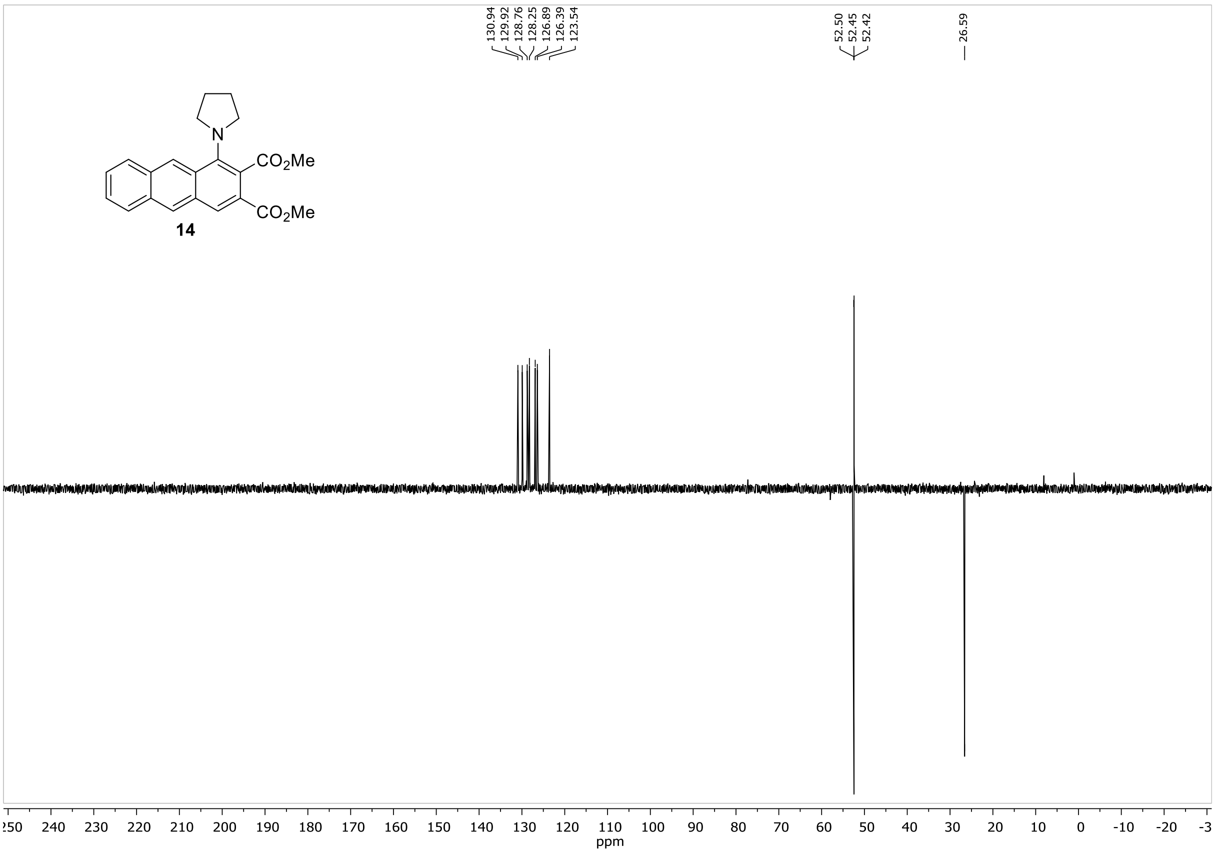

Supplementary Figure 85. DEPT-135 of **14** (101 MHz, $CDCl_3$)

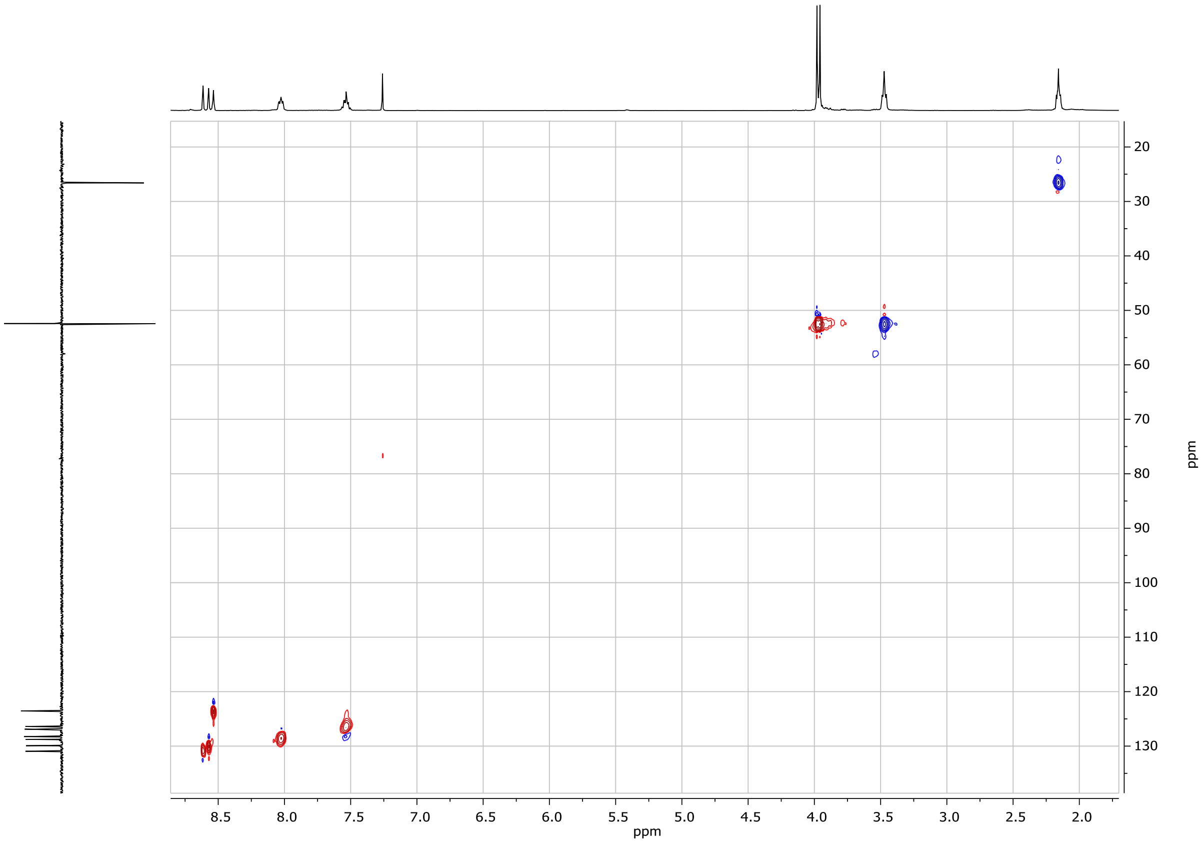

Supplementary Figure 86. HSQC of **14** (400 MHz, $CDCl_3$)

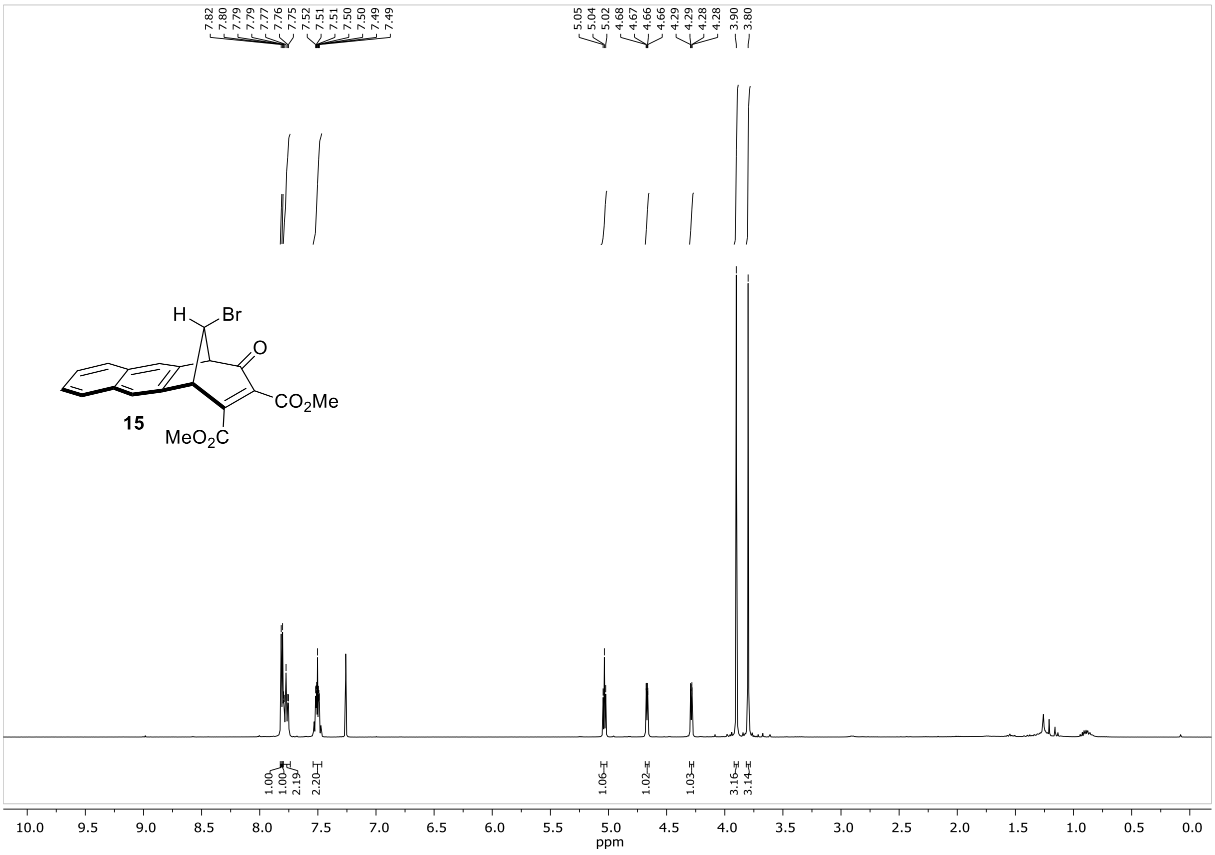

Supplementary Figure 87. ^{1}H NMR of **15** (400 MHz, $CDCl_3$)

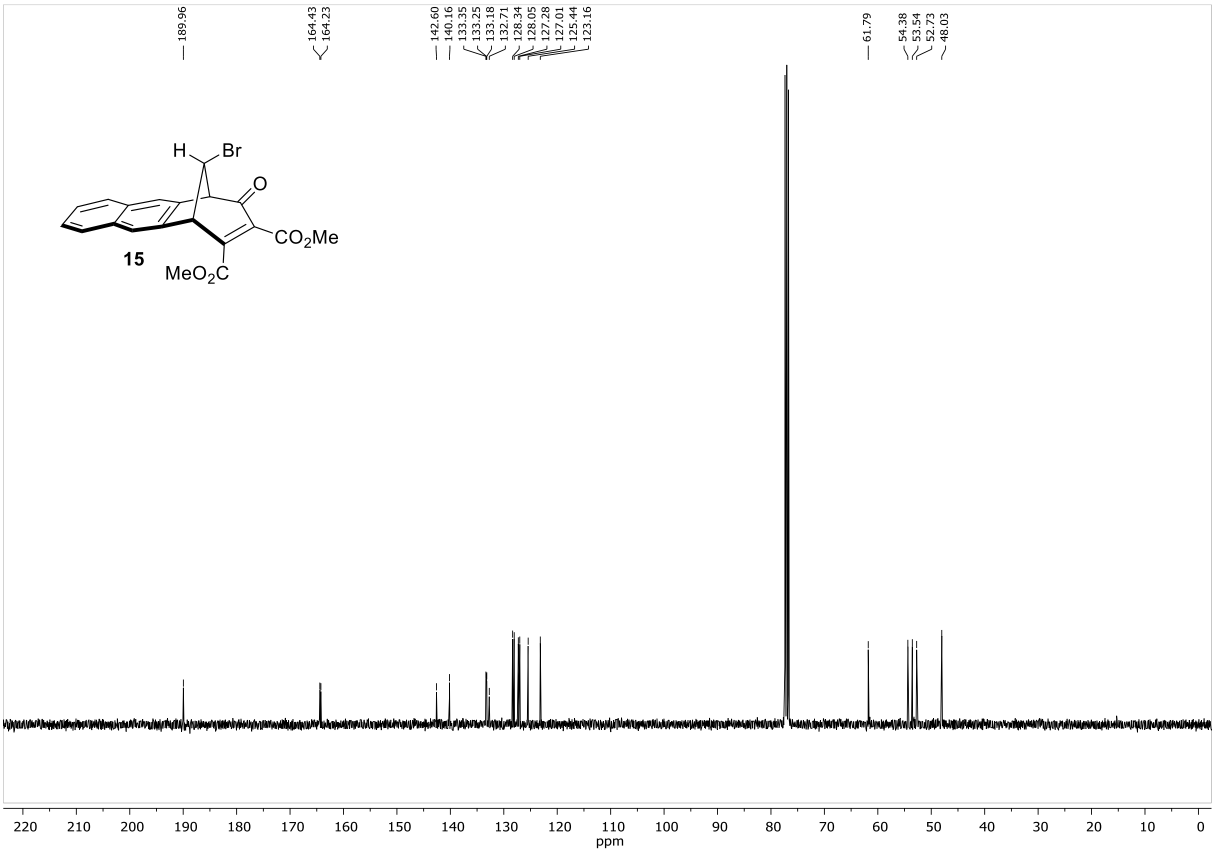

Supplementary Figure 88. ^{13}C NMR of **15** (101 MHz, $CDCl_3$)

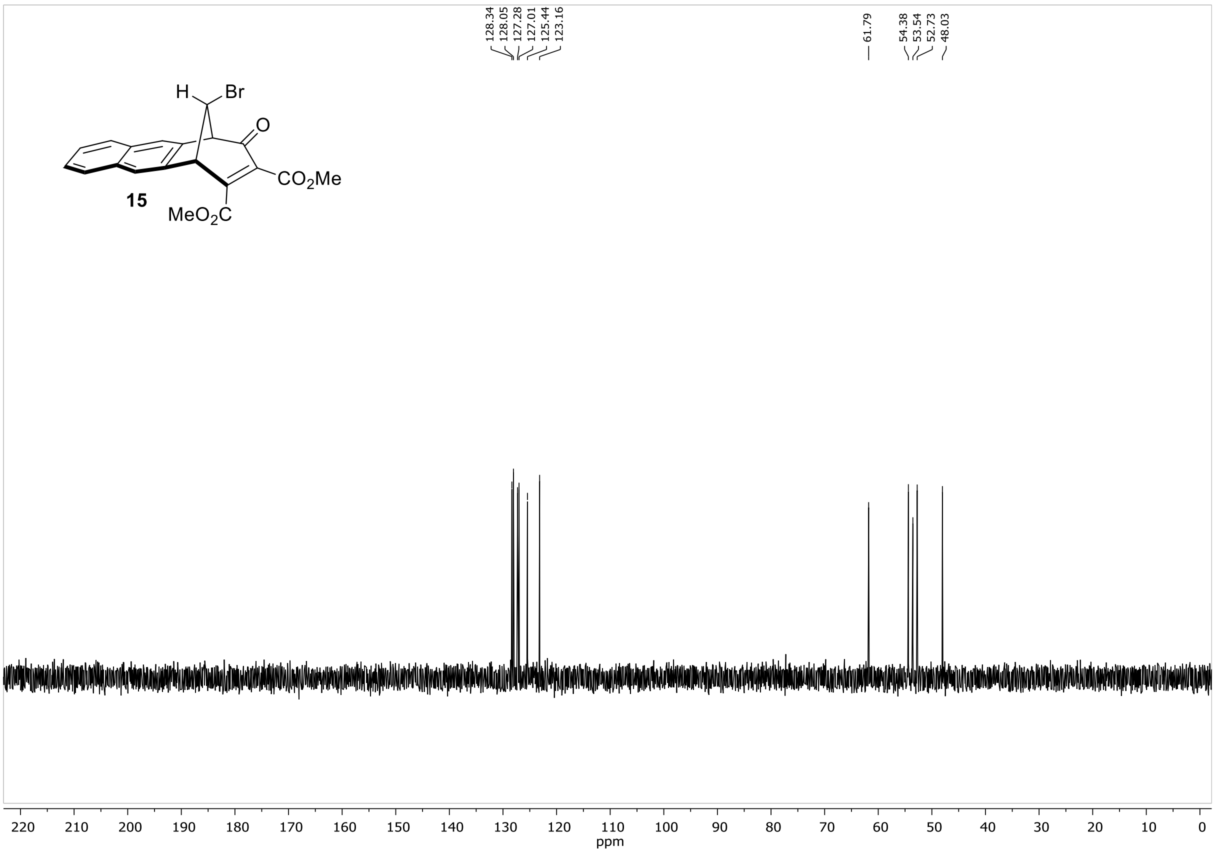

Supplementary Figure 89. DEPT-135 of **15** (101 MHz, $CDCl_3$)

6. X-Ray Crystallographic Studies

Dimethyl 1,5-dimethoxy-1,4-dihydro-1,4-ethenoanthracene-2,3-dicarboxylate (3bD)

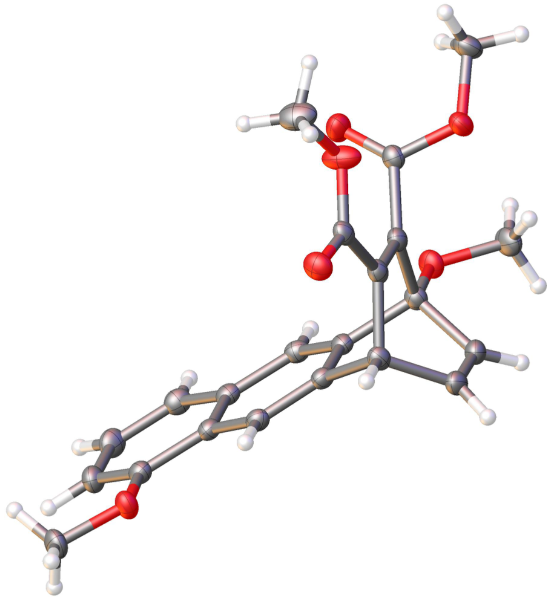

**Compound**	**N095**
CCDC	1979830
Formula	$C_{22}H_{20}O_6$
$D_{calc.}$/ g cm^{-3}	1.388
μ/mm^{-1}	0.838
Formula Weight	380.38
Colour	yellowish colourless
Shape	block
Size/mm^3	0.26×0.11×0.07
T/K	124(1)
Crystal System	triclinic
Space Group	P-1
a/Å	9.0753(2)
b/Å	9.76091(19)
c/Å	10.8610(2)
α/°	91.7335(16)
β/°	108.5566(19)
γ/°	92.3162(17)
V/Å^3	910.40(3)
Z	2
Z'	1
Wavelength/Å	1.54184
Radiation type	Cu K$_\alpha$
Θ_{min}/°	4.298
Θ_{max}/°	66.030
Measured Refl's.	10461
Ind't Refl's	3154
Refl's with I > 2(I)	2892
R_{int}	0.0202
Parameters	257
Restraints	0
Largest Peak	0.278
Deepest Hole	-0.250
GooF	1.038
wR_2 (all data)	0.1032
wR_2	0.1004
R_1 (all data)	0.0399
R_1	0.0372

Dimethyl 1,5-bis(dimethylamino)-1,4-dihydro-1,4-ethenoanthracene-2,3-dicarboxylate (3cD)

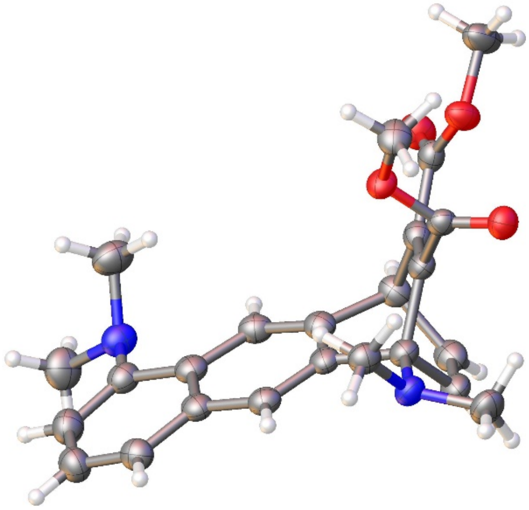

**Compound**	**L086**
CCDC	1979832
Formula	$C_{48}H_{52}N_4O_8$
$D_{calc.}$/ g cm^{-3}	1.309
μ/mm^{-1}	0.724
Formula Weight	812.93
Colour	light yellow
Shape	prism
Size/mm^3	0.08×0.05×0.04
T/K	123.00(10)
Crystal System	monoclinic
Space Group	$C2/c$
a/Å	19.2416(13)
b/Å	19.8451(8)
c/Å	12.2391(8)
α/°	90
β/°	117.999(9)
γ/°	90
V/Å^3	4126.5(5)
Z	4
Z'	0.5
Wavelength/Å	1.54184
Radiation type	Cu K_α
Θ_{min}/°	3.425
Θ_{max}/°	63.070
Measured Refl's.	9176
Ind't Refl's	3208
Refl's with I > 2(I)	2767
R_{int}	0.0235
Parameters	275
Restraints	0
Largest Peak	0.272
Deepest Hole	-0.197
GooF	1.048
wR_2 (all data)	0.1191
wR_2	0.1135
R_1 (all data)	0.0483
R_1	0.0413

Dimethyl 1,5-di(pyrrolidin-1-yl)-1,4-dihydro-1,4-ethenoanthracene-2,3-dicarboxylate (3dD)

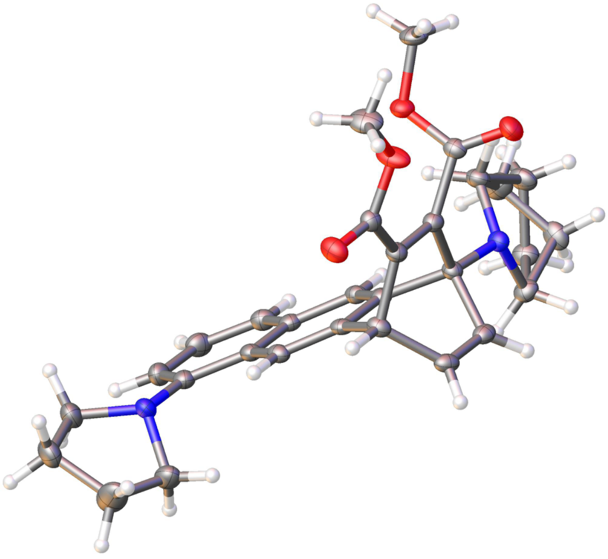

**Compound**	**N077**
CCDC	1979833
Formula	$C_{28}H_{30}N_2O_4$
$D_{calc.}$/ g cm^{-3}	1.344
μ/mm^{-1}	0.724
Formula Weight	458.54
Colour	colourless
Shape	prism
Size/mm^3	0.24×0.11×0.08
T/K	123.01(10)
Crystal System	orthorhombic
Flack Parameter	0.22(5)
Hooft Parameter	0.25(5)
Space Group	$P2_12_12_1$
a/Å	9.08860(17)
b/Å	9.11769(15)
c/Å	27.3374(4)
α/°	90
β/°	90
γ/°	90
V/Å^3	2265.37(7)
Z	4
Z'	1
Wavelength/Å	1.54184
Radiation type	Cu K$_\alpha$
Θ_{min}/°	5.114
Θ_{max}/°	74.063
Measured Refl's.	35025
Ind't Refl's	4539
Refl's with I > 2(I)	4444
R_{int}	0.0436
Parameters	343
Restraints	36
Largest Peak	0.284
Deepest Hole	-0.164
GooF	1.057
wR_2 (all data)	0.0879
wR_2	0.0871
R_1 (all data)	0.0335
R_1	0.0329

Dimethyl 1-(pyrrolidin-1-yl)-1,4-dihydro-1,4-ethenoanthracene-2,3-dicarboxylate (3eD)

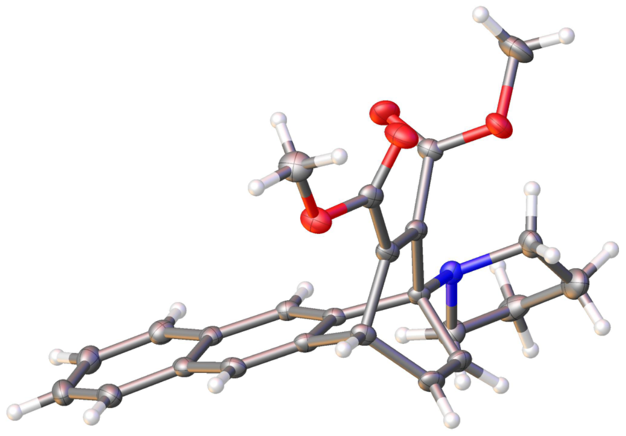

**Compound**	**R040**
CCDC	1979834
Formula	$C_{24}H_{23}NO_4$
$D_{calc.}$/ g cm^{-3}	1.347
μ/mm^{-1}	0.742
Formula Weight	389.43
Colour	clear yellow
Shape	block
Size/mm^3	0.30×0.27×0.20
T/K	122.9(2)
Crystal System	orthorhombic
Flack Parameter	-0.06(5)
Hooft Parameter	-0.05(5)
Space Group	$P2_12_12_1$
a/Å	6.74420(10)
b/Å	7.90290(10)
c/Å	36.0260(3)
α/°	90
β/°	90
γ/°	90
V/Å^3	1920.14(4)
Z	4
Z'	1
Wavelength/Å	1.54184
Radiation type	CuK$_\alpha$
Θ_{min}/°	4.910
Θ_{max}/°	74.780
Measured Refl.	22748
Independent Refl.	3901
Reflections Used	3824
R_{int}	0.0300
Parameters	264
Restraints	0
Largest Peak	0.155
Deepest Hole	-0.245
GooF	1.049
wR_2 (all data)	0.0771
wR_2	0.0765
R_1 (all data)	0.0291
R_1	0.0285

Dimethyl (6*R*,12*S*)-12-bromo-9-oxo-9,10-dihydro-6*H*-6,10-methanocyclohepta-[*b*]naphthalene-7,8-dicarboxylate (15)

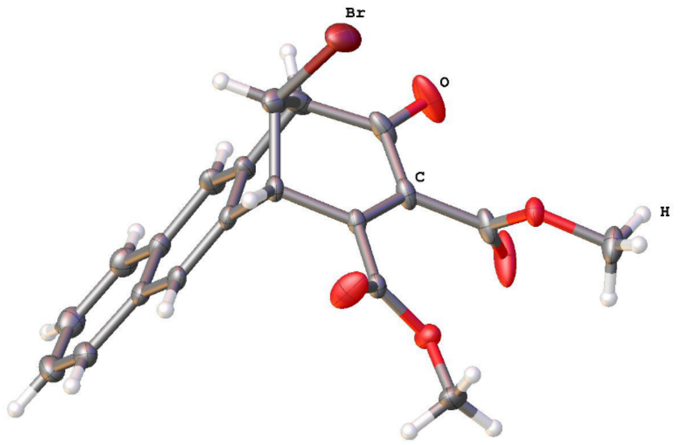

**Compound**	**T086**
CCDC	2017368
Formula	$C_{20}H_{15}BrO_5$
$D_{calc.}$/ g cm^{-3}	1.587
μ/mm^{-1}	3.482
Formula Weight	415.23
Colour	clear yellow
Shape	irregular
Size/mm^3	0.24×0.13×0.08
T/K	123.01(10)
Crystal System	monoclinic
Space Group	$P2_1/n$
a/Å	7.79240(10)
b/Å	11.7162(2)
c/Å	19.1249(3)
α/°	90
β/°	95.530(2)
γ/°	90
V/Å^3	1737.93(5)
Z	4
Z'	1
Wavelength/Å	1.54184
Radiation type	Cu K$_\alpha$
Θ_{min}/°	4.431
Θ_{max}/°	72.612
Measured Refl's.	23376
Indep't Refl's	3442
Refl's I≥2 σ(I)	3136
R_{int}	0.0461
Parameters	237
Restraints	0
Largest Peak	0.700
Deepest Hole	-0.975
GooF	1.053
wR_2 (all data)	0.0750
wR_2	0.0721
R_1 (all data)	0.0338
R_1	0.0301

7. Computational Studies

All calculations have been conducted with the Gaussian09 E.01 suite of programs[6]. The calculations were carried out at the B3LYP-D3/6-31G**[7–9] level of theory with Grimme's D3 dispersion correction[10] accounting for long-range dispersion interactions. Stationary points were confirmed as ground or transition states with the computation of the harmonic vibrational frequencies and evaluating the number of imaginary frequencies (0 for ground state, 1 for transition state). IRC calculations were conducted to classify the transition states as the correct one and also to find potential hints of step-wise processes by analyzing the RMS gradient. Population analyses according to Mulliken[11] and NBO[12–16] were employed to derive FMO information and provide access to partial charges.

8. Calculated Thermodynamic Data

Supplementary Table 2: Thermodynamic data for the reaction of anthracenes with DMAD D.

#	parent anthracene	9,10 addition				1,4 addition			
		DG≠ (kcal mol^{-1})	DH≠ (kcal mol^{-1})	D_RG (kcal mol^{-1})	D_RH (kcal mol^{-1})	DG≠ (kcal mol^{-1})	DH≠ (kcal mol^{-1})	D_RG (kcal mol^{-1})	D_RH (kcal mol^{-1})
1	**1a**	25.7	13.5	−24.6	−39.6	31.9	19.5	−12.4	−27.2
2	**1b**	26.7	13.6	−22.7	−38.0	28.1	14.3	−6.9	−22.7
3	**1c**	25.0	11.9	−25.6	−41.4	25.0	10.2	−5.1	−21.7
4	**1d**	24.4	8.2	−26.8	−42.9	21.6	5.4	−5.0	−21.0
5[a]	**1e**	29.1	14.8	−24.8	−40.9	23.1	7.0	−19.7	−2.3

[a] values (in kcal mol^{-1}) for 5,8 addition: DG≠ = 36.1 ; DH≠ = 22.0 ; D_RG = -10.8 ; D_RH = -42.3

Supplementary Table 3: Thermodynamic data for the reaction of anthracenes with maleic anhydride B.

#	parent anthracene	9,10 addition				1,4 addition (endo)			
		DG≠ (kcal mol^{-1})	DH≠ (kcal mol^{-1})	D_RG (kcal mol^{-1})	D_RH (kcal mol^{-1})	DG≠ (kcal mol^{-1})	DH≠ (kcal mol^{-1})	D_RG (kcal mol^{-1})	D_RH (kcal mol^{-1})
1	**1a**	23.2	9.8	-8.6	-22.6	31.0	17.5	4.1	-9.9
2	**1b**	22.8	8.9	-7.4	-21.9	33.4	19.5	11.4	-3.4
3	**1c**	22.1	8.0	-8.5	-23.4	24.8	10.6	14.3	-0.8
4	**1d**	19.8	6.1	-10.1	-24.5	17.1	1.2	8.1	-7.5
5[a,b]	**1e**	28.5	14.1	-7.3	-21.9	19.5	4.3	7.9	-7.5

[a] values (in kcal mol^{-1}) for 5,8 addition: DG≠ = 31.0 ; DH≠ = 17.0 ; D_RG = 4.4 ; D_RH = -10.1 ;
[b] Values for 9,10 addition for endo pathway

Supplementary Table 4: Thermodynamic data for the reaction of anthracenes with maleic anhydride B.

#	parent anthracene	9,10 addition				1,4 addition (exo)			
		DG≠ (kcal mol^{-1})	DH≠ (kcal mol^{-1})	D_RG (kcal mol^{-1})	D_RH (kcal mol^{-1})	DG≠ (kcal mol^{-1})	DH≠ (kcal mol^{-1})	D_RG (kcal mol^{-1})	D_RH (kcal mol^{-1})
1	**1a**	23.2	9.8	-8.6	-22.6	30.6	17.3	3.9	-10.0
2	**1b**	22.8	8.9	-7.4	-21.9	29.9	16.2	10.1	-4.8
3	**1c**	22.1	8.0	-8.5	-23.4	22.6	7.8	12.7	-2.5
4	**1d**	19.8	6.1	-10.1	-24.5	17.9	2.5	11.5	-3.6
5[a,b]	**1e**	22.7	9.0	-7.3	-22.6	17.4	2.3	10.9	-3.5

[a] values (in kcal mol^{-1}) for 5,8 addition: DG≠ = 30.4 ; DH≠ = 16.6 ; D_RG = 4.2 ; D_RH = -10.3 ;
[b] Values for 9,10 addition for exo pathway

9. Calculation of Bond Orders

The bond orders have been calculated with Pauling's equation (equation 1) with 0.6 as a fitting factor for transition states.[17,18] As reference, the according product structures were used.

$$BO = n_0 \times e^{\frac{r_0 - r_x}{c}} \quad (1)$$

With n_0 being the reference bond order, r_0 being the reference bond length in Å, r_x being the examined bond length in Å and c a factor with 0.3 for ground states and 0.6 for transition states.

Supplementary Table 5: Bond lengths in the product structures for 2a-eB and 3a-eB.

#	parent anthracene	1,4-Addition *exo*		1,4-Addition *endo*		9,10-Addition	
		C1 – CA (Å)	C4 – CB (Å)	C1 – CA (Å)	C4 – CB (Å)	C9-CA (Å)	C10-CB (Å)
1	**1a**	1.57	1.57	1.57	1.57	1.57	1.57
2	**1b**	1.58	1.58	1.59	1.57	1.57	1.57
3	**1c**	1.59	1.58	1.63	1.56	1.56	1.57
4	**1d**	1.59	1.57	1.59	1.57	1.57	1.57
5[a,b,c]	**1e**	1.60	1.57	1.59	1.57	1.57	1.57

[a] for 5,8 addition endo product: C5 – CA = 1.57, C8 – CB = 1.57 ; [b] for 5,8 addition exo product: C5 – CA = 1.57, C8 – CB = 1.57 ; [c] values in table for exo addition product. Values for endo product: C9 – CA = 1.57, C10 – CB = 1.57

Supplementary Table 6: Bond lengths in the transition state structures for 2a-eB and 3a-eB.

#	parent anthracene	1,4-Addition *exo*		1,4-Addition *endo*		9,10-Addition	
		C1 – CA (Å)	C4 – CB (Å)	C1 – CA (Å)	C4 – CB (Å)	C9-CA (Å)	C10-CB (Å)
1	**1a**	2.13	2.13	2.14	2.14	2.19	2.19
2	**1b**	2.46	1.86	2.42	1.93	2.17	2.18
3	**1c**	2.66	1.85	2.73	1.83	2.15	2.25
4	**1d**	2.76	1.86	2.79	1.85	2.16	2.18
5[a,b,c]	**1e**	2.75	1.86	2.78	1.86	2.20	2.16

[a] for 5,8 addition *endo* product: C5 – CA = 2.13, C8 – CB = 2.13 ; [b] for 5,8 addition *exo* product: C5 – CA = 2.14, C8 – CB = 2.14 ; [c] values in table for *exo* addition transition state. Values for *endo* transition state: C9 – CA = 2.28, C10 – CB = 2.12.

Supplementary Table 7: Bond orders after Pauling, calculated after equation 1 for structures 2a-eB and 3a-eB.

#	parent anthracene	1,4-Addition *exo* C1 – CA (Å)	C4 – CB (Å)	1,4-Addition *endo* C1 – CA (Å)	C4 – CB (Å)	9,10-Addition C9-CA (Å)	C10-CB (Å)
1	**1a**	0.39	0.39	0.39	0.39	0.36	0.36
2	**1b**	0.23	0.63	0.25	0.55	0.37	0.36
3	**1c**	0.17	0.64	0.16	0.64	0.37	0.32
4	**1d**	0.14	0.62	0.14	0.63	0.37	0.36
5[a,b,c]	**1e**	0.15	0.62	0.14	0.62	0.35	0.37

[a] for 5,8 addition *endo*: C5 – CA = 0.39, C8 – CB = 0.39 ; [b] for 5,8 addition *exo* product: C5 – CA = 0.39, C8 – CB = 0.39 ; [c] values in table for *exo* addition transition state. Values for *endo* transition state: C9 – CA = 0.31, C10 – CB = 0.40.

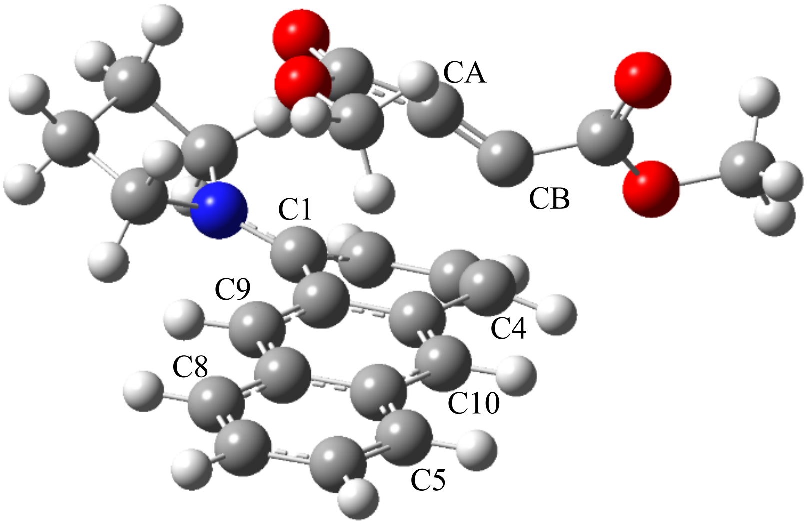

Supplementary Figure 90: Assignment of the carbon atoms for the calculation of the bond orders.

Supplementary Table 8: Computed bond lengths in product structures for 2a-eD and 3a-eD.

#	parent anthracene	1,4-Addition C1 – CA (Å)	C4 – CB (Å)	9,10-Addition C9-CA (Å)	C10-CB (Å)
1	**1a**	1.54	1.53	1.54	1.54
2	**1b**	1.56	1.56	1.53	1.53
3	**1c**	1.55	1.53	1.54	1.54
4	**1d**	1.56	1.55	1.53	1.53
5[a]	**1e**	1.56	1.53	1.54	1.53

[a] for 5,8 addition, values are: C5-CA = 1.53, C8 – CB = 1.54

Supplementary Table 9: Computed bond lengths in transition state structures for 2a-eD and 3a-eD.

#	parent anthracene	1,4-Addition		9,10-Addition	
		C1 – CA (Å)	C4 – CB (Å)	C9-CA (Å)	C10-CB (Å)
1	**1a**	2.40	2.04	2.50	2.05
2	**1b**	2.76	1.91	2.61	2.03
3	**1c**	3.08	1.96	2.26	2.15
4	**1d**	3.35	1.94	3.50	1.86
5[a]	**1e**	3.18	1.95	2.20	2.31

[a] for 5,8 addition, values are: C5-CA = 2.20, C8 – CB = 2.20

Supplementary Table 10: Calculated bond orders in transition state structures for 2a-eD and 3a-eD.

#	parent anthracene	1,4-Addition		9,10-Addition	
		C1 – CA (Å)	C4 – CB (Å)	C9-CA (Å)	C10-CB (Å)
1	**1a**	0.24	0.43	0.20	0.42
2	**1b**	0.13	0.56	0.17	0.44
3	**1c**	0.08	0.51	0.30	0.36
4	**1d**	0.05	0.52	0.04	0.58
5[a]	**1e**	0.07	0.50	0.33	0.28

[a] for 5,8 addition, values are: C5-CA = 0.33, C8 – CB = 0.33

10. Marcus Analysis

The data for the intrinsic barrier were calculated after the following equation 2[19,20]:

$$\Delta G^{\neq} = \Delta G^{\neq\circ} + 0.5\Delta_R G + \frac{\Delta_R G^2}{16\Delta G^{\neq\circ}} \qquad (2)$$

With $\Delta G^{\neq}$ being the activation barrier, $\Delta G^{\neq\circ}$ being the intrinsic barrier and $\Delta_R G$ being the driving force.

Supplementary Table 11: Calculated intrinsic barriers for the reaction of anthracenes with DMAD D.

#	cmpd	R, R' =	$\Delta G^{\neq}_{9,10}$ [a]	$\Delta_R G_{9,10}$ [a]	$\Delta G^{\neq\circ}_{9,10}$ [a]	$\Delta G^{\neq}_{1,4}$ [a]	$\Delta_R G_{1,4}$ [a]	$\Delta G^{\neq\circ}_{1,4}$ [a]
1	**1a**	H	25.7	−24.6	37.0	31.9	−12.4	37.9
2	**1b**	OMe	26.7	−22.7	37.2	28.1	−6.9	31.5
3	**1c**	NMe_2	25.0	−25.6	35.7	25.0	−5.1	27.5
4	**1d**	$N(CH_2)_4$	23.1	−26.8	36.6	21.6	−5.0	24.0
5	**1e**[b]	H, $N(CH_2)_4$	29.1	−24.8	40.6	23.1	−2.3	24.2

[a] (kcal mol^{-1}), [b] values for 5,8 position: $\Delta G^{\neq}_{5,8} = 36.1$; $\Delta_R G_{5,8} = -10.8$, $\Delta G^{\neq\circ}_{5,8} = 41.3$.

Supplementary Table 12: Calculated intrinsic barriers for the reaction of anthracenes with maleic anhydride B (*endo* path for 1,4-addition process).

#	cmpd	R, R' =	$\Delta G^{\neq}_{9,10}$ [a]	$\Delta_R G_{9,10}$ [a]	$\Delta G^{\neq\circ}_{9,10}$ [a]	$\Delta G^{\neq}_{1,4}$ [a]	$\Delta_R G_{1,4}$ [a]	$\Delta G^{\neq\circ}_{1,4}$ [a]
1	**1a**	H	23.2	-8.6	27.3	31.0	4.1	28.9
2	**1b**	OMe	22.8	-7.4	26.4	33.4	11.4	27.4
3	**1c**	NMe_2	22.1	-8.5	26.2	24.8	14.3	16.9
4	**1d**	$N(CH_2)_4$	19.8	-10.1	24.6	17.1	8.1	12.7
5	**1e**[b]	H, $N(CH_2)_4$	28.5	-7.3	33.0	19.5	7.9	15.3

[a] (kcal mol^{-1}), [b] values for 5,8 position: $\Delta G^{\neq}_{5,8} = 31.0$; $\Delta_R G_{5,8} = 4.4$; $\Delta G^{\neq\circ}_{5,8} = 28.8$.

Supplementary Table 13: Calculated intrinsic barriers for the 1,4-addition process of anthracenes with maleic anhydride, *exo* pathway.

#	cmpd	R, R' =	$\Delta G^{\neq}_{9,10}$ [a]	$\Delta_R G_{9,10}$ [a]	$\Delta G^{\neq\circ}_{9,10}$ [a]	$\Delta G^{\neq}_{1,4}$ [a]	$\Delta_R G_{1,4}$ [a]	$\Delta G^{\neq\circ}_{1,4}$ [a]
1	**1a**	H	23.2	-8.6	27.3	30.6	3.9	28.6
2	**1b**	OMe	22.8	-7.4	26.4	29.9	10.1	24.6
3	**1c**	NMe_2	22.1	-8.5	26.2	22.6	12.7	15.6
4	**1d**	$N(CH_2)_4$	19.8	-10.1	24.6	17.9	11.5	11.4
5	**1e**[b]	H, $N(CH_2)_4$	22.7	-7.3	26.2	17.4	10.9	11.3

11. FMO Analysis

The computed orbital energies from the Mulliken analysis were used for the calculation of the HOMO-LUMO gap.

Supplementary Table 14: FMO Analysis for anthracenes 1a-e with DMAD D.

#	parent anthracene	normal electron demand			inverse electron demand		
		e_{HOMO} (D) (eV)	e_{LUMO}(DP) (eV)	$D_{HOMO-LUMO}$ (eV)	e_{HOMO} (DP) (eV)	e_{LUMO}(D) (eV)	$D_{HOMO-LUMO}$ (eV)
1	**1a**	-5.23		3.74		-1.65	6.24
2	**1b**	-4.82		3.33		-1.27	6.62
3	**1c**	-4.81	-1.49	3.32	-7.89	-1.40	6.49
4	**1d**	-4.37		2.88		-1.12	6.77
5	**1e**	-4.68		3.19		-1.38	6.51

Supplementary Table 15: FMO Analysis for anthracenes 1a-e with maleic anhydride B.

#	parent anthracene	normal electron demand			inverse electron demand		
		e_{HOMO} (D) (eV)	e_{LUMO}(DP) (eV)	$D_{HOMO-LUMO}$ (eV)	e_{HOMO} (DP) (eV)	e_{LUMO}(D) (eV)	$D_{HOMO-LUMO}$ (eV)
1	**1a**	-5.23		2.04		-1.65	6.49
2	**1b**	-4.82		1.63		-1.27	6.87
3	**1c**	-4.81	-3.19	1.62	-8.14	-1.40	6.74
4	**1d**	-4.37		1.18		-1.12	7.02
5	**1e**	-4.68		1.49		-1.38	6.76

12. NBO Analysis (Partial Charges)

The partial charges were taken from the NBO analysis. In all cases, the charge from the neighbouring hydrogen atom was added to the charge of the carbon centre.

Supplementary Table 16: Partial charges at the reaction centres for the reaction of anthracenes with DMAD D.

			**1,4-addition**				**9,10-addition**			
#	**Cmpd**	**R = R′ =**	**CA**	**CB**	**C1**	**C4**	**CA**	**CB**	**C9**	**C10**
1	**1a**	H	-0.023	-0.130	0.049	0.076	-0.132	-0.013	0.074	0.102
2	**1b**	OMe	-0.025	-0.153	0.410	0.005	-0.130	-0.022	0.083	0.119
3	**1c**	NMe_2	-0.060	-0.131	0.269	-0.012	-0.103	-0.052	0.077	0.091
4	**1d**	$N(CH_2)_4$	-0.058	-0.148	0.283	-0.022	-0.173	-0.047	0.019	0.125
5	**1e**[b]	H, $N(CH_2)_4$	-0.103	-0.138	0.282	-0.032	-0.123	-0.086	0.068	0.096

[a] Values for 5,8-addition: CA = -0.106, CB = -0.110, C5 = 0.074, C8 = 0.056.

Supplementary Table 17: Partial charges at the reaction centers for the reaction of anthracenes with maleic anhydride B.

			**1,4-addition *endo***				**9,10-addition**			
#	**Cmpd**	**R = R′ =**	**CA**	**CB**	**C1**	**C4**	**CA**	**CB**	**C9**	**C10**
1	**1a**	H	-0.086	-0.086	0.075	0.075	-0.083	-0.083	0.102	0.102
2	**1b**	OMe	-0.150	-0.076	0.403	0.030	-0.087	-0.087	0.118	0.117
3	**1c**	NMe_2	-0.201	-0.070	0.273	0.004	-0.083	-0.096	0.107	0.112
4	**1d**	$N(CH_2)_4$	-0.222	-0.057	0.293	-0.003	-0.084	-0.084	0.106	0.101
5	**1e**[b]	H, $N(CH_2)_4$	-0.214	-0.065	0.287	-0.004	-0.094	-0.078	0.104	0.093

[a] Values for 5,8-addition *endo*: CA = -0.089, CB = -0.087, C5 = 0.074, C8 = 0.074.

[b] Values for 9,10-addition are for endo case. Values for 9,10-addition *exo*: CA = -0.093, CB = -0.084, C9 = 0.112, C10 = 0.088

Supplementary Table 18: Partial charges at the reaction centres for the reaction of anthracenes with maleic anhydride B.

			**1,4-addition *exo***			
**#**	**Cmpd**	**R = R' =**	**CA**	**CB**	**C1**	**C4**
1	**1a**	H	-0.079	-0.079	0.074	0.074
2	**1b**	OMe	-0.142	-0.066	0.414	0.020
3	**1c**	NMe_2	-0.185	-0.060	0.272	0.005
4	**1d**	$N(CH_2)_4$	-0.201	-0.059	0.284	-0.003
5	**1e**[b]	H, $N(CH_2)_4$	-0.200	-0.057	0.282	-0.002

[a] Values for 5,8-addition *exo*: CA = -0.079, CB = -0.080, C5 = 0.073, C8 = 0.074

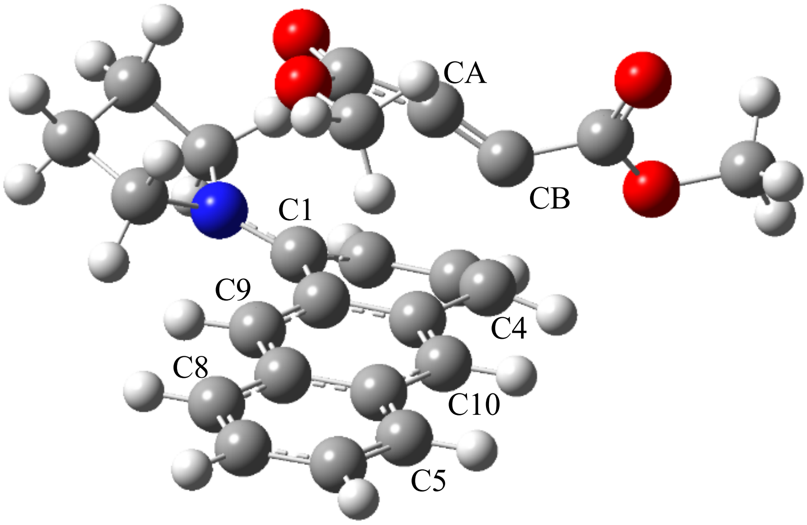

Supplementary Figure 91: Assignment of the carbon atoms for the calculation of the bond orders.

13. All Obtained IRC Plots

IRC Plots with DMAD D as Dienophile

The SCF Energy was plotted against the longer critical bond length in the transition state

(a) IRCs for R = R' = H

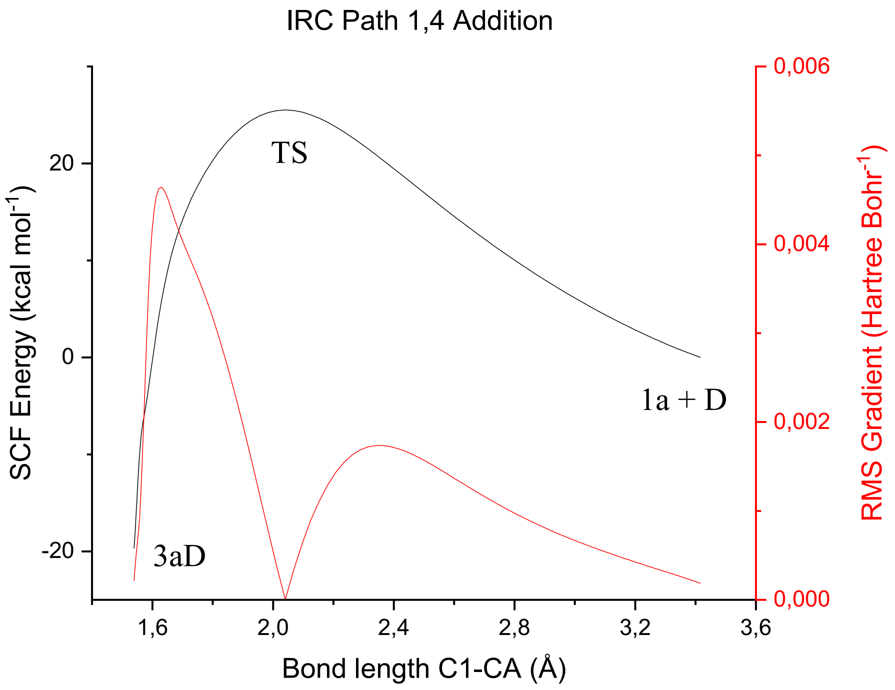

Supplementary Figure 92

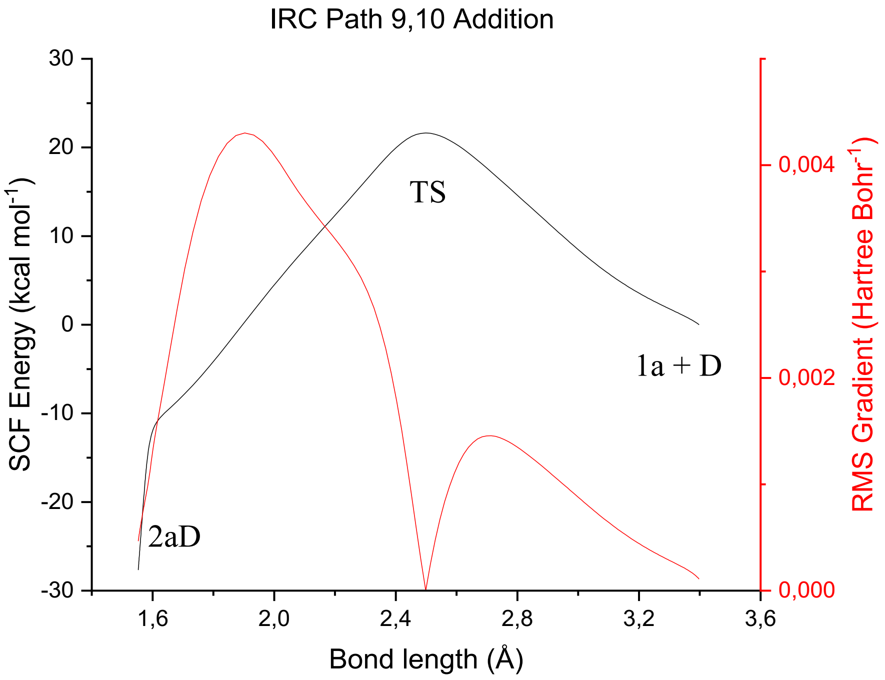

Supplementary Figure 93

(b) IRCs for R = R' = OMe

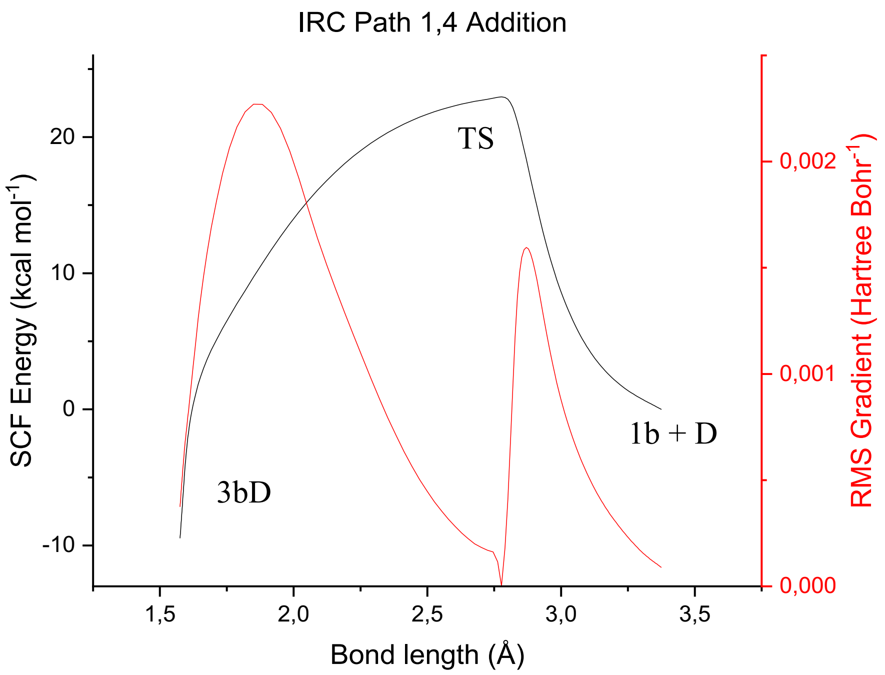

Supplementary Figure 94

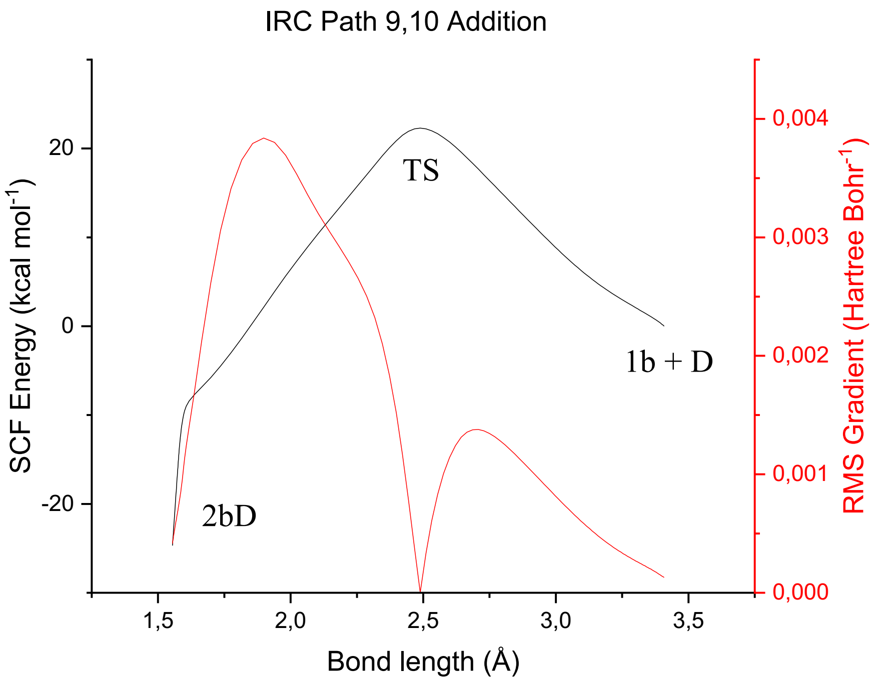

Supplementary Figure 95

(c) IRCs for R = R' = NMe_2

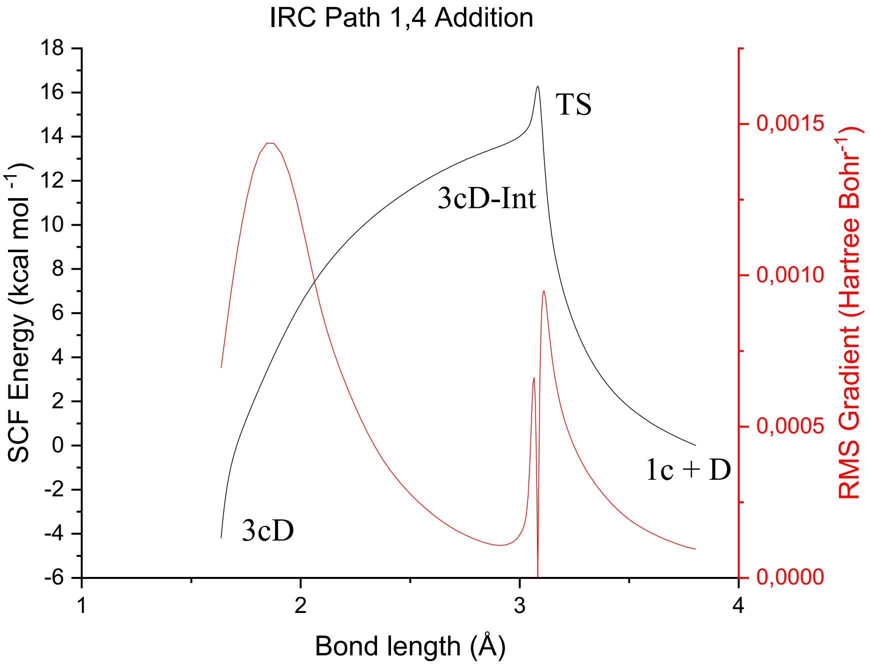

Supplementary Figure 96

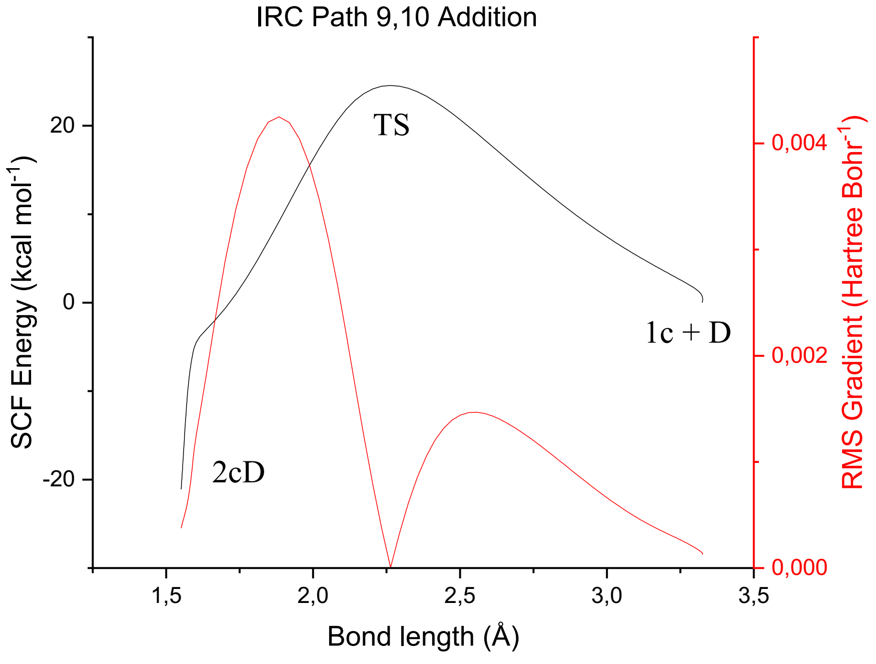

Supplementary Figure 97

IRC plots with maleic anhydride B as dienophile

(a) Plots for R = R’ = H

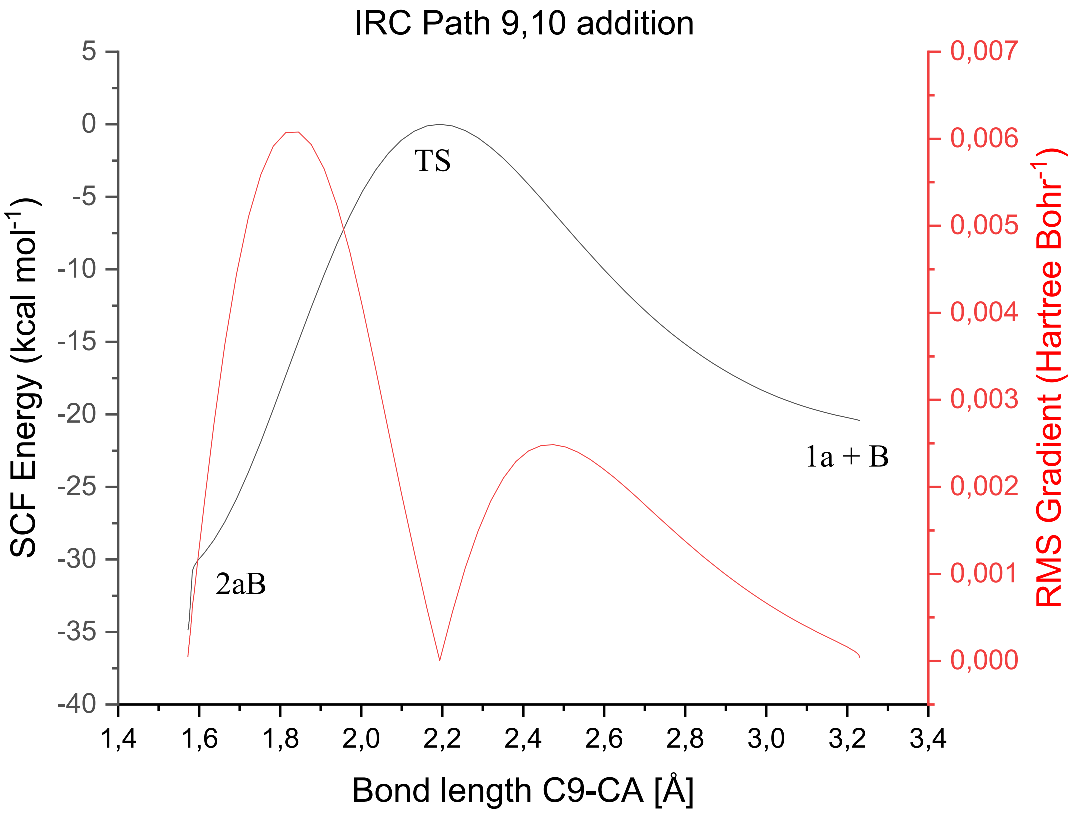

Supplementary Figure 98

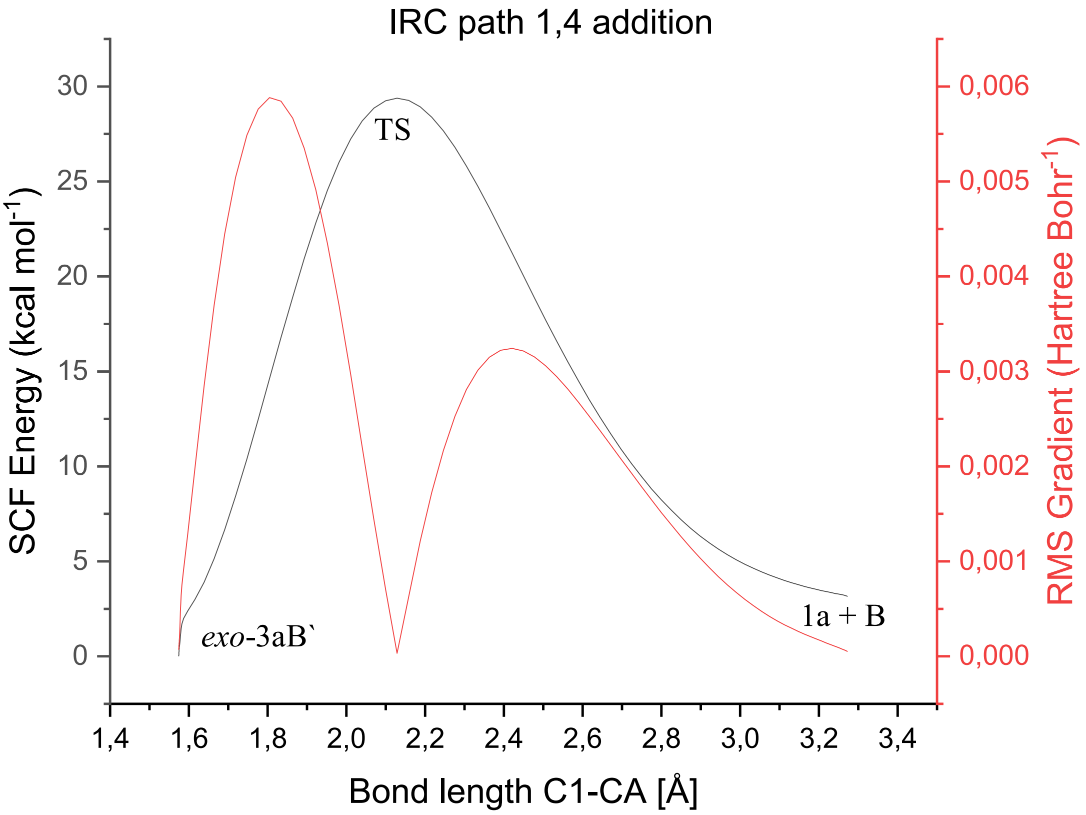

Supplementary Figure 99

(b) Plots for R = R' = OMe

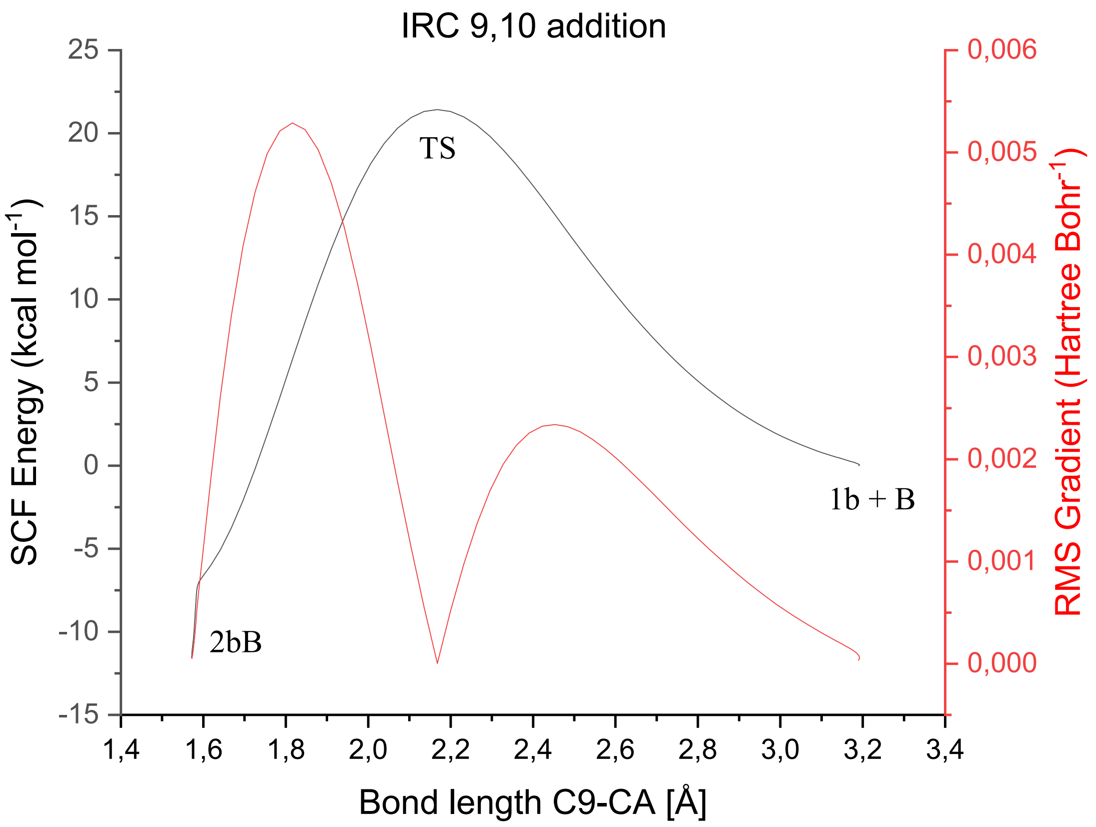

Supplementary Figure 100

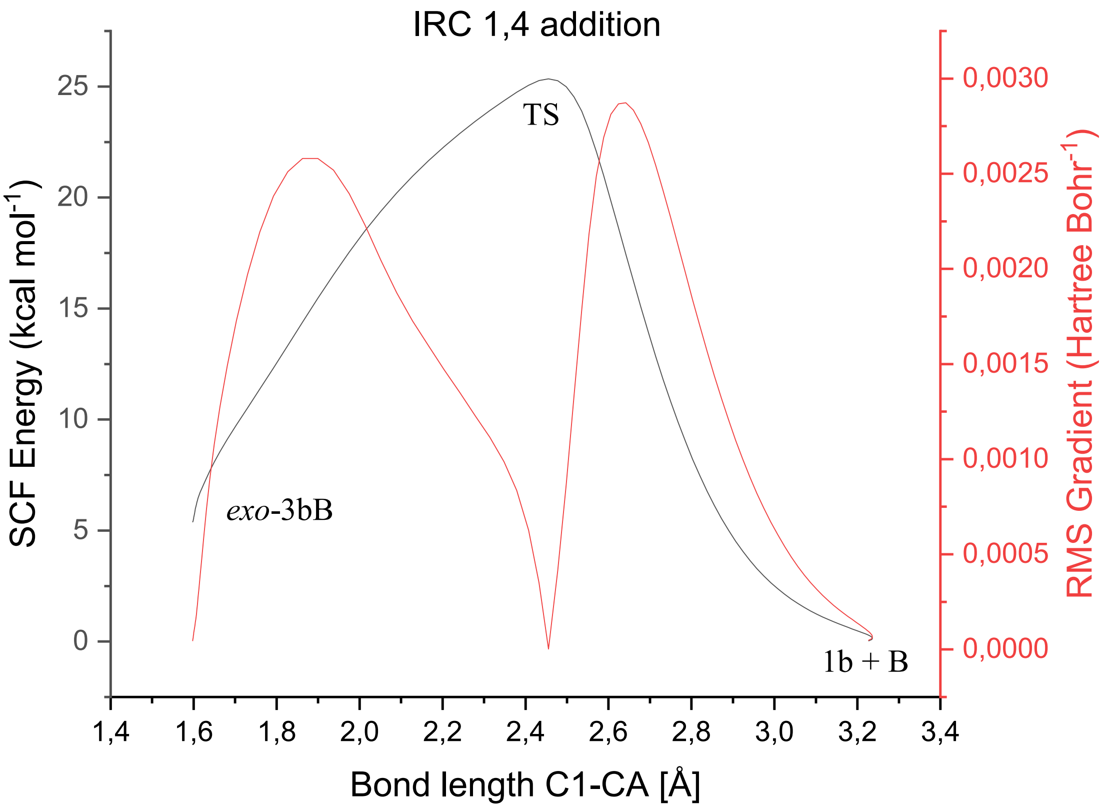

Supplementary Figure 101

(c) Plots for R = R’ = NMe_2

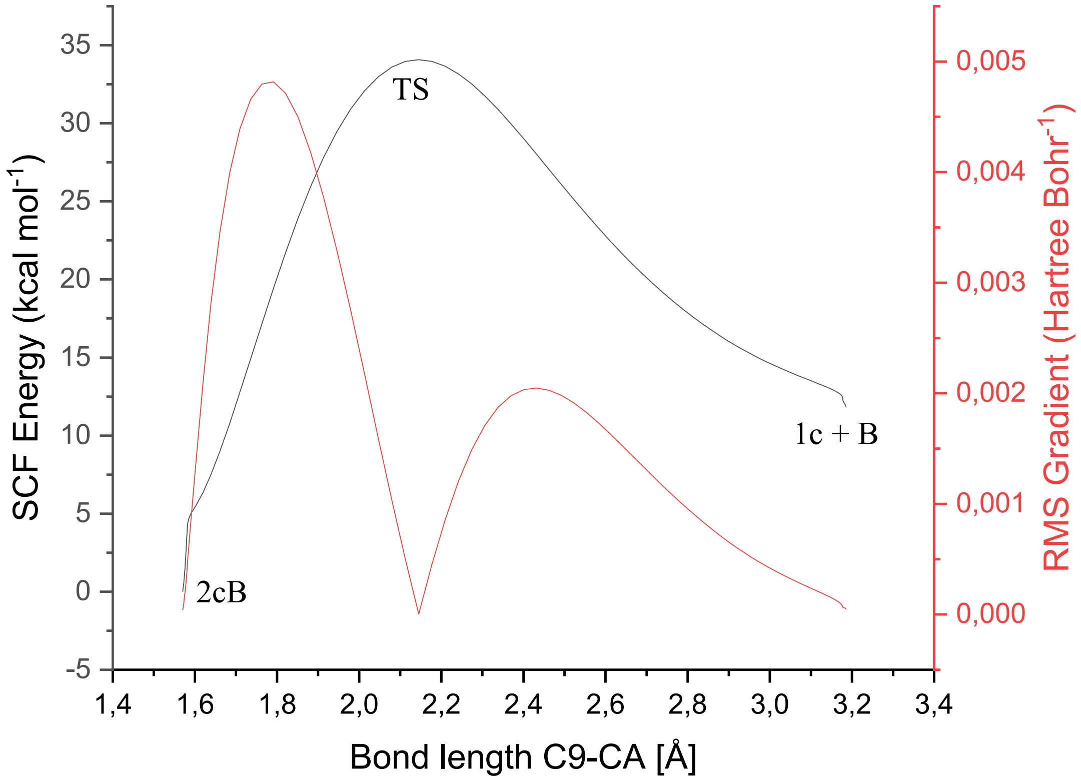

Supplementary Figure 102

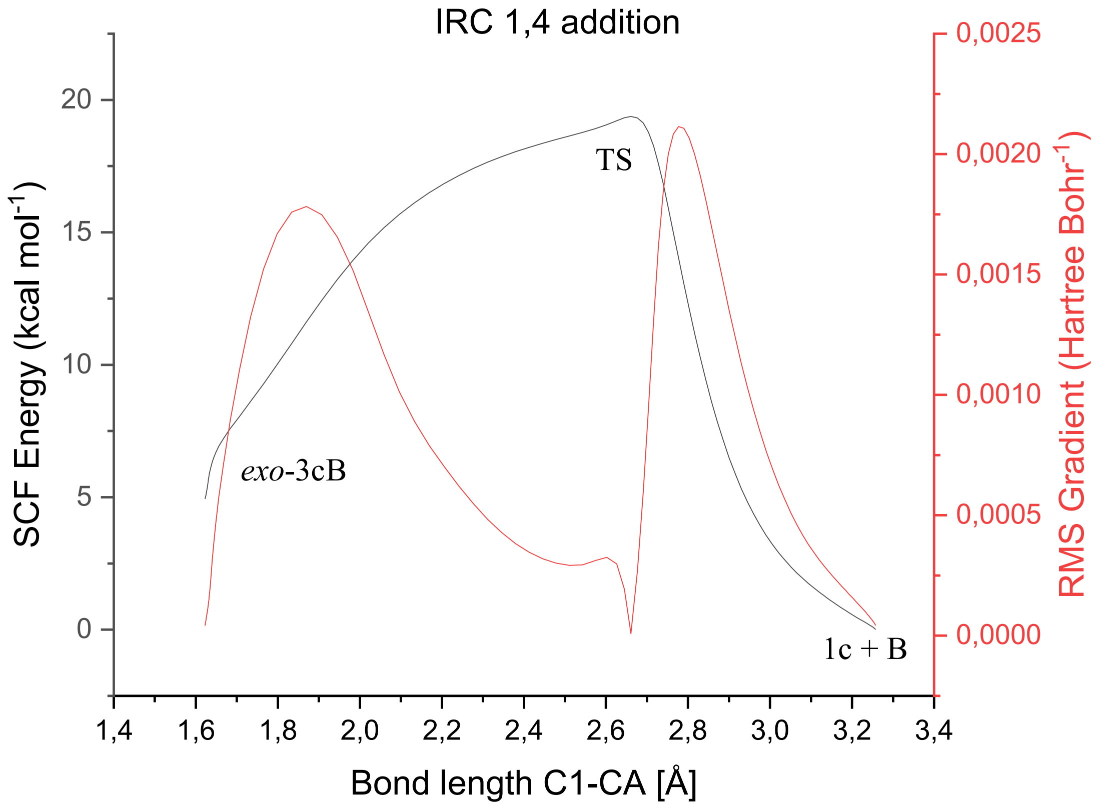

Supplementary Figure 103

(d) Plots for R = R’ = Pyrrolidine

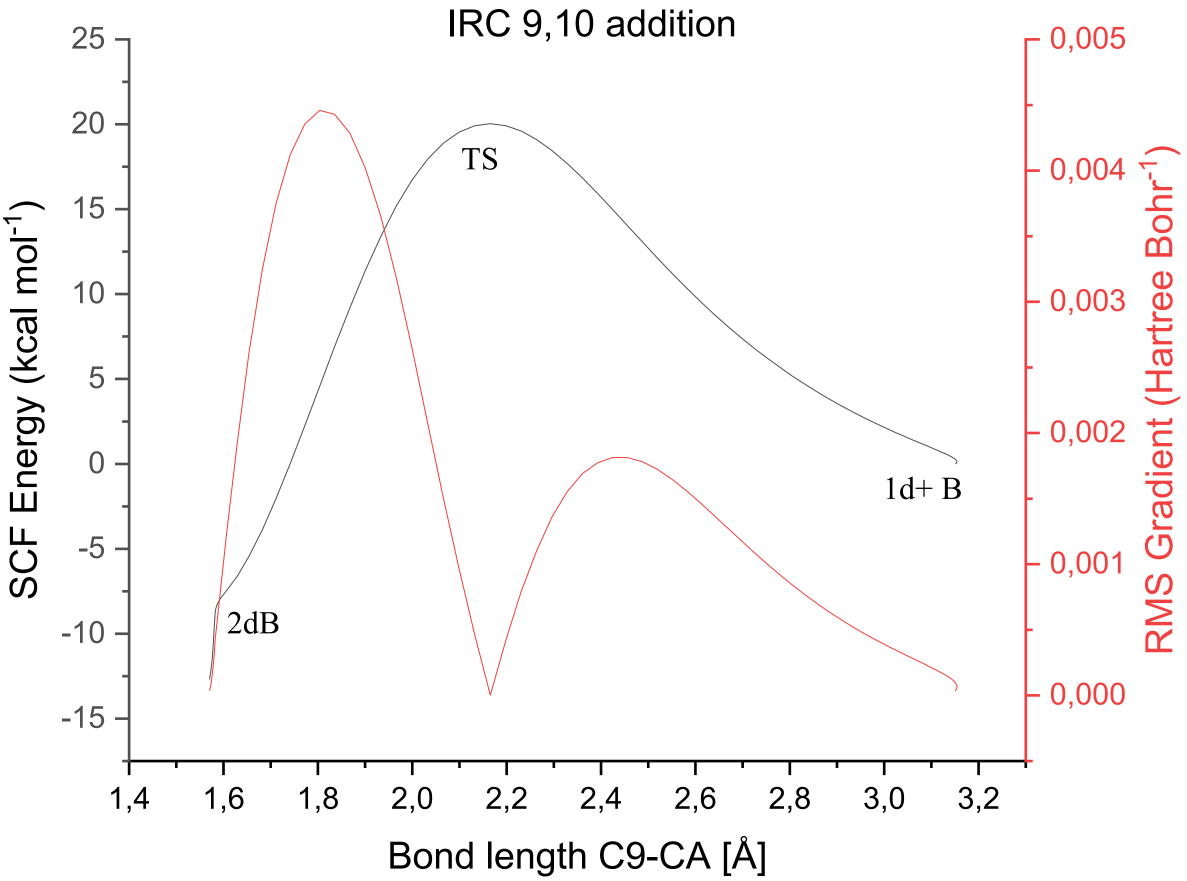

Supplementary Figure 104

(e) Plots for R = H ; R' = Pyrrolidine

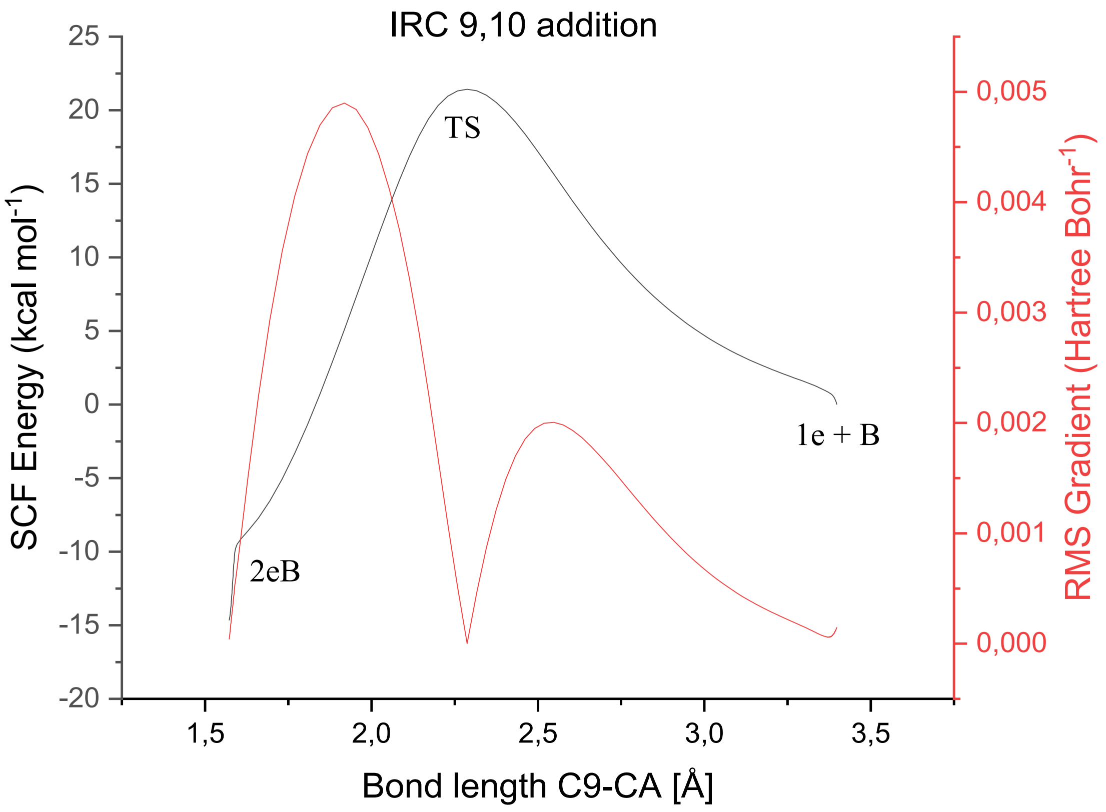

Supplementary Figure 105

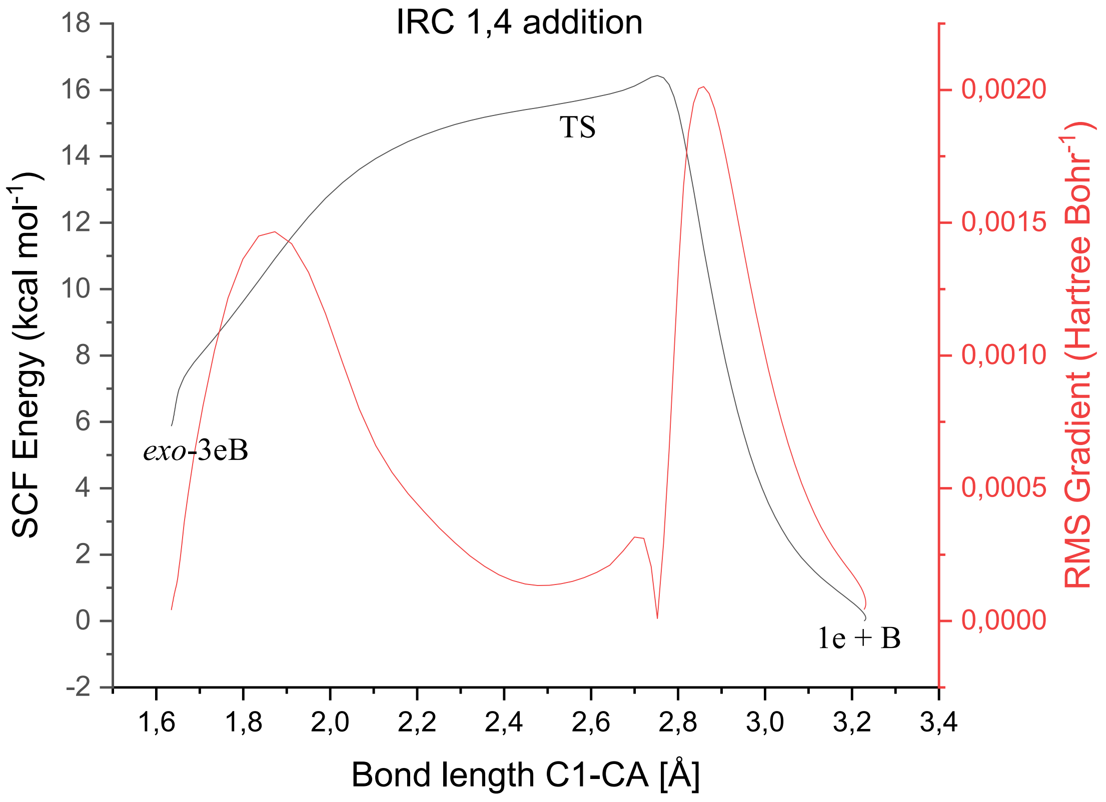

Supplementary Figure 106

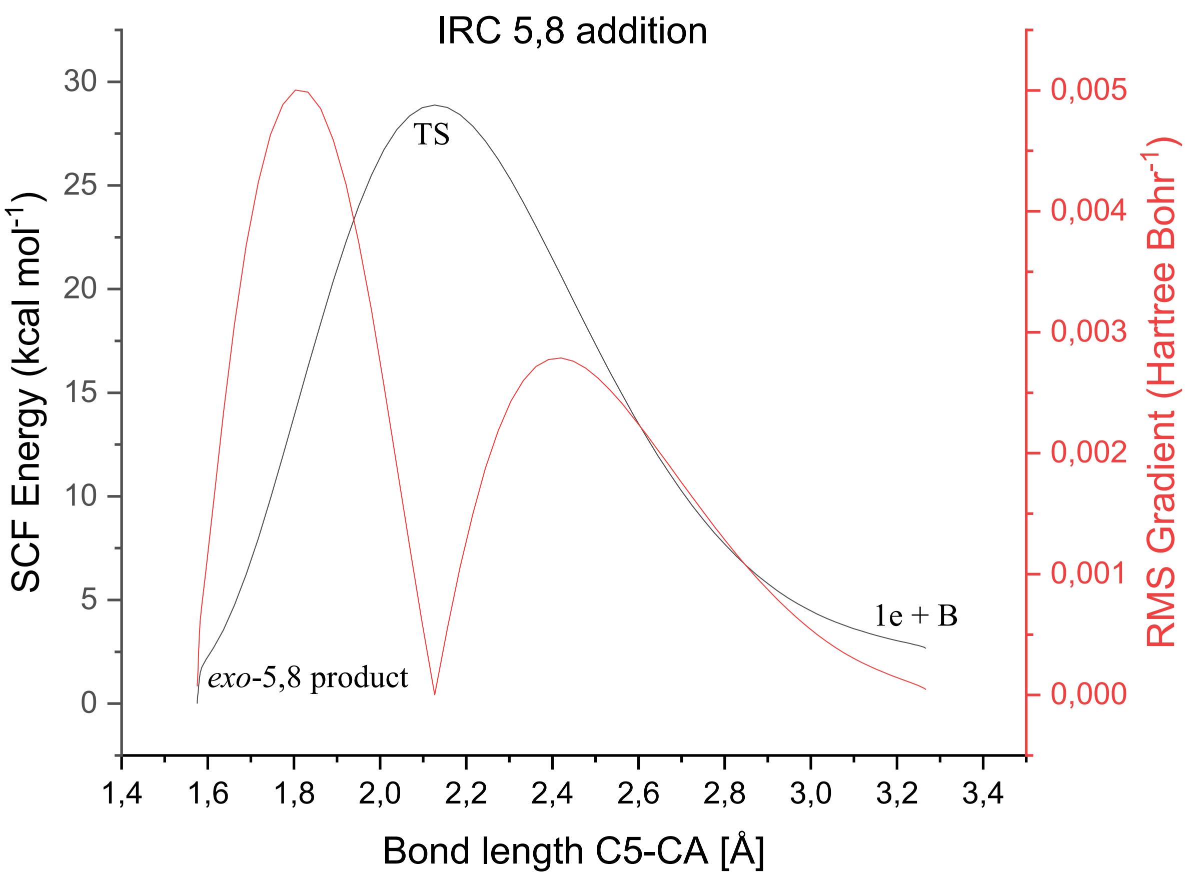

Supplementary Figure 107

14. Supplementary References

1. Carreno, M. C., Ruano, J. L. & Urbanoy, A. Asymmetric Diels-Alder reactions of (S)-2-(p-tolylsulfinyl)-1,4-naphthoquinones. *J. Org. Chem.* **57,** 6870–6876 (1992).

2. Tius, M. A., Gomez-Galeno, J. & Zaidi, J. H. A Bifunctional Anthraquinone Synthon. *Tetrahedron Lett.* **29,** 6909–6912 (1988).

3. Klanderman, B. H. Novel Products from the Reaction of Benzyne with Anthracenes. *J. Am. Chem. Soc.* **87,** 4649–4651 (1965); 10.1021/ja00948a049.

4. Verma, S. M. & Singh, M. D. Structural elucidation with nuclear magnetic resonance spectroscopy. Diels-Alder adducts of 1-aminoanthracene and maleic anhydride: restricted rotation about the aryl C(1)-N bond and intrinsic asymmetry about the imide (Nsp2-Csp3) system. *J. Org. Chem.* **42,** 3736–3740 (1977).

5. Bouffard, J., Eaton, R. F., Müller, P. & Swager, T. M. Iptycene-derived pyridazines and phthalazines. *J. Org. Chem.* **72,** 10166–10180 (2007); 10.1021/jo702000d.

6. M. J. Frisch, G. W. Trucks, H. B. Schlegel, G. E. Scuseria, M. A. Robb, J. R. Cheeseman, G. Scalmani, V. Barone, B. Mennucci, G. A. Petersson, H. Nakatsuji, M. Caricato, X. Li, H. P. Hratchian, A. F. Izmaylov, J. Bloino, G. Zheng, J. L. Sonnenberg, M. Hada, M. Ehara, K. Toyota, R. Fukuda, J. Hasegawa, M. Ishida, T. Nakajima, Y. Honda, O. Kitao, H. Nakai, T. Vreven, J. A. Montgomery, Jr., J. E. Peralta, F. Ogliaro, M. Bearpark, J. J. Heyd, E. Brothers, K. N. Kudin, V. N. Staroverov, T. Keith, R. Kobayashi, J. Normand, K. Raghavachari, A. Rendell, J. C. Burant, S. S. Iyengar, J. Tomasi, M. Cossi, N. Rega, J. M. Millam, M. Klene, J. E. Knox, J. B. Cross, V. Bakken, C. Adamo, J. Jaramillo, R. Gomperts, R. E. Stratmann, O. Yazyev, A. J. Austin, R. Cammi, C. Pomelli, J. W. Ochterski, R. L. Martin, K. Morokuma, V. G. Zakrzewski, G. A. Voth, P. Salvador, J. J. Dannenberg, S. Dapprich, A. D. Daniels, O. Farkas, J. B. Foresman, J. V. Ortiz, J. Cioslowski, and D. J. Fox. *Gaussian 09, Revision E.01* (Gaussian, Inc., Wallingford CT, 2013).

7. Lee, C., Yang, W. & Parr, R. G. Development of the Colle-Salvetti correlation-energy formula into a functional of the electron density. *Phys. Rev. B* **37,** 785–789 (1988).

8. Krishnan, R., Binkley, J. S., Seeger, R. & Pople, J. A. Self-consistent molecular orbital methods. XX. A basis set for correlated wave functions. *J. Chern. Phys.* **72,** 650–654 (1980); 10.1063/1.438955.

9. Becke, A. D. Density-functional thermochemistry. III. The role of exact exchange. *J. Chem. Phys.* **98,** 5648–5652 (1993); 10.1063/1.464913.

10. Grimme, S., Antony, J., Ehrlich, S. & Krieg, H. A consistent and accurate ab initio parametrization of density functional dispersion correction (DFT-D) for the 94 elements H-Pu. *J. Phys. Chem.* **132,** 154104 (2010); 10.1063/1.3382344.

11. Mulliken, R. S. Electronic Population Analysis on LCAO–MO Molecular Wave Functions. I. *J. Chem. Phys.* **23,** 1833–1840 (1955); 10.1063/1.1740588.

12. NBO Version 3.1, E. D. Glendening, A. E. Reed, J. E. Carpenter, and F. Weinhold as implemented in Gaussian09.E01.

13. Foster, J. P. & Weinhold, F. Natural hybrid orbitals. *J. Am. Chem. Soc.* **102,** 7211–7218 (1980).

14. Reed, A. E., Weinstock, R. B. & Weinhold, F. Natural population analysis. *J. Chem. Phys.* **83,** 735–746 (1985); 10.1063/1.449486.

15. Reed, A. E. & Weinhold, F. Natural localized molecular orbitals. *J. Chem. Phys.* **83,** 1736–1740 (1985); 10.1063/1.449360.

16. Reed, A. E., Curtiss, L. A. & Weinhold, F. Intermolecular interactions from a natural bond orbital, donor-acceptor viewpoint. *Chem. Rev.* **88,** 899–926 (1988).

17. Pauling, L. Atomic Radii and Interatomic Distances in Metals. *J. Am. Chem. Soc.* **69,** 543–553 (1947).

18. Houk, K. N., Gustafson, S. M. & Black, K. A. Theoretical secondary kinetic isotope effects and the interpretation of transition state geometries. 1. The Cope rearrangement. *J. Am. Chem. Soc.* **114,** 8565–8572 (1992).

19. Marcus, R. A. Theoretical relations among rate constants, barriers, and Broensted slopes of chemical reactions. *J. Phys. Chem.* **72,** 891–899 (1968).

20. Follet, E., Mayer, P. & Mayr, H. Lewis Acidities of Indol-3-ylmethylium Ions and Intrinsic Barriers of Their Reactions with Phosphines and Pyridines. *Eur. J. Org. Chem.* **2016,** 4050–4058 (2016); 10.1002/ejoc.201600572.
