# Supplementary material for: Diels–Alder reactions and electrophilic substitutions with atypical regioselectivity enable functionalization of terminal rings of anthracene
Source: Commun Chem. 2020 Nov 6;3:158. doi: 10.1038/s42004-020-00407-9 (PMC9814106; doi:10.1038/s42004-020-00407-9)
Supplement: Supplementary file 2 — Description of Additional Supplementary Files [file 42004_2020_407_MOESM2_ESM.pdf]

Description of Additional Supplementary Files

File Name: Supplementary Data 1
Description: Atomic coordinates for optimized computational models

File Name: Supplementary Data 2
Description: Cif file for compound 3bD

File Name: Supplementary Data 3
Description: Cif file for compound 3cD

File Name: Supplementary Data 4
Description: Cif file for compound 3dD

File Name: Supplementary Data 5
Description: Cif file for compound 3eD

File Name: Supplementary Data 6
Description: Cif file for compound 15
