# Supplementary material for: Diels–Alder reactions and electrophilic substitutions with atypical regioselectivity enable functionalization of terminal rings of anthracene
Source: Commun Chem. 2020 Nov 6;3:158. doi: 10.1038/s42004-020-00407-9 (PMC9814106; doi:10.1038/s42004-020-00407-9)
Supplement: Supplementary file 3 — Supplementary Data 1 [file 42004_2020_407_MOESM3_ESM.pdf]

Supplementary Data 1

for

Diels-Alder Reactions and Electrophilic Substitutions with Atypical Regioselectivity Enable Functionalization of Terminal Rings of Anthracene

Vinh Ngoc Huynh,[a,b] Michael Leitner,[a] Aditya Bhattacharyya,[a] Lisa Uhlstein,[a] Peter Kreitmeier,[a] Patrick Sakrausky,[a] Julia Rehbein,[a,*] Oliver Reiser [a,*]

[a]*Institut für Organische Chemie, Universität Regensburg, Universitätsstr. 31, 93053 Regensburg; email: Oliver.Reiser@chemie.uni-regensburg.de; Julia.Rehbein@chemie.uni-regensburg.de*

[b]*University of Science, Vietnam National University, 227 Nguyễn Văn Cừ street, district 5, Ho Chi Minh City, Vietnam*

Description: Supplementary Data 1

Contents: Atomic coordinates for optimized computational models

All Calculated Structures

1. Ground state optimized structures with B3LYP-D3/6-31G**

1.1. Anthracene 1a

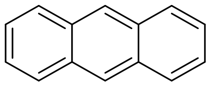	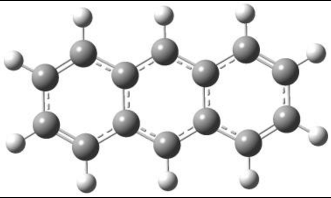

0 1

H	4.60484000	0.00001400	-1.24643200
C	3.65881400	0.00000500	-0.71316800
H	2.47564000	0.00001300	-2.49424500
C	2.47810100	0.00000700	-1.40734500
C	2.47810100	-0.00000700	1.40734500
C	1.22300900	0.00000300	-0.72269600
C	3.65881400	-0.00000500	0.71316800
C	1.22300900	-0.00000300	0.72269600
C	0.00000000	0.00000000	-1.40411000
H	4.60484000	-0.00001400	1.24643200
H	0.00000000	0.00000000	2.49184400
H	2.47564000	-0.00001300	2.49424500
C	-1.22300900	-0.00000300	-0.72269600
H	0.00000000	0.00000000	-2.49184400
C	-2.47810100	-0.00000700	-1.40734500
C	-1.22300900	0.00000300	0.72269600
H	-2.47564000	0.00001300	2.49424500
C	0.00000000	0.00000000	1.40411000
C	-3.65881400	-0.00000500	-0.71316800
H	-2.47564000	-0.00001300	-2.49424500
H	-4.60484000	-0.00001400	-1.24643200
C	-3.65881400	0.00000500	0.71316800
H	-4.60484000	0.00001400	1.24643200
C	-2.47810100	0.00000700	1.40734500

Zero-point correction= 0.194307 (Hartree/Particle)
Thermal correction to Energy= 0.203741
Thermal correction to Enthalpy= 0.204685
Thermal correction to Gibbs Free Energy= 0.160855
Sum of electronic and zero-point Energies= -539.371900
Sum of electronic and thermal Energies= -539.362467
Sum of electronic and thermal Enthalpies= -539.361522
Sum of electronic and thermal Free Energies= -539.405352

	E (Thermal)	CV	S
	KCal/Mol	Cal/Mol-Kelvin	Cal/Mol-Kelvin
Total	127.849	40.633	92.248

1.2. Dimethyl acetylenedicarboxylate D

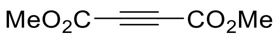

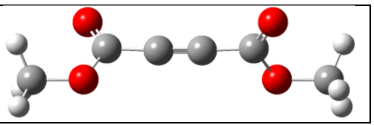

0 1

C	0.60213200	0.19400200	0.06109600
C	-0.60213400	0.19400400	-0.06110000
C	2.03464000	0.24929200	0.27780300
O	2.56531000	0.97764300	1.08820500
O	2.67629700	-0.60882500	-0.53440200
C	4.11172100	-0.61982300	-0.38824800
H	4.52464100	0.36786200	-0.60770500
H	4.46910400	-1.35696300	-1.10585100
H	4.38913600	-0.90254000	0.63022400
C	-2.03464600	0.24938700	-0.27772500
O	-2.56532100	0.97777800	-1.08809200
O	-2.67629400	-0.60887500	0.53433400
C	-4.11170800	-0.61989900	0.38816300
H	-4.52465100	0.36781300	0.60745100
H	-4.46909500	-1.35693900	1.10586900
H	-4.38910400	-0.90278300	-0.63027200

Zero-point correction= 0.114966 (Hartree/Particle)
Thermal correction to Energy= 0.125976
Thermal correction to Enthalpy= 0.126920
Thermal correction to Gibbs Free Energy= 0.076287
Sum of electronic and zero-point Energies= -532.977427
Sum of electronic and thermal Energies= -532.966417
Sum of electronic and thermal Enthalpies= -532.965473
Sum of electronic and thermal Free Energies= -533.016106

	E (Thermal)	CV	S
	KCal/Mol	Cal/Mol-Kelvin	Cal/Mol-Kelvin
Total	79.051	35.743	106.566

1.3. Maleic anhydride B

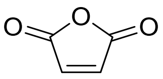

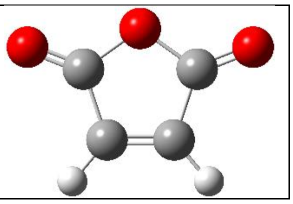

0 1

C	-0.00061900	-0.15894500	-1.13147900
C	0.00274000	1.25870200	-0.66761800
C	0.00274000	1.25870200	0.66761800
C	-0.00061900	-0.15894500	1.13147900

H	0.00446900	2.08953900	-1.36004200
H	0.00446900	2.08953900	1.36004200
O	-0.00306100	-0.97284900	0.00000000
O	-0.00061900	-0.59958500	-2.24506400
O	-0.00061900	-0.59958500	2.24506400

Zero-point correction= 0.055770 (Hartree/Particle)
Thermal correction to Energy= 0.060965
Thermal correction to Enthalpy= 0.061909
Thermal correction to Gibbs Free Energy= 0.026687
Sum of electronic and zero-point Energies= -379.241002
Sum of electronic and thermal Energies= -379.235807
Sum of electronic and thermal Enthalpies= -379.234863
Sum of electronic and thermal Free Energies= -379.270085

	E (Thermal)	CV	S
	KCal/Mol	Cal/Mol-Kelvin	Cal/Mol-Kelvin
Total	38.256	18.611	74.131

1.4. 1,5-Dimethoxyanthracene 1b

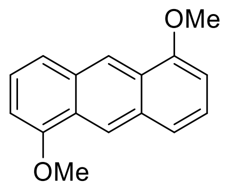

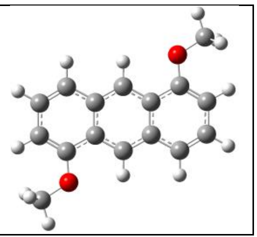

0 1

H	-3.48922000	-3.24574800	-0.00020300
C	-2.89584000	-2.33613800	-0.00015600
H	-1.02027800	-3.36517300	0.00003600
C	-1.53000500	-2.40629400	-0.00006500
C	-2.85477100	0.08946000	-0.00030000
C	-0.74722900	-1.21012000	-0.00008000
C	-3.57429600	-1.08284500	-0.00034600
C	-1.41318700	0.06833300	-0.00017000
C	0.65359200	-1.24151800	-0.00005000
H	-4.65707200	-1.07320900	-0.00055500
H	-1.16231600	2.19908300	-0.00020500
C	1.41319700	-0.06831100	-0.00009000
H	1.16233300	-2.19906100	-0.00002100
C	2.85477800	-0.08945000	-0.00012600
C	0.74724100	1.21014100	-0.00011700
H	1.02028800	3.36519400	-0.00013600
C	-0.65357900	1.24153900	-0.00015700
C	3.57430100	1.08285900	-0.00019900
H	4.65707400	1.07323200	-0.00026500
C	2.89584500	2.33615600	-0.00013900

H	3.48922200	3.24576700	-0.00020000
C	1.53001300	2.40631500	-0.00013000
O	3.40215100	-1.33913300	-0.00020800
C	4.81626800	-1.45252200	0.00083700
H	5.25843000	-0.99290900	-0.89283200
H	5.03394900	-2.52153300	0.00156900
H	5.25721500	-0.99191400	0.89459700
O	-3.40217200	1.33912700	-0.00056200
C	-4.81629400	1.45244000	0.00119100
H	-5.25888100	0.99301800	-0.89237200
H	-5.03402400	2.52144000	0.00227300
H	-5.25675900	0.99159000	0.89505500

Zero-point correction= 0.260091 (Hartree/Particle)
Thermal correction to Energy= 0.274675
Thermal correction to Enthalpy= 0.275619
Thermal correction to Gibbs Free Energy= 0.218832
Sum of electronic and zero-point Energies= -768.365534
Sum of electronic and thermal Energies= -768.350950
Sum of electronic and thermal Enthalpies= -768.350006
Sum of electronic and thermal Free Energies= -768.406793

	E (Thermal)	CV	S
	KCal/Mol	Cal/Mol-Kelvin	Cal/Mol-Kelvin
Total	172.361	58.689	119.519

1.5. N^{1},N^{1},N^{5},N^{5}-tetramethylanthracene-1,5-diamine 1c

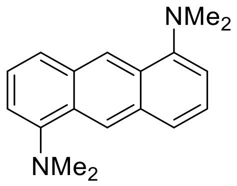

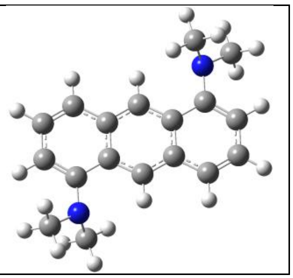

0 1

C	2.55784700	2.69731600	0.14896100
H	3.02046600	3.67859300	0.20469100
H	0.56364400	3.44077800	0.37430100
C	1.19933400	2.56920700	0.24548500
C	2.86000800	0.28227700	-0.05672500
C	0.58869100	1.28113200	0.14859500
C	3.39093600	1.55210200	0.00731000
C	1.42186700	0.11659800	-0.03139400
C	-0.80393000	1.12798400	0.19225500
H	4.46585700	1.69260200	-0.02039000
H	1.42135000	-2.00095300	-0.37440900

C	-1.42189900	-0.11665400	0.03143100
H	-1.42140600	2.00089900	0.37437100
C	-2.86003500	-0.28231400	0.05676000
C	-0.58873100	-1.28118500	-0.14859400
H	-0.56371100	-3.44082200	-0.37439900
C	0.80389100	-1.12803700	-0.19224900
C	-3.39098700	-1.55212400	-0.00737500
H	-4.46590800	-1.69261600	0.02029100
C	-2.55790700	-2.69734200	-0.14907100
H	-3.02053500	-3.67861100	-0.20485800
C	-1.19938900	-2.56924700	-0.24554900
N	-3.66430600	0.88689900	0.16853700
C	-3.75664900	1.67084900	-1.06763700
H	-4.15603600	2.66548600	-0.84198600
H	-4.41707300	1.19261600	-1.81195200
H	-2.76987300	1.78925700	-1.51718600
C	-4.97014000	0.71710700	0.78843700
H	-4.86912200	0.14528800	1.71397300
H	-5.70734900	0.20823000	0.14120500
H	-5.37584300	1.70450100	1.03287900
N	3.66434000	-0.88688900	-0.16844500
C	4.97008700	-0.71705900	-0.78850200
H	5.37584800	-1.70444500	-1.03288200
H	5.70732100	-0.20804600	-0.14140300
H	4.86892000	-0.14534600	-1.71408800
C	3.75689200	-1.67071400	1.06778800
H	2.77020800	-1.78904700	1.51755200
H	4.41747500	-1.19242300	1.81192600
H	4.15621200	-2.66538700	0.84216900

Zero-point correction= 0.341237 (Hartree/Particle)
Thermal correction to Energy= 0.358704
Thermal correction to Enthalpy= 0.359648
Thermal correction to Gibbs Free Energy= 0.297024
Sum of electronic and zero-point Energies= -807.182341
Sum of electronic and thermal Energies= -807.164874
Sum of electronic and thermal Enthalpies= -807.163929
Sum of electronic and thermal Free Energies= -807.226553

	E (Thermal)	CV	S
	KCal/Mol	Cal/Mol-Kelvin	Cal/Mol-Kelvin
Total	225.090	70.589	131.803

1.6. 1,5-di(pyrrolidin-1-yl)anthracene 1d

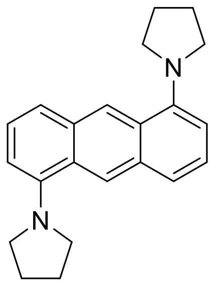

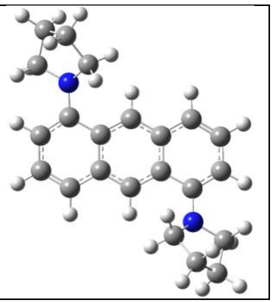

0 1

C	3.03867900	2.16219700	-0.48298000
C	2.76917600	0.81820700	-0.27531400
C	1.38358700	0.38099300	-0.31714900
C	0.33809400	1.37466000	-0.39800800
C	0.68258900	2.75070400	-0.55152700
C	1.99865200	3.11717400	-0.62630900
C	1.00236100	-0.96359800	-0.37624200
C	-1.00235300	0.96363300	-0.37630900
C	-1.38359000	-0.38095700	-0.31716000
C	-0.33808400	-1.37463100	-0.39795800
C	-0.68255100	-2.75067900	-0.55146700
H	0.11213500	-3.48695900	-0.63078400
C	-1.99861000	-3.11715100	-0.62633200
C	-3.03865400	-2.16219600	-0.48307100
C	-2.76918800	-0.81819100	-0.27535200
H	1.76093300	-1.73000900	-0.46722600
H	4.06106400	2.51679300	-0.50756200
H	-0.11208700	3.48698900	-0.63090800
H	2.26835200	4.16015700	-0.76761800
H	-1.76089600	1.73006500	-0.46731600
H	-2.26829700	-4.16013500	-0.76766300
H	-4.06102700	-2.51681100	-0.50775700
C	-5.18778600	-0.30226700	-0.26599100
C	-3.72189600	1.12978400	1.00417900
C	-5.99629500	0.86671800	0.29445200
H	-5.42728300	-1.21200600	0.31100900
H	-5.38365800	-0.51862500	-1.32174900
C	-5.16184900	1.24493200	1.52622500
H	-3.39421200	2.09978300	0.61209700
H	-3.00580900	0.83037400	1.77615300
H	-6.01988400	1.69544900	-0.42233000
H	-7.02653300	0.58812300	0.53156700
H	-5.38251600	2.24067600	1.92070600
H	-5.33650900	0.51808700	2.32807500
C	5.18772700	0.30195200	-0.26629300
C	3.72188200	-1.12930600	1.00480700

C	5.99614300	-0.86710400	0.29412400
H	5.42754300	1.21170900	0.31055700
H	5.38337800	0.51815900	-1.32212000
C	5.16202900	-1.24479500	1.52628800
H	3.39356500	-2.09934600	0.61338800
H	3.00628900	-0.82916800	1.77696500
H	6.01925200	-1.69599900	-0.42248300
H	7.02653700	-0.58872900	0.53082400
H	5.38254100	-2.24052200	1.92089600
H	5.33724400	-0.51784100	2.32791700
N	-3.79421600	0.11491600	-0.07861600
N	3.79414500	-0.11501400	-0.07857100

Zero-point correction= 0.414861 (Hartree/Particle)
Thermal correction to Energy= 0.434308
Thermal correction to Enthalpy= 0.435252
Thermal correction to Gibbs Free Energy= 0.366018
Sum of electronic and zero-point Energies= -961.966795
Sum of electronic and thermal Energies= -961.947347
Sum of electronic and thermal Enthalpies= -961.946403
Sum of electronic and thermal Free Energies= -962.015638

	E (Thermal)	CV	S
	KCal/Mol	Cal/Mol-Kelvin	Cal/Mol-Kelvin
Total	272.533	79.427	145.717

1.7. 1-(anthracen-1-yl)pyrrolidine 1e

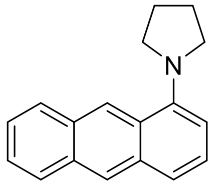

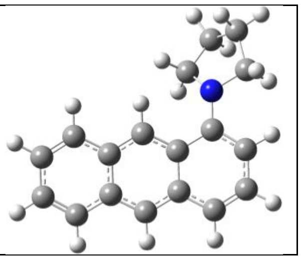

0 1

C	-4.77056900	-0.96774700	0.06393900
C	-4.17125400	0.25036400	0.25029600
C	-2.75471000	0.39630000	0.12799200
C	-1.96369300	-0.76647000	-0.18618800
C	-2.62821600	-2.01581000	-0.38219000
C	-3.98957000	-2.11615700	-0.25916800
C	-2.10613300	1.62776700	0.26696700
C	-0.57139100	-0.63947900	-0.28993600
C	0.08886500	0.57896500	-0.09364100
C	-0.71775300	1.76100600	0.13679900
C	-0.09456800	3.04458300	0.17214200
H	-0.71095500	3.92444500	0.33095700

C	1.25283200	3.15073000	-0.03556400
C	2.06684400	2.00082500	-0.20529300
C	1.53505200	0.72099000	-0.17664600
H	-2.70013600	2.51920000	0.45553800
H	-5.84815100	-1.06542600	0.15872300
H	-4.76509600	1.12841400	0.49089000
H	-2.02957500	-2.88997600	-0.62601500
H	-4.48234800	-3.07268900	-0.40640300
H	-0.00509100	-1.51839400	-0.56742700
H	1.72879800	4.12740000	-0.04287300
H	3.13278700	2.14753100	-0.32108900
C	3.77553300	-0.24713500	-0.57923700
C	2.17281800	-1.57517500	0.63781700
C	4.37312700	-1.62681800	-0.30455900
H	4.25539100	0.48867000	0.08799900
H	3.90792100	0.10243000	-1.60875900
C	3.60025600	-2.04303000	0.95401100
H	1.62479700	-2.38406500	0.13937400
H	1.60342300	-1.29367100	1.52910300
H	4.15624900	-2.31126800	-1.13261700
H	5.45651700	-1.59205400	-0.16282100
H	3.64848000	-3.11405100	1.16920700
H	3.99371100	-1.50519900	1.82438000
N	2.34978100	-0.41336300	-0.27191500

Zero-point correction= 0.304804 (Hartree/Particle)
Thermal correction to Energy= 0.319161
Thermal correction to Enthalpy= 0.320105
Thermal correction to Gibbs Free Energy= 0.263314
Sum of electronic and zero-point Energies= -750.667726
Sum of electronic and thermal Energies= -750.653369
Sum of electronic and thermal Enthalpies= -750.652425
Sum of electronic and thermal Free Energies= -750.709215

	E (Thermal)	CV	S
	KCal/Mol	Cal/Mol-Kelvin	Cal/Mol-Kelvin
Total	200.276	59.972	119.526

1.8. Dimethyl 1,4-dihydro-1,4-ethenoanthracene-2,3-dicarboxylate 3aD

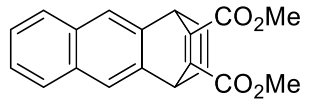

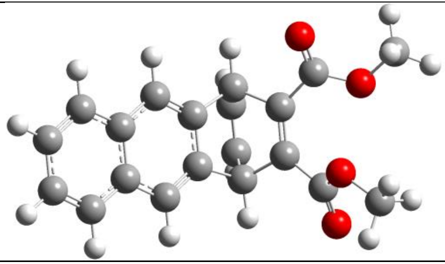

0 1

O	-2.04480700	2.52000100	-1.02458500
O	-3.64262000	1.04688700	-0.39109600
O	-2.57265800	-2.67816500	0.58668800
O	-2.59987600	-1.38944400	-1.27320600
C	4.29213100	1.32922100	-0.94691500
C	5.39010100	0.58316200	-1.32016500
C	5.41457500	-0.81128100	-1.08921100
C	4.34049400	-1.43416800	-0.48941400
C	3.19699400	-0.69540900	-0.09356500
C	3.17226600	0.71910800	-0.32753400
C	2.02419900	1.47199200	0.06647000
C	0.97053900	0.84089000	0.66563500
C	0.99386400	-0.56430700	0.89645500
C	2.07188700	-1.31919100	0.52699400
C	-0.33330300	1.47936800	1.15601300
C	-0.45051800	1.09457500	2.63534400
C	-0.43362600	-0.22153300	2.84662000
C	-0.29627000	-1.05063700	1.56550400
C	-1.45012600	0.72706700	0.41755700
C	-1.41130400	-0.60458400	0.60900400
C	-2.38047400	1.51926400	-0.42108800
C	-4.57037300	1.73822900	-1.24371100
C	-2.26150900	-1.65765200	0.00307900
C	-3.47437000	-2.35154400	-1.88595800
H	6.24011300	1.06561000	-1.79364000
H	6.28336100	-1.39123000	-1.38645800
H	4.35679200	-2.50638300	-0.31106700
H	2.00041100	2.54276700	-0.11884600
H	2.08717000	-2.39256700	0.69944000
H	-0.37727400	2.55041400	0.96413300
H	-0.51646400	1.85478300	3.40483600
H	-0.48485300	-0.70483000	3.81513100
H	-4.26664300	1.64870000	-2.29030800
H	-4.62036900	2.79808100	-0.98138600
H	-5.53364600	1.25523200	-1.08133900
H	-4.42641000	-2.39820200	-1.35018100
H	-3.01879700	-3.34501300	-1.88107000
H	-3.62502700	-2.00200800	-2.90703100
H	-0.32018300	-2.12644000	1.73295600
H	4.27047300	2.40143600	-1.12424700

Zero-point correction= 0.314353 (Hartree/Particle)
Thermal correction to Energy= 0.334391
Thermal correction to Enthalpy= 0.335336
Thermal correction to Gibbs Free Energy= 0.264491
Sum of electronic and zero-point Energies= -1072.388842
Sum of electronic and thermal Energies= -1072.368803

Sum of electronic and thermal Enthalpies= -1072.367859
Sum of electronic and thermal Free Energies= -1072.438703

	E (Thermal)	CV	S
	KCal/Mol	Cal/Mol-Kelvin	Cal/Mol-Kelvin
Total	209.834	77.601	149.104

1.9. Dimethyl 9,10-dihydro-9,10-ethenoanthracene-11,12-dicarboxylate 2aD

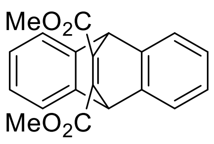

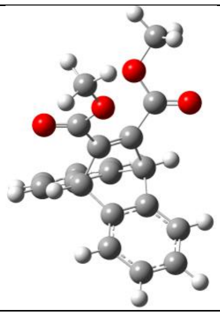

0 1

O	1.83348400	0.01683300	2.73167700
O	2.84865600	-0.99437400	0.97975700
O	1.83350700	-0.01702000	-2.73159700
O	2.84868800	0.99430200	-0.97976600
C	-2.05979400	-2.44832500	1.01023000
C	-2.67652400	-3.35369500	0.13485300
C	-2.67141200	-3.12079700	-1.23981400
C	-2.05045600	-1.97823400	-1.76438700
C	-1.44159400	-1.08450200	-0.89541300
C	-1.44547300	-1.31931200	0.48785200
C	-0.73018900	-0.21643300	1.26997600
C	0.67639000	-0.12901400	0.66088700
C	0.67642200	0.12893500	-0.66081000
C	-0.73013500	0.21643300	-1.26995400
C	-1.44537200	1.31935800	-0.48785000
C	-1.44155100	1.08454700	0.89541500
C	1.83273900	-0.33178700	1.56681800
C	4.01807400	-1.16249400	1.79834900
C	1.83278800	0.33166000	-1.56675800
C	4.01806600	1.16247900	-1.79841500
C	-2.05960900	2.44840700	-1.01025200
C	-2.67631300	3.35381400	-0.13489700
C	-2.67126000	3.12091600	1.23977200
C	-2.05039100	1.97831900	1.76436600
H	-3.15892100	-4.24197600	0.53187300
H	-3.14919200	-3.82867700	-1.91061800
H	-2.04172200	-1.79505400	-2.83506200
H	-0.69318900	-0.39161900	2.34488100
H	-0.69308400	0.39160400	-2.34485800

H	3.77201200	-1.70166600	2.71665600
H	4.44129200	-0.18988100	2.06394500
H	4.72036400	-1.73418600	1.19219700
H	3.77189600	1.70147100	-2.71679700
H	4.44141100	0.18988300	-2.06386900
H	4.72029300	1.73435900	-1.19236800
H	-3.15864600	4.24212400	-0.53193000
H	-3.14902100	3.82882700	1.91055900
H	-2.04170300	1.79513700	2.83504200
H	-2.06156800	-2.62669900	2.08200800
H	-2.06133400	2.62677400	-2.08203100

Zero-point correction= 0.315100 (Hartree/Particle)
Thermal correction to Energy= 0.335118
Thermal correction to Enthalpy= 0.336062
Thermal correction to Gibbs Free Energy= 0.265390
Sum of electronic and zero-point Energies= -1072.408573
Sum of electronic and thermal Energies= -1072.388555
Sum of electronic and thermal Enthalpies= -1072.387611
Sum of electronic and thermal Free Energies= -1072.458284

	E (Thermal)	CV	S
	KCal/Mol	Cal/Mol-Kelvin	Cal/Mol-Kelvin
Total	210.290	76.981	148.743

1.10. Dimethyl 1,5-dimethoxy-1,4-dihydro-1,4-ethenoanthracene-2,3-dicarboxylate 3bD

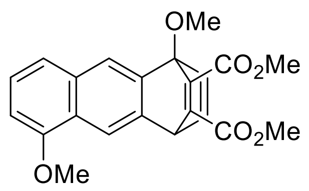

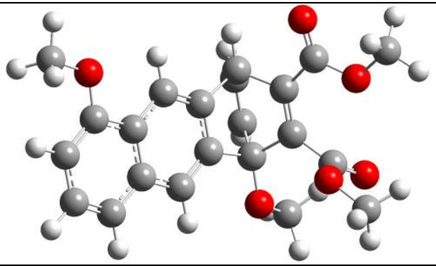

0 1

O	-1.72637100	3.36997800	0.54438800
O	-3.23587400	2.11696300	-0.58298200
O	-4.04863700	-1.08912700	-0.47824800
O	-2.48642600	-0.41671400	-1.96965200
C	4.20118800	0.47593800	-0.29057600
C	5.10210500	-0.32284000	-0.97234100
C	4.72423400	-1.62436300	-1.37634700
C	3.46945900	-2.11682000	-1.10384600
C	2.52459700	-1.31903200	-0.40662900
C	2.88718600	0.00043200	0.01139800
C	1.94190400	0.80757900	0.71110900

C	0.69683400	0.30868200	0.97821900
C	0.33690600	-1.00087100	0.56228700
C	1.21364800	-1.80123300	-0.11283400
C	-0.45154200	1.01895800	1.70413700
C	-0.83726700	0.09284400	2.85722500
C	-1.17174500	-1.13926100	2.47578000
C	-1.10303900	-1.36593800	0.95755200
C	-1.60989500	0.99667400	0.70002900
C	-1.94806000	-0.23847900	0.29898400
C	-2.16711400	2.28032600	0.22964500
C	-3.81396900	3.33090500	-1.08773600
C	-2.96014600	-0.61653000	-0.72992500
C	-3.39748800	-0.75714000	-3.03006700
H	6.09837600	0.03321800	-1.20312700
H	5.44592200	-2.23582200	-1.91014100
H	3.18467400	-3.11688500	-1.41724200
H	2.22306600	1.80781700	1.01936100
H	0.91745100	-2.79736900	-0.42360800
H	-0.20594300	2.03527500	2.00677900
H	-0.79827800	0.43348600	3.88532800
H	-1.43961100	-1.94985000	3.14069400
H	-3.08592800	3.88237700	-1.68866400
H	-4.14674900	3.96946300	-0.26537700
H	-4.66075500	3.01979900	-1.69907200
H	-4.29396000	-0.13394000	-2.97289900
H	-3.69154700	-1.80753600	-2.96224200
H	-2.85438800	-0.56688400	-3.95516600
O	4.46638700	1.74380300	0.14357800
C	5.74648100	2.29375900	-0.12183000
H	6.54476300	1.70769300	0.35240300
H	5.73700200	3.29862000	0.30306100
H	5.94301400	2.35846200	-1.20010600
O	-1.42047900	-2.65028200	0.48544200
C	-2.52783300	-3.30930900	1.10101600
H	-2.82823500	-4.10191500	0.41271700
H	-3.37466300	-2.63279000	1.24835500
H	-2.24164900	-3.76589000	2.05724900

Zero-point correction= 0.379029 (Hartree/Particle)
Thermal correction to Energy= 0.404401
Thermal correction to Enthalpy= 0.405345
Thermal correction to Gibbs Free Energy= 0.323173
Sum of electronic and zero-point Energies= -1301.377999
Sum of electronic and thermal Energies= -1301.352627
Sum of electronic and thermal Enthalpies= -1301.351683
Sum of electronic and thermal Free Energies= -1301.433855

	E (Thermal)	CV	S
	KCal/Mol	Cal/Mol-Kelvin	Cal/Mol-Kelvin
Total	253.765	96.392	172.945

1.11. Dimethyl 1,5-dimethoxy-9,10-dihydro-9,10-ethenoanthracene-11,12-dicarboxylate 2bD

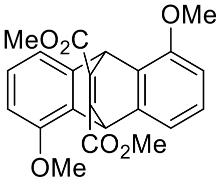

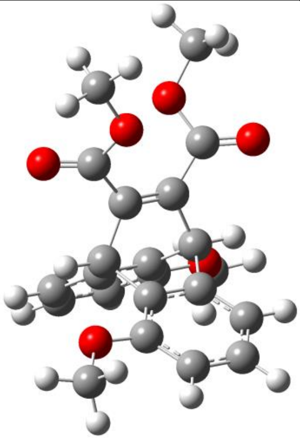

0 1

O	-1.71337600	2.05613200	-2.31563400
O	-1.74795200	3.03849900	-0.24102800
O	1.16788400	2.40470200	2.32031400
O	1.00695200	3.35781500	0.23759200
C	-2.39625600	-1.97836200	0.46546100
C	-2.62770700	-2.62714200	1.69130300
C	-1.69224700	-2.51032400	2.72745200
C	-0.52165400	-1.75498200	2.56724800
C	-0.30703300	-1.11827800	1.34581300
C	-1.23313800	-1.22609600	0.30099200
C	-0.81761400	-0.46113200	-0.95989000
C	-0.58188700	0.98706900	-0.49207600
C	0.33861400	1.09592000	0.49041200
C	0.90455500	-0.25760900	0.95955900
C	1.48617400	-0.90690500	-0.30032800
C	0.56071600	-1.01716900	-1.34538900
C	-1.36793700	2.05751100	-1.12499700
C	-2.46549500	4.18502500	-0.81420600
C	0.84955800	2.32102300	1.12517400
C	1.42192000	4.64527400	0.81013400
C	2.79159500	-1.37027700	-0.46397200
C	3.16772200	-1.94741700	-1.68985400
C	2.23152300	-2.04948200	-2.72685100
C	0.91787700	-1.58573200	-2.56708500
H	-3.52340700	-3.21590900	1.84569600
H	-1.88359500	-3.01373500	3.66980700
H	0.19867600	-1.66362200	3.37267600

H	-1.55014900	-0.52208500	-1.76044300
H	1.63090600	-0.14648800	1.76038000
H	-3.34341500	3.84389400	-1.36739900
H	-1.80531900	4.73594400	-1.48906000
H	-2.74831000	4.79320700	0.04293300
H	2.33583300	4.52269600	1.39562400
H	0.62957000	5.03589000	1.45395500
H	1.58474000	5.29277900	-0.04938600
H	4.17541100	-2.31292500	-1.84389000
H	2.53489200	-2.49417200	-3.66933200
H	0.19653600	-1.66287200	-3.37307800
O	-3.25667100	-2.02480500	-0.62925300
C	-4.48614100	-2.78760200	-0.51790700
H	-4.28034000	-3.84876900	-0.33128100
H	-4.98239700	-2.67244500	-1.48103000
H	-5.12816400	-2.39323700	0.27929100
O	3.63912300	-1.21763300	0.63127000
C	5.01292600	-1.67088500	0.51727200
H	5.06122700	-2.75018300	0.32751400
H	5.46950400	-1.44531600	1.48048300
H	5.54369600	-1.13491600	-0.27913300

Zero-point correction= 0.381556 (Hartree/Particle)
Thermal correction to Energy= 0.407062
Thermal correction to Enthalpy= 0.408006
Thermal correction to Gibbs Free Energy= 0.324748
Sum of electronic and zero-point Energies= -1300.992979
Sum of electronic and thermal Energies= -1300.967473
Sum of electronic and thermal Enthalpies= -1300.966529
Sum of electronic and thermal Free Energies= -1301.049787

	E (Thermal)	CV	S
	KCal/Mol	Cal/Mol-Kelvin	Cal/Mol-Kelvin
Total	255.435	95.193	175.233

1.12. Dimethyl 1,5-bis(dimethylamino)-1,4-dihydro-1,4-ethenoanthracene-2,3-dicarboxylate 3cD

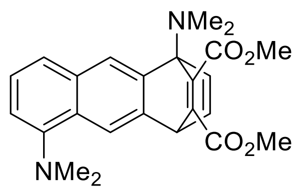

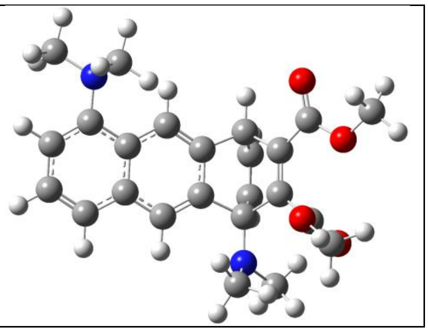

0 1

O	-1.50578800	3.48353500	0.60505300
O	-3.29846400	2.41491700	-0.26548900
O	-4.25358800	-0.79647600	-0.25596600
O	-2.92200100	0.03970400	-1.88455800
N	4.58580600	1.54176900	-0.02009000
N	-1.74197300	-2.62676600	0.45176500
C	4.21815400	0.19044900	-0.27710600
C	5.11041000	-0.74615200	-0.77687700
C	4.70549400	-2.07347500	-1.03787000
C	3.40318000	-2.46415400	-0.82592600
C	2.45954200	-1.54642800	-0.29776600
C	2.87264900	-0.21240200	0.01562400
C	5.99572200	1.79212600	0.23913500
C	4.03507800	2.50910700	-0.97477300
C	1.94405300	0.65516600	0.66418300
C	0.66211400	0.23985900	0.89697000
C	0.21864900	-1.04974400	0.49070000
C	1.10906800	-1.93809800	-0.04865900
C	-0.40925700	1.00862800	1.67095300
C	-0.82377900	0.06838900	2.80444300
C	-1.25190700	-1.12465400	2.39408000
C	-1.26511100	-1.32236400	0.86338800
C	-1.75673900	-2.91975600	-0.98014800
C	-2.90293100	-3.18434800	1.15376400
C	-1.59676600	1.10686600	0.71720400
C	-2.04137500	-0.08485500	0.28911600
C	-2.09792200	2.44994900	0.35593400
C	-3.81857700	3.69273700	-0.66515700
C	-3.20325500	-0.30710300	-0.61425700
C	-4.00900300	-0.12570700	-2.81057800
H	6.13274500	-0.45829000	-0.99514300
H	5.43016900	-2.77978600	-1.43282700
H	3.08399700	-3.47867900	-1.04812400
H	6.63152900	1.73461300	-0.66303000
H	6.37118400	1.07468100	0.97284800
H	6.10487300	2.79986800	0.65329900
H	4.54981900	2.46673900	-1.95060800
H	2.97419400	2.31838000	-1.14185500
H	4.14309100	3.52191700	-0.57236800
H	2.28354200	1.62457100	1.01172400
H	0.80933300	-2.95582400	-0.27306700
H	-0.07549600	1.99125600	1.99978500
H	-0.71045400	0.35966700	3.84187200
H	-1.51507200	-1.94782400	3.04531600
H	-1.03085200	-2.30296000	-1.51048900
H	-2.74234400	-2.74700000	-1.43939800

H	-1.49724300	-3.97190200	-1.15217300
H	-3.70400000	-3.42306900	0.44614700
H	-3.33292600	-2.46940600	1.85785500
H	-2.64042900	-4.10127600	1.69850700
H	-3.14687800	4.17470900	-1.38062000
H	-3.93679900	4.34774500	0.20179500
H	-4.78480800	3.48510200	-1.12437900
H	-4.85902600	0.49361600	-2.51232700
H	-4.32952100	-1.17063800	-2.84654500
H	-3.62169100	0.19110800	-3.77849300

Zero-point correction= 0.459794 (Hartree/Particle)
Thermal correction to Energy= 0.487443
Thermal correction to Enthalpy= 0.488387
Thermal correction to Gibbs Free Energy= 0.402274
Sum of electronic and zero-point Energies= -1340.191242
Sum of electronic and thermal Energies= -1340.163593
Sum of electronic and thermal Enthalpies= -1340.162649
Sum of electronic and thermal Free Energies= -1340.248762

	E (Thermal)	CV	S
	KCal/Mol	Cal/Mol-Kelvin	Cal/Mol-Kelvin
Total	305.875	106.603	181.240

1.13. Dimethyl 1,5-bis(dimethylamino)-9,10-dihydro-9,10-ethenoanthracene-11,12-dicarboxylate 2cD

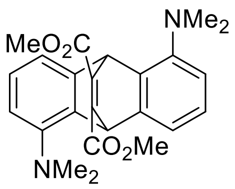

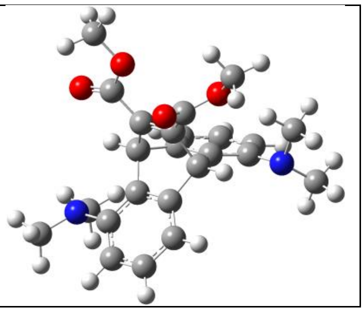

0 1

O	-1.45809900	2.87421700	-1.79649500
O	0.54348800	3.52573100	-0.96910900
O	1.47440800	2.88914400	1.79510700
O	3.01402200	1.67515900	0.66607800
N	-3.98905600	-0.31654200	-0.12273700
N	2.79765000	-2.56371400	0.25464000
C	-2.98481500	-0.56066900	0.85424200
C	-3.27401100	-0.98241800	2.16746000
C	-2.25484900	-1.23047900	3.08594300
C	-0.91191000	-1.08587700	2.72221900
C	-0.62330400	-0.65704900	1.43314300
C	-1.63951200	-0.37172200	0.51654000

C	-1.09673700	0.15328400	-0.81625200
C	-0.19671300	1.34419900	-0.47654400
C	0.77538100	1.05577100	0.40603200
C	0.77381400	-0.41498800	0.85503400
C	0.85138300	-1.25577900	-0.42376400
C	-0.17257600	-0.95189800	-1.32934900
C	-0.45952300	2.63734900	-1.14325500
C	0.31813600	4.82410400	-1.53930700
C	1.74890400	1.99996000	1.02295700
C	4.04347700	2.48790400	1.25893500
C	1.76595600	-2.28085400	-0.68930300
C	1.60624500	-3.00433500	-1.88809900
C	0.57975700	-2.70264300	-2.78121600
C	-0.31811900	-1.66300200	-2.51395200
C	-5.30579900	0.05106300	0.37370300
C	-4.06537500	-1.33316700	-1.17314700
C	3.91513200	-1.61636100	0.21437900
C	3.26343300	-3.94274800	0.29841800
H	-4.30553900	-1.12781000	2.46903600
H	-2.51028300	-1.55495000	4.09066600
H	-0.11439200	-1.29334300	3.42926100
H	-1.87930800	0.44096600	-1.51412900
H	1.56729000	-0.65134100	1.56142500
H	-0.58311500	5.27715400	-1.11831900
H	0.20243000	4.75283600	-2.62417600
H	1.19831300	5.41325200	-1.28304600
H	4.00593200	2.42085800	2.34927800
H	3.91822600	3.53309700	0.96561900
H	4.98500800	2.09158300	0.87973200
H	2.29760200	-3.80545000	-2.12564900
H	0.48549600	-3.27569000	-3.69924400
H	-1.11557800	-1.41884200	-3.20960100
H	-5.21138800	0.82881700	1.13539000
H	-5.87212500	-0.79480200	0.80455300
H	-5.89638500	0.45351200	-0.45582400
H	-4.55428900	-2.25992600	-0.82315700
H	-3.06662200	-1.59847700	-1.52384000
H	-4.63760300	-0.94220400	-2.02138700
H	3.54812800	-0.58988500	0.19641400
H	4.55684700	-1.76714700	-0.67226400
H	4.53414600	-1.74682200	1.10913600
H	3.90737900	-4.22368800	-0.55462800
H	2.40911100	-4.62349600	0.32599200
H	3.84976300	-4.08828600	1.21189700

Zero-point correction= 0.461286 (Hartree/Particle)
Thermal correction to Energy= 0.488767
Thermal correction to Enthalpy= 0.489712

Thermal correction to Gibbs Free Energy= 0.404135
Sum of electronic and zero-point Energies= -1340.223153
Sum of electronic and thermal Energies= -1340.195672
Sum of electronic and thermal Enthalpies= -1340.194728
Sum of electronic and thermal Free Energies= -1340.280304

	E (Thermal)	CV	S
	KCal/Mol	Cal/Mol-Kelvin	Cal/Mol-Kelvin
Total	306.706	105.417	180.110

1.14. Dimethyl 1,5-di(pyrrolidin-1-yl)-1,4-dihydro-1,4-ethenoanthracene-2,3-dicarboxylate 3dD

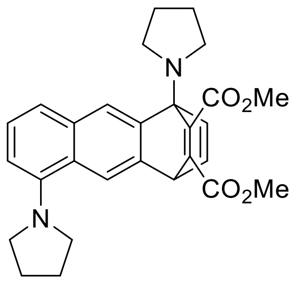

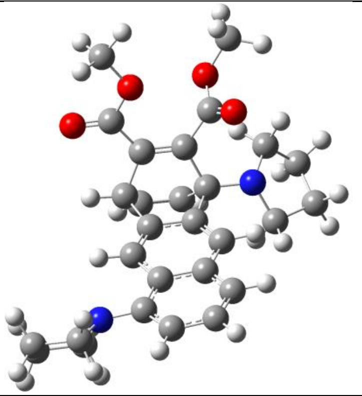

0 1

C	4.37129100	-1.53724400	-1.74216900
C	3.89036100	-0.70046100	-0.74506700
C	2.49276400	-0.73130100	-0.42111500
C	1.64941100	-1.69622700	-1.05790200
C	2.19206900	-2.55161900	-2.05073200
C	3.51956700	-2.45145600	-2.39934800
C	1.89745600	0.21855800	0.46169200
C	0.26647300	-1.75608700	-0.70881200
C	-0.24650500	-0.90288400	0.22970700
C	0.56809700	0.13168500	0.77045300
C	-0.24136100	1.14846100	1.57797400
C	-1.33624600	1.60385100	0.60373700
C	-2.10169900	0.60283300	0.13019000
C	-1.71981700	-0.79929900	0.69259900
H	5.42027600	-1.50959500	-2.01428700
H	1.54075400	-3.27024700	-2.54022400
H	3.93083900	-3.09453200	-3.17223500
H	2.49742400	1.04415900	0.82873700
H	-0.37637100	-2.45917800	-1.22544200
C	-2.18886500	-3.23430000	0.71157000
C	-4.02752900	-1.70071800	0.47867300
C	-3.48931300	-4.03382800	0.63098100
H	-1.81532600	-3.22437800	1.74947300

H	-1.39554200	-3.66288600	0.09626000
C	-4.52317200	-3.00954100	1.11318500
H	-4.53116200	-1.51593800	-0.47789600
H	-4.22567800	-0.83498000	1.12159700
H	-3.68971500	-4.31818100	-0.40824500
H	-3.45879500	-4.94526700	1.23442000
H	-5.55102800	-3.25201600	0.82923800
H	-4.48862000	-2.93736700	2.20706600
C	4.99471700	-0.07032300	1.37319900
C	6.01710000	0.57524800	-0.60861200
C	5.94448000	1.07215700	1.78137500
H	5.49433800	-1.04979700	1.48039200
H	4.07358200	-0.10523700	1.95724900
C	6.58160800	1.54605400	0.44257700
H	5.89512300	1.01609300	-1.60211500
H	6.68895600	-0.29836800	-0.70495100
H	5.38782300	1.88707700	2.25226500
H	6.69121300	0.72675700	2.50157100
H	6.27121700	2.56914200	0.21419300
H	7.67470200	1.52805800	0.46322900
N	-2.57014800	-1.88669400	0.23787900
N	4.71876700	0.20927100	-0.04787900
C	-0.92900100	0.36029900	2.68569500
C	-1.68726500	-0.63278300	2.23058900
H	0.35253700	1.99021600	1.92962800
H	-0.78381400	0.61404500	3.72980700
H	-2.26614700	-1.31044800	2.84738400
C	-3.11464500	0.72144300	-0.95884000
C	-1.36773500	3.03052500	0.22208900
O	-2.95667600	0.29055900	-2.07767000
O	-4.24304100	1.32000200	-0.52252100
O	-0.73818400	3.89844900	0.79915000
O	-2.15447400	3.27822400	-0.84917600
C	-2.22649200	4.65491600	-1.25088800
H	-1.23287200	5.03384700	-1.50334600
H	-2.87691400	4.67108200	-2.12517700
H	-2.64345900	5.26933400	-0.44836000
C	-5.24883900	1.50876100	-1.53123700
H	-6.08148000	2.00081700	-1.02907400
H	-4.86179800	2.13369800	-2.34045000
H	-5.56286600	0.54785100	-1.94834500

Zero-point correction= 0.533999 (Hartree/Particle)
Thermal correction to Energy= 0.564083
Thermal correction to Enthalpy= 0.565028
Thermal correction to Gibbs Free Energy= 0.471701
Sum of electronic and zero-point Energies= -1494.977434
Sum of electronic and thermal Energies= -1494.947350

Sum of electronic and thermal Enthalpies= -1494.946406
Sum of electronic and thermal Free Energies= -1495.039733

	E (Thermal)	CV	S
	KCal/Mol	Cal/Mol-Kelvin	Cal/Mol-Kelvin
Total	353.968	116.701	196.423

1.15. Dimethyl 1,5-di(pyrrolidin-1-yl)-9,10-dihydro-9,10-ethenoanthracene-11,12-dicarboxylate 2dD

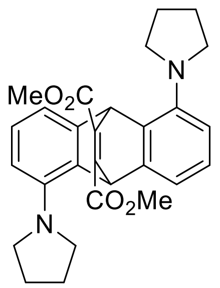

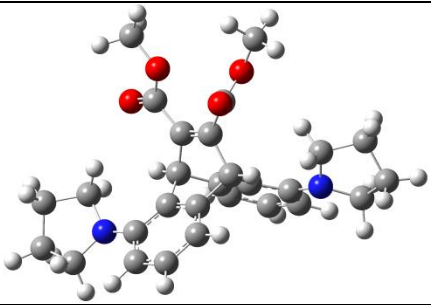

0 1

C	1.14934800	-2.37624800	-2.91329300
C	0.03391100	-1.61306400	-2.55433900
C	0.07836200	-0.93052900	-1.34680600
C	1.19747000	-0.99020300	-0.50922600
C	2.36448700	-1.66881000	-0.90674400
C	2.29014100	-2.40154100	-2.11710300
C	-1.06551400	-0.09479700	-0.76709100
C	0.91897000	-0.28466600	0.82539900
C	-0.35651700	-0.91414100	1.39154500
C	-1.46536500	-0.78907200	0.54580000
C	-2.71531400	-1.32729400	0.91916400
C	-2.75222800	-2.05494500	2.13763100
C	-1.63194600	-2.18625600	2.94964800
C	-0.41422800	-1.59866200	2.59720400
H	1.14150000	-2.94410400	-3.83955400
H	3.14062000	-2.98417000	-2.44828500
H	-3.67803700	-2.51362500	2.46064500
H	-1.71652300	-2.74535700	3.87737100
C	0.58479700	1.16112900	0.44950300
C	-0.47434900	1.26229100	-0.37368200
H	-1.86851000	0.02398300	-1.48366600
H	1.72997600	-0.34430400	1.54323300
C	1.43184200	2.23542000	1.03105500
C	-1.13023800	2.48998800	-0.85996800
O	-0.62381200	3.61595300	-0.30800800
O	-2.05842200	2.49870200	-1.65074100
O	2.14712500	2.87806000	0.08397000
O	1.54087900	2.43871400	2.22154600

C	-1.23217200	4.84011600	-0.74676700
H	-0.71532100	5.63395000	-0.20783200
H	-1.11183600	4.96534600	-1.82617000
H	-2.29943400	4.84633800	-0.50998300
C	2.99408200	3.93052400	0.57777000
H	3.70613800	3.54254000	1.31093600
H	3.51427700	4.32301000	-0.29556700
H	2.39223200	4.71111400	1.05063900
C	4.69277700	-2.44753000	-0.54719700
C	4.00149700	-0.37835600	0.48443300
C	5.82728200	-1.92342700	0.33416100
H	4.95619100	-2.29700300	-1.60798100
H	4.48218900	-3.51340800	-0.40625600
C	5.53131200	-0.41736400	0.35557900
H	3.72124600	-0.34533600	1.54531800
H	3.55588800	0.49601600	-0.00419600
H	5.75100500	-2.34247300	1.34396900
H	6.81462600	-2.17000100	-0.06525900
H	6.03205100	0.11878300	1.16648600
H	5.84364400	0.03552700	-0.59260000
C	-5.10983100	-1.86227300	0.54797300
C	-4.16416900	0.00154000	-0.66502000
C	-6.14667200	-1.32300400	-0.43938300
H	-5.40515900	-1.59728300	1.57762800
H	-4.99642300	-2.95177300	0.50752800
C	-5.69201700	0.13265800	-0.60755200
H	-3.84202100	-0.13779900	-1.70519000
H	-3.64838400	0.88771400	-0.28432000
H	-6.07592000	-1.85680400	-1.39404900
H	-7.16946000	-1.42430400	-0.06658100
H	-6.10057700	0.62442900	-1.49429200
H	-5.98807700	0.71802200	0.27087900
N	3.53978200	-1.63358100	-0.14984800
N	-3.86852900	-1.19278900	0.15014600
H	0.46202900	-1.68695900	3.23160500
H	-0.84715700	-1.56781200	-3.18684100

Zero-point correction= 0.535727 (Hartree/Particle)
Thermal correction to Energy= 0.565837
Thermal correction to Enthalpy= 0.566781
Thermal correction to Gibbs Free Energy= 0.472548
Sum of electronic and zero-point Energies= -1495.011341
Sum of electronic and thermal Energies= -1494.981231
Sum of electronic and thermal Enthalpies= -1494.980287
Sum of electronic and thermal Free Energies= -1495.074520

	E (Thermal)	CV	S
	KCal/Mol	Cal/Mol-Kelvin	Cal/Mol-Kelvin
Total	355.068	115.877	198.330

1.16. Dimethyl 5-(pyrrolidin-1-yl)-1,4-dihydro-1,4-ethanoanthracene-2,3-dicarboxylate

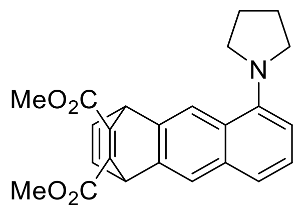

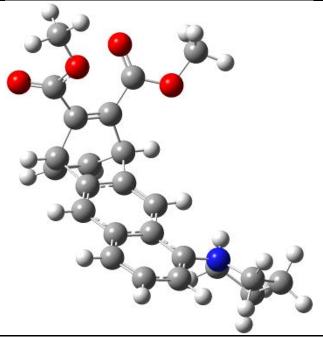

0 1

C	1.95517000	-0.69969400	2.79923100
C	2.01921300	-1.38899500	1.43151500
C	0.58806300	-1.36230300	0.88504000
C	0.06192800	-0.04434700	0.84253700
C	1.07400400	0.99298300	1.33982800
C	1.46224600	0.53775400	2.75265100
C	-0.14843500	-2.41433500	0.41928900
C	-1.20253000	0.19498800	0.37959900
C	-2.03164800	-0.88318900	-0.05847100
C	-1.46840300	-2.20378600	-0.08556600
C	-2.20101700	-3.27407500	-0.65565900
C	-3.44183900	-3.03844100	-1.20062500
C	-4.03115600	-1.76102200	-1.13535000
C	-3.38041300	-0.68800100	-0.52855000
H	0.26270500	-3.42078400	0.40779500
H	2.27458300	-1.21872400	3.69510900
H	2.45619100	-2.38627100	1.46333700
H	0.69270700	2.01121200	1.29545100
H	1.31154900	1.18642500	3.60773400
H	-1.55854600	1.21334700	0.30326700
H	-3.99606200	-3.84794600	-1.66738200
H	-5.02561400	-1.63492400	-1.54365900
C	-5.26107700	0.81646000	-1.11355200
C	-4.00812000	1.29196000	0.88929900
C	-5.75322200	2.13564400	-0.51984100
H	-6.01326500	0.03478600	-0.91231100
H	-5.09465600	0.84568200	-2.19572600
C	-5.39496700	1.94842700	0.96088900
H	-3.23347000	2.06808900	0.91051300
H	-3.81027100	0.60995100	1.72300900

H	-5.19789600	2.97898000	-0.94599200
H	-6.81940300	2.30214700	-0.69514200
H	-5.38602600	2.87930200	1.53470900
H	-6.11495900	1.26900800	1.43170100
N	-4.00014000	0.56548500	-0.40651000
C	2.32010700	0.79904000	0.45817700
C	2.79873900	-0.45820000	0.49411900
H	-1.75718200	-4.26506600	-0.68161900
C	2.93343600	1.92093300	-0.29096000
C	3.97381800	-1.01397400	-0.22470000
O	4.78394500	-1.74493300	0.31101000
O	4.00323700	-0.66563500	-1.52114600
O	4.10469900	2.01428000	-0.59646300
O	2.01254900	2.87492700	-0.57720600
C	2.52190600	4.01034600	-1.29505500
H	1.66901100	4.67250500	-1.44320400
H	3.30470700	4.51107400	-0.71886200
H	2.94035600	3.70059500	-2.25629600
C	5.18748200	-1.06019100	-2.23421400
H	6.05975600	-0.54586400	-1.82248500
H	5.34076400	-2.13996700	-2.16375900
H	5.02086200	-0.75896400	-3.26795400

Zero-point correction= 0.424540 (Hartree/Particle)
Thermal correction to Energy= 0.449641
Thermal correction to Enthalpy= 0.450585
Thermal correction to Gibbs Free Energy= 0.367413
Sum of electronic and zero-point Energies= -1283.685386
Sum of electronic and thermal Energies= -1283.660285
Sum of electronic and thermal Enthalpies= -1283.659341
Sum of electronic and thermal Free Energies= -1283.742513

	E (Thermal)	CV	S
	KCal/Mol	Cal/Mol-Kelvin	Cal/Mol-Kelvin
Total	282.154	97.056	175.052

1.17. Dimethyl 1-(pyrrolidin-1-yl)-1,4-dihydro-1,4-ethenoanthracene-2,3-dicarboxylate 3eD

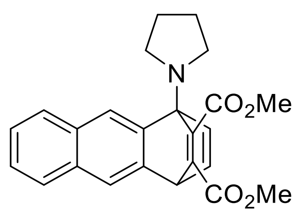

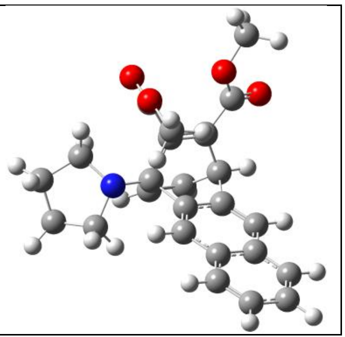

0 1

C	5.62223300	1.03673300	0.49618600
C	4.60881300	1.55188000	-0.28424300
C	3.34014200	0.92150800	-0.33779500
C	3.12375300	-0.26568700	0.43369300
C	4.18514500	-0.76934500	1.22771100
C	5.40849200	-0.13407000	1.25964200
C	2.26556500	1.43986100	-1.12046600
C	1.84999000	-0.90845000	0.38698100
C	0.85020500	-0.40275300	-0.40018800
C	1.06065100	0.79411800	-1.14439900
C	-0.20565500	1.25769700	-1.86106500
C	-0.63285500	0.09356500	-2.75162900
C	-0.83543900	-1.03923500	-2.08450700
C	-0.58855900	-0.96385100	-0.55590100
H	2.41123800	2.35831800	-1.68344500
H	6.58920700	1.53007300	0.52706500
H	4.76986100	2.45267000	-0.87081600
H	4.01805800	-1.67029600	1.81268900
H	6.21254800	-0.53190200	1.87165600
H	1.68993200	-1.79487200	0.98988700
H	-0.73662800	0.21912300	-3.82326400
H	-1.13842900	-1.98171500	-2.52497100
C	-2.19033600	-2.70914300	0.23247700
C	0.05825800	-3.34454300	-0.35312700
C	-2.08976600	-4.23547300	0.15976100
H	-2.79212100	-2.32260500	-0.60270900
H	-2.66672900	-2.37976300	1.15686500
C	-0.91212800	-4.44567900	-0.79578800
H	0.70060000	-3.71621600	0.45556500
H	0.71879000	-3.01314500	-1.15908500
H	-1.85066300	-4.64614600	1.14768300
H	-3.02280900	-4.69429200	-0.17781400
H	-0.46919900	-5.44417100	-0.73975400
H	-1.23485300	-4.28139700	-1.83128400
N	-0.78511100	-2.22145200	0.15714900
C	-1.46115600	0.23131000	-0.05925900
C	-1.23763500	1.37143700	-0.73860500
H	-0.08469500	2.20237600	-2.38870100
C	-2.52842500	0.09185700	0.99328800
C	-1.84227500	2.69375100	-0.47596800
O	-1.82379000	3.62020600	-1.26404900
O	-2.39159400	2.76903800	0.75570300
O	-3.69492300	0.00472400	0.68901000
O	-2.15541200	0.01818700	2.28389500
C	-0.76462000	0.16339200	2.62490300
H	-0.73652300	0.21069100	3.71382800

H	-0.19546000	-0.69828700	2.27067800
H	-0.35140000	1.08461500	2.20524100
C	-3.08819000	3.99045700	1.05288300
H	-2.40355700	4.84139200	1.00740500
H	-3.90020700	4.14976600	0.33906100
H	-3.48267300	3.86270200	2.06020100

Zero-point correction= 0.424439 (Hartree/Particle)
Thermal correction to Energy= 0.449152
Thermal correction to Enthalpy= 0.450096
Thermal correction to Gibbs Free Energy= 0.370417
Sum of electronic and zero-point Energies= -1283.674888
Sum of electronic and thermal Energies= -1283.650176
Sum of electronic and thermal Enthalpies= -1283.649231
Sum of electronic and thermal Free Energies= -1283.728910

	E (Thermal)	CV	S
	KCal/Mol	Cal/Mol-Kelvin	Cal/Mol-Kelvin
Total	281.847	97.322	167.698

1.18. Dimethyl 1-(pyrrolidin-1-yl)-9,10-dihydro-9,10-ethenoanthracene-11,12-dicarboxylate 2eD

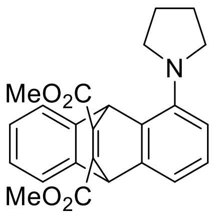

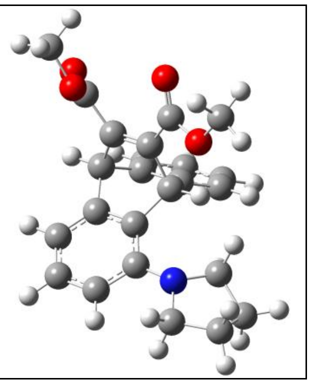

0 1

C	-0.93541000	4.13626100	1.67691100
C	-1.42474000	3.48727700	0.53368300
C	-0.99993200	2.19566300	0.26060500
C	-0.09758900	1.54519300	1.11391100
C	0.38830600	2.18373400	2.24605100
C	-0.03645700	3.49110900	2.52507800
C	-1.39788500	1.32887400	-0.93296300
C	0.25110700	0.13872700	0.62859000
C	0.82698300	0.31520100	-0.78875000
C	-0.09680700	0.93809100	-1.64022300
C	0.14834400	1.12300000	-2.99340400
C	1.36222200	0.65512800	-3.50321500

C	2.30595700	0.06278900	-2.67252200
C	2.08392500	-0.09613600	-1.28019300
H	-2.11629400	1.81581300	-1.59248100
H	-1.26302700	5.14667700	1.90290100
H	-2.12921800	3.98513400	-0.12647300
H	1.08414000	1.67503300	2.90811300
H	0.33387300	4.00032100	3.41004300
H	0.90003800	-0.39182000	1.31182300
H	1.58384500	0.76122700	-4.56144000
H	3.23774000	-0.27303000	-3.10930700
C	4.31811400	-1.15691500	-1.06269200
C	3.30114800	-0.23267100	0.92211500
C	5.18878300	-1.48761500	0.15086100
H	4.81686300	-0.38778300	-1.67641800
H	4.12425600	-2.02108300	-1.70804600
C	4.81880700	-0.35512700	1.11759800
H	2.78113100	-0.89806500	1.62488700
H	2.93451400	0.78639000	1.08903200
H	4.90199800	-2.45805000	0.57183700
H	6.25298900	-1.52315400	-0.09641900
H	5.09128700	-0.55546300	2.15742400
H	5.31501700	0.57262200	0.81027700
N	3.07270700	-0.65680000	-0.47243500
C	-1.08901100	-0.59681700	0.47620000
C	-1.94679200	0.02156800	-0.35546100
H	-0.58906500	1.59729400	-3.63330400
C	-1.40829100	-1.80986000	1.26437200
C	-3.33298400	-0.38000200	-0.70912400
O	-4.26538600	0.39873500	-0.68801100
O	-3.41551900	-1.65992900	-1.10583200
O	-2.52260200	-2.21165700	1.53228700
O	-0.27696500	-2.42757700	1.69098300
C	-0.49413600	-3.61065700	2.47562200
H	0.49835000	-3.97708900	2.73771700
H	-1.07001400	-3.37639800	3.37502700
H	-1.04070200	-4.36011300	1.89710500
C	-4.75005500	-2.14507000	-1.33065200
H	-5.30660400	-2.15147300	-0.38982300
H	-5.27482400	-1.51683200	-2.05472400
H	-4.63310000	-3.15958400	-1.71052000

Zero-point correction= 0.425496 (Hartree/Particle)
Thermal correction to Energy= 0.450568
Thermal correction to Enthalpy= 0.451513
Thermal correction to Gibbs Free Energy= 0.368809
Sum of electronic and zero-point Energies= -1283.708135
Sum of electronic and thermal Energies= -1283.683063

Sum of electronic and thermal Enthalpies= -1283.682119
Sum of electronic and thermal Free Energies= -1283.764823

	E (Thermal)	CV	S
	KCal/Mol	Cal/Mol-Kelvin	Cal/Mol-Kelvin
Total	282.736	96.424	174.065

1.19. (9R,10R,11S)-9,10-dihydro-9,10-[3,4]furanoanthracene-12,14-dione 2aB

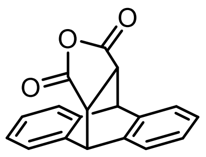
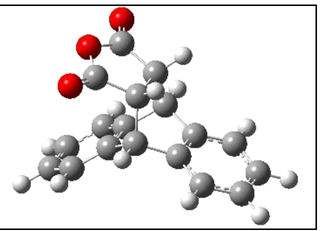

0 1

C	-1.94290800	2.89556900	-0.69730800
C	-1.19790800	1.94601700	-1.40538300
C	-0.44556800	1.01115200	-0.70272100
C	-0.44553200	1.01087000	0.70304500
C	-1.19779800	1.94543900	1.40613900
C	-1.94281900	2.89531400	0.69850800
C	0.37969700	-0.11322900	-1.30340600
C	0.37968500	-0.11380000	1.30325000
C	1.77268600	-0.05073300	0.70324100
C	1.77266100	-0.05049600	-0.70331600
C	2.97210600	-0.01367400	-1.40483200
H	2.96941000	-0.01479200	-2.49152600
C	4.17979100	0.03041300	-0.69768700
C	4.17980100	0.03017100	0.69763300
C	2.97212200	-0.01416700	1.40477500
H	-2.52955600	3.63172600	-1.23838300
H	-1.21003400	1.93085400	-2.49137100
H	-1.20980800	1.92985400	2.49212200
H	-2.52935900	3.63131100	1.23991700
H	0.37743400	-0.10248600	2.39486000
H	5.12024800	0.06612400	-1.23933500
H	5.12027200	0.06568200	1.23927000
H	2.96940000	-0.01572700	2.49146500
H	0.37751800	-0.10148400	-2.39500100
C	-0.24622600	-1.45256100	-0.77062800
C	-0.24615000	-1.45288900	0.76996200
C	-1.71374400	-1.57094400	-1.14863300
C	-1.71365300	-1.57173100	1.14803300
O	-2.19951800	-1.58912700	-2.24237400

O	-2.19911000	-1.59064300	2.24192700
O	-2.49398600	-1.64327100	-0.00018100
H	0.29384900	-2.30033600	-1.19956000
H	0.29407800	-2.30073100	1.19854000

Zero-point correction= 0.256480 (Hartree/Particle)
Thermal correction to Energy= 0.270788
Thermal correction to Enthalpy= 0.271732
Thermal correction to Gibbs Free Energy= 0.215089
Sum of electronic and zero-point Energies= -918.645259
Sum of electronic and thermal Energies= -918.630952
Sum of electronic and thermal Enthalpies= -918.630007
Sum of electronic and thermal Free Energies= -918.686650

	E (Thermal)	CV	S
	KCal/Mol	Cal/Mol-Kelvin	Cal/Mol-Kelvin
Total	169.922	59.971	119.215

1.20. (9R,10R,11S)-1,5-dimethoxy-9,10-dihydro-9,10-[3,4]furanoanthracene-12,14-dione 2bB

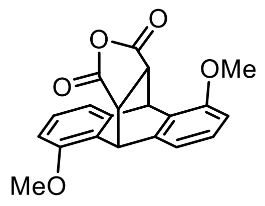

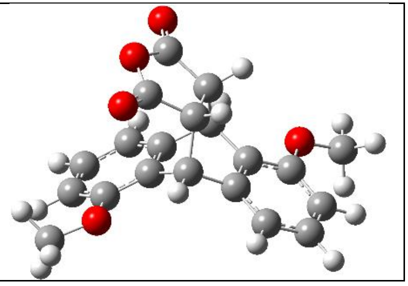

0 1

C	-2.63214200	0.91294300	2.10168500
C	-2.17495100	1.21388900	0.80994000
C	-1.00815300	0.59829600	0.34370800
C	-0.31679200	-0.31001900	1.15242500
C	-0.77172600	-0.62255600	2.43169800
C	-1.93012000	0.00208400	2.89589500
C	-0.41495600	0.76681400	-1.04277300
C	0.88287400	-0.92637000	0.45331100
C	1.74646000	0.19900900	-0.08423400
C	1.05545600	1.11317300	-0.88746700
C	1.71127100	2.19608500	-1.46868500
H	1.16744000	2.90145100	-2.08939100
C	3.07683700	2.35621600	-1.22756200
C	3.78280900	1.45162400	-0.42961400

C	3.11668900	0.35881600	0.14602000
H	-3.52916400	1.37809400	2.49151400
H	-0.23948600	-1.34248100	3.04483700
H	-2.30226200	-0.22267800	3.89100100
H	1.43225800	-1.62167000	1.08618500
H	3.60771800	3.19724300	-1.66381800
H	4.84187500	1.60500500	-0.26180800
H	-0.96988500	1.48217200	-1.64815900
C	-0.44643800	-0.65944500	-1.70223700
C	0.32357200	-1.66024000	-0.81944000
C	-1.86135100	-1.21378100	-1.75459000
C	-0.70864500	-2.71170300	-0.44841600
O	-2.81979100	-0.76114400	-2.31082100
O	-0.56791300	-3.68501000	0.23439400
O	-1.93615000	-2.39046700	-1.01666900
O	3.70860300	-0.58917500	0.93229000
O	-2.78458300	2.07598000	-0.05307100
C	-4.04999600	2.61110000	0.30863900
H	-4.37928600	3.20528700	-0.54469800
H	-4.78119900	1.81593500	0.50042600
H	-3.97886000	3.25828500	1.19264300
C	5.09686400	-0.46740400	1.20623500
H	5.35291700	-1.31473400	1.84374400
H	5.69609300	-0.51225100	0.28746700
H	5.32375900	0.46610500	1.73722100
H	-0.05736800	-0.59773400	-2.72186000
H	1.14875100	-2.15068000	-1.34152200

Zero-point correction= 0.321756 (Hartree/Particle)
Thermal correction to Energy= 0.341441
Thermal correction to Enthalpy= 0.342385
Thermal correction to Gibbs Free Energy= 0.273485
Sum of electronic and zero-point Energies= -1147.640325
Sum of electronic and thermal Energies= -1147.620640
Sum of electronic and thermal Enthalpies= -1147.619696
Sum of electronic and thermal Free Energies= -1147.688596

	E (Thermal)	CV	S
	KCal/Mol	Cal/Mol-Kelvin	Cal/Mol-Kelvin
Total	214.257	78.275	145.013

1.21. (9R,10R,11S)-1,5-bis(dimethylamino)-9,10-dihydro-9,10-[3,4]furanoanthracene-12,14-dione 2cB

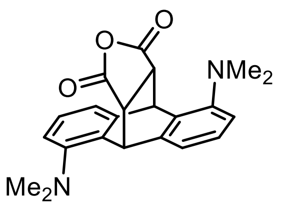	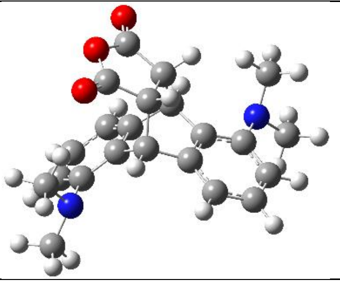

0 1

C	2.52289700	-0.94727600	2.21732700
C	2.28938400	-1.02713900	0.83246900
C	1.14589400	-0.38835700	0.32734400
C	0.25147700	0.26316000	1.18963200
C	0.49962400	0.34497500	2.55612300
C	1.64678100	-0.26768600	3.06358900
C	0.67229800	-0.39503600	-1.11630400
C	-0.99186500	0.79199600	0.49288200
C	-1.62178800	-0.36322800	-0.27027800
C	-0.71875700	-1.01229200	-1.12513000
C	-1.10214800	-2.13015200	-1.85875800
H	-0.39076300	-2.63338200	-2.50677400
C	-2.40963800	-2.60247300	-1.72189800
C	-3.32026000	-1.95027100	-0.89068100
C	-2.94702500	-0.80959600	-0.15530000
H	3.39986300	-1.42669500	2.63839200
H	-0.19776500	0.85881400	3.21040800
H	1.85884700	-0.22294900	4.12783600
H	-1.67746600	1.26647600	1.19319900
H	-2.72793600	-3.48206600	-2.27402300
H	-4.33350100	-2.32936000	-0.81753600
H	1.36429100	-0.91246800	-1.77944000
C	0.46288000	1.09883900	-1.56489900
C	-0.51209400	1.80162700	-0.60007900
C	1.72650300	1.93864200	-1.48662700
C	0.29499600	2.93744500	0.00200400
O	2.74778300	1.81110600	-2.10238500
O	-0.03609100	3.72810500	0.83684200
O	1.56828900	2.95813000	-0.56013800
N	-3.84692400	-0.11843100	0.70655100
N	3.13020600	-1.77728700	-0.04495100
C	3.75548200	-2.95511100	0.54471400
H	4.14106900	-3.58846700	-0.26110700

H	4.60245400	-2.72449300	1.21558300
H	3.01404300	-3.52716500	1.10794700
C	4.09831300	-0.97748000	-0.80110900
H	4.89953000	-0.57264900	-0.15824800
H	4.56160600	-1.60720800	-1.56795900
H	3.60986100	-0.14148500	-1.29908500
C	-4.95036700	-0.90055000	1.24770300
H	-5.40676900	-0.34327700	2.07211600
H	-5.74620900	-1.11595300	0.51238400
H	-4.57520800	-1.84845200	1.64062800
C	-4.30574700	1.17308500	0.19185000
H	-5.01435600	1.06382200	-0.64794700
H	-4.80422100	1.72900700	0.99243700
H	-3.46280500	1.77217200	-0.15673500
H	0.11829800	1.10667400	-2.60207800
H	-1.37672900	2.23164600	-1.11318400

Zero-point correction= 0.402972 (Hartree/Particle)
Thermal correction to Energy= 0.425532
Thermal correction to Enthalpy= 0.426477
Thermal correction to Gibbs Free Energy= 0.352254
Sum of electronic and zero-point Energies= -1186.459538
Sum of electronic and thermal Energies= -1186.436978
Sum of electronic and thermal Enthalpies= -1186.436034
Sum of electronic and thermal Free Energies= -1186.510257

	E (Thermal)	CV	S
	KCal/Mol	Cal/Mol-Kelvin	Cal/Mol-Kelvin
Total	267.026	90.286	156.215

1.22. (9R,10R,11S)-1,5-di(pyrrolidin-1-yl)-9,10-dihydro-9,10-[3,4]furanoanthracene-12,14-dione 2dB

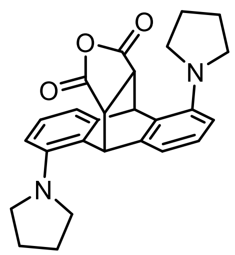	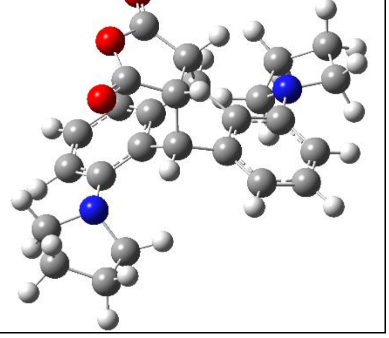

0 1

C	-2.79276000	-2.52899200	-1.01366600
C	-2.69270100	-1.23424100	-0.46917900
C	-1.44676800	-0.59093500	-0.53495600
C	-0.32603600	-1.25283500	-1.05644500
C	-0.43138000	-2.53899200	-1.57889500
C	-1.68071700	-3.16417400	-1.56717300
C	-1.15786500	0.84262800	-0.12032900
C	0.93316800	-0.40758000	-1.01865700
C	1.11070200	0.12403600	0.39564400
C	-0.00481300	0.82992700	0.86637400
C	0.03386400	1.50707200	2.08265400
H	-0.82358600	2.08189200	2.41916400
C	1.20932700	1.45730000	2.83529300
C	2.31264100	0.72743500	2.39189700
C	2.28127900	0.03608500	1.16625600
H	-3.74363800	-3.04917700	-0.99404500
H	0.43931900	-3.03715900	-1.99581900
H	-1.78925700	-4.16413600	-1.97734100
H	1.81992700	-0.91885200	-1.38913500
H	1.26713800	1.98596200	3.78228100
H	3.20650700	0.69195200	3.00436500
H	-2.03522900	1.37118400	0.24806200
C	-0.62484100	1.56099400	-1.41181900
C	0.63159000	0.83849100	-1.92421400
C	-0.15020500	2.96884800	-1.09393200
C	1.73487300	1.88011500	-1.82731100
O	-0.78579800	3.88910300	-0.66556600
O	2.89627100	1.76488300	-2.09192900
O	1.21084400	3.07819500	-1.34998700
N	3.37120700	-0.71361400	0.67733300
C	4.71496800	-0.46866800	1.19340500
C	3.23197800	-2.17829300	0.64239800
C	5.56547200	-1.46772900	0.39221600
H	4.99744100	0.57630200	1.03931400
C	4.59825800	-2.65384200	0.10764000
H	3.04994500	-2.57434100	1.65747000
H	6.46032300	-1.77248200	0.94147600
H	4.91267300	-3.57689700	0.60215800
N	-3.78423700	-0.56291600	0.12801400
C	-5.14650400	-1.02454700	-0.13272100
C	-3.72997800	-0.36823400	1.58950300
C	-5.99696300	-0.06245900	0.71349300
H	-5.36674100	-0.98756600	-1.20356500
C	-5.07394400	0.31451200	1.90867600

H	-3.65476600	-1.34294200	2.10298100
H	-6.24654600	0.82809800	0.13066300
H	-4.95149400	1.39892600	1.97357500
H	-5.30664100	-2.06340400	0.21126100
H	-6.93516200	-0.52524700	1.03096500
H	-5.47105600	-0.02842200	2.86777500
H	-2.85879000	0.22068900	1.88401500
H	2.38687000	-2.48462500	0.02092000
H	4.54569300	-2.85865900	-0.96505600
H	5.88854200	-1.00914300	-0.54575600
H	4.79495100	-0.69088800	2.27423000
H	-1.42888000	1.61639200	-2.14936000
H	0.54727600	0.51528200	-2.96509000

Zero-point correction= 0.476471 (Hartree/Particle)
Thermal correction to Energy= 0.501140
Thermal correction to Enthalpy= 0.502084
Thermal correction to Gibbs Free Energy= 0.420541
Sum of electronic and zero-point Energies= -1341.245963
Sum of electronic and thermal Energies= -1341.221294
Sum of electronic and thermal Enthalpies= -1341.220350
Sum of electronic and thermal Free Energies= -1341.301893

	E (Thermal)	CV	S
	KCal/Mol	Cal/Mol-Kelvin	Cal/Mol-Kelvin
Total	314.470	98.789	171.622

1.23. (9R,10S,11S)-1-(pyrrolidin-1-yl)-9,10-dihydro-9,10-[3,4]furanoanthracene-12,14-dione endo-2eB

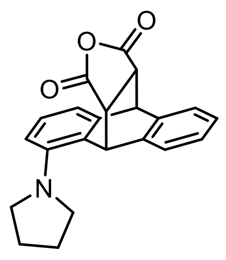

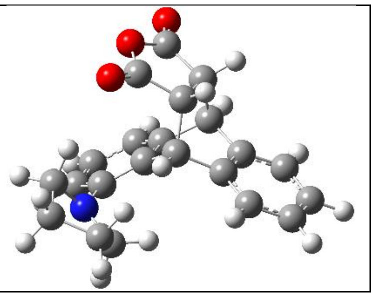

0 1

C	-1.62289700	-1.02674200	2.42250300
C	-1.51874600	-0.24707300	1.25706600
C	-0.25423700	-0.14017900	0.65537800
C	0.87497900	-0.72135400	1.25853900
C	0.75420900	-1.48938200	2.41118300

C	-0.51142700	-1.65235100	2.98326300
C	0.07012300	0.55198600	-0.65990400
C	2.16614500	-0.46910100	0.49717500
C	2.28638900	1.02440800	0.24561600
C	1.15489400	1.57621800	-0.38287000
C	1.11204800	2.93036100	-0.69328500
H	0.23668500	3.35049600	-1.18150000
C	2.20513300	3.74320500	-0.36764800
C	3.32853500	3.19646300	0.25318700
C	3.37445100	1.83025300	0.55896300
H	-2.59969900	-1.10995200	2.88846000
H	1.63015600	-1.95520800	2.85262900
H	-0.62486100	-2.24794200	3.88413500
H	3.03764400	-0.89465000	0.99828000
H	2.17688300	4.80347800	-0.60036300
H	4.17243000	3.83263500	0.50267400
H	-0.80745800	0.96901000	-1.15081400
C	0.73865600	-0.51759000	-1.59865400
C	1.97983100	-1.11945700	-0.91680200
C	-0.16696100	-1.71040200	-1.85145600
C	1.67202400	-2.60362900	-0.80919600
O	-1.24336000	-1.72854600	-2.37834700
O	2.33816200	-3.46947700	-0.32133000
O	0.42589300	-2.86825700	-1.37022200
N	-2.67580500	0.39835400	0.75904400
C	-3.54694700	-0.30218800	-0.19861200
C	-2.69371100	1.85305400	0.53776900
C	-4.47276700	0.81147100	-0.70205100
H	-2.97642700	-0.73984500	-1.03037200
C	-3.54111500	2.03514000	-0.73100400
H	-3.16028200	2.36027200	1.39613200
H	-4.91375000	0.57969700	-1.67519900
H	-2.90347600	1.98899000	-1.62305900
H	-4.09375100	-1.11602600	0.29255700
H	-5.28771400	0.97703300	0.01195700
H	-4.07363500	2.99023000	-0.75193600
H	-1.68659200	2.27567700	0.43698400
H	4.24915000	1.40336400	1.04228300
H	0.96321300	-0.04976900	-2.56059400
H	2.89491100	-0.99439000	-1.50132400

Zero-point correction= 0.366266 (Hartree/Particle)
Thermal correction to Energy= 0.385673
Thermal correction to Enthalpy= 0.386617

Thermal correction to Gibbs Free Energy= 0.317955
Sum of electronic and zero-point Energies= -1129.942584
Sum of electronic and thermal Energies= -1129.923177
Sum of electronic and thermal Enthalpies= -1129.922233
Sum of electronic and thermal Free Energies= -1129.990895

	E (Thermal)	CV	S
	KCal/Mol	Cal/Mol-Kelvin	Cal/Mol-Kelvin
Total	242.014	79.495	144.512

1.24. (9S,10R,11S)-4-(pyrrolidin-1-yl)-9,10-dihydro-9,10-[3,4]furanoanthracene-12,14-dione exo-2eB

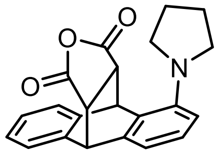

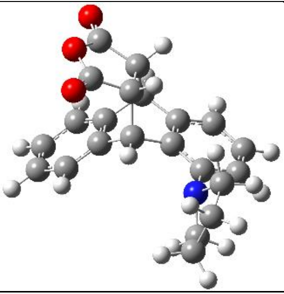

0 1

C	-2.21876900	1.35297500	2.95328100
C	-1.20008600	1.51190600	2.00719500
C	-1.02078500	0.53768100	1.03113300
C	-1.86266400	-0.58726900	0.98795100
C	-2.88464200	-0.73745500	1.91910300
C	-3.05534800	0.23643600	2.90936200
C	0.00368300	0.56765800	-0.08760100
C	-1.55616600	-1.52127100	-0.17020300
C	-0.08045500	-1.87739400	-0.10798400
C	0.75644600	-0.74834000	-0.06584000
C	2.14351200	-0.89107000	-0.01926300
C	2.68052300	-2.19097800	-0.00763100
C	1.85004300	-3.30890900	-0.04736700
C	0.45882000	-3.15760500	-0.10236600
H	-2.36270100	2.10687200	3.72139000
H	-0.55751200	2.38735300	2.02618200
H	-3.84776900	0.12463600	3.64322100
H	-2.21088900	-2.39465300	-0.19152900
H	3.75809900	-2.31965800	0.03317200
H	2.28450300	-4.30424900	-0.03553500
H	-0.19131200	-4.02750800	-0.13441600
H	0.67253800	1.42606700	-0.04211800

C	-0.80978700	0.55733600	-1.43517000
C	-1.73058400	-0.67707800	-1.47995000
C	-1.75869900	1.74046400	-1.52495200
C	-3.13266200	-0.09928500	-1.58603900
O	-1.49609200	2.90810500	-1.51505200
O	-4.17563700	-0.68558300	-1.62957800
O	-3.07193500	1.28843600	-1.61076900
H	-0.11274200	0.60544000	-2.27546400
H	-1.55076600	-1.31510900	-2.34899500
C	3.85167800	0.43545500	1.17130800
C	3.81653900	0.50818800	-1.16178500
C	4.65580200	1.69631800	0.81552600
H	4.52891900	-0.42982700	1.29557700
H	3.26481500	0.53146000	2.08964600
C	4.69906900	1.70184900	-0.74110600
H	3.20123300	0.70784600	-2.04485600
H	4.44372800	-0.37344600	-1.38818600
H	4.13767800	2.58631000	1.18259200
H	5.65144000	1.68294600	1.26702200
H	4.29671000	2.63935800	-1.13331900
H	5.71543500	1.59481000	-1.12967400
N	2.96275700	0.28359800	0.01305900
H	-3.54420000	-1.59917100	1.87145200

Zero-point correction= 0.365858 (Hartree/Particle)
Thermal correction to Energy= 0.384680
Thermal correction to Enthalpy= 0.385624
Thermal correction to Gibbs Free Energy= 0.317867
Sum of electronic and zero-point Energies= -1129.942996
Sum of electronic and thermal Energies= -1129.924175
Sum of electronic and thermal Enthalpies= -1129.923230
Sum of electronic and thermal Free Energies= -1129.990988

	E (Thermal)	CV	S
	KCal/Mol	Cal/Mol-Kelvin	Cal/Mol-Kelvin
Total	241.390	77.459	142.608

1.25. (3aR,4R,11S,11aS)-3a,4,11,11a-tetrahydro-4,11-ethenoanthra[2,3-c]furan-1,3-dione endo-3aB

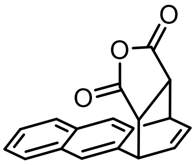

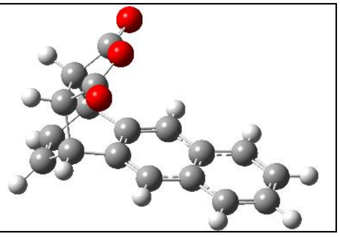

0 1

C	-4.56489800	-0.70774600	0.35457100
C	-3.40984500	-1.40238500	0.07052500
C	-2.20317200	-0.71727400	-0.22670500
C	-2.20317800	0.71731400	-0.22636200
C	-3.40984200	1.40228100	0.07123800
C	-4.56489500	0.70750000	0.35493900
C	-0.99474000	-1.41226800	-0.51931700
C	-0.99478100	1.41247700	-0.51869100
C	0.14548100	0.71301400	-0.81723500
C	0.14550600	-0.71263100	-0.81751900
C	1.51766900	-1.29697200	-1.10831800
H	1.51219000	-2.38770900	-1.11819700
C	2.03444200	-0.66802100	-2.39389400
C	2.03443900	0.66914600	-2.39361100
C	1.51762600	1.29754000	-1.10780300
H	-5.48076100	-1.24509200	0.58184400
H	-3.40630100	-2.48915700	0.07244900
H	-3.40629100	2.48905100	0.07374600
H	-5.48074900	1.24473700	0.58250500
H	-0.98744400	2.49918400	-0.49660600
H	2.37174300	-1.28137000	-3.22243100
H	2.37169200	1.28287200	-3.22189100
H	1.51212500	2.38827400	-1.11726400
H	-0.98738000	-2.49898600	-0.49776400
C	2.46652500	-0.76897900	0.03095500
C	2.46646000	0.76906300	0.03130500
C	1.95156100	-1.14863700	1.41011300
C	1.95137000	1.14804500	1.41060300
O	1.76968500	-2.24277400	1.85992400
O	1.76933500	2.24192800	1.86096400
O	1.67876600	-0.00050000	2.14545900
H	3.46228300	-1.19996800	-0.09831900
H	3.46223600	1.20013700	-0.09761100

Zero-point correction= 0.255837 (Hartree/Particle)
Thermal correction to Energy= 0.270138
Thermal correction to Enthalpy= 0.271082
Thermal correction to Gibbs Free Energy= 0.214418
Sum of electronic and zero-point Energies= -918.625061
Sum of electronic and thermal Energies= -918.610761
Sum of electronic and thermal Enthalpies= -918.609817
Sum of electronic and thermal Free Energies= -918.666480

	E (Thermal)	CV	S
	KCal/Mol	Cal/Mol-Kelvin	Cal/Mol-Kelvin
Total	169.514	60.536	119.259

1.26. (3aS,4S,11S,11aS)-4,9-dimethoxy-3a,4,11,11a-tetrahydro-4,11-ethenoanthra[2,3-c]furan-1,3-dione endo-3bB

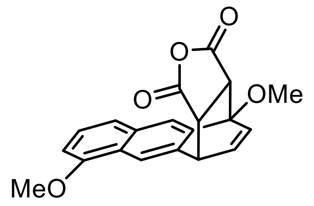

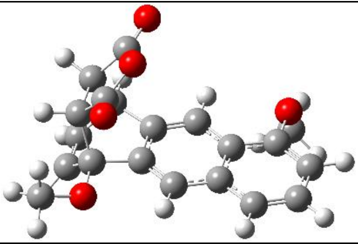

0 1

C	-4.35371400	1.03950400	0.60038200
C	-3.56869600	-0.01972100	0.19946500
C	-2.18044600	0.15426000	-0.06720200
C	-1.61650300	1.46469300	0.07798500
C	-2.45100400	2.53688200	0.48839600
C	-3.78714600	2.32605500	0.74852800
C	-1.35015900	-0.93362100	-0.45560200
C	-0.23009000	1.65205000	-0.18915800
C	0.53343100	0.59005600	-0.59571900
C	-0.02469800	-0.71251300	-0.72705500
C	1.00817200	-1.76038000	-1.10267400
H	0.57485000	-2.75613300	-1.20329100
C	1.73833300	-1.27271900	-2.34476700
C	2.25870500	-0.04757200	-2.23131500
C	2.02861800	0.63199400	-0.88280900
H	-5.40463100	0.86362200	0.80590100
H	-2.01539700	3.52557700	0.60011800
H	-4.41655900	3.15022100	1.07037500
H	0.22185200	2.63035400	-0.06898600
H	1.80153900	-1.88484500	-3.23803300

H	2.79223900	0.48393900	-3.01172100
H	-1.77780500	-1.92873700	-0.50887800
C	2.07099100	-1.74455500	0.05429400
C	2.69655400	-0.34631600	0.17940700
O	2.47219600	1.96061400	-0.82048900
O	-4.11681900	-1.28137900	0.07453300
C	-4.67553000	-1.54395200	-1.21372700
H	-5.07067200	-2.56142400	-1.18191600
H	-5.48865500	-0.84369900	-1.44554900
H	-3.91316900	-1.47305700	-2.00124100
C	3.87563600	2.17375000	-0.74123100
H	4.03371200	3.23551100	-0.93990200
H	4.25369400	1.93919500	0.26054900
H	4.42749800	1.59091700	-1.49252600
C	1.42286400	-1.98750600	1.40960900
C	2.41486900	0.07321900	1.60893500
O	2.76693600	1.06708300	2.17703600
O	0.79465800	-2.93975700	1.77001600
O	1.64932500	-0.89841200	2.24353800
H	2.81341800	-2.52743900	-0.12029800
H	3.77661000	-0.35085000	0.02386800

Zero-point correction= 0.320097 (Hartree/Particle)
Thermal correction to Energy= 0.339808
Thermal correction to Enthalpy= 0.340753
Thermal correction to Gibbs Free Energy= 0.272304
Sum of electronic and zero-point Energies= -1147.610939
Sum of electronic and thermal Energies= -1147.591227
Sum of electronic and thermal Enthalpies= -1147.590283
Sum of electronic and thermal Free Energies= -1147.658731

	E (Thermal)	CV	S
	KCal/Mol	Cal/Mol-Kelvin	Cal/Mol-Kelvin
Total	213.233	79.290	144.061

1.27. (3aS,4S,11S,11aS)-4,9-bis(dimethylamino)-3a,4,11,11a-tetrahydro-4,11-ethenoanthra[2,3-c]furan-1,3-dione endo-3cB

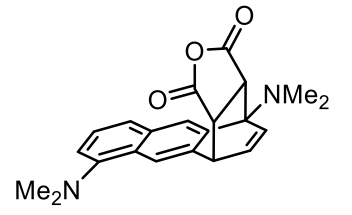

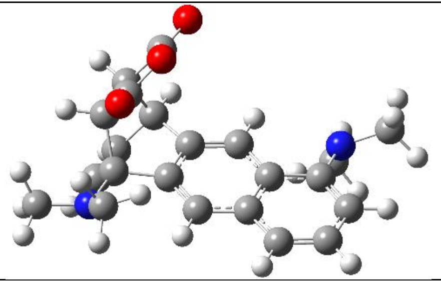

0 1

C	4.16954700	1.49953000	-0.55405100
C	3.50901200	0.37213200	-0.09458600
C	2.09002900	0.43117200	0.11462400
C	1.40508700	1.67158900	-0.08306700
C	2.13105400	2.80885900	-0.52401600
C	3.47941900	2.71266200	-0.77575000
C	1.33142500	-0.70420600	0.51261500
C	-0.00253800	1.73821700	0.12930700
C	-0.71738600	0.62129700	0.48423800
C	-0.02089900	-0.60447500	0.71808600
C	-0.89315100	-1.71995100	1.26740300
H	-0.31753700	-2.61748900	1.49776400
C	-1.64311500	-1.14227000	2.45020900
C	-2.31545400	-0.02218200	2.18082600
C	-2.23067800	0.50324800	0.73976100
H	5.23919400	1.46564800	-0.72897800
H	1.60164100	3.74457600	-0.67992500
H	4.03152700	3.57800900	-1.13084800
H	-0.48948900	2.69798600	0.01140500
H	-1.59522300	-1.59994200	3.43220800
H	-2.83913400	0.55323500	2.93265600
H	1.83432100	-1.65529500	0.64531300
C	-1.97772100	-2.03572100	0.19318400
C	-2.73286600	-0.74738500	-0.17592800
N	-2.99534100	1.73316200	0.59571400
N	4.17730500	-0.85831900	0.16821200
C	4.39107500	-1.12816100	1.59288900
H	3.47582300	-0.94013100	2.15675500
H	4.67042900	-2.17850300	1.72832800
H	5.19098500	-0.49864800	2.02076100
C	5.37863000	-1.11975400	-0.61218400
H	6.25356400	-0.52315500	-0.29656400
H	5.64588500	-2.17612500	-0.50486800

H	5.18457800	-0.91877700	-1.66833400
C	-2.97095300	2.45153600	-0.68839600
H	-2.05493000	2.26550500	-1.24092000
H	-3.79579200	2.15330900	-1.34635400
H	-3.05149200	3.52722600	-0.48956200
C	-4.38014600	1.67661100	1.05626400
H	-4.46940400	1.26205600	2.06037800
H	-4.77611300	2.69652000	1.08655800
H	-5.03694200	1.09280600	0.38570000
C	-1.31753200	-2.49554300	-1.09285100
C	-2.49697300	-0.61878500	-1.67508100
O	-0.60762700	-3.44477100	-1.26496800
O	-2.96654900	0.15289800	-2.46211400
O	-1.63164000	-1.61787300	-2.11422600
H	-2.63331200	-2.83232900	0.55437900
H	-3.81100100	-0.83293500	-0.02384200

Zero-point correction= 0.402152 (Hartree/Particle)
Thermal correction to Energy= 0.424548
Thermal correction to Enthalpy= 0.425493
Thermal correction to Gibbs Free Energy= 0.351711
Sum of electronic and zero-point Energies= -1186.423436
Sum of electronic and thermal Energies= -1186.401040
Sum of electronic and thermal Enthalpies= -1186.400096
Sum of electronic and thermal Free Energies= -1186.473878

	E (Thermal)	CV	S
	KCal/Mol	Cal/Mol-Kelvin	Cal/Mol-Kelvin
Total	266.408	91.121	155.287

1.28. (3aS,4S,11S,11aS)-4,9-di(pyrrolidin-1-yl)-3a,4,11,11a-tetrahydro-4,11-ethenoanthra[2,3-c]furan-1,3-dione endo-3dB

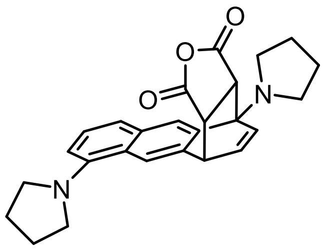

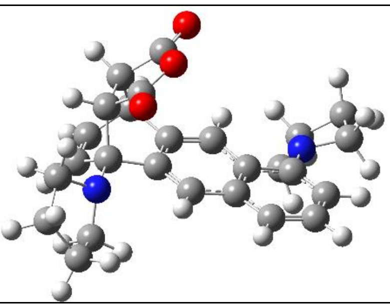

0 1

C	-3.48001400	-2.30253900	-0.99710600
C	-3.18197200	-1.12791500	-0.32392700

C	-1.80504200	-0.78499500	-0.09579100
C	-0.78116500	-1.71316000	-0.47448700
C	-1.13897400	-2.91912500	-1.13268700
C	-2.45866200	-3.18856400	-1.40812900
C	-1.41919800	0.47788000	0.43289000
C	0.58132500	-1.38953400	-0.21915500
C	0.91272600	-0.20471800	0.38823600
C	-0.09718900	0.75275700	0.67829100
C	0.46749200	2.09173200	1.11172100
H	-0.31119900	2.83153300	1.30211900
C	1.40725600	1.86061700	2.27932500
C	2.35902900	0.95490900	2.04327400
C	2.33709000	0.30082800	0.65436400
H	-4.51231000	-2.56386100	-1.19940700
H	-0.35499300	-3.60956200	-1.43007200
H	-2.73145300	-4.10169900	-1.92931700
H	1.36290700	-2.07037800	-0.53977400
H	1.29957400	2.41006600	3.20894200
H	3.12831700	0.68188500	2.75672000
H	-2.17655500	1.23927600	0.58290500
C	1.38331900	2.56204500	-0.07610400
C	2.49529600	1.52988000	-0.34144200
C	0.60626700	2.62805900	-1.38115200
C	2.30649800	1.14178200	-1.80029100
O	-0.36308100	3.28356800	-1.63418300
O	2.98114800	0.42552500	-2.48196000
O	1.18070400	1.77786000	-2.31589400
N	3.33326200	-0.73064200	0.42968600
C	4.72394000	-0.31312400	0.20543400
C	3.38964300	-1.81488300	1.41696800
C	5.54793600	-1.61771700	0.35771000
H	4.81962400	0.12725600	-0.78952800
C	4.53837600	-2.66981000	0.87997000
H	3.63123000	-1.45477700	2.43474500
H	6.36808600	-1.46994100	1.06627700
H	4.96311800	-3.33023600	1.64098300
N	-4.17819800	-0.23125300	0.12249000
C	-5.52152000	-0.30625000	-0.44704900
C	-4.39503700	-0.11380400	1.57628000
C	-6.26768700	0.81320300	0.29735600
H	-5.48510700	-0.17180900	-1.53176300
C	-5.56280600	0.88649200	1.68309300
H	-4.68034200	-1.09257600	2.00133500
H	-6.15183100	1.75904700	-0.23829200

H	-5.19164600	1.89725300	1.87350200
H	-6.00716900	-1.27866500	-0.24099200
H	-7.33795000	0.60418800	0.37612000
H	-6.23168900	0.62307300	2.50690700
H	-3.48853000	0.21400000	2.08814600
H	2.43479600	-2.34210300	1.47648300
H	4.17478700	-3.29084400	0.05594000
H	5.99152300	-1.92453800	-0.59252300
H	5.05800600	0.43436600	0.94908400
H	1.77827400	3.55780400	0.14159500
H	3.49335600	1.95462500	-0.22463900

Zero-point correction= 0.475683 (Hartree/Particle)
Thermal correction to Energy= 0.500069
Thermal correction to Enthalpy= 0.501013
Thermal correction to Gibbs Free Energy= 0.421423
Sum of electronic and zero-point Energies= -1341.218584
Sum of electronic and thermal Energies= -1341.194197
Sum of electronic and thermal Enthalpies= -1341.193253
Sum of electronic and thermal Free Energies= -1341.272843

	E (Thermal)	CV	S
	KCal/Mol	Cal/Mol-Kelvin	Cal/Mol-Kelvin
Total	313.798	99.486	167.513

1.29. (3aS,4S,11S,11aS)-4-(pyrrolidin-1-yl)-3a,4,11,11a-tetrahydro-4,11-ethenoanthra[2,3-c]furan-1,3-dione endo-3eB

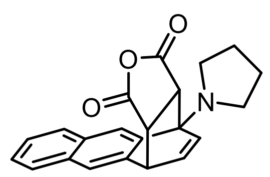

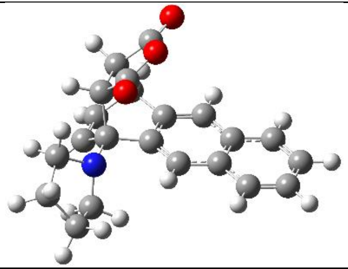

0 1

C	-1.73818900	-0.59140900	1.98657200
C	-1.39052100	-0.11140600	0.57015700
C	0.11575000	0.18317600	0.52413400
C	0.91761800	-0.85562900	1.07671300
C	0.10312000	-2.05230900	1.52784500
C	-0.97666700	-1.57161200	2.47720500
C	0.70305400	1.26187200	-0.08722800
C	2.28659700	-0.77420300	1.05356900

C	2.92682700	0.36248800	0.48495000
C	2.12033400	1.39072100	-0.10553400
C	2.76241900	2.50870900	-0.69941500
H	2.14851400	3.28370100	-1.15065400
C	4.13568900	2.61270100	-0.70637200
C	4.93131700	1.59909900	-0.12006000
C	4.33927600	0.49942700	0.46056000
H	-2.57090700	-0.14563300	2.51841200
H	0.72481300	-2.85536700	1.92589900
H	-1.10544300	-2.01703000	3.45827000
H	2.89305300	-1.58533800	1.44799900
H	4.61398300	3.47322900	-1.16463100
H	6.01332200	1.69072000	-0.13325300
H	0.08743300	2.00716800	-0.57968300
C	-1.54842400	-1.41855300	-0.31981300
C	-0.65776100	-2.54261900	0.24252300
N	-2.17745300	1.00381100	0.07414800
C	-3.54768200	0.73943100	-0.38439600
C	-2.26846100	2.18093400	0.94649700
C	-4.18324500	2.14686900	-0.52266900
H	-3.52207400	0.19949100	-1.33317400
C	-3.16040400	3.11376600	0.12619600
H	-2.75057000	1.95694300	1.91638400
H	-4.36460500	2.40111300	-1.56968500
H	-2.56075100	3.60713500	-0.64435300
H	-4.11950800	0.13556500	0.34508900
H	-5.14755500	2.18263000	-0.00779500
H	-3.63094700	3.88880400	0.73735200
H	-1.27815600	2.58876700	1.16169900
H	4.94792400	-0.28296200	0.90612300
C	0.31051700	-2.86192400	-0.88523200
C	-1.05817200	-1.23177900	-1.74838500
O	0.03242900	-2.06382800	-1.98589400
O	1.20439100	-3.65837000	-0.89106400
O	-1.49298500	-0.51951000	-2.60637700
H	-1.22321600	-3.44767500	0.47897000
H	-2.60176500	-1.70076800	-0.35223400

Zero-point correction= 0.365709 (Hartree/Particle)
Thermal correction to Energy= 0.384972
Thermal correction to Enthalpy= 0.385916
Thermal correction to Gibbs Free Energy= 0.318512
Sum of electronic and zero-point Energies= -1129.919442
Sum of electronic and thermal Energies= -1129.900180

Sum of electronic and thermal Enthalpies= -1129.899235
Sum of electronic and thermal Free Energies= -1129.966639

	E (Thermal)	CV	S
	KCal/Mol	Cal/Mol-Kelvin	Cal/Mol-Kelvin
Total	241.573	80.149	141.864

1.30. (3aR,4S,11R,11aS)-3a,4,11,11a-tetrahydro-4,11-ethenoanthra[2,3-c]furan-1,3-dione exo-3aB

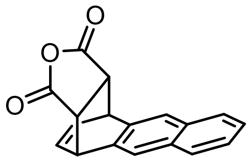

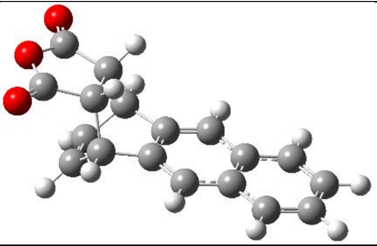

0 1

C	-0.38740400	-5.22334500	0.70747200
C	-0.21308700	-4.04621300	1.40192800
C	-0.03188700	-2.81672800	0.71719200
C	-0.03188700	-2.81672800	-0.71719200
C	-0.21308700	-4.04621300	-1.40192800
C	-0.38740400	-5.22334500	-0.70747200
C	0.14945800	-1.58531900	1.41170300
C	0.14945800	-1.58531900	-1.41170300
C	0.33395100	-0.42109700	-0.71352200
C	0.33395100	-0.42109700	0.71352200
C	0.52548000	0.96794400	1.29779100
H	0.54436000	0.97645300	2.38831400
C	1.77129400	1.57432400	0.66875900
C	1.77129400	1.57432400	-0.66875900
C	0.52548000	0.96794400	-1.29779100
H	-0.52561600	-6.15667900	1.24500700
H	-0.21198800	-4.04391500	2.48882200
H	-0.21198800	-4.04391500	-2.48882200
H	-0.52561600	-6.15667900	-1.24500700
H	0.14465200	-1.58459600	-2.49914500
H	2.56607100	1.98319100	1.28255500
H	2.56607100	1.98319100	-1.28255500
H	0.54436000	0.97645300	-2.38831400
H	0.14465200	-1.58459600	2.49914500
C	-0.69690400	1.80537500	0.76915200
C	-0.69690400	1.80537500	-0.76915200

C	-0.57436800	3.27201600	1.14784000
C	-0.57436800	3.27201600	-1.14784000
O	-0.52640200	4.05470800	0.00000000
O	-0.50641200	3.75445500	-2.24132900
O	-0.50641200	3.75445500	2.24132900
H	-1.61977100	1.40933400	-1.20047200
H	-1.61977100	1.40933400	1.20047200

Zero-point correction= 0.255805 (Hartree/Particle)
Thermal correction to Energy= 0.270155
Thermal correction to Enthalpy= 0.271099
Thermal correction to Gibbs Free Energy= 0.214254
Sum of electronic and zero-point Energies= -918.625233
Sum of electronic and thermal Energies= -918.610883
Sum of electronic and thermal Enthalpies= -918.609939
Sum of electronic and thermal Free Energies= -918.666785

	E (Thermal)	CV	S
	KCal/Mol	Cal/Mol-Kelvin	Cal/Mol-Kelvin
Total	169.525	60.517	119.641

1.31. (3aR,4S,11S,11aR)-4,9-dimethoxy-3a,4,11,11a-tetrahydro-4,11-ethenoanthra[2,3-c]furan-1,3-dione exo-3bB

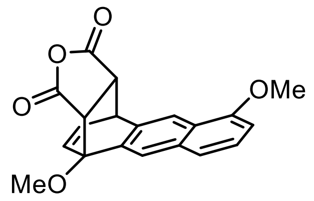

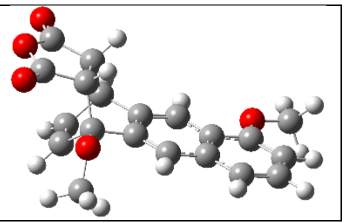

0 1

C	4.90485900	0.41760300	-0.39732200
C	3.92614000	-0.50135300	-0.06668800
C	2.55188600	-0.10750000	0.00593800
C	2.21479700	1.25709300	-0.26556400
C	3.24201800	2.17827800	-0.60360700
C	4.55001300	1.76104000	-0.66609600
C	1.52400100	-1.03672800	0.32725000
C	0.84912200	1.65462100	-0.19187000
C	-0.11410600	0.74571300	0.15896800
C	0.22184700	-0.61270200	0.40630100
C	-0.99845300	-1.47289900	0.66884800
H	-0.75877300	-2.52008700	0.85641000
C	-1.80190700	-0.82784100	1.78513600

C	-2.11890200	0.45378900	1.57607800
C	-1.61624000	1.02764800	0.25380400
H	5.94593500	0.12566200	-0.45502300
H	2.97730400	3.21094900	-0.81044300
H	5.33472400	2.46567400	-0.92504600
H	0.57667500	2.67910900	-0.42485600
H	-2.11144500	-1.39595800	2.65527200
H	-2.72204900	1.05113400	2.24856900
H	1.78502300	-2.07427900	0.50308600
C	-1.86970500	-1.34280800	-0.63714400
C	-2.25511500	0.12816300	-0.87260300
C	-3.19664600	-2.06447300	-0.47625100
C	-3.77801200	0.13291300	-0.82631500
O	-3.38199600	-3.22733900	-0.25345000
O	-4.54002000	1.04359700	-0.95468000
O	-4.24153100	-1.16594500	-0.59671900
O	-1.95559300	2.36184100	-0.02923100
O	4.15982800	-1.81673500	0.21014800
C	5.49796900	-2.28909200	0.16586400
H	5.45163000	-3.34887900	0.42005800
H	5.93308500	-2.17507200	-0.83544300
H	6.13282400	-1.76868200	0.89456600
C	-1.79226300	3.29365200	1.03493800
H	-1.78887800	4.28447000	0.57577000
H	-2.62332200	3.24572500	1.74969400
H	-0.84783700	3.14425700	1.57328400
H	-1.32635700	-1.78495400	-1.47563300
H	-1.93187200	0.52689700	-1.83680000

Zero-point correction= 0.320547 (Hartree/Particle)
Thermal correction to Energy= 0.340181
Thermal correction to Enthalpy= 0.341125
Thermal correction to Gibbs Free Energy= 0.272946
Sum of electronic and zero-point Energies= -1147.613108
Sum of electronic and thermal Energies= -1147.593474
Sum of electronic and thermal Enthalpies= -1147.592530
Sum of electronic and thermal Free Energies= -1147.660708

	E (Thermal)	CV	S
	KCal/Mol	Cal/Mol-Kelvin	Cal/Mol-Kelvin
Total	213.467	79.184	143.494

1.32. (3aR,4S,11S,11aR)-4,9-bis(dimethylamino)-3a,4,11,11a-tetrahydro-4,11-ethenoanthra[2,3-c]furan-1,3-dione exo-3cB

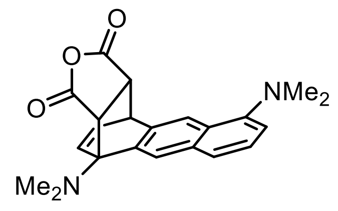

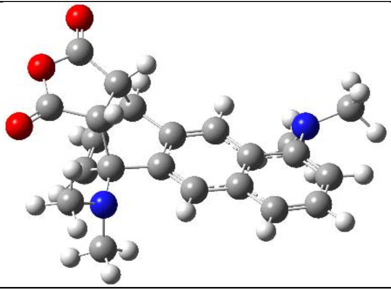

0 1

C	4.72603500	0.92024600	-0.65968600
C	3.90099900	-0.09233200	-0.19785300
C	2.48792000	0.14729100	-0.10185200
C	1.97808700	1.45260800	-0.40149700
C	2.86806300	2.46470300	-0.84831600
C	4.20750600	2.19176500	-0.99497600
C	1.56470400	-0.88337800	0.23193700
C	0.58220800	1.70402700	-0.27218800
C	-0.27194000	0.70916000	0.12985300
C	0.22819100	-0.60008700	0.35172900
C	-0.88294900	-1.59744100	0.58305000
H	-0.52909700	-2.62246300	0.70331700
C	-1.73172000	-1.10482700	1.74167200
C	-2.18193000	0.14802200	1.61654100
C	-1.80165100	0.86211100	0.31078100
H	5.79346600	0.75148400	-0.74729900
H	2.47098200	3.44815200	-1.08345500
H	4.88639500	2.96224300	-1.34891700
H	0.18900600	2.68882700	-0.50127900
H	-1.94905400	-1.74703500	2.58852000
H	-2.81901700	0.63854100	2.34319300
H	1.92920600	-1.89825100	0.34907200
C	-1.74675500	-1.47896200	-0.72860900
C	-2.42651300	-0.09059600	-0.79776300
C	-2.87788200	-2.48129400	-0.71244600
C	-3.91229800	-0.43693300	-0.59332800
O	-2.81312400	-3.67725000	-0.73948200
O	-4.86311100	0.27514300	-0.44891000
O	-4.08601800	-1.81622100	-0.61462300
N	-2.14311100	2.28237500	0.21084900
N	4.39003900	-1.37817900	0.16819400
C	4.40565000	-1.62761600	1.61349200
H	4.54359100	-2.69827700	1.79789200

H	5.22007400	-1.08085000	2.11969400
H	3.46063500	-1.31996900	2.06332900
C	5.63478800	-1.80055300	-0.45801600
H	6.52887500	-1.28803700	-0.05969800
H	5.76864900	-2.87377600	-0.28757300
H	5.58633100	-1.62525900	-1.53538800
C	-1.83733800	3.03104800	1.42622000
H	-1.73740000	4.09350900	1.17879000
H	-2.61720400	2.94518400	2.20337600
H	-0.89000200	2.69445000	1.85318000
C	-3.39495200	2.73571600	-0.37962700
H	-4.27783800	2.57612000	0.25591200
H	-3.29828600	3.81035400	-0.57296700
H	-3.58501600	2.24592500	-1.33374000
H	-2.30988600	0.36907600	-1.78390300
H	-1.10421100	-1.68877500	-1.58593300

Zero-point correction= 0.402064 (Hartree/Particle)
Thermal correction to Energy= 0.424402
Thermal correction to Enthalpy= 0.425346
Thermal correction to Gibbs Free Energy= 0.351724
Sum of electronic and zero-point Energies= -1186.426059
Sum of electronic and thermal Energies= -1186.403721
Sum of electronic and thermal Enthalpies= -1186.402776
Sum of electronic and thermal Free Energies= -1186.476398

	E (Thermal)	CV	S
	KCal/Mol	Cal/Mol-Kelvin	Cal/Mol-Kelvin
Total	266.316	90.955	154.950

1.33. (3aR,4S,11S,11aR)-4,9-di(pyrrolidin-1-yl)-3a,4,11,11a-tetrahydro-4,11-ethenoanthra[2,3-c]furan-1,3-dione exo-3dB

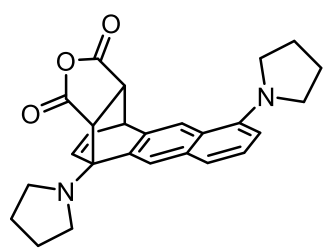

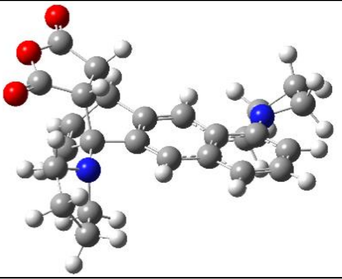

0 1

C	4.12013600	2.02528700	-0.94269600
C	3.62224600	0.84172700	-0.41958800

C	2.20066400	0.68824900	-0.27336400
C	1.34305400	1.79815200	-0.56561300
C	1.90465900	3.00287300	-1.06518100
C	3.26049700	3.09741600	-1.27276400
C	1.60565500	-0.55161400	0.09097600
C	-0.06229300	1.66134600	-0.38091900
C	-0.59677700	0.47962300	0.06734300
C	0.24760300	-0.64339200	0.26091800
C	-0.54111500	-1.90832600	0.49964200
H	0.08198600	-2.79895400	0.59303200
C	-1.46941200	-1.68910800	1.67947300
C	-2.25261200	-0.61074900	1.58036000
C	-2.10218600	0.20531100	0.29163500
H	5.18764000	2.14848200	-1.08397000
H	1.24590600	3.83555900	-1.29474000
H	3.68872900	4.01188700	-1.67306000
H	-0.71737700	2.49464900	-0.61310000
H	-1.48858300	-2.38218200	2.51382500
H	-2.99282800	-0.32860200	2.32045500
H	2.23394000	-1.43139900	0.18015700
C	-1.44060100	-2.02117200	-0.78810600
C	-2.45294900	-0.84928400	-0.85678800
N	-2.81720400	1.47359000	0.23381300
C	-4.24953800	1.56479200	-0.06744700
C	-2.63677400	2.34424500	1.40557300
C	-4.53975300	3.07345700	0.08063200
H	-4.46759300	1.18560000	-1.06471600
C	-3.41742800	3.60594500	1.01414000
H	-3.07528700	1.90229800	2.31879000
H	-5.53956300	3.24007500	0.49038200
H	-3.80482900	4.13133800	1.89135700
N	4.45416600	-0.23945200	-0.05062800
C	5.82615400	-0.29163800	-0.55212100
C	4.57366100	-0.54914400	1.38770300
C	6.37255400	-1.58065000	0.08320200
H	5.83469200	-0.29606600	-1.64586700
C	5.58743500	-1.70954000	1.42047800
H	4.96556000	0.32631500	1.93484600
H	6.16305500	-2.43575100	-0.56496100
H	5.07284200	-2.67285700	1.47419400
H	6.42499600	0.57370000	-0.21159100
H	7.45494700	-1.53237100	0.22915900
H	6.23830400	-1.63811300	2.29602100
H	3.60489000	-0.80097000	1.82230600

H	-1.57872600	2.52527100	1.60617100
H	-2.76479500	4.29926700	0.47548800
H	-4.49928600	3.57030100	-0.89251100
H	-4.86435300	0.98276900	0.63821500
C	-3.80754600	-1.56920100	-0.74167000
C	-2.27706600	-3.27859600	-0.72267800
O	-4.92227700	-1.13320700	-0.74951300
O	-1.90560000	-4.41718200	-0.68371400
O	-3.61620900	-2.94461200	-0.66903400
H	-0.78999600	-2.07516000	-1.66368300
H	-2.42992600	-0.33009000	-1.81925400

Zero-point correction= 0.475630 (Hartree/Particle)
Thermal correction to Energy= 0.500076
Thermal correction to Enthalpy= 0.501020
Thermal correction to Gibbs Free Energy= 0.420674
Sum of electronic and zero-point Energies= -1341.212392
Sum of electronic and thermal Energies= -1341.187947
Sum of electronic and thermal Enthalpies= -1341.187003
Sum of electronic and thermal Free Energies= -1341.267349

	E (Thermal)	CV	S
	KCal/Mol	Cal/Mol-Kelvin	Cal/Mol-Kelvin
Total	313.802	99.535	169.102

1.34. (3aR,4S,11S,11aR)-4-(pyrrolidin-1-yl)-3a,4,11,11a-tetrahydro-4,11-ethenoanthra[2,3-c]furan-1,3-dione exo-3eB

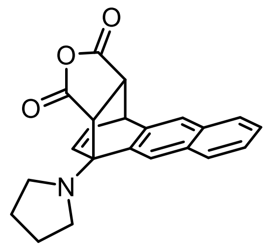

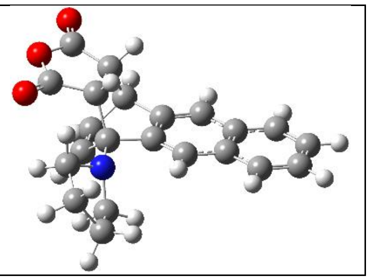

0 1

C	-1.44835200	-0.04776400	1.59810500
C	-0.88504700	0.46509100	0.26783500
C	0.59944900	0.03668200	0.17813700
C	0.85093900	-1.31347500	0.54248600
C	-0.42533400	-2.07445900	0.80815000
C	-1.22436700	-1.33743100	1.86644700
C	1.62750100	0.82851600	-0.26820500
C	2.11859200	-1.83477600	0.51057200

C	3.21567600	-1.02070700	0.11109500
C	2.96027000	0.32889500	-0.30006000
C	4.05000300	1.13297500	-0.72599600
H	3.85286300	2.15539000	-1.03773000
C	5.33335400	0.63295900	-0.74224200
C	5.58505300	-0.69856100	-0.33274300
C	4.54920700	-1.50579000	0.08327000
H	-2.02826900	0.61114400	2.23405000
H	-0.26421400	-3.12879700	1.03729700
H	-1.59009100	-1.84969600	2.74990200
H	2.29491400	-2.87438100	0.77618600
H	6.15785300	1.25941900	-1.06934500
H	6.60077300	-1.08248500	-0.34907200
H	1.42303900	1.83945400	-0.60474100
C	-1.59750800	-0.46289700	-0.81982700
C	-1.21365600	-1.93969900	-0.54789700
C	-3.13511600	-0.50477600	-0.79139600
C	-2.52305400	-2.69031700	-0.45488500
O	-3.93855200	0.36571200	-0.96246200
O	-2.69567100	-3.86173900	-0.27210400
O	-3.57655900	-1.80544600	-0.57501000
N	-0.96729200	1.90200600	0.03748200
C	-2.18523100	2.56562600	-0.43771800
C	-0.51296200	2.74041000	1.15803200
C	-1.78257100	4.05478900	-0.44730200
H	-2.47858500	2.19817100	-1.41999100
C	-0.63039500	4.16188000	0.59022200
H	-1.16850900	2.63689200	2.04157700
H	-1.43313500	4.34953000	-1.44048900
H	0.30406100	4.45176300	0.10082800
H	-3.04212200	2.40162400	0.23568800
H	-2.63437200	4.69276400	-0.19726100
H	-0.83118300	4.89469100	1.37650400
H	0.49856900	2.47214900	1.47030200
H	4.74031200	-2.52912400	0.39582400
H	-1.29069700	-0.12565600	-1.81408600
H	-0.61262000	-2.37812400	-1.34768700

Zero-point correction= 0.365421 (Hartree/Particle)
Thermal correction to Energy= 0.384820
Thermal correction to Enthalpy= 0.385764
Thermal correction to Gibbs Free Energy= 0.316692
Sum of electronic and zero-point Energies= -1129.913271
Sum of electronic and thermal Energies= -1129.893872

Sum of electronic and thermal Enthalpies= -1129.892928
Sum of electronic and thermal Free Energies= -1129.962000

	E (Thermal)	CV	S
	KCal/Mol	Cal/Mol-Kelvin	Cal/Mol-Kelvin
Total	241.478	80.248	145.376

1.35. (3aR,4S,11R,11aS)-6-(pyrrolidin-1-yl)-3a,4,11,11a-tetrahydro-4,11-ethenoanthra[2,3-c]furan-1,3-dione

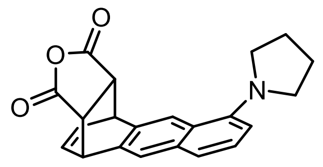

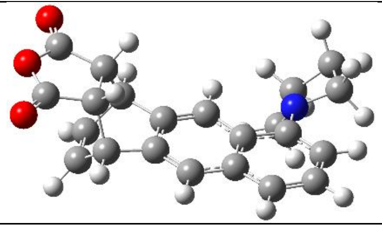

0 1

C	3.92728500	1.73937100	-0.47318900
C	3.13280700	0.62763000	-0.23850900
C	1.72220100	0.80857000	-0.03125400
C	1.19151500	2.13822400	0.03825200
C	2.05310000	3.24673600	-0.16981500
C	3.38452200	3.04341600	-0.44742900
C	0.82647700	-0.29477600	0.06541500
C	-0.20180800	2.31830400	0.28014100
C	-1.02705200	1.23433100	0.42500700
C	-0.50828600	-0.08585000	0.29816700
C	-1.58692300	-1.15066700	0.39883900
H	-1.19774600	-2.16609600	0.31399500
C	-2.36486100	-0.90834300	1.68427800
C	-2.84923800	0.33197200	1.81223500
C	-2.52974600	1.25525600	0.64588000
H	4.98849800	1.61761200	-0.65688900
H	1.64101400	4.25089900	-0.12575300
H	4.03957800	3.89094900	-0.62736800
H	-0.59923900	3.32882100	0.33929300
H	-2.51384400	-1.70264000	2.40725600
H	-3.44312200	0.67529700	2.65194500
H	1.21156700	-1.29768200	-0.08358200
C	-2.57561900	-0.84676400	-0.78644600
C	-3.13640900	0.57775200	-0.63865000
C	-3.80124700	-1.74328900	-0.73649000
C	-4.63794400	0.38250500	-0.51075100
O	-3.85194600	-2.93837800	-0.78849700

O	-5.48491800	1.21243600	-0.34575500
O	-4.94785000	-0.97018400	-0.59319900
N	3.64921000	-0.68876900	-0.21817100
C	4.94330600	-0.96146500	-0.84100900
C	3.71695500	-1.36884600	1.09065800
C	5.13028500	-2.46494400	-0.58507000
H	4.92399300	-0.68826000	-1.89993600
C	4.39325400	-2.71525800	0.76236700
H	4.33523400	-0.78298800	1.79317000
H	4.66213700	-3.04131000	-1.38754300
H	3.64963700	-3.50966900	0.65498400
H	5.76423100	-0.39798400	-0.35951000
H	6.18708400	-2.74264800	-0.54936600
H	5.07680200	-3.01456700	1.56137500
H	2.72588800	-1.47321000	1.53567900
H	-2.93387600	2.26112200	0.76715100
H	-2.06066000	-1.01128400	-1.73630500
H	-2.93938300	1.21084600	-1.50755500

Zero-point correction= 0.365930 (Hartree/Particle)
Thermal correction to Energy= 0.385364
Thermal correction to Enthalpy= 0.386308
Thermal correction to Gibbs Free Energy= 0.317329
Sum of electronic and zero-point Energies= -1129.924081
Sum of electronic and thermal Energies= -1129.904647
Sum of electronic and thermal Enthalpies= -1129.903703
Sum of electronic and thermal Free Energies= -1129.972682

	E (Thermal)	CV	S
	KCal/Mol	Cal/Mol-Kelvin	Cal/Mol-Kelvin
Total	241.819	79.823	145.178

1.36. (3aS,4S,11R,11aR)-6-(pyrrolidin-1-yl)-3a,4,11,11a-tetrahydro-4,11-ethenoanthra[2,3-c]furan-1,3-dione

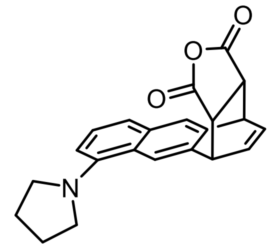

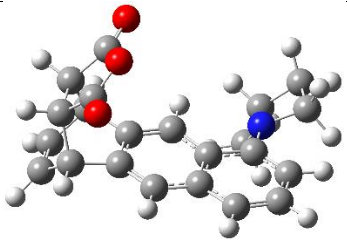

0 1

C	3.13849400	2.09458500	-0.30237000

C	2.56160900	0.85591300	-0.06937000
C	1.16409400	0.78700600	0.25919700
C	0.42810000	2.00385500	0.44031500
C	1.07341000	3.24980400	0.22581300
C	2.39164800	3.28587700	-0.16311700
C	0.47644000	-0.45494300	0.36371300
C	-0.95062000	1.93634700	0.79320700
C	-1.56660700	0.72198900	0.94970600
C	-0.84853000	-0.48402800	0.71654600
C	-1.72671800	-1.71897600	0.82584100
H	-1.17129700	-2.64193200	0.65369900
C	-2.44224900	-1.66754900	2.16800600
C	-3.11631400	-0.53436600	2.39148800
C	-3.03428500	0.47959200	1.25986200
H	4.18489200	2.16148000	-0.57705100
H	0.50477000	4.16663000	0.35182400
H	2.87936500	4.23862600	-0.34803100
H	-1.51022000	2.85979100	0.91740600
H	-2.39429300	-2.49959700	2.86230800
H	-3.68652100	-0.32698000	3.29062500
H	1.00222500	-1.37049300	0.11734700
C	-2.85563100	-1.55378200	-0.25858700
C	-3.62862000	-0.24920200	-0.00315000
N	3.28517300	-0.35409300	-0.17064200
C	4.53936200	-0.38282400	-0.92012200
C	3.58281100	-1.07977600	1.07839400
C	4.98578100	-1.84730100	-0.77664600
H	4.37459500	-0.06659600	-1.95392700
C	4.40609200	-2.29076600	0.59810400
H	4.18130500	-0.44550500	1.75629100
H	4.55729300	-2.44923200	-1.58223900
H	3.76779600	-3.17084200	0.48088000
H	5.30347700	0.28355800	-0.47778800
H	6.07332500	-1.94597400	-0.83015700
H	5.18727700	-2.54656000	1.31903500
H	2.66795300	-1.35289300	1.60738900
H	-3.57953600	1.40468800	1.45184300
C	-2.27227200	-1.38246100	-1.65195500
C	-3.42252200	0.56783200	-1.26878800
O	-3.83153700	1.66444800	-1.51991100
O	-1.58770700	-2.14536800	-2.26997400
O	-2.63631900	-0.14354600	-2.16801400
H	-3.49390100	-2.44075300	-0.25688600
H	-4.70105200	-0.40400200	0.13858900

Zero-point correction= 0.365885 (Hartree/Particle)
Thermal correction to Energy= 0.385285
Thermal correction to Enthalpy= 0.386230
Thermal correction to Gibbs Free Energy= 0.317361
Sum of electronic and zero-point Energies= -1129.923758
Sum of electronic and thermal Energies= -1129.904358
Sum of electronic and thermal Enthalpies= -1129.903413
Sum of electronic and thermal Free Energies= -1129.972282

	E (Thermal)	CV	S
	KCal/Mol	Cal/Mol-Kelvin	Cal/Mol-Kelvin
Total	241.770	79.871	144.946

2. Transition State optimized structures for 9,10-addition with B3LYP-D3/6-31G**

2.1. TS 9,10 addition anthracene + DMAD

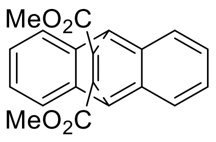

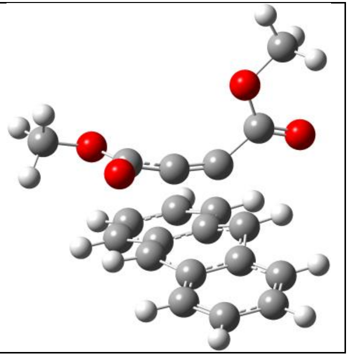

0 1

C	-0.81566700	0.54413500	1.25257100
C	-0.79432400	-0.87788200	1.08015000
C	0.36053300	-1.56807500	1.61126700
H	0.46365700	-2.62549500	1.38280200
C	0.37748400	1.12580300	1.86851700
H	0.41311500	2.20767300	1.94064300
C	1.06652800	0.35380400	2.84472500
C	1.07676000	-1.01772700	2.69131000
C	1.60424700	0.78093000	0.27499700
C	1.71448800	-0.43884900	-0.01689600
C	-1.81353500	-1.50679400	0.37999400
H	-1.77829600	-2.58120700	0.22125600
C	-1.86007500	1.28894400	0.73725800
H	-1.85787300	2.37048600	0.84501000

C	-2.90164700	-0.76709100	-0.12726300
C	-2.92959300	0.66069100	0.06075300
C	1.99302900	-1.61576700	-0.79119800
C	1.98768000	2.10127400	-0.24464800
O	1.22050900	-2.10971000	-1.59278100
O	1.59075300	3.17660000	0.16196100
O	3.20656500	-2.13653600	-0.48255600
O	2.86294200	1.97039400	-1.26578300
C	3.53785100	-3.33979700	-1.19636300
H	3.55928200	-3.15819500	-2.27416300
H	2.80709000	-4.12704800	-0.99015700
H	4.52522100	-3.62978800	-0.83727000
C	3.28918500	3.21058200	-1.85748300
H	3.78160100	3.84377600	-1.11456300
H	2.43531200	3.75368900	-2.27115600
H	3.98584400	2.93098900	-2.64740800
H	1.73630100	-1.64592300	3.28125700
H	1.71797800	0.84461400	3.56054400
C	-4.03160000	1.39716200	-0.45915200
H	-4.05051700	2.47446800	-0.31689400
C	-3.97597900	-1.38726700	-0.82810200
H	-3.95006900	-2.46429500	-0.97096400
C	-5.02184000	-0.64357300	-1.31719100
H	-5.83320600	-1.12919100	-1.85118000
C	-5.04996100	0.76442200	-1.13016000
H	-5.88298400	1.33990500	-1.52300900

ν_{imag} = -389.7 cm^{-1}

Zero-point correction= 0.309938 (Hartree/Particle)
Thermal correction to Energy= 0.331057
Thermal correction to Enthalpy= 0.332001
Thermal correction to Gibbs Free Energy= 0.257365
Sum of electronic and zero-point Energies= -1072.315521
Sum of electronic and thermal Energies= -1072.294402
Sum of electronic and thermal Enthalpies= -1072.293458
Sum of electronic and thermal Free Energies= -1072.368094

	E (Thermal)	CV	S
	KCal/Mol	Cal/Mol-Kelvin	Cal/Mol-Kelvin
Total	207.741	79.410	157.085

2.2. TS 9,10 addition 1,5-Dimethoxyanthracene + DMAD

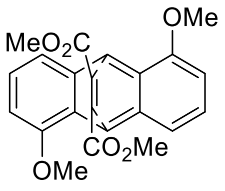

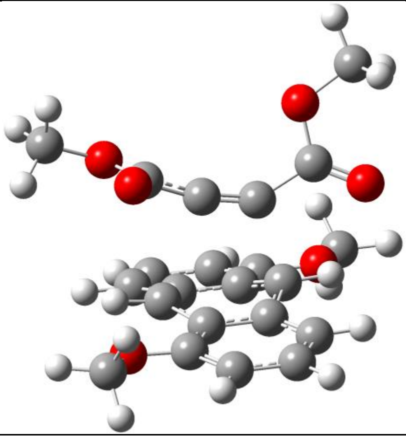

0 1

C	-0.32697300	-1.86405200	0.00073400
C	-1.30904400	-1.12724400	-0.70738500
C	-0.89158000	0.11355600	-1.30901500
H	-1.64925400	0.70739100	-1.80578200
C	1.00601100	-1.35832300	-0.03853200
H	1.75837600	-1.82710200	0.58644600
C	1.44395800	-0.52796700	-1.10408100
C	0.46174400	0.22380100	-1.80166600
C	0.25964600	0.67662600	1.18378900
C	-0.58477300	1.18142700	0.40122400
C	2.82289600	-0.31207700	-1.39310500
C	0.85064900	1.15568800	-2.78529200
H	0.09356800	1.74683700	-3.28960600
C	3.18694400	0.58849400	-2.38091900
H	4.22883700	0.75265200	-2.62640600
C	2.19176600	1.31422300	-3.07277500
H	2.50086800	2.01894900	-3.83918200
C	-2.66676800	-1.53437700	-0.63556900
C	-0.70091100	-2.99419900	0.76788000
H	0.05734200	-3.54127700	1.31900100
C	-3.01266900	-2.66789400	0.08648100
H	-4.04047200	-3.00628400	0.13055200
C	-2.02064400	-3.38818300	0.78931300
H	-2.31732600	-4.26569700	1.35614500
C	-1.38211300	2.40715300	0.25158700
C	1.24546400	0.45751000	2.19638200
O	-1.82924100	2.84519200	-0.79058400
O	2.36641100	0.93736000	2.18578700
O	-1.55239500	3.01467900	1.44746400
O	0.79050500	-0.38771000	3.15878100

C	-2.29491700	4.24494200	1.39543000
H	-3.29577700	4.07556500	0.98889400
H	-1.78213500	4.97924900	0.76834200
H	-2.35229500	4.59409400	2.42625400
C	1.74935800	-0.69255300	4.18277700
H	2.06930800	0.21525600	4.70149000
H	2.63210000	-1.17832900	3.75622500
H	1.23803500	-1.36591400	4.87094800
O	-3.54642500	-0.74076500	-1.31177500
C	-4.92704000	-1.06433900	-1.25561400
H	-5.43972600	-0.29517700	-1.83467800
H	-5.30209100	-1.05349900	-0.22389400
H	-5.12964200	-2.04768100	-1.70000200
O	3.69132500	-1.04970400	-0.64738200
C	5.07527100	-0.73919000	-0.72652200
H	5.48363900	-0.96249500	-1.72077600
H	5.56820100	-1.37197200	0.01274500
H	5.25936100	0.31489500	-0.48577900

ν_{imag} = -396.8 cm^{-1}

Zero-point correction= 0.375499 (Hartree/Particle)
Thermal correction to Energy= 0.402000
Thermal correction to Enthalpy= 0.402944
Thermal correction to Gibbs Free Energy= 0.316447
Sum of electronic and zero-point Energies= -1301.321270
Sum of electronic and thermal Energies= -1301.294770
Sum of electronic and thermal Enthalpies= -1301.293826
Sum of electronic and thermal Free Energies= -1301.380322

	E (Thermal)	CV	S
	KCal/Mol	Cal/Mol-Kelvin	Cal/Mol-Kelvin
Total	252.259	97.471	182.047

2.3. TS 9,10 addition *N*[1],*N*[1],*N*[5],*N*[5]-tetramethylanthracene-1,5-diamine + DMAD

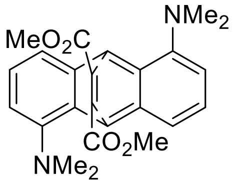

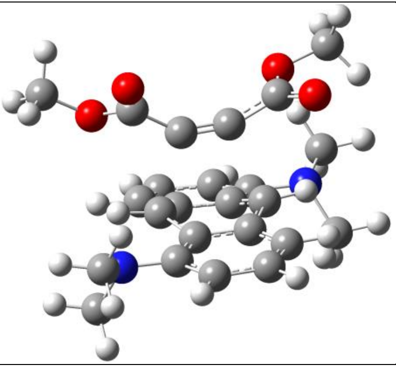

0 1

C	-2.80033100	-1.48134700	-0.08790500
C	-0.64999500	-2.86659100	1.14323700
C	-1.45800700	-1.05573700	-0.30230200
C	-3.02495900	-2.58451200	0.72699900
C	-1.95483700	-3.26343900	1.34731600
C	-0.38601300	-1.76471100	0.29891900
H	-4.03665400	-2.93634500	0.89506900
H	-2.17042000	-4.11372200	1.98761200
H	0.17504300	-3.37562000	1.63143300
C	-1.11456200	0.19246000	-0.92831500
H	-1.92462000	0.82152300	-1.27797100
C	0.92313600	-1.20542300	0.13611200
H	1.74329000	-1.66388900	0.67757300
C	1.23698000	-0.41282000	-1.01448300
C	1.71853900	1.49534500	-3.00489200
C	0.16750800	0.33660700	-1.56994300
C	2.57011500	-0.19431800	-1.46820700
C	2.78668400	0.74939200	-2.46458900
C	0.42407700	1.30254100	-2.56525200
H	3.78977000	0.92200000	-2.83853000
H	-0.39599100	1.89608800	-2.95688800
H	1.92618000	2.22911400	-3.77839900
C	-0.53099100	1.18922000	0.88262100
C	0.45229300	0.60991600	1.40409400
N	-3.84686900	-0.73313500	-0.69550600
N	3.61868300	-0.93258300	-0.85011400
C	3.68240000	-2.34350700	-1.24003200
H	4.32927700	-2.88703800	-0.54330100
H	4.08191500	-2.47728300	-2.26035600
H	2.68791700	-2.79205600	-1.20549200
C	4.93252900	-0.30657500	-0.85092000
H	5.57816600	-0.84112200	-0.14631100

H	4.84203000	0.73075400	-0.52014400
H	5.43352000	-0.32129300	-1.83532000
C	-5.14663200	-0.78995300	-0.04217900
H	-5.02822600	-0.61626000	1.02984700
H	-5.67574800	-1.74878600	-0.18804300
H	-5.78211600	0.00153500	-0.45260400
C	-3.95762800	-0.90812200	-2.14600600
H	-4.39911000	-1.88341700	-2.41533400
H	-2.97386500	-0.84255500	-2.61396600
H	-4.59085300	-0.11771100	-2.56285300
C	-1.49162000	2.27821200	1.02599900
O	-2.17680100	2.48013800	2.00614600
O	-1.54092200	3.04102000	-0.09529900
C	-2.46765200	4.13760500	-0.03361600
H	-2.37672800	4.65212800	-0.99026100
H	-2.21694400	4.81116300	0.79006200
H	-3.48791000	3.77193800	0.11198200
C	1.55700000	0.38680300	2.30663400
O	1.57015100	-0.40789400	3.22678000
O	2.62739400	1.13191100	1.92992500
C	3.83412700	0.85116900	2.65692300
H	3.69053100	1.00302800	3.72955000
H	4.57851500	1.54529900	2.26646400
H	4.14880500	-0.18215000	2.48381200

ν_{imag} = -399.2 cm^{-1}

Zero-point correction=	0.456680 (Hartree/Particle)
Thermal correction to Energy=	0.486126
Thermal correction to Enthalpy=	0.487070
Thermal correction to Gibbs Free Energy=	0.394724
Sum of electronic and zero-point Energies=	-1340.140804
Sum of electronic and thermal Energies=	-1340.111359
Sum of electronic and thermal Enthalpies=	-1340.110414
Sum of electronic and thermal Free Energies=	-1340.202760

	E (Thermal)	CV	S
	KCal/Mol	Cal/Mol-Kelvin	Cal/Mol-Kelvin
Total	305.048	109.422	194.357

2.4. TS 9,10 addition 1,5-di(pyrrolidin-1-yl)anthracen + DMAD

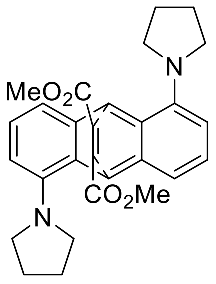

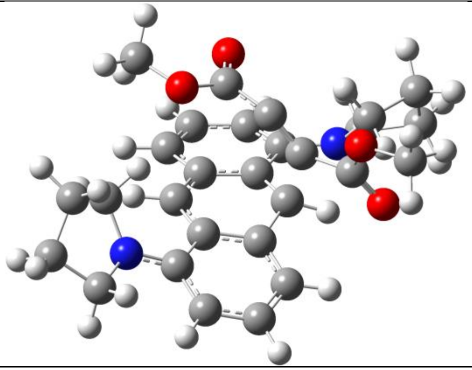

0 1

C	1.92000700	2.57616400	-2.28472000
C	0.62884300	2.19180600	-1.95954200
C	0.42151800	0.93877800	-1.36799700
C	1.54270300	0.07008300	-1.11118400
C	2.89391700	0.58473800	-1.26714400
C	3.03996100	1.80197300	-1.94053900
C	-0.89059600	0.50375000	-0.90911900
C	1.25609200	-1.26588200	-0.83582300
C	-0.05375200	-1.80647600	-0.82220700
C	-1.16982000	-0.92391600	-0.86758200
C	-2.49112800	-1.46922000	-0.86753500
C	-2.62417000	-2.85907100	-0.95849500
C	-1.51354500	-3.71897100	-0.91950500
C	-0.23916800	-3.21179300	-0.81613300
H	2.08433700	3.52485400	-2.78849900
H	4.02427100	2.18983700	-2.16659200
H	-3.61129000	-3.29934700	-1.02055500
H	-1.67638200	-4.79218400	-0.95234700
C	-0.32318800	0.42335200	1.79237200
C	-0.74375900	1.15212700	0.82930800
H	-1.71212200	1.11045800	-1.27740200
H	2.06883200	-1.97430800	-0.73976300
C	-0.04366200	-0.90346800	2.20260300
C	-1.15545800	2.57349400	0.80309800
O	-0.85098000	3.20234800	1.95792000
O	-1.71350300	3.13912900	-0.12341600
O	1.27720200	-1.11373800	2.51826500
O	-0.89893600	-1.77336000	2.33050200
C	-1.25279300	4.57993200	2.01603900
H	-0.93802200	4.93297600	2.99806300
H	-0.76850300	5.15897800	1.22485500
H	-2.33647100	4.67401700	1.90294400

C	1.58225200	-2.46765900	2.87118400
H	1.35867200	-3.14654100	2.04150100
H	2.65020700	-2.48107000	3.09564000
H	1.00572000	-2.78810300	3.74303900
C	5.34543400	0.39258900	-1.03894900
C	3.96143000	-0.94107600	0.43428500
C	6.23344300	-0.46491100	-0.13404800
H	5.42502500	1.45077400	-0.74483200
H	5.60112800	0.32198100	-2.10191400
C	5.32598500	-0.68638500	1.08315100
H	3.87070500	-2.00697000	0.18437700
H	3.11452900	-0.67147800	1.07057700
H	6.46242100	-1.42180600	-0.61702100
H	7.17833500	0.02961000	0.10497500
H	5.64255800	-1.51499700	1.72245900
H	5.28836800	0.22108600	1.69650000
C	-4.92066400	-1.15914300	-1.23625300
C	-3.79032100	0.33260200	0.28597000
C	-5.87728800	-0.02725900	-0.86602300
H	-5.20487700	-2.06306200	-0.66928700
H	-4.92032900	-1.41939200	-2.30024100
C	-5.31303000	0.40676800	0.49445800
H	-3.39864100	1.31479500	0.01280400
H	-3.25722100	-0.01358500	1.17627400
H	-5.79923700	0.78756200	-1.59446200
H	-6.91981300	-0.35454000	-0.82309000
H	-5.63760300	1.40338100	0.80575200
H	-5.62775100	-0.30453000	1.26656300
N	3.98650700	-0.11351100	-0.79550200
N	-3.61219200	-0.63095700	-0.84014500
H	0.62678200	-3.86571900	-0.77043800
H	-0.21956700	2.83599800	-2.15456700

ν_{imag} = -301.1 cm^{-1}

Zero-point correction= 0.531850 (Hartree/Particle)
Thermal correction to Energy= 0.562416
Thermal correction to Enthalpy= 0.563361
Thermal correction to Gibbs Free Energy= 0.469316
Sum of electronic and zero-point Energies= -1494.934758
Sum of electronic and thermal Energies= -1494.904192
Sum of electronic and thermal Enthalpies= -1494.903248
Sum of electronic and thermal Free Energies= -1494.997293

	E (Thermal)	CV	S
	KCal/Mol	Cal/Mol-Kelvin	Cal/Mol-Kelvin
Total	352.922	117.830	197.934

2.5. TS 9,10 addition 1-(pyrrolidin-1-yl)anthracen + DMAD

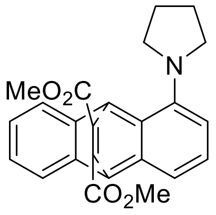

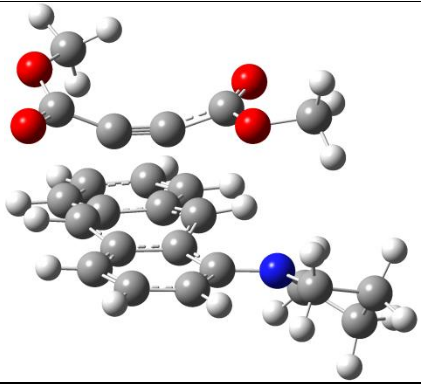

0 1

C	3.31678300	-0.89188200	-3.02683800
C	3.07814900	0.28424700	-2.34182100
C	1.85584100	0.46749800	-1.65499200
C	0.89083300	-0.57419000	-1.65134900
C	1.17035000	-1.78070600	-2.32885300
C	2.35772500	-1.93015700	-3.02048200
C	1.56854600	1.59258000	-0.82433800
C	-0.26523100	-0.38353500	-0.82069200
C	-0.77075800	0.93787200	-0.57090000
C	0.21958600	1.96639700	-0.52803400
C	-0.09178900	3.25399500	-0.04258500
C	-1.37834800	3.51175200	0.37807200
C	-2.37619000	2.52436900	0.31930000
C	-2.11491000	1.23335600	-0.15699300
H	2.33703400	2.35028300	-0.69540000
H	4.24748600	-1.02442300	-3.57031100
H	3.81951700	1.07842800	-2.32719000
H	0.43940900	-2.58476700	-2.30560800
H	2.56036800	-2.85100800	-3.55902400
H	-0.93197800	-1.22575000	-0.72233100
H	-1.63916500	4.49455800	0.75983100
H	-3.37174700	2.78669400	0.65232300
C	-4.44019000	0.57691000	0.39194400
C	-3.24700400	-0.77452800	-1.21286200
C	-5.29065700	-0.63790900	0.01700400
H	-4.88216300	1.48638500	-0.04850800
H	-4.35667900	0.73918800	1.47237800
C	-4.75923100	-0.96974000	-1.38314000

H	-2.79518800	-1.71412700	-0.86557800
H	-2.74306300	-0.48285400	-2.14051000
H	-5.09446700	-1.46840300	0.70544300
H	-6.36165500	-0.42114600	0.04100400
H	-5.01362600	-1.97628400	-1.72625200
H	-5.15463100	-0.25249600	-2.11157200
N	-3.12743900	0.27893200	-0.18869800
C	0.86356800	-0.54991400	1.05629100
C	1.74192800	0.34240400	1.10453000
H	0.68691000	4.00626500	0.02115900
C	0.33879500	-1.72887300	1.72982000
C	2.84829200	1.06491100	1.70769400
O	2.74582400	2.17752700	2.17868100
O	4.05131300	0.44037500	1.68349700
O	1.02956300	-2.58332300	2.25303400
O	-1.01349100	-1.77914500	1.69651200
C	-1.59907000	-2.89714100	2.38337700
H	-1.26025600	-3.84048600	1.94649400
H	-1.32815900	-2.88483200	3.44235500
H	-2.67602900	-2.78035000	2.26199600
C	4.14069800	-0.91640000	1.20037400
H	3.45970400	-1.57956200	1.74112400
H	3.92352200	-0.96689400	0.12884800
H	5.17497700	-1.21230000	1.37925700

ν_{imag} = -376.7 cm^{-1}

Zero-point correction= 0.420932 (Hartree/Particle)
Thermal correction to Energy= 0.446840
Thermal correction to Enthalpy= 0.447784
Thermal correction to Gibbs Free Energy= 0.363171
Sum of electronic and zero-point Energies= -1283.621187
Sum of electronic and thermal Energies= -1283.595279
Sum of electronic and thermal Enthalpies= -1283.594334
Sum of electronic and thermal Free Energies= -1283.678947

	E (Thermal)	CV	S
	KCal/Mol	Cal/Mol-Kelvin	Cal/Mol-Kelvin
Total	280.396	98.531	178.083

2.6. TS 9,10 addition anthracene + maleic anhydride

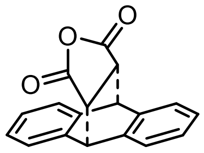

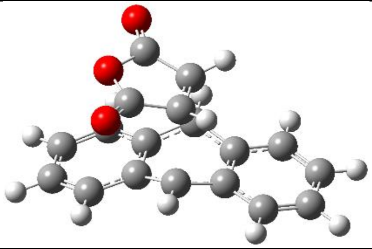

0 1

C	-2.71390000	-2.18500200	0.70529600
C	-1.66034200	-1.62949200	1.40727100
C	-0.55589000	-1.09457700	0.71091500
C	-0.55585400	-1.09470100	-0.71078700
C	-1.66026900	-1.62980100	-1.40706200
C	-2.71385300	-2.18517000	-0.70501200
C	0.52804800	-0.39847800	1.35200700
C	0.52810800	-0.39866500	-1.35197200
C	1.81926800	-0.38949700	-0.71020900
C	1.81922000	-0.38939700	0.71025400
C	3.03852700	-0.25784600	1.40507400
H	3.03345800	-0.24345000	2.49149900
C	4.22941700	-0.16646900	0.70499400
C	4.22945800	-0.16653900	-0.70485100
C	3.03860600	-0.25799300	-1.40498400
H	-3.55588300	-2.61093400	1.24189100
H	-1.67156300	-1.59591600	2.49218600
H	-1.67145400	-1.59645700	-2.49198300
H	-3.55580500	-2.61122900	-1.24155400
H	0.49241900	-0.29840400	-2.43426000
H	5.16955300	-0.09003800	1.24265400
H	5.16962500	-0.09015700	-1.24246400
H	3.03359100	-0.24371700	-2.49141000
H	0.49232900	-0.29810600	2.43427300
C	0.00630800	1.63197200	0.70586900
C	0.00638300	1.63182600	-0.70600300
C	-1.41019800	1.65066100	1.14044700
C	-1.41009800	1.65044500	-1.14071900
O	-1.88395200	1.69972900	2.24362200
O	-1.88375000	1.69939200	-2.24394200
O	-2.21164000	1.54807100	-0.00016300
H	0.76034400	2.08671500	1.33229600
H	0.76048700	2.08649300	-1.33244100

ν_{imag} = -459.9 cm^{-1}

Zero-point correction=	0.251910 (Hartree/Particle)	
Thermal correction to Energy=	0.266837	
Thermal correction to Enthalpy=	0.267781	
Thermal correction to Gibbs Free Energy=	0.210070	
Sum of electronic and zero-point Energies=	-918.594245	
Sum of electronic and thermal Energies=	-918.579317	
Sum of electronic and thermal Enthalpies=	-918.578373	
Sum of electronic and thermal Free Energies=	-918.636085	

	E (Thermal)	CV	S
	KCal/Mol	Cal/Mol-Kelvin	Cal/Mol-Kelvin
Total	167.443	61.772	121.464

2.7. TS 9,10 addition 1,5-dimethoxyanthracene + maleic anhydride

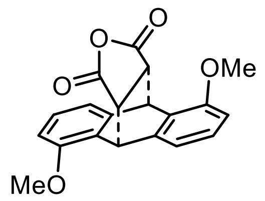

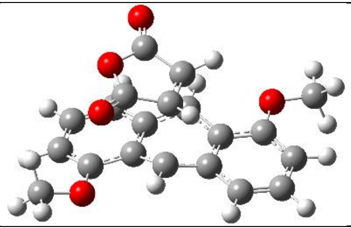

0 1

C	3.03000800	-0.22846600	-1.83702400
C	2.35556400	0.73722500	-1.10370400
C	0.99037200	0.53981000	-0.77126800
C	0.34549000	-0.66199100	-1.15224200
C	1.05050700	-1.64377900	-1.88114500
C	2.36812500	-1.41070500	-2.22463400
C	0.27839300	1.40678300	0.12945200
C	-0.96777600	-0.87412100	-0.59645500
C	-1.80661700	0.27228100	-0.35614200
C	-1.16057100	1.47099500	0.02767600
C	-1.92040700	2.59653300	0.40852700
H	-1.41620900	3.50976100	0.70874200
C	-3.30005500	2.52352400	0.37452200
C	-3.96229000	1.33534200	0.00378600
C	-3.22445200	0.20849100	-0.33713900
H	4.07011900	-0.08693400	-2.10453300
H	0.55807500	-2.57301700	-2.14654500
H	2.91828200	-2.15583300	-2.79100900
H	-1.45037500	-1.82652900	-0.78792600

H	-3.89577000	3.39183900	0.63961300
H	-5.04492400	1.31388100	-0.00474700
H	0.80961200	2.27547400	0.50353700
C	0.29122600	0.10192700	1.86049200
C	-0.37677500	-1.09159200	1.49278500
C	1.74021600	-0.21551600	1.91022800
C	0.65608600	-2.13845600	1.33244700
O	2.68053600	0.47222200	2.21137800
O	0.55037800	-3.30996900	1.08823800
O	1.90996200	-1.52983300	1.47543100
O	-3.75507800	-1.00252500	-0.66924200
O	2.90587100	1.89839500	-0.65254400
C	4.31357000	1.91814100	-0.42580500
H	4.51757700	2.84998200	0.10315700
H	4.61219500	1.06936000	0.19877000
H	4.87738800	1.91130600	-1.36698700
C	-5.16934700	-1.14424500	-0.66852300
H	-5.36715300	-2.18022700	-0.94589700
H	-5.59317800	-0.94614300	0.32427500
H	-5.64000200	-0.47612000	-1.40092000
H	-1.37044900	-1.37611600	1.80852800
H	-0.10616400	0.85991100	2.52086000

ν_{imag} = -462.0 cm^{-1}

Zero-point correction= 0.317316 (Hartree/Particle)
Thermal correction to Energy= 0.337578
Thermal correction to Enthalpy= 0.338522
Thermal correction to Gibbs Free Energy= 0.268760
Sum of electronic and zero-point Energies= -1147.591949
Sum of electronic and thermal Energies= -1147.571687
Sum of electronic and thermal Enthalpies= -1147.570743
Sum of electronic and thermal Free Energies= -1147.640504

	E (Thermal)	CV	S
	KCal/Mol	Cal/Mol-Kelvin	Cal/Mol-Kelvin
Total	211.833	79.964	146.826

2.8. TS 9,10 addition N^1,N^1,N^5,N^5-tetramethylanthracene-1,5-diamine + maleic anhydride

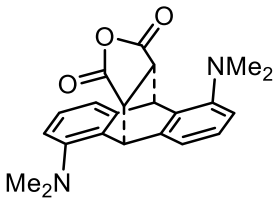

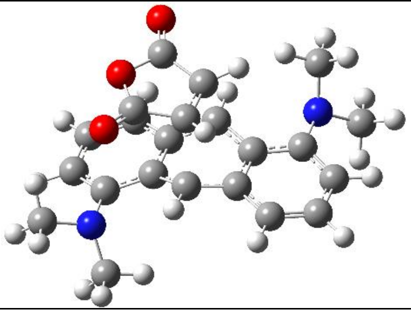

0 1

C	-3.01981600	-0.36055700	1.83062800
C	-2.50112900	0.54217800	0.91039300
C	-1.12128700	0.45045300	0.57535400
C	-0.30526100	-0.53756000	1.17903600
C	-0.86676000	-1.44987300	2.10002300
C	-2.20754900	-1.35321200	2.41440900
C	-0.52867400	1.18198800	-0.51457900
C	1.03519600	-0.66330700	0.67170600
C	1.71045500	0.50428200	0.16464100
C	0.88455600	1.48006500	-0.45174900
C	1.45272500	2.62944300	-1.03302500
H	0.81058100	3.36470500	-1.50896600
C	2.81959400	2.82262300	-0.95223000
C	3.64792900	1.85672500	-0.35250800
C	3.12577500	0.68043900	0.18168700
H	-4.06795900	-0.30656400	2.10353000
H	-0.24433500	-2.22639900	2.53241700
H	-2.64869900	-2.04857100	3.12208000
H	1.63697300	-1.46839800	1.07822000
H	3.26783400	3.72154100	-1.36510900
H	4.71748300	2.03155400	-0.32364300
H	-1.17789800	1.86008800	-1.05880200
C	-0.36818700	-0.43478300	-1.91506700
C	0.49765400	-1.40352200	-1.35650300
C	-1.74109900	-1.00092500	-1.83819400
C	-0.32987900	-2.55087800	-0.93637100
O	-2.78622300	-0.56808800	-2.24421200
O	-0.01432900	-3.61055100	-0.46383900
O	-1.67223700	-2.20611200	-1.13710400
N	3.94488800	-0.31560200	0.78498200
N	-3.27914700	1.53667600	0.25830700
C	-3.11009400	2.88373000	0.80040900

H	-3.54315500	3.61325400	0.10793800
H	-3.59597400	3.00988200	1.78410900
H	-2.04735400	3.11050900	0.91817100
C	-4.66940000	1.19463700	-0.00738100
H	-5.31250100	1.20747300	0.89056900
H	-5.07837000	1.92104200	-0.71693400
H	-4.71053300	0.20632400	-0.46943500
C	5.19782400	0.15078200	1.36615400
H	5.60255800	-0.63848200	2.00755400
H	5.97167600	0.39919300	0.61805400
H	5.01451200	1.03437500	1.98196900
C	4.14879600	-1.51839900	-0.02749900
H	4.78166400	-1.32531900	-0.91092400
H	4.63464100	-2.28701400	0.58151700
H	3.19747700	-1.92476900	-0.37221100
H	-0.11651300	0.21624800	-2.74086600
H	1.52179700	-1.56615800	-1.65702600

ν_{imag} = -456.0 cm^{-1}

Zero-point correction=	0.398318 (Hartree/Particle)
Thermal correction to Energy=	0.421508
Thermal correction to Enthalpy=	0.422453
Thermal correction to Gibbs Free Energy=	0.347114
Sum of electronic and zero-point Energies=	-1186.410229
Sum of electronic and thermal Energies=	-1186.387039
Sum of electronic and thermal Enthalpies=	-1186.386095
Sum of electronic and thermal Free Energies=	-1186.461433

	E (Thermal)	CV	S
	KCal/Mol	Cal/Mol-Kelvin	Cal/Mol-Kelvin
Total	264.501	92.063	158.562

2.9. TS 9,10 addition 1,5-di(pyrrolidin-1-yl)anthracen + maleic anhydride

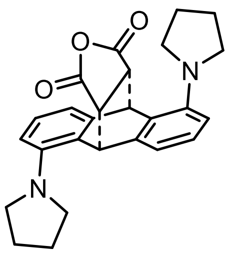

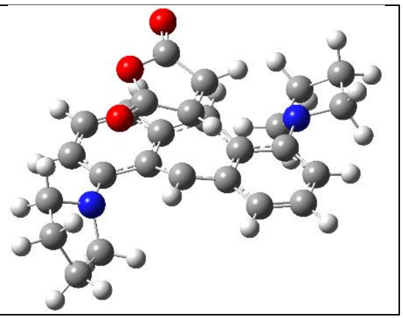

0 1

C	2.62438100	-1.50012800	-1.81055000
C	2.43610700	-0.34020000	-1.06664800
C	1.12292700	-0.02903500	-0.61347100
C	0.04360100	-0.89702800	-0.91532500
C	0.27044900	-2.07577000	-1.65861100
C	1.54824100	-2.36107100	-2.09968100
C	0.85328400	1.01225300	0.34410700
C	-1.20652000	-0.60503700	-0.26125700
C	-1.54226900	0.77078900	0.01461400
C	-0.45861800	1.61401900	0.36597600
C	-0.70129800	2.90775800	0.87232500
H	0.13532000	3.53808200	1.15881500
C	-2.00428500	3.35060700	0.99997600
C	-3.08768300	2.53300800	0.62601700
C	-2.88121900	1.24727200	0.12902500
H	3.61740100	-1.75249300	-2.16581600
H	-0.55397000	-2.75407200	-1.85246500
H	1.73271100	-3.26408500	-2.67384500
H	-2.01476500	-1.32012800	-0.36845100
H	-2.20395400	4.34826900	1.38012200
H	-4.09500900	2.92139800	0.72016800
H	1.69871300	1.59412200	0.69558000
C	0.50750800	-0.22959300	2.08375800
C	-0.58933300	-1.06945800	1.78090500
C	1.72553500	-1.07528400	2.03316700
C	-0.04819100	-2.42596500	1.54757000
O	2.87311600	-0.80608800	2.27213400
O	-0.61198100	-3.46095200	1.31098600
O	1.34904600	-2.34664800	1.59519400
N	-3.93751700	0.38653400	-0.23216200
C	-5.29791400	0.69234400	0.20789100
C	-4.07905400	0.01431500	-1.65355700
C	-6.11802200	-0.46644700	-0.38272900
H	-5.34188800	0.75681500	1.29892100
C	-5.33613300	-0.87620100	-1.66452400
H	-4.23348600	0.91764000	-2.26894400
H	-7.14767600	-0.16519500	-0.59235000
H	-5.91819200	-0.72241100	-2.57692100
N	3.48808100	0.51903000	-0.69320900
C	4.83011200	-0.01430900	-0.48350800
C	3.62782600	1.80675900	-1.39004900
C	5.57871600	1.23361500	-0.00636700
H	4.79289000	-0.81792300	0.25611500
C	4.97535600	2.37197800	-0.86912500

H	3.66066300	1.66486800	-2.48357300
H	5.35305000	1.39612500	1.05132200
H	4.83646600	3.28740000	-0.28759000
H	5.29095200	-0.40432000	-1.41067700
H	6.66305900	1.14593100	-0.11640700
H	5.62929600	2.62022400	-1.71017000
H	2.77308600	2.45640400	-1.17558600
H	-3.18553600	-0.48767200	-2.02821500
H	-5.06053600	-1.93342000	-1.62580100
H	-6.15355500	-1.29970500	0.32384800
H	-5.65782600	1.65360600	-0.20334400
H	0.47861500	0.61390600	2.75910900
H	-1.59224200	-0.95797500	2.16768300

ν_{imag} = --448.5 cm^{-1}

Zero-point correction= 0.471903 (Hartree/Particle)
Thermal correction to Energy= 0.497158
Thermal correction to Enthalpy= 0.498102
Thermal correction to Gibbs Free Energy= 0.415514
Sum of electronic and zero-point Energies= -1341.197709
Sum of electronic and thermal Energies= -1341.172455
Sum of electronic and thermal Enthalpies= -1341.171510
Sum of electronic and thermal Free Energies= -1341.254098

	E (Thermal)	CV	S
	KCal/Mol	Cal/Mol-Kelvin	Cal/Mol-Kelvin
Total	311.971	100.463	173.821

2.10. TS endo 9,10 addition 1,5-di(pyrrolidin-1-yl)anthracen + maleic anhydride

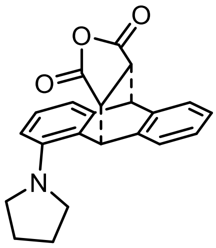

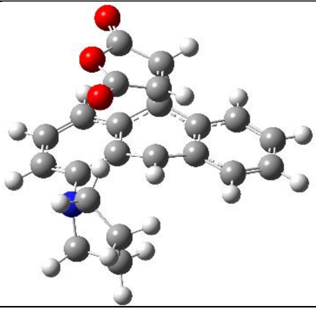

0 1

C	2.04024800	-1.64592400	-1.71648300
C	1.82351700	-0.60517200	-0.81656000
C	0.47193000	-0.17472100	-0.60979300
C	-0.58644600	-0.82816800	-1.30995100
C	-0.31946300	-1.90756600	-2.17268400
C	0.98982600	-2.29403000	-2.38607600
C	0.06059000	0.85570700	0.30264400
C	-1.93181500	-0.39307300	-1.01695700
C	-2.14875400	1.00035000	-0.70993500
C	-1.10521700	1.64344500	0.00250500
C	-1.28772900	2.96391500	0.46403700
H	-0.49029800	3.44865000	1.02095700
C	-2.46568800	3.63633400	0.18859300
C	-3.50243000	2.99572000	-0.52092600
C	-3.35501200	1.68685800	-0.94917000
H	3.06623800	-1.95898800	-1.87827900
H	-1.14266000	-2.41414100	-2.66571000
H	1.21270100	-3.11289800	-3.06314100
H	-2.74091400	-0.94067800	-1.49492200
H	-2.59601600	4.66133000	0.52196200
H	-4.42291500	3.53289600	-0.72834100
H	0.80180000	1.31426000	0.94176600
C	-1.09150600	-0.48186900	1.75676400
C	-2.11539900	-1.07206400	0.98301300
C	-0.08685400	-1.52863200	2.01611900
C	-1.73268200	-2.49343900	0.77357100
O	0.91143900	-1.51034600	2.68934600
O	-2.32641200	-3.38047100	0.22273800
O	-0.45698100	-2.67520200	1.30362400
N	2.97916900	0.00831600	-0.26980700
C	3.11306500	0.39746100	1.14067600
C	3.62798700	1.06259800	-1.06124200

C	3.29098400	1.94992800	1.16153500
H	2.27940900	0.03143900	1.73803800
C	3.26335100	2.36697200	-0.32715100
H	4.71858700	0.91761100	-1.05466000
H	2.51083400	2.45368500	1.74229600
H	2.25377000	2.67517700	-0.62205100
H	4.01341000	-0.07955900	1.54941300
H	4.24593200	2.21541500	1.62508400
H	3.94412000	3.19335100	-0.55335700
H	3.28959800	1.02858000	-2.09921200
H	-4.15812700	1.18530400	-1.48209700
H	-1.21437400	0.33047300	2.45752200
H	-3.16467500	-0.81975400	1.05253800

ν_{imag} = -454.1 cm^{-1}

Zero-point correction=	0.361760 (Hartree/Particle)
Thermal correction to Energy=	0.381711
Thermal correction to Enthalpy=	0.382655
Thermal correction to Gibbs Free Energy=	0.313477
Sum of electronic and zero-point Energies=	-1129.885643
Sum of electronic and thermal Energies=	-1129.865692
Sum of electronic and thermal Enthalpies=	-1129.864747
Sum of electronic and thermal Free Energies=	-1129.933925

	E (Thermal)	CV	S
	KCal/Mol	Cal/Mol-Kelvin	Cal/Mol-Kelvin
Total	239.527	81.371	145.597

2.11. TS exo 9,10 addition 1,5-di(pyrrolidin-1-yl)anthracen + maleic anhydride

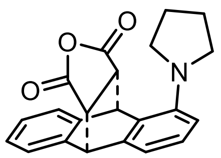

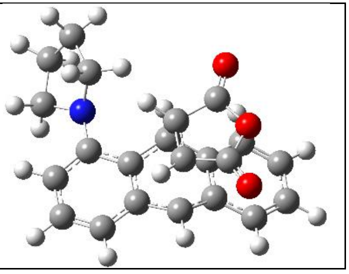

0 1

C	-2.93299900	-1.44448400	-2.43818400
C	-1.65436300	-1.50423100	-1.91541100
C	-1.13813500	-0.40601900	-1.19516500
C	-1.95262000	0.73540900	-0.97930600

C	-3.26850600	0.76092900	-1.48537000
C	-3.74161100	-0.30939900	-2.22264000
C	0.13873300	-0.42479600	-0.53415300
C	-1.40426600	1.74564800	-0.11212300
C	0.02471300	1.96349900	-0.12551600
C	0.83654500	0.81478800	-0.31521600
C	2.25557200	0.91861700	-0.19790100
C	2.80596000	2.18785400	-0.02153600
C	1.99222100	3.32468400	0.13267400
C	0.61359700	3.21805000	0.12311800
H	-3.32489200	-2.28262200	-3.00591600
H	-1.03872600	-2.38816100	-2.05084700
H	-4.74894300	-0.28389100	-2.62648200
H	-2.04221100	2.59119200	0.13516000
H	3.88232200	2.30678200	0.02059600
H	2.46122500	4.29299700	0.28053200
H	-0.01656000	4.09003100	0.27021000
H	0.72363900	-1.33156800	-0.63389600
C	-0.60725900	-0.51678700	1.55474400
C	-1.43536800	0.61514400	1.72926500
C	-1.48869000	-1.69187500	1.41021100
C	-2.83652000	0.12581900	1.70149500
O	-1.21786200	-2.85680900	1.28281700
O	-3.86850700	0.72024000	1.86042500
O	-2.81216000	-1.23677700	1.39284800
H	0.38005300	-0.63934600	1.97410100
H	-1.19060300	1.47548100	2.33642000
C	4.46237700	-0.10731700	-0.66832700
C	3.01373200	-1.22091600	0.79972100
C	4.92778000	-1.56474600	-0.70185900
H	5.03914400	0.45196600	0.09190200
H	4.56379400	0.41695200	-1.62291900
C	4.15569400	-2.21547900	0.47403100
H	2.04201100	-1.71341200	0.87051500
H	3.19952700	-0.71572100	1.76243400
H	4.63130200	-2.02019700	-1.65097100
H	6.01253600	-1.65942500	-0.60479300
H	3.76616700	-3.19932600	0.20132600
H	4.79838400	-2.35243400	1.34790100
N	3.04996400	-0.24296600	-0.31030800
H	-3.89918800	1.62186100	-1.28714300

ν_{imag} = -454.9 cm^{-1}

Zero-point correction= 0.362077 (Hartree/Particle)
Thermal correction to Energy= 0.382139
Thermal correction to Enthalpy= 0.383083
Thermal correction to Gibbs Free Energy= 0.313042
Sum of electronic and zero-point Energies= -1129.894018
Sum of electronic and thermal Energies= -1129.873957
Sum of electronic and thermal Enthalpies= -1129.873013
Sum of electronic and thermal Free Energies= -1129.943054

	E (Thermal)	CV	S
	KCal/Mol	Cal/Mol-Kelvin	Cal/Mol-Kelvin
Total	239.796	81.136	147.414

3. Transition state optimized structures for 1,4 addition with B3LYP-D3/6-31G**

3.1. TS 1,4-addition anthracene + DMAD

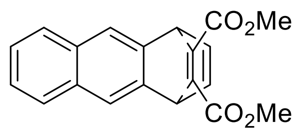

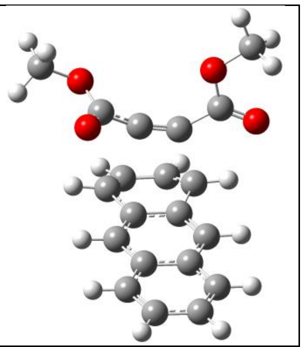

0 1

C	-0.81566700	0.54413500	1.25257100
C	-0.79432400	-0.87788200	1.08015000
C	0.36053300	-1.56807500	1.61126700
H	0.46365700	-2.62549500	1.38280200
C	0.37748400	1.12580300	1.86851700
H	0.41311500	2.20767300	1.94064300
C	1.06652800	0.35380400	2.84472500
C	1.07676000	-1.01772700	2.69131000
C	1.60424700	0.78093000	0.27499700
C	1.71448800	-0.43884900	-0.01689600
C	-1.81353500	-1.50679400	0.37999400
H	-1.77829600	-2.58120700	0.22125600
C	-1.86007500	1.28894400	0.73725800
H	-1.85787300	2.37048600	0.84501000
C	-2.90164700	-0.76709100	-0.12726300

C	-2.92959300	0.66069100	0.06075300
C	1.99302900	-1.61576700	-0.79119800
C	1.98768000	2.10127400	-0.24464800
O	1.22050900	-2.10971000	-1.59278100
O	1.59075300	3.17660000	0.16196100
O	3.20656500	-2.13653600	-0.48255600
O	2.86294200	1.97039400	-1.26578300
C	3.53785100	-3.33979700	-1.19636300
H	3.55928200	-3.15819500	-2.27416300
H	2.80709000	-4.12704800	-0.99015700
H	4.52522100	-3.62978800	-0.83727000
C	3.28918500	3.21058200	-1.85748300
H	3.78160100	3.84377600	-1.11456300
H	2.43531200	3.75368900	-2.27115600
H	3.98584400	2.93098900	-2.64740800
H	1.73630100	-1.64592300	3.28125700
H	1.71797800	0.84461400	3.56054400
C	-4.03160000	1.39716200	-0.45915200
H	-4.05051700	2.47446800	-0.31689400
C	-3.97597900	-1.38726700	-0.82810200
H	-3.95006900	-2.46429500	-0.97096400
C	-5.02184000	-0.64357300	-1.31719100
H	-5.83320600	-1.12919100	-1.85118000
C	-5.04996100	0.76442200	-1.13016000
H	-5.88298400	1.33990500	-1.52300900

ν_{imag} = -423.4 cm^{-1}

Zero-point correction=	0.309938 (Hartree/Particle)
Thermal correction to Energy=	0.331057
Thermal correction to Enthalpy=	0.332001
Thermal correction to Gibbs Free Energy=	0.257365
Sum of electronic and zero-point Energies=	-1072.315521
Sum of electronic and thermal Energies=	-1072.294402
Sum of electronic and thermal Enthalpies=	-1072.293458
Sum of electronic and thermal Free Energies=	-1072.368094

	E (Thermal)	CV	S
	KCal/Mol	Cal/Mol-Kelvin	Cal/Mol-Kelvin
Total	207.741	79.410	157.085

3.2. TS 1,4-addition 1,5-dimethoxyanthracene + DMAD

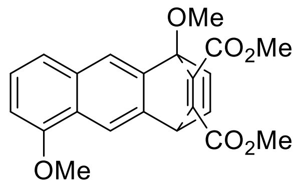

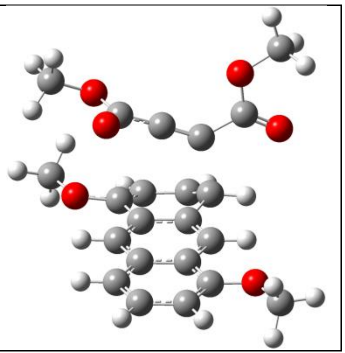

0 1

C	-0.47670400	0.20573800	1.15122600
C	-0.09056800	-1.13871600	0.85588100
C	1.23782100	-1.54338000	1.27292600
C	0.57311000	1.09940400	1.67615700
H	0.24138300	2.09090200	1.97113900
C	1.55628400	0.48342600	2.52152900
C	1.92911400	-0.81827900	2.26809300
C	1.56624700	1.44170700	0.18159600
C	1.97540400	0.45620200	-0.50750400
C	-0.96626200	-1.99085500	0.19719300
H	-0.65232400	-2.99870600	-0.04872600
C	-1.74107200	0.64388200	0.81277700
H	-2.03350300	1.66753100	1.01675600
C	-2.26497700	-1.56309900	-0.14468300
C	-2.66132100	-0.22272200	0.18406400
C	2.26063800	-0.62299300	-1.36538100
C	1.72213200	2.90797700	0.04229100
O	1.47047900	-1.09207600	-2.17386700
O	1.35774800	3.74043500	0.85281600
O	3.52607500	-1.12602400	-1.18161900
O	2.34342200	3.22565600	-1.11319800
C	3.84872800	-2.21162400	-2.06218800
H	3.82236700	-1.89039400	-3.10731800
H	3.14374100	-3.03828800	-1.93742600
H	4.85808800	-2.52201600	-1.78807000
C	2.53919600	4.63348500	-1.32437700
H	3.15784100	5.06225600	-0.53111300
H	1.58064500	5.15922400	-1.34277100
H	3.04024700	4.71573700	-2.28876800
H	2.78875900	-1.25113500	2.76376900
H	2.12474200	1.09400000	3.21530900
C	-3.98794200	0.20255200	-0.15962000

C	-3.18983800	-2.42685600	-0.80080800
H	-2.87941300	-3.43733700	-1.04854000
C	-4.44694800	-1.97926400	-1.11348700
H	-5.15109200	-2.63649300	-1.61509300
C	-4.85898400	-0.65826200	-0.79498500
H	-5.86053800	-0.34236800	-1.05890000
O	1.63303300	-2.74399500	0.82016700
C	2.93352500	-3.22477400	1.17426600
H	3.08799000	-4.11837400	0.56943100
H	2.97868300	-3.49279600	2.23585300
H	3.69440600	-2.47791100	0.93244000
O	-4.28266600	1.48808500	0.18986400
C	-5.56513100	1.99839600	-0.13885500
H	-5.73823500	1.98524100	-1.22281100
H	-5.57914800	3.02974700	0.21641000
H	-6.36406400	1.43286000	0.35845500

ν_{imag} = -396.8 cm^{-1}

Zero-point correction= 0.379029 (Hartree/Particle)
Thermal correction to Energy= 0.404401
Thermal correction to Enthalpy= 0.405345
Thermal correction to Gibbs Free Energy= 0.323173
Sum of electronic and zero-point Energies= -1301.377999
Sum of electronic and thermal Energies= -1301.352627
Sum of electronic and thermal Enthalpies= -1301.351683
Sum of electronic and thermal Free Energies= -1301.433855

	E (Thermal)	CV	S
	KCal/Mol	Cal/Mol-Kelvin	Cal/Mol-Kelvin
Total	253.765	96.392	172.945

3.3. **TS 1,4-addition** ***N***^{1},***N***^{1},***N***^{5},***N***^{5}-tetramethylanthracene-1,5-diamine + DMAD

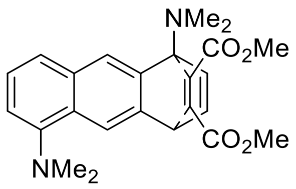

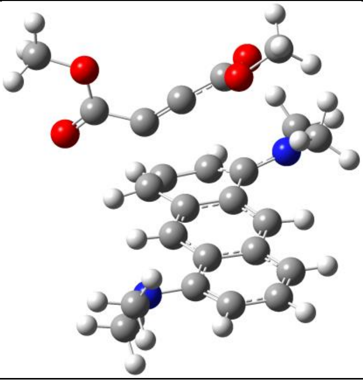

0 1

C	-0.03709600	-1.10907800	-0.93950600
C	-0.37495100	0.28458500	-0.97591900
C	0.61548300	1.23341900	-1.47178200
H	0.27419800	2.24949000	-1.63788500
C	1.29529300	-1.50171200	-1.41808800
C	1.94183200	-0.61847100	-2.32203400
H	2.81381200	-0.94407600	-2.87152500
C	1.57354200	0.71027300	-2.38220400
H	2.14601900	1.40309800	-2.99037100
C	2.23712300	0.53895000	0.69119900
C	1.85174900	1.52968100	0.01516200
C	-1.01897300	-2.01798700	-0.54861100
H	-0.82708100	-3.08319100	-0.58318500
C	-1.62543700	0.70478000	-0.54849600
H	-1.84340100	1.76651000	-0.52521000
C	-2.29836300	-1.60391000	-0.13074700
C	-2.61878400	-0.20409000	-0.13830600
C	-3.26694300	-2.54365800	0.32895200
H	-3.01370000	-3.60026200	0.33560500
C	-3.93444000	0.21588700	0.27849100
C	-4.48494200	-2.10921200	0.78220800
H	-5.21704400	-2.82162300	1.15138900
C	-4.82255600	-0.73084500	0.74987200
H	-5.81053400	-0.42906900	1.07938800
N	1.84884600	-2.70492300	-1.10645200
C	1.53753000	-3.44213600	0.12213600
H	0.95133200	-2.82572700	0.80041400
H	2.47619300	-3.68217800	0.62329500
H	0.99245500	-4.36977800	-0.09140800
C	3.11373500	-3.09729100	-1.72902900
H	3.32543600	-4.13250200	-1.45845300
H	3.93069200	-2.46030300	-1.37210500
H	3.04393300	-3.04776500	-2.81865500
N	-4.25097900	1.60097300	0.20357000
C	-5.24782800	2.08885900	1.14540800
H	-5.01389900	1.73054800	2.15073400
H	-6.28107900	1.78577900	0.89688300
H	-5.22004300	3.18339200	1.15229700
C	-4.51354300	2.08515400	-1.15568700
H	-4.48220800	3.17979700	-1.16669700
H	-5.50211200	1.76234700	-1.52632200
H	-3.75542700	1.71257900	-1.84597800
C	2.66880100	-0.63026900	1.33537100

C	2.21908100	2.95672800	-0.07124900
O	3.60171700	-1.35048600	0.97762500
O	2.01263900	3.68730900	-1.02366100
O	1.92636600	-0.88605900	2.46231700
O	2.84569700	3.36866000	1.05203500
C	2.41670000	-1.94705900	3.28737000
H	2.24305900	-2.92673700	2.83044000
H	1.85729300	-1.87459800	4.22110200
H	3.48857600	-1.83885900	3.47298900
C	3.27267400	4.74057800	1.03330300
H	3.98504400	4.91406200	0.22197400
H	3.74477500	4.91171900	2.00070800
H	2.41916200	5.41054700	0.89733900

ν_{imag} = -341.2 cm^{-1}

Zero-point correction=	0.459794 (Hartree/Particle)
Thermal correction to Energy=	0.487443
Thermal correction to Enthalpy=	0.488387
Thermal correction to Gibbs Free Energy=	0.402274
Sum of electronic and zero-point Energies=	-1340.191242
Sum of electronic and thermal Energies=	-1340.163593
Sum of electronic and thermal Enthalpies=	-1340.162649
Sum of electronic and thermal Free Energies=	-1340.248762

	E (Thermal)	CV	S
	KCal/Mol	Cal/Mol-Kelvin	Cal/Mol-Kelvin
Total	305.875	106.603	181.240

3.4. TS 1,4-addition 1,5-di(pyrrolidin-1-yl)anthracene + DMAD

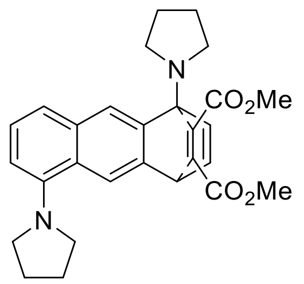

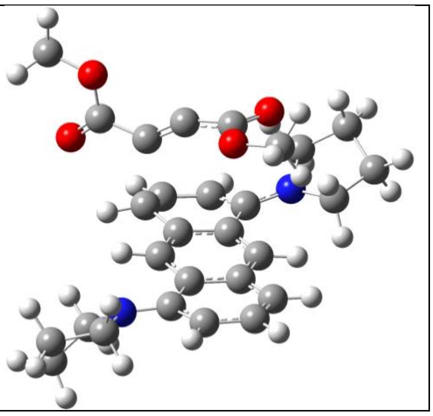

0 1

C	-3.81674600	2.41855700	-0.74256200

C	-3.43507200	1.20472600	-0.17955300
C	-2.04886600	1.04788000	0.21724300
C	-1.17454800	2.18788600	0.17113500
C	-1.64667100	3.42836800	-0.34413500
C	-2.92726000	3.51524000	-0.82378700
C	-1.47595000	-0.18041600	0.59383100
C	0.16456900	2.05303400	0.58632700
C	0.71548400	0.83792700	0.99036800
C	-0.15184800	-0.31006000	0.98505200
C	0.38323400	-1.62830000	1.31028000
C	1.18702900	-2.29399200	-0.32875800
C	1.67199100	-1.48335700	-1.17550800
C	2.11336100	0.61668200	1.39905100
H	-4.82682200	2.55775800	-1.10476700
H	-0.97195900	4.27879200	-0.38264700
H	-3.28834600	4.44585800	-1.25269100
H	-2.06628900	-1.08441800	0.54671700
H	0.75421400	2.95459700	0.57208900
C	3.09867900	2.77460100	0.48171600
C	4.53703800	0.93008900	1.12908600
C	4.56788600	3.19858200	0.37596400
H	2.52383700	3.46891200	1.10007400
H	2.64192900	2.69945900	-0.50859700
C	5.29689800	1.86515800	0.18791400
H	4.54541200	-0.10427700	0.78014600
H	4.94174500	0.96884300	2.15018700
H	4.72612700	3.90299100	-0.44453700
H	4.88857300	3.68428300	1.30507300
H	5.17250100	1.50798300	-0.83682100
H	6.36243800	1.91567000	0.42671800
C	-4.41271300	-0.69011700	1.17189500
C	-5.68185600	0.27820600	-0.63653300
C	-5.91056900	-0.95914900	1.37191000
H	-3.94628200	-0.18713300	2.02472500
H	-3.88877500	-1.64210700	1.02168100
C	-6.44939000	-0.91321800	-0.06393400
H	-5.62036800	0.28580700	-1.73017300
H	-6.17934800	1.21157600	-0.32291900
H	-6.09686700	-1.90683100	1.88449300
H	-6.36614400	-0.15790600	1.96542800
H	-6.18850300	-1.83207100	-0.60127300
H	-7.53261200	-0.77807900	-0.12201100
N	3.14649100	1.43908300	1.10255500
N	-4.34372100	0.15276300	-0.04692600

C	1.54901000	-1.64757800	2.11985800
C	2.40493100	-0.56786300	2.12754700
H	-0.34225800	-2.41823900	1.47599600
H	1.82732100	-2.57329100	2.61192900
H	3.35692200	-0.65138500	2.63550500
C	2.00331600	-0.20221500	-1.66650800
C	1.12073100	-3.76251000	-0.21856300
O	3.09723700	0.36023100	-1.58177800
O	0.93660800	0.34329200	-2.33237100
O	0.82496900	-4.38447100	0.78787700
O	1.44531500	-4.36501700	-1.38365000
C	1.42970600	-5.80029200	-1.34177800
H	0.43444200	-6.17111600	-1.08089700
H	1.70847800	-6.12387900	-2.34468900
H	2.14483100	-6.17561900	-0.60430600
C	1.16079800	1.62877800	-2.91231600
H	0.29706500	1.81893800	-3.55074800
H	1.21242800	2.40252200	-2.13874000
H	2.08486100	1.64728200	-3.49642200

ν_{imag} = -347.8 cm^{-1}

Zero-point correction= 0.531850 (Hartree/Particle)
Thermal correction to Energy= 0.562416
Thermal correction to Enthalpy= 0.563361
Thermal correction to Gibbs Free Energy= 0.469316
Sum of electronic and zero-point Energies= -1494.934758
Sum of electronic and thermal Energies= -1494.904192
Sum of electronic and thermal Enthalpies= -1494.903248
Sum of electronic and thermal Free Energies= -1494.997293

	E (Thermal)	CV	S
	KCal/Mol	Cal/Mol-Kelvin	Cal/Mol-Kelvin
Total	352.922	117.830	197.934

3.5. TS 1,4-addition 1-Pyrrolidin-1-ylanthracen + DMAD

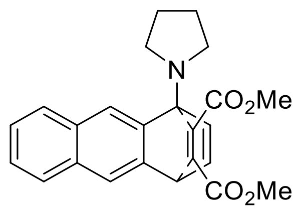

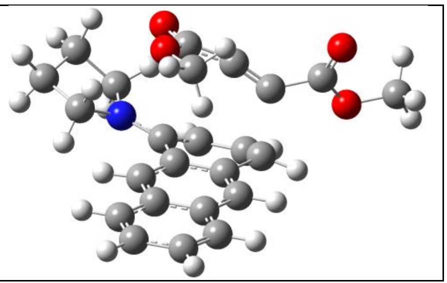

0 1

C	-4.19061700	-3.49391400	0.12362300
C	-2.85454500	-3.51871800	-0.19350900
C	-2.14599200	-2.30911000	-0.44429500
C	-2.84815700	-1.06204500	-0.36056100
C	-4.23596200	-1.07489600	-0.03463800
C	-4.89000400	-2.25831800	0.20308200
C	-0.77768000	-2.27745500	-0.77251700
C	-2.15249700	0.13964200	-0.59735100
C	-0.79358700	0.17167200	-0.90984200
C	-0.10240300	-1.09196900	-1.01561700
C	1.32793300	-1.10869000	-1.30628900
C	1.82133000	0.00635800	-2.03928800
C	1.18543700	1.22145400	-1.96379500
C	-0.01236200	1.39122200	-1.20554600
H	-0.23096400	-3.21497700	-0.83798100
H	-4.72172200	-4.42141300	0.31586100
H	-2.31739800	-4.46139200	-0.25545500
H	-4.76700100	-0.12847000	0.02736200
H	-5.94630200	-2.25681200	0.45452500
H	-2.73203900	1.04599900	-0.54270500
H	2.77683500	-0.07565500	-2.54666200
H	1.64251400	2.08509200	-2.42698900
C	0.59714700	3.76877600	-0.96691800
C	-1.54536300	3.05695100	-0.05586300
C	0.11812700	4.80228100	0.05334100
H	0.57371000	4.16105300	-1.99325000
H	1.60131400	3.41411300	-0.73328100
C	-1.39476900	4.57505500	0.10466400
H	-1.54461000	2.53106800	0.90323300
H	-2.46554200	2.82838000	-0.59676200
H	0.56640400	4.56950700	1.01989800
H	0.38867800	5.82072500	-0.23777600
H	-1.85305300	4.92589800	1.03245000
H	-1.88946300	5.08716900	-0.72894500
N	-0.36035800	2.64196900	-0.83926200
C	1.66219900	0.11914700	1.17482200
C	2.15460200	-0.77467200	0.42637900
H	1.74590800	-2.07994000	-1.54821600
C	0.92385800	1.19841300	1.68748800
C	3.31749600	-1.67141900	0.58676800
O	3.72302100	-2.09848800	1.64647100
O	3.91404100	-1.95889100	-0.60147900
O	1.23066400	2.38210200	1.56044500

O	-0.21649600	0.90593900	2.39846200
C	-0.53212200	-0.47042500	2.64610900
H	-1.41970600	-0.46076600	3.28045100
H	-0.75374000	-1.00623400	1.71939200
H	0.28997600	-0.97540800	3.16438000
C	5.07858200	-2.79388700	-0.50110200
H	5.84661600	-2.31168300	0.10935500
H	4.82684300	-3.75600200	-0.04682900
H	5.43151400	-2.93205000	-1.52326600

ν_{imag} = -360.5 cm^{-1}

Zero-point correction= 0.421943 (Hartree/Particle)
Thermal correction to Energy= 0.447428
Thermal correction to Enthalpy= 0.448372
Thermal correction to Gibbs Free Energy= 0.366595
Sum of electronic and zero-point Energies= -1283.633109
Sum of electronic and thermal Energies= -1283.607624
Sum of electronic and thermal Enthalpies= -1283.606680
Sum of electronic and thermal Free Energies= -1283.688457

	E (Thermal)	CV	S
	KCal/Mol	Cal/Mol-Kelvin	Cal/Mol-Kelvin
Total	280.765	98.521	172.114

3.6. TS endo 1,4-addition anthracene + maleic anhydride

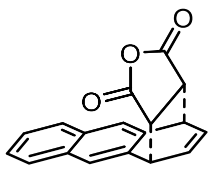

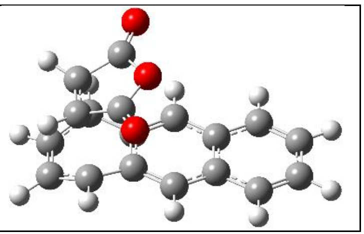

0 1

C	4.40356200	-0.71068600	-0.15935200
C	3.25419500	-1.40554500	0.12602200
C	2.04214300	-0.71981300	0.42508300
C	2.04218500	0.72007700	0.42410600
C	3.25421000	1.40544300	0.12412600
C	4.40357300	0.71022600	-0.16032400
C	0.84406500	-1.40955600	0.70681700
C	0.84418300	1.41022100	0.70495600
C	-0.31411600	0.71583200	1.01453600
C	-0.31423400	-0.71464900	1.01538300

C	-1.61148300	-1.34287000	1.23471500
H	-1.65587600	-2.42476100	1.14139500
C	-2.52500900	-0.68382500	2.11595600
C	-2.52482000	0.68676300	2.11515900
C	-1.61115600	1.34450800	1.23316100
H	5.32003800	-1.24559000	-0.38964200
H	3.24994400	-2.49207700	0.12267300
H	3.24992600	2.49197300	0.11935700
H	5.32005000	1.24482300	-0.39132000
H	0.83290600	2.49598800	0.67101000
H	-3.29341600	-1.25324800	2.62924600
H	-3.29313800	1.25699800	2.62768200
H	-1.65546500	2.42625000	1.13819200
H	0.83271200	-2.49535900	0.67420900
C	-2.54716800	-0.70990900	-0.58150100
C	-2.54701800	0.70974600	-0.58265300
C	-1.52206600	-1.14025400	-1.56607700
C	-1.52147400	1.13842900	-1.56751800
O	-1.21536500	-2.24341100	-1.92799000
O	-1.21393100	2.24100700	-1.93048200
O	-0.86469200	-0.00136700	-2.03676000
H	-3.42680400	-1.32592900	-0.45726800
H	-3.42641300	1.32626800	-0.45924900

ν_{imag} = -500.3 cm^{-1}

Zero-point correction= 0.251848 (Hartree/Particle)
Thermal correction to Energy= 0.266662
Thermal correction to Enthalpy= 0.267606
Thermal correction to Gibbs Free Energy= 0.210070
Sum of electronic and zero-point Energies= -918.581773
Sum of electronic and thermal Energies= -918.566959
Sum of electronic and thermal Enthalpies= -918.566015
Sum of electronic and thermal Free Energies= -918.623551

	E (Thermal)	CV	S
	KCal/Mol	Cal/Mol-Kelvin	Cal/Mol-Kelvin
Total	167.333	61.817	121.096

3.7. TS endo 1,4-addition 1,5-dimethoxyanthracene + maleic anhydride

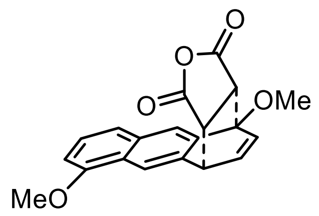

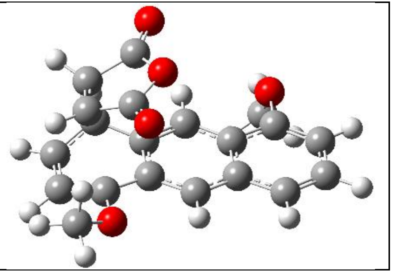

0 1

C	-4.20293500	1.11733900	0.48904200
C	-3.44735300	0.04588500	0.07685200
C	-2.05593300	0.19560100	-0.21340500
C	-1.46899000	1.50261500	-0.08666700
C	-2.28294200	2.59206200	0.33792100
C	-3.61186900	2.40001900	0.62499200
C	-1.25111600	-0.90126700	-0.58603700
C	-0.09979600	1.66578200	-0.37895900
C	0.66073500	0.58638600	-0.80409800
C	0.08540800	-0.71864200	-0.89125300
C	1.02064600	-1.82150500	-1.15631300
H	0.56859800	-2.80855500	-1.22108800
C	2.09394900	-1.51886300	-2.07070300
C	2.65124500	-0.27162600	-2.01164700
C	2.07917200	0.68664700	-1.11423000
H	-5.25283900	0.96057000	0.71383600
H	-1.82982200	3.57388900	0.43822700
H	-4.22431800	3.23249200	0.95772500
H	0.36506000	2.63883200	-0.27126300
H	2.55015800	-2.30748100	-2.66033800
H	3.55010500	-0.01834900	-2.56285200
H	-1.68690400	-1.89370700	-0.59283300
C	1.99783700	-1.90994900	0.51428300
C	2.75839700	-0.72548800	0.72391300
O	2.60621400	1.91075800	-0.91729500
O	-4.01962900	-1.20443100	-0.03583300
C	-4.54768300	-1.48552400	-1.33347800
H	-4.95758700	-2.49662400	-1.29121500
H	-5.34466300	-0.77882000	-1.59945700
H	-3.76466200	-1.44132400	-2.10219600
C	4.00187400	2.04863100	-0.63085600
H	4.23569100	3.10283300	-0.78235200
H	4.17096300	1.77844900	0.41541300
H	4.62871600	1.44260000	-1.29231000

C	0.91033800	-1.85657400	1.55531400
C	2.11607400	0.06861000	1.75405100
O	2.40760300	1.13905000	2.22818400
O	0.09517700	-2.69333600	1.83357900
O	0.94572400	-0.61583700	2.16459200
H	2.46317600	-2.87952000	0.37213300
H	3.80525700	-0.59575500	0.50043200

ν_{imag} = -454.5 cm^{-1}

Zero-point correction= 0.316477 (Hartree/Particle)
Thermal correction to Energy= 0.336865
Thermal correction to Enthalpy= 0.337810
Thermal correction to Gibbs Free Energy= 0.267899
Sum of electronic and zero-point Energies= -1147.575129
Sum of electronic and thermal Energies= -1147.554741
Sum of electronic and thermal Enthalpies= -1147.553797
Sum of electronic and thermal Free Energies= -1147.623707

	E (Thermal)	CV	S
	KCal/Mol	Cal/Mol-Kelvin	Cal/Mol-Kelvin
Total	211.386	80.498	147.139

3.8. TS endo 1,4-addition *N*[1],*N*[1],*N*[5],*N*[5]-tetramethylanthracene-1,5-diamine + maleic anhydride

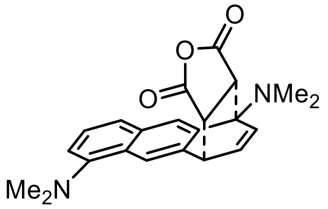

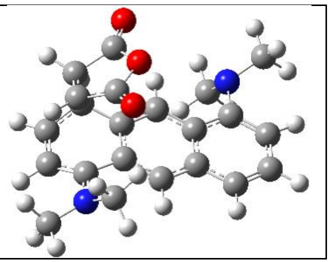

0 1

C	-3.93573200	1.63649600	0.51733600
C	-3.31269600	0.53315500	-0.03574300
C	-1.88757700	0.56818600	-0.24308600
C	-1.16769400	1.77495700	0.04790100
C	-1.86456000	2.89726600	0.58022600
C	-3.20982000	2.81272600	0.83214400
C	-1.16428800	-0.55391500	-0.70158400
C	0.22531400	1.81513300	-0.16990400
C	0.92340000	0.70330500	-0.62793400
C	0.19893400	-0.49497900	-0.92083700
C	0.97208200	-1.66096100	-1.39403600

H	0.35795700	-2.49571100	-1.72739300
C	2.09194500	-1.30918500	-2.23831800
C	2.80686200	-0.17885700	-1.96013700
C	2.36167100	0.71275000	-0.92491400
H	-5.00521900	1.62076600	0.69469700
H	-1.31009900	3.80373500	0.80576400
H	-3.73966100	3.65958000	1.25852300
H	0.74138600	2.75142100	0.00839400
H	2.47606700	-2.03255400	-2.95125500
H	3.75382800	-0.00847100	-2.45549500
H	-1.68688600	-1.49168600	-0.84872100
C	1.79416700	-2.35319300	0.08199300
C	2.62387400	-1.41190400	0.76967000
N	3.17115000	1.71128400	-0.46491300
N	-4.01261500	-0.65139300	-0.38792900
C	-4.19547400	-0.84520900	-1.82891400
H	-3.26585400	-0.64108600	-2.36223500
H	-4.48423600	-1.88328300	-2.02411200
H	-4.97808300	-0.18384000	-2.24033600
C	-5.23480200	-0.92963300	0.35197400
H	-6.08597800	-0.28769000	0.06100200
H	-5.52786300	-1.96825900	0.16849400
H	-5.05533000	-0.80656000	1.42244500
C	3.09857400	2.17685500	0.92745800
H	2.20689800	1.81210700	1.42559400
H	3.95252200	1.77665700	1.48619300
H	3.12982800	3.27097600	0.95938900
C	4.49061900	1.90939400	-1.05375400
H	5.18537100	1.09685300	-0.79455500
H	4.42749800	1.99355300	-2.14073300
H	4.89958800	2.84522900	-0.66844000
C	0.61867800	-2.57154200	1.01472900
C	1.94148400	-0.95616300	1.94318300
O	-0.27793200	-3.36769800	0.89747200
O	2.21846200	-0.15664400	2.80973300
O	0.68010300	-1.64996400	2.01818600
H	2.20742000	-3.27016100	-0.33219700
H	3.67626600	-1.23445500	0.61703900

ν_{imag} = -288.2 cm^{-1}

Zero-point correction= 0.398999 (Hartree/Particle)
Thermal correction to Energy= 0.422081
Thermal correction to Enthalpy= 0.423025

Thermal correction to Gibbs Free Energy= 0.347827
Sum of electronic and zero-point Energies= -1186.405997
Sum of electronic and thermal Energies= -1186.382915
Sum of electronic and thermal Enthalpies= -1186.381971
Sum of electronic and thermal Free Energies= -1186.457169

	E (Thermal)	CV	S
	KCal/Mol	Cal/Mol-Kelvin	Cal/Mol-Kelvin
Total	264.860	92.232	158.267

3.9. TS endo 1,4-addition 1,5-di(pyrrolidin-1-yl)anthracene + maleic anhydride

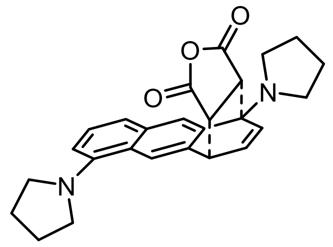

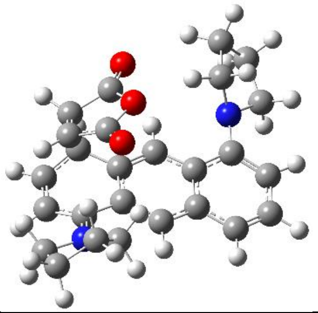

0 1

C	3.43776400	2.64435900	-0.17396000
C	3.06083700	1.34501200	0.11986200
C	1.67490300	1.07697000	0.41355600
C	0.70503800	2.11976700	0.24358800
C	1.14099300	3.43235000	-0.09767500
C	2.47868800	3.68438900	-0.26781800
C	1.24402800	-0.16376100	0.92309200
C	-0.65863900	1.82231500	0.44155300
C	-1.08054900	0.55726500	0.84009600
C	-0.09284100	-0.42844100	1.15785900
C	-0.54988300	-1.73682600	1.66490700
H	0.25161900	-2.36374600	2.05129400
C	-1.75424100	-1.66677400	2.46167900
C	-2.72573700	-0.77121000	2.12120000
C	-2.48290100	0.19292100	1.07556600
H	4.47835400	2.87536800	-0.37023800
H	0.40420900	4.22271100	-0.20955100
H	2.81640700	4.68711000	-0.51377800
H	-1.37308400	2.62550700	0.31076100
H	-1.95742400	-2.43329000	3.20337700
H	-3.69521500	-0.80669400	2.60206200
H	1.98375800	-0.92083400	1.15279700
C	-1.09696000	-2.68488400	0.17860500
C	-2.19332500	-2.08335100	-0.51174500

C	0.07638200	-2.50024400	-0.76006000
C	-1.71855400	-1.41821200	-1.67486100
O	1.19268900	-2.94911500	-0.64384900
O	-2.24834300	-0.74740800	-2.53708000
O	-0.28761500	-1.65522100	-1.76084600
N	-3.51075300	0.85081700	0.50426400
C	-4.90575400	0.42365500	0.74518000
C	-3.42548200	1.62878400	-0.75940500
C	-5.72281700	1.12025600	-0.34941100
H	-4.96516800	-0.66841900	0.64538800
C	-4.71145400	1.25578300	-1.49440200
H	-3.40130800	2.70203400	-0.53070900
H	-6.04869700	2.10963000	-0.00862800
H	-4.99364100	2.00601500	-2.23706300
N	3.96739100	0.27136400	0.17290600
C	3.86334100	-0.77173300	-0.87437200
C	5.39914900	0.54242900	0.27850900
C	5.00599100	-1.73626800	-0.51452200
H	2.88501000	-1.25076800	-0.88932500
C	6.01441900	-0.86856100	0.29417800
H	5.77739100	1.10375100	-0.59630200
H	4.62621600	-2.56110400	0.09409800
H	6.09404000	-1.23230200	1.32234700
H	4.03336500	-0.31756300	-1.86611200
H	5.45225700	-2.17118500	-1.41265300
H	7.01973300	-0.87217900	-0.13606900
H	5.61332400	1.13289800	1.17443800
H	-2.53254000	1.35252400	-1.31489700
H	-4.55187600	0.30148800	-2.00559600
H	-6.61389200	0.54634300	-0.61509000
H	-5.22759600	0.68958600	1.75739100
H	-3.24456700	-2.21411800	-0.31207000
H	-1.17536800	-3.68001800	0.61094000

ν_{imag} = -296.1 cm^{-1}

Zero-point correction= 0.473307 (Hartree/Particle)
Thermal correction to Energy= 0.497882
Thermal correction to Enthalpy= 0.498826
Thermal correction to Gibbs Free Energy= 0.419784
Sum of electronic and zero-point Energies= -1341.204884
Sum of electronic and thermal Energies= -1341.180309
Sum of electronic and thermal Enthalpies= -1341.179365
Sum of electronic and thermal Free Energies= -1341.258407

	E (Thermal)	CV	S
	KCal/Mol	Cal/Mol-Kelvin	Cal/Mol-Kelvin
Total	312.426	100.483	166.358

3.10. TS endo 1,4-addition 1-pyrrolidin-1-ylanthracene + maleic anhydride

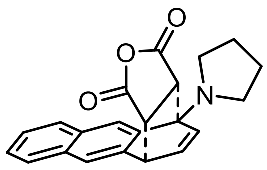

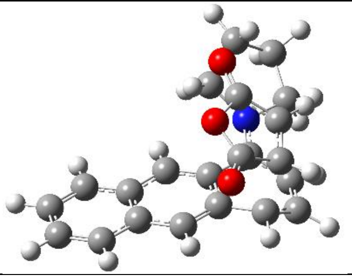

0 1

C	-1.79037200	0.56945300	-2.10788600
C	-1.35001100	-0.36130700	-1.09916000
C	0.09240900	-0.37721900	-0.81927700
C	0.81367800	0.84018500	-1.05730800
C	0.05411100	2.02063000	-1.50740400
C	-1.06645800	1.70032600	-2.35795800
C	0.79907500	-1.51498100	-0.44159000
C	2.17487800	0.89387200	-0.82178400
C	2.89588700	-0.24273000	-0.40007800
C	2.19166300	-1.48107100	-0.22757900
C	2.92258600	-2.64088800	0.15994100
H	2.38724100	-3.57826200	0.28676500
C	4.27650800	-2.57492800	0.37989100
C	4.97069500	-1.34546400	0.22065500
C	4.29812500	-0.20995600	-0.16163600
H	-2.72119400	0.39218500	-2.63151400
H	0.67715700	2.85814000	-1.81466100
H	-1.43472900	2.43532300	-3.06715600
H	2.70056500	1.83379100	-0.95991000
H	4.82358600	-3.46316600	0.68120500
H	6.04028300	-1.30827800	0.40428100
H	0.29614200	-2.46751600	-0.33516200
C	-1.70505800	1.79912600	0.60880600
C	-0.77251600	2.69973200	0.01867500
N	-2.19789800	-1.29340900	-0.61647800
C	-3.64370900	-1.21878200	-0.91752900
C	-1.97455900	-2.09142100	0.61757000
C	-4.30407800	-2.16025200	0.09643700
H	-3.98429200	-0.18474500	-0.77278400
C	-3.34529800	-2.09725600	1.29135100

H	-1.66633300	-3.10989900	0.34761300
H	-5.32374100	-1.84707800	0.33342400
H	-3.45844000	-1.16269100	1.84908700
H	-3.84219100	-1.49877700	-1.95726200
H	-4.34948900	-3.17943100	-0.30408100
H	-3.45934700	-2.93319000	1.98584300
H	-1.21060100	-1.62798500	1.23686600
H	4.82582100	0.73180800	-0.28344300
C	0.37559700	2.74911400	1.00522200
C	-1.12283100	1.20192600	1.76488900
O	0.18972400	1.77163000	1.94119400
O	1.34385700	3.46633500	0.98105000
O	-1.51226100	0.37010300	2.55963400
H	-1.07903400	3.66824800	-0.36903700
H	-2.74860400	1.68218400	0.36475700

ν_{imag} = -327.9 cm^{-1}

Zero-point correction= 0.362983 (Hartree/Particle)
Thermal correction to Energy= 0.382589
Thermal correction to Enthalpy= 0.383533
Thermal correction to Gibbs Free Energy= 0.315774
Sum of electronic and zero-point Energies= -1129.900973
Sum of electronic and thermal Energies= -1129.881367
Sum of electronic and thermal Enthalpies= -1129.880423
Sum of electronic and thermal Free Energies= -1129.948182

	E (Thermal)	CV	S
	KCal/Mol	Cal/Mol-Kelvin	Cal/Mol-Kelvin
Total	240.078	81.239	142.611

3.11. TS exo 1,4-addition anthracene + maleic anhydride

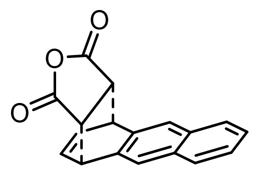

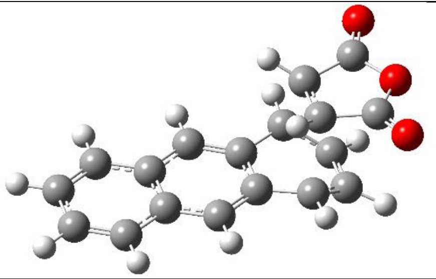

0 1

C	0.55753000	5.12562900	0.71013800
C	0.27037500	3.97588800	1.40506300

C	-0.02952400	2.76417600	0.71986900
C	-0.02952400	2.76417600	-0.71986900
C	0.27037500	3.97588800	-1.40506300
C	0.55753000	5.12562900	-0.71013800
C	-0.31913500	1.56502300	1.40860500
C	-0.31913500	1.56502300	-1.40860500
C	-0.63314700	0.40886100	-0.71535400
C	-0.63314700	0.40886100	0.71535400
C	-0.88909700	-0.88485700	1.34333900
H	-0.80147200	-0.94540200	2.42488800
C	-1.80027000	-1.76713200	0.68575900
C	-1.80027000	-1.76713200	-0.68575900
C	-0.88909700	-0.88485700	-1.34333900
H	0.78587800	6.04243200	1.24520900
H	0.26841200	3.97399800	2.49178700
H	0.26841200	3.97399800	-2.49178700
H	0.78587800	6.04243200	-1.24520900
H	-0.30522400	1.55981500	-2.49577900
H	-2.32664100	-2.52598300	1.25440900
H	-2.32664100	-2.52598300	-1.25440900
H	-0.80147200	-0.94540200	-2.42488800
H	-0.30522400	1.55981500	2.49577900
C	0.95055600	-1.74811800	0.70913200
C	0.95055600	-1.74811800	-0.70913200
C	0.73705300	-3.15130700	1.14216000
C	0.73705300	-3.15130700	-1.14216000
O	0.48202900	-3.91810600	0.00000000
O	0.72827500	-3.63288300	-2.24218300
O	0.72827500	-3.63288300	2.24218300
H	1.55868700	-1.09835800	-1.32323700
H	1.55868700	-1.09835800	1.32323700

ν_{imag} = -507.9cm^{-1}

Zero-point correction= 0.251861 (Hartree/Particle)
Thermal correction to Energy= 0.266717
Thermal correction to Enthalpy= 0.267661
Thermal correction to Gibbs Free Energy= 0.209754
Sum of electronic and zero-point Energies= -918.582161
Sum of electronic and thermal Energies= -918.567305
Sum of electronic and thermal Enthalpies= -918.566361
Sum of electronic and thermal Free Energies= -918.624268

E (Thermal) CV S

	KCal/Mol	Cal/Mol-Kelvin	Cal/Mol-Kelvin
Total	167.367	61.780	121.875

3.12. TS exo 1,4-addition 1,5-dimethoxyanthracene + maleic anhydride

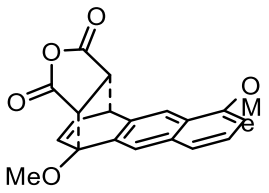

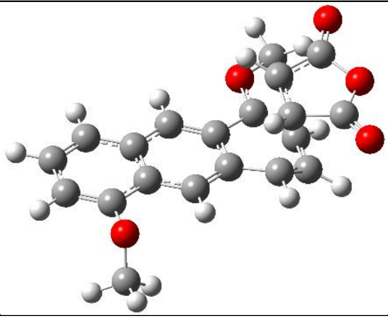

0 1

C	4.90275900	0.41379200	-0.71704600
C	3.93082700	-0.49136600	-0.35900600
C	2.59379900	-0.06725900	-0.09262700
C	2.28798500	1.33333100	-0.19613600
C	3.31919700	2.24440600	-0.56227300
C	4.59109200	1.79290100	-0.82092500
C	1.57324300	-0.98333900	0.25031700
C	0.96895900	1.76321100	0.06874700
C	-0.00824900	0.85242300	0.43650500
C	0.29598900	-0.54111500	0.52773200
C	-0.84456200	-1.43761800	0.80136200
H	-0.59567400	-2.48971800	0.92411000
C	-1.82671400	-0.90919400	1.72215600
C	-2.14007400	0.41912200	1.63577800
C	-1.38501300	1.23798400	0.74013700
H	5.90468100	0.05193000	-0.92372900
H	3.08062100	3.30132200	-0.63789700
H	5.37076400	2.49218100	-1.10697900
H	0.72009400	2.81590500	-0.00960000
H	-2.43332800	-1.58598200	2.31340400
H	-2.99096000	0.83082700	2.16390900
H	1.81015100	-2.04160100	0.28161700
C	-1.70876600	-1.39450700	-0.83970300
C	-2.08041400	-0.06671100	-1.22010600
C	-3.01137300	-2.10238500	-0.56297300
C	-3.51618800	0.06475900	-1.09222700
O	-3.19841400	-3.26101200	-0.30187600
O	-4.27604000	0.97715300	-1.31531800
O	-4.02246400	-1.16799800	-0.58295900
O	-1.67178600	2.51972700	0.48193900
O	4.22457600	-1.83730100	-0.28011500

C	4.73510000	-2.24720000	0.99142200
H	4.92023800	-3.32088200	0.92183000
H	5.67329000	-1.72950600	1.22881700
H	4.00996600	-2.05249500	1.79263500
C	-2.95218700	3.05281800	0.86410700
H	-3.01689200	3.15266100	1.95334800
H	-2.99851500	4.04071500	0.40682900
H	-3.76098200	2.43235400	0.47653400
H	-0.98172200	-1.95995500	-1.41571200
H	-1.51116700	0.61338300	-1.83392800

ν_{imag} = -401.0 cm^{-1}

Zero-point correction= 0.316932 (Hartree/Particle)
Thermal correction to Energy= 0.337247
Thermal correction to Enthalpy= 0.338191
Thermal correction to Gibbs Free Energy= 0.268032
Sum of electronic and zero-point Energies= -1147.580262
Sum of electronic and thermal Energies= -1147.559947
Sum of electronic and thermal Enthalpies= -1147.559003
Sum of electronic and thermal Free Energies= -1147.629162

	E (Thermal)	CV	S
	KCal/Mol	Cal/Mol-Kelvin	Cal/Mol-Kelvin
Total	211.625	80.283	147.662

3.13. TS exo 1,4-addition N^1,N^1,N^5,N^5-tetramethylanthracene-1,5-diamine + maleic anhydride

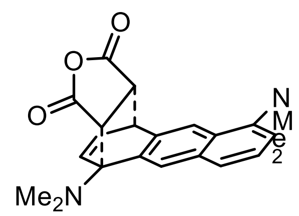

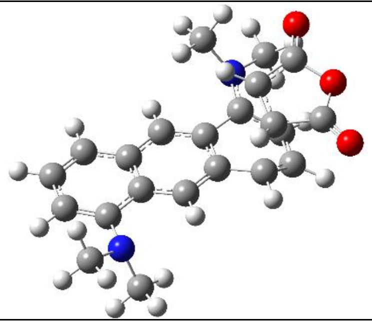

0 1

C	4.70923700	0.97899700	-0.50228800
C	3.86283800	-0.06880000	-0.18997700
C	2.46907600	0.20580000	0.04608600
C	2.01295200	1.56649100	0.03441000
C	2.93417100	2.61272400	-0.25499500

C	4.24256000	2.31657200	-0.54210100
C	1.52335900	-0.81941900	0.27593900
C	0.64808900	1.83726600	0.27901200
C	-0.26818600	0.81761800	0.49739700
C	0.19519900	-0.53721600	0.52679100
C	-0.80957300	-1.58613800	0.78431700
H	-0.40769900	-2.58593100	0.93651200
C	-1.84411100	-1.18269800	1.71064200
C	-2.31972300	0.09710000	1.65998300
C	-1.69311800	1.05218900	0.79049600
H	5.75846700	0.78704600	-0.69686000
H	2.57931000	3.63941400	-0.26633300
H	4.94255700	3.11157200	-0.78190700
H	0.32906600	2.87335700	0.31527600
H	-2.37473300	-1.93890500	2.27881800
H	-3.22940800	0.35309400	2.18587000
H	1.85411100	-1.85171900	0.24487100
C	-1.67950300	-1.73725700	-0.83881500
C	-2.11887900	-0.47911700	-1.34372600
C	-2.95666200	-2.47249800	-0.49978600
C	-3.52956900	-0.35510800	-1.15823200
O	-3.09426300	-3.61738400	-0.15311900
O	-4.31811900	0.54313400	-1.37257000
O	-4.00025800	-1.58427400	-0.57955700
N	-2.29263100	2.24020400	0.49925300
N	4.30230700	-1.41980400	-0.11117400
C	4.42769000	-1.92974900	1.25901900
H	3.54739900	-1.66491800	1.84664700
H	4.51245500	-3.02123600	1.23640100
H	5.31622700	-1.52278700	1.77175100
C	5.46347000	-1.77019900	-0.91770800
H	6.41664100	-1.38126700	-0.51699400
H	5.54636300	-2.86100400	-0.95998300
H	5.33555000	-1.39374400	-1.93538500
C	-2.09414500	2.88245800	-0.80803100
H	-1.19016800	2.51506400	-1.28585900
H	-2.94926600	2.64015500	-1.44835100
H	-2.01233100	3.96689700	-0.68505000
C	-3.59313700	2.56350900	1.09750800
H	-3.53768400	2.49847900	2.18669700
H	-3.83441200	3.59605100	0.83838600
H	-4.38573300	1.91232100	0.71726300
H	-0.92880800	-2.32347700	-1.36328400
H	-1.54672000	0.22178000	-1.92871800

ν_{imag} = -327.3 cm^{-1}

Zero-point correction= 0.399529 (Hartree/Particle)
Thermal correction to Energy= 0.422403
Thermal correction to Enthalpy= 0.423347
Thermal correction to Gibbs Free Energy= 0.349089
Sum of electronic and zero-point Energies= -1186.410182
Sum of electronic and thermal Energies= -1186.387309
Sum of electronic and thermal Enthalpies= -1186.386365
Sum of electronic and thermal Free Energies= -1186.460622

	E (Thermal)	CV	S
	KCal/Mol	Cal/Mol-Kelvin	Cal/Mol-Kelvin
Total	265.062	92.064	156.288

3.14. TS exo 1,4-addition 1,5-di(pyrrolidin-1-yl)anthracene + maleic anhydride

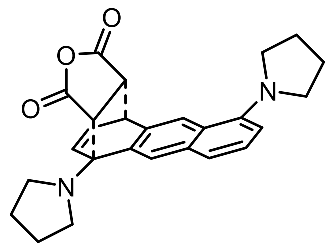

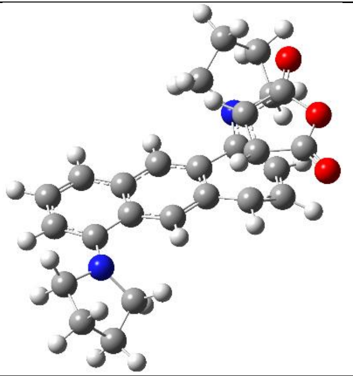

0 1

C	-4.13450800	-2.13975000	-0.65049500
C	-3.64234600	-0.88737600	-0.29280400
C	-2.22864100	-0.77362300	0.00038500
C	-1.42881900	-1.96777900	0.06274600
C	-2.00887800	-3.23104300	-0.23592700
C	-3.32370100	-3.29589700	-0.62053200
C	-1.56799300	0.46205700	0.18879700
C	-0.05418400	-1.86665700	0.37289800
C	0.57132000	-0.64344500	0.57044600
C	-0.22274200	0.54626300	0.49250000
C	0.45812000	1.83856600	0.68890100
H	-0.19933600	2.70189400	0.77227300
C	1.53476700	1.78729300	1.65100300
C	2.31731500	0.67126100	1.72257900
C	1.98983700	-0.48535800	0.93354500

H	-5.17308900	-2.25591400	-0.93090500
H	-1.39106200	-4.12300100	-0.19085600
H	-3.76872000	-4.25050300	-0.88634000
H	0.50196400	-2.79090500	0.46253100
H	1.82979100	2.69339700	2.16896900
H	3.22560700	0.68429500	2.30990500
H	-2.11787400	1.38637300	0.08083600
C	1.28967500	2.12706100	-0.94461400
C	2.06179100	1.01416800	-1.38376400
N	2.90887300	-1.45206300	0.73595800
C	4.30452400	-1.30032500	1.22324100
C	2.85235000	-2.44736300	-0.35639800
C	5.07285100	-2.42507400	0.52094200
H	4.68926700	-0.32384100	0.91967000
C	4.31061100	-2.58465400	-0.80054900
H	2.46827000	-3.40520000	0.01675800
H	5.02429200	-3.34940600	1.10850600
H	4.49963900	-3.53825400	-1.30051700
N	-4.47383100	0.23628100	-0.26734700
C	-5.84048200	0.13299500	-0.79515300
C	-4.44643600	1.21388100	0.84999000
C	-6.50330500	1.43701400	-0.35344200
H	-5.82174600	-0.01500400	-1.88019000
C	-5.91468800	1.61345400	1.05268500
H	-3.98999000	0.77674800	1.74381900
H	-6.19746900	2.26373600	-1.00457900
H	-6.01753700	2.62629100	1.45128100
H	-6.38849600	-0.71477900	-0.35094600
H	-7.59453400	1.37554800	-0.36711300
H	-6.40514200	0.92404200	1.74961700
H	-3.86250900	2.10278800	0.58046000
H	2.18663500	-2.09359900	-1.14592600
H	4.55869100	-1.75994600	-1.47299500
H	6.12435300	-2.16475500	0.38185600
H	4.33269500	-1.37972000	2.31505400
C	3.44437200	1.28297300	-1.18115100
C	2.31198700	3.19728000	-0.63598400
O	4.45851500	0.63359600	-1.35234700
O	2.12387000	4.35287600	-0.34972400
O	3.55551100	2.62409600	-0.65926900
H	0.41444600	2.46135100	-1.49656800
H	1.70661400	0.13875100	-1.90322300

ν_{imag} = -322.6 cm^{-1}

Zero-point correction= 0.473456 (Hartree/Particle)
Thermal correction to Energy= 0.498107
Thermal correction to Enthalpy= 0.499051
Thermal correction to Gibbs Free Energy= 0.419085
Sum of electronic and zero-point Energies= -1341.202848
Sum of electronic and thermal Energies= -1341.178198
Sum of electronic and thermal Enthalpies= -1341.177254
Sum of electronic and thermal Free Energies= -1341.257219

	E (Thermal)	CV	S
	KCal/Mol	Cal/Mol-Kelvin	Cal/Mol-Kelvin
Total	312.567	100.592	168.302

3.15. TS exo 1,4-addition 1-pyrrolidin-1-ylanthracene + maleic anhydride

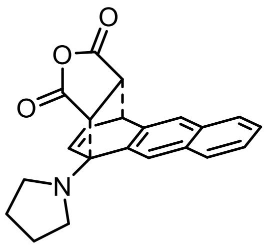

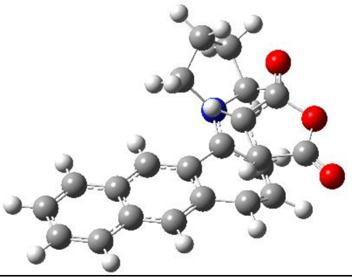

0 1

C	-1.41212500	-0.26212100	1.74629300
C	-0.66882000	0.62888300	0.89804500
C	0.70058400	0.20758500	0.55321500
C	0.97128600	-1.20385800	0.54748400
C	-0.15534000	-2.11610000	0.81312800
C	-1.11959000	-1.59526100	1.75440800
C	1.74909100	1.09419600	0.34257600
C	2.23996900	-1.66027900	0.25245100
C	3.30901700	-0.77028300	0.00131900
C	3.05698700	0.64084100	0.06454800
C	4.13674700	1.54283300	-0.15433100
H	3.94442000	2.61129400	-0.10073400
C	5.39802400	1.07419800	-0.43352700
C	5.64453400	-0.32225600	-0.50374700
C	4.62623600	-1.22078700	-0.28912100
H	-2.25024600	0.11229900	2.31848400
H	0.11782700	-3.15985600	0.95502300
H	-1.73569900	-2.28481200	2.32111200
H	2.43073900	-2.73065600	0.23445700
H	6.21331100	1.77112300	-0.60243300
H	6.64601300	-0.67805500	-0.72627600

H	1.58979900	2.16247900	0.41835900
C	-1.31780100	-0.85171000	-1.32971400
C	-1.04288800	-2.15114700	-0.81610500
C	-2.69396300	-0.54325400	-1.13488300
C	-2.40247900	-2.71273900	-0.46447100
O	-3.36953800	0.44260500	-1.35875200
O	-2.68279000	-3.83002900	-0.11154700
O	-3.31958100	-1.69820500	-0.53981300
N	-1.13898800	1.86476200	0.63458200
C	-2.47822800	2.29982600	1.10953900
C	-0.70225100	2.69877500	-0.50574800
C	-2.74881500	3.59563900	0.33644500
H	-3.21834500	1.53774900	0.85453500
C	-1.99195900	3.37300300	-0.97943000
H	0.02796100	3.44860400	-0.17597000
H	-3.81899200	3.76030200	0.19405700
H	-2.54745200	2.67824700	-1.61367700
H	-2.46320700	2.44386200	2.19496600
H	-2.33920300	4.45781200	0.87566300
H	-1.79432300	4.29700000	-1.52913500
H	-0.23124700	2.07092600	-1.26477100
H	4.81398000	-2.29004700	-0.33757500
H	-0.65246300	-0.21789000	-1.89382300
H	-0.37398600	-2.83458400	-1.33368500

ν_{imag} = -329.6 cm^{-1}

Zero-point correction= 0.363166 (Hartree/Particle)
Thermal correction to Energy= 0.382788
Thermal correction to Enthalpy= 0.383732
Thermal correction to Gibbs Free Energy= 0.315808
Sum of electronic and zero-point Energies= -1129.904216
Sum of electronic and thermal Energies= -1129.884594
Sum of electronic and thermal Enthalpies= -1129.883650
Sum of electronic and thermal Free Energies= -1129.951574

	E (Thermal)	CV	S
	KCal/Mol	Cal/Mol-Kelvin	Cal/Mol-Kelvin
Total	240.203	81.187	142.959

4. Transition state optimized structures for 5,8 addition with B3LYP-D3/6-31G**

4.1. 5,8-TS 1-Pyrrolidin-1-ylanthracen + DMAD

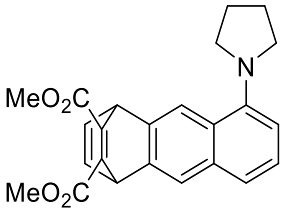

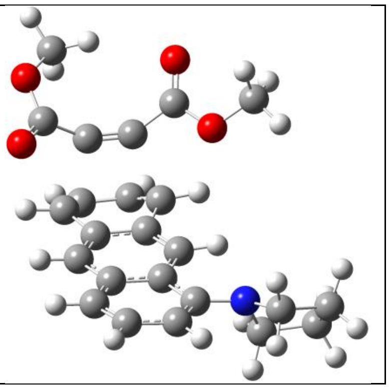

0 1

C	2.62053300	-0.67299200	2.57271600
C	2.20782500	-1.51969800	1.51692000
C	0.83543400	-1.43918800	1.04146400
C	0.20382300	-0.16267500	1.16621600
C	1.02719000	0.89957900	1.72415600
C	2.01977100	0.56512200	2.67626400
C	0.18442700	-2.46566600	0.37904900
C	-1.07353300	0.03282100	0.66827100
C	-1.78691800	-1.02698300	0.06998700
C	-1.12599000	-2.29230100	-0.11667000
C	-1.78738800	-3.32520900	-0.84018400
C	-3.03766300	-3.11028000	-1.36126900
C	-3.71775700	-1.88576300	-1.14149400
C	-3.13557900	-0.86383000	-0.41454000
H	0.69619300	-3.40882100	0.21144200
H	3.49988000	-0.92317200	3.15780900
H	2.74050700	-2.44844800	1.33604500
H	0.60992500	1.90170400	1.72263200
H	2.41017100	1.32583900	3.34483600
H	-1.51929400	1.02056700	0.69561500
H	-3.53251300	-3.89084600	-1.93189600
H	-4.72095200	-1.76888900	-1.53522100
C	-4.95341200	0.73903900	-0.95264600
C	-4.21149500	0.61623100	1.24399000
C	-5.36559600	2.08696900	-0.33812000
H	-5.78448900	0.01590400	-0.85328000
H	-4.68245100	0.79532600	-2.01078400
C	-4.93372600	1.97451000	1.15303400
H	-3.35560400	0.61830000	1.92088600

H	-4.90509500	-0.17159300	1.58733700
H	-4.82862000	2.90271500	-0.82981000
H	-6.43592300	2.27578700	-0.45697900
H	-4.25800600	2.79132000	1.42173400
H	-5.78427000	2.01544800	1.83881000
N	-3.79513000	0.35795100	-0.14766200
C	2.37978200	0.90438500	-0.00996100
C	2.94160200	-0.21814100	-0.10038900
H	-1.27519600	-4.27104300	-0.99033500
C	2.23209000	2.20606000	-0.64347600
C	3.74103800	-1.15916300	-0.88340400
O	3.29527300	-2.20340400	-1.30954100
O	5.03473800	-0.83417500	-1.10581700
O	3.15978600	2.89187300	-1.03403500
O	0.93626800	2.57517000	-0.74732700
C	0.71007400	3.82474800	-1.42341300
H	-0.37201200	3.95085800	-1.44611000
H	1.18480000	4.64744300	-0.88201200
H	1.11508900	3.79181400	-2.43801600
C	5.56101200	0.41454500	-0.61445600
H	5.01492600	1.27108500	-1.01680300
H	5.52879900	0.44942700	0.47923900
H	6.59827200	0.43518500	-0.95002900

ν_{imag} = -416.6 cm^{-1}

Zero-point correction= 0.420425 (Hartree/Particle)
Thermal correction to Energy= 0.446350
Thermal correction to Enthalpy= 0.447294
Thermal correction to Gibbs Free Energy= 0.362278
Sum of electronic and zero-point Energies= -1283.609638
Sum of electronic and thermal Energies= -1283.583713
Sum of electronic and thermal Enthalpies= -1283.582769
Sum of electronic and thermal Free Energies= -1283.667785

	E (Thermal)	CV	S
	KCal/Mol	Cal/Mol-Kelvin	Cal/Mol-Kelvin
Total	280.089	98.606	178.932

4.2. 5,8-endo-TS 1-Pyrrolidin-1-ylanthracen + maleic anhydride

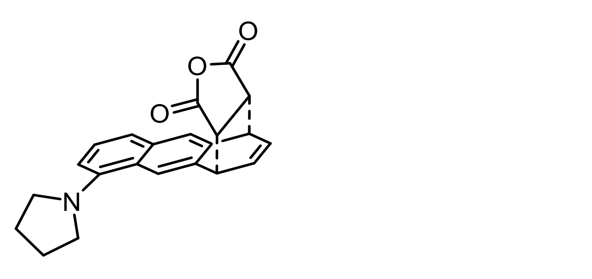

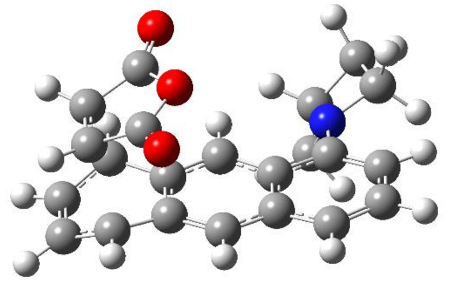

0 1

C	-2.95614900	2.12641500	0.14723900
C	-2.43934700	0.86006000	-0.05340200
C	-1.04762300	0.72082600	-0.40896200
C	-0.26704300	1.90448200	-0.65775900
C	-0.86255700	3.18514600	-0.47642500
C	-2.16371400	3.28444500	-0.05571400
C	-0.41466000	-0.53757700	-0.46052800
C	1.08793800	1.77118200	-1.02842900
C	1.67058800	0.52065900	-1.15608500
C	0.91132300	-0.64993500	-0.85000700
C	1.65676000	-1.90235400	-0.85357600
H	1.12606500	-2.79167500	-0.52385800
C	2.71223900	-2.03765200	-1.81021500
C	3.43884900	-0.91496000	-2.11018400
C	3.08242400	0.29826500	-1.44146800
H	-3.99155300	2.24837100	0.44354400
H	-0.26223900	4.07309100	-0.65075700
H	-2.61083600	4.26095600	0.10621600
H	1.68315800	2.66479700	-1.19332000
H	3.02102900	-3.02273800	-2.14569200
H	4.35166300	-0.96675600	-2.69528600
H	-0.96524600	-1.41856200	-0.15151100
C	2.92151700	-1.50743200	0.82066200
C	3.67046400	-0.34029100	0.51588200
N	-3.20540700	-0.31380000	0.10943900
C	-4.44785500	-0.26154200	0.87676200
C	-3.54647900	-1.08628700	-1.09988500
C	-4.95026700	-1.71313100	0.80731000
H	-4.25466700	0.09503300	1.89226000
C	-4.40630300	-2.24181300	-0.55206100
H	-4.13112600	-0.46222100	-1.79891300
H	-4.53367800	-2.29278700	1.63508000
H	-3.79839000	-3.13800700	-0.40068400
H	-5.19261800	0.41281100	0.41402800

H	-6.03991500	-1.76786100	0.87803000
H	-5.20604500	-2.50265100	-1.25046400
H	-2.65005900	-1.41751500	-1.62689600
H	3.70203900	1.18010200	-1.58187600
C	1.90305000	-1.11381600	1.82709500
C	3.10212700	0.76022800	1.33473200
O	3.45003700	1.90532400	1.43616400
O	1.09273200	-1.78368000	2.40734000
O	1.98162200	0.26893700	2.00817700
H	3.33257300	-2.50585300	0.87458200
H	4.73087200	-0.32610000	0.30611000

ν_{imag} = -501.4 cm^{-1}

Zero-point correction= 0.361901 (Hartree/Particle)
Thermal correction to Energy= 0.381822
Thermal correction to Enthalpy= 0.382766
Thermal correction to Gibbs Free Energy= 0.312963
Sum of electronic and zero-point Energies= -1129.880999
Sum of electronic and thermal Energies= -1129.861077
Sum of electronic and thermal Enthalpies= -1129.860133
Sum of electronic and thermal Free Energies= -1129.929937

	E (Thermal)	CV	S
	KCal/Mol	Cal/Mol-Kelvin	Cal/Mol-Kelvin
Total	239.597	81.134	146.915

4.3. 5,8-exo-TS 1-Pyrrolidin-1-ylanthracen + maleic anhydride

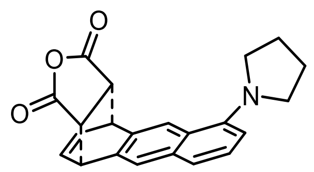

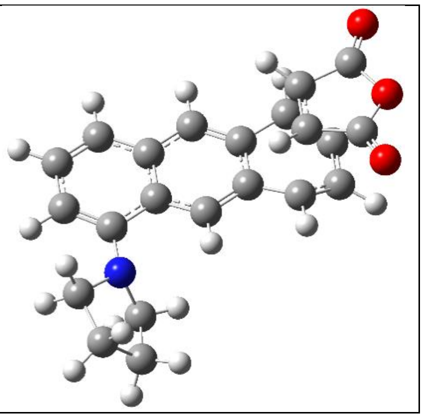

0 1

C	3.81795400	1.75614900	-0.60410400
C	3.07423700	0.64319000	-0.25588500
C	1.67458100	0.80685000	0.05436300
C	1.11966100	2.13390200	0.10629300
C	1.94308100	3.24882800	-0.21873800

C	3.24845900	3.05387000	-0.59236600
C	0.81940900	-0.29746500	0.25835600
C	-0.24467500	2.29694400	0.43638200
C	-1.04808700	1.20363200	0.70988700
C	-0.50976100	-0.11501200	0.60271100
C	-1.46930500	-1.20175800	0.77593700
H	-1.11797900	-2.21561700	0.60298200
C	-2.52535300	-1.00054300	1.71835200
C	-3.04024800	0.26560900	1.82778800
C	-2.47980200	1.27801900	0.98874600
H	4.86377300	1.64843400	-0.86715100
H	1.51528600	4.24660300	-0.18551400
H	3.87089100	3.90244400	-0.86096800
H	-0.66310600	3.29991400	0.47095400
H	-3.00929800	-1.85336500	2.18189100
H	-3.95127000	0.46287300	2.38231500
H	1.21071900	-1.29895600	0.11637400
C	-2.52975300	-0.79621800	-1.02033600
C	-3.06133400	0.51474100	-0.90950100
C	-3.66610600	-1.73793300	-0.87014300
C	-4.52214500	0.37242000	-0.69306300
O	-3.70119400	-2.93543500	-0.95642600
O	-5.38133200	1.20749500	-0.60946600
O	-4.80284100	-0.99008400	-0.54318300
N	3.61684600	-0.65982800	-0.22264400
C	4.86767300	-0.93338400	-0.92917400
C	3.79164100	-1.29434800	1.09948400
C	5.10332900	-2.42342700	-0.63560800
H	4.76577600	-0.69859100	-1.99247800
C	4.46015700	-2.64232400	0.76447100
H	4.45290500	-0.67718500	1.73236000
H	4.59851200	-3.03698600	-1.38653500
H	3.71937700	-3.44571200	0.72797400
H	5.70874400	-0.33791200	-0.52816200
H	6.16623100	-2.67783700	-0.65914700
H	5.19754100	-2.91317200	1.52471100
H	2.83895500	-1.39537400	1.62192300
H	-2.94369500	2.26089000	0.98499300
H	-2.69531200	1.37318100	-1.45562800
H	-1.70469500	-1.07109200	-1.66290600

ν_{imag} = -509.5 cm^{-1}

Zero-point correction= 0.362020 (Hartree/Particle)

Thermal correction to Energy= 0.381958
Thermal correction to Enthalpy= 0.382902
Thermal correction to Gibbs Free Energy= 0.312869
Sum of electronic and zero-point Energies= -1129.881641
Sum of electronic and thermal Energies= -1129.861703
Sum of electronic and thermal Enthalpies= -1129.860759
Sum of electronic and thermal Free Energies= -1129.930791

	E (Thermal)	CV	S
	KCal/Mol	Cal/Mol-Kelvin	Cal/Mol-Kelvin
Total	239.682	81.077	147.396
